# Estimating excess mortality due to the COVID-19 pandemic: a systematic analysis of COVID-19-related mortality, 2020–21

**DOI:** 10.1016/S0140-6736(21)02796-3

**Published:** 2022-04-16

**Authors:** COVID-19 Excess Mortality Collaborators, Haidong Wang, Katherine R Paulson, Spencer A Pease, Stefanie Watson, Haley Comfort, Peng Zheng, Aleksandr Y Aravkin, Catherine Bisignano, Ryan M Barber, Tahiya Alam, John E Fuller, Erin A May, Darwin Phan Jones, Meghan E Frisch, Cristiana Abbafati, Christopher Adolph, Adrien Allorant, Joanne O Amlag, Bree Bang-Jensen, Gregory J Bertolacci, Sabina S Bloom, Austin Carter, Emma Castro, Suman Chakrabarti, Jhilik Chattopadhyay, Rebecca M Cogen, James K Collins, Kimberly Cooperrider, Xiaochen Dai, William James Dangel, Farah Daoud, Carolyn Dapper, Amanda Deen, Bruce B Duncan, Megan Erickson, Samuel B Ewald, Tatiana Fedosseeva, Alize J Ferrari, Joseph Jon Frostad, Nancy Fullman, John Gallagher, Amiran Gamkrelidze, Gaorui Guo, Jiawei He, Monika Helak, Nathaniel J Henry, Erin N Hulland, Bethany M Huntley, Maia Kereselidze, Alice Lazzar-Atwood, Kate E LeGrand, Akiaja Lindstrom, Emily Linebarger, Paulo A Lotufo, Rafael Lozano, Beatrice Magistro, Deborah Carvalho Malta, Johan Månsson, Ana M Mantilla Herrera, Fatima Marinho, Alemnesh H Mirkuzie, Awoke Temesgen Misganaw, Lorenzo Monasta, Paulami Naik, Shuhei Nomura, Edward G O'Brien, James Kevin O'Halloran, Latera Tesfaye Olana, Samuel M Ostroff, Louise Penberthy, Robert C Reiner Jr, Grace Reinke, Antonio Luiz P Ribeiro, Damian Francesco Santomauro, Maria Inês Schmidt, David H Shaw, Brittney S Sheena, Aleksei Sholokhov, Natia Skhvitaridze, Reed J D Sorensen, Emma Elizabeth Spurlock, Ruri Syailendrawati, Roman Topor-Madry, Christopher E Troeger, Rebecca Walcott, Ally Walker, Charles Shey Wiysonge, Nahom Alemseged Worku, Bethany Zigler, David M Pigott, Mohsen Naghavi, Ali H Mokdad, Stephen S Lim, Simon I Hay, Emmanuela Gakidou, Christopher J L Murray

## Abstract

**Background:**

Mortality statistics are fundamental to public health decision making. Mortality varies by time and location, and its measurement is affected by well known biases that have been exacerbated during the COVID-19 pandemic. This paper aims to estimate excess mortality from the COVID-19 pandemic in 191 countries and territories, and 252 subnational units for selected countries, from Jan 1, 2020, to Dec 31, 2021.

**Methods:**

All-cause mortality reports were collected for 74 countries and territories and 266 subnational locations (including 31 locations in low-income and middle-income countries) that had reported either weekly or monthly deaths from all causes during the pandemic in 2020 and 2021, and for up to 11 year previously. In addition, we obtained excess mortality data for 12 states in India. Excess mortality over time was calculated as observed mortality, after excluding data from periods affected by late registration and anomalies such as heat waves, minus expected mortality. Six models were used to estimate expected mortality; final estimates of expected mortality were based on an ensemble of these models. Ensemble weights were based on root mean squared errors derived from an out-of-sample predictive validity test. As mortality records are incomplete worldwide, we built a statistical model that predicted the excess mortality rate for locations and periods where all-cause mortality data were not available. We used least absolute shrinkage and selection operator (LASSO) regression as a variable selection mechanism and selected 15 covariates, including both covariates pertaining to the COVID-19 pandemic, such as seroprevalence, and to background population health metrics, such as the Healthcare Access and Quality Index, with direction of effects on excess mortality concordant with a meta-analysis by the US Centers for Disease Control and Prevention. With the selected best model, we ran a prediction process using 100 draws for each covariate and 100 draws of estimated coefficients and residuals, estimated from the regressions run at the draw level using draw-level input data on both excess mortality and covariates. Mean values and 95% uncertainty intervals were then generated at national, regional, and global levels. Out-of-sample predictive validity testing was done on the basis of our final model specification.

**Findings:**

Although reported COVID-19 deaths between Jan 1, 2020, and Dec 31, 2021, totalled 5·94 million worldwide, we estimate that 18·2 million (95% uncertainty interval 17·1–19·6) people died worldwide because of the COVID-19 pandemic (as measured by excess mortality) over that period. The global all-age rate of excess mortality due to the COVID-19 pandemic was 120·3 deaths (113·1–129·3) per 100 000 of the population, and excess mortality rate exceeded 300 deaths per 100 000 of the population in 21 countries. The number of excess deaths due to COVID-19 was largest in the regions of south Asia, north Africa and the Middle East, and eastern Europe. At the country level, the highest numbers of cumulative excess deaths due to COVID-19 were estimated in India (4·07 million [3·71–4·36]), the USA (1·13 million [1·08–1·18]), Russia (1·07 million [1·06–1·08]), Mexico (798 000 [741 000–867 000]), Brazil (792 000 [730 000–847 000]), Indonesia (736 000 [594 000–955 000]), and Pakistan (664 000 [498 000–847 000]). Among these countries, the excess mortality rate was highest in Russia (374·6 deaths [369·7–378·4] per 100 000) and Mexico (325·1 [301·6–353·3] per 100 000), and was similar in Brazil (186·9 [172·2–199·8] per 100 000) and the USA (179·3 [170·7–187·5] per 100 000).

**Interpretation:**

The full impact of the pandemic has been much greater than what is indicated by reported deaths due to COVID-19 alone. Strengthening death registration systems around the world, long understood to be crucial to global public health strategy, is necessary for improved monitoring of this pandemic and future pandemics. In addition, further research is warranted to help distinguish the proportion of excess mortality that was directly caused by SARS-CoV-2 infection and the changes in causes of death as an indirect consequence of the pandemic.

**Funding:**

Bill & Melinda Gates Foundation, J Stanton, T Gillespie, and J and E Nordstrom

## Introduction

An accurate measurement of the number of deaths due to the COVID-19 pandemic is crucial for each country and region to understand the magnitude of the pandemic's impact on public health. Accurate measurement of death is also needed to understand the determinants of variation in the infection–fatality ratio across populations, and is a direct input into forecasting the pandemic and investigating alternative policy options. Reported deaths attempt to quantify the magnitude of the COVID-19 pandemic in different populations and locations over time, and they are widely seen as a more reliable indicator for tracking the pandemic relative to reported case rates.^1^ However, reported deaths represent only a partial count of total death tolls from the COVID-19 pandemic, and the reliability of reported deaths varies greatly between locations and over time.

**Research in context**

**Evidence before this study**

Many attempts have been made to estimate the total number of deaths related to the COVID-19 pandemic. WHO estimated a total of 3 million excess deaths due to the COVID-19 pandemic during the year 2020, although they have not made available excess mortality estimates by location. Numerous studies have examined excess mortality due to COVID-19 for specific countries or subnational units during different stages of the pandemic. Various models have been proposed to estimate expected mortality based on past rates and trends for all-cause mortality. Such modelling approaches include simple count models with fixed effects on week and year; time series-type models including autoregressive integrated moving average (ARIMA) and Holt-Winters models; and simpler assumptions that expected mortality during the pandemic can be assumed to equate to average mortality rates in 2015–19. The World Mortality Dataset provides regularly updated excess mortality estimates for 117 countries and subnational units for a subset of the countries; expected mortality in this dataset is modelled with a linear regression on weekly, monthly, or quarterly death counts with a dummy variable on week, month, or quarter, and year as a continuous variable. *The Economist*, whose analytical model has not been peer-reviewed, provides the most comprehensive assessment of excess mortality due to COVID-19 to date; they used a machine learning algorithm with a vast list of covariates to produce excess mortality estimates for 187 countries. They provide a global estimate of 18·0 million (95% uncertainty interval 12·9–21·0) excess deaths for the period of Jan 1, 2020, to Dec 27, 2021.

**Added value of this study**

This study makes several important contributions to understanding the full impact of the COVID-19 pandemic on mortality. First, we corrected all-cause mortality data for lags in reporting and for under-registration of death. Second, we excluded weeks with heat waves, for which excess mortality due to COVID would be overestimated due to the large increase in observed mortality from excessive heat during the pandemic. Third, we used an ensemble of six models to predict the expected mortality rate in the absence of COVID-19. Weights in the ensemble were based on the out-of-sample predictive validity of each model. Fourth, for locations without weekly or monthly reported all-cause mortality data, we developed a statistical model that captured the relationship between the key COVID-19-related covariates, such as seroprevalence, infection-detection ratio, and other population-level burden of disease metrics, and excess mortality rate. Fifth, to evaluate the robustness of the model predicting excess mortality, we conducted out-of-sample predictive validity testing, which indicated a small error rate (0·85%). Sixth, by looking comprehensively across countries, we were able to generate estimates of excess mortality for 191 countries and territories, in addition to internally consistent regional and global aggregates. These estimates will form part of the next iteration of the Global Burden of Diseases, Injuries, and Risk Factors Study.

**Implications of all the available evidence**

Our estimates of COVID-19 excess mortality suggest the mortality impact from the COVID-19 pandemic has been more devastating than the situation documented by official statistics. Official statistics on reported COVID-19 deaths provide only a partial picture of the true burden of mortality. The difference between excess mortality and reported COVID-19 deaths might be a function of underdiagnosis due to insufficient testing, reporting challenges, or higher than expected mortality from other diseases due to pandemic-related changes in behaviours or reduced access to health care or other essential services. The gap between estimated excess mortality and reported COVID-19 deaths is much larger in south Asia and sub-Saharan Africa than in other regions. The differences shown between reported COVID-19 mortality and COVID-19-related excess mortality highlight the importance of the use of COVID-19-related excess mortality estimates in policy and in monitoring and evaluation efforts.

Excess mortality due to the COVID-19 pandemic, defined as the net difference between the number of deaths during the pandemic (measured by observed or estimated all-cause mortality) and the number of deaths that would be expected on the basis of past trends in all-cause mortality, is therefore a crucial measure of the true toll of the COVID-19 pandemic. Gaps exist between reported and excess deaths related to the COVID-19 pandemic for a number of reasons. First, health-care reporting systems generally do not list COVID-19 as the cause of death without a positive SARS-CoV-2 test, and thus deaths due to COVID-19 will be missed in official counts in locations with low testing capacity. For example, early in the pandemic, before tests were widely available, many deaths due to COVID-19 among older individuals in high-income countries, particularly in long-term care facilities, are unlikely to have been attributed to COVID-19,^2^ with evidence of extensive under-reporting in many locations.^3^, ^4^, ^5^, ^6^ Second, country systems for registering deaths vary in their quality and compre-hensiveness, as well as in the definitions used for counting COVID-19 deaths. Third, among the global medical community, there is no universal agreement as to when a death of someone infected with SARS-CoV-2 should be reported as a death due to COVID-19. Fourth, other factors including political considerations appear to have prevented accurate reporting of deaths in some locations. Fifth, the magnitude and distribution of many other causes of death might have changed because of social, economic, and behavioural responses to the pandemic, including strict lockdowns. Under these conditions, excess mortality can provide a more accurate assessment of the total mortality impact of the COVID-19 pandemic than reported COVID-19 deaths.

Several attempts have been made to estimate excess mortality from COVID-19, although these have generally been limited in geographical scope by the availability of data. WHO has estimated regional excess mortality for the Americas and Europe but has not yet produced country-level estimates for these regions.^7^ Other country-specific or location-specific studies have evaluated the effect of the COVID-19 pandemic on all-cause mortality for specific locations during different stages of the pandemic.^8^, ^9^, ^10^ Such studies have used a variety of models to estimate expected mortality during the pandemic on the basis of past rates and trends in all-cause mortality, making direct comparisons of excess mortality between locations difficult. *The Economist*, whose estimation model has not been peer-reviewed, produces the only other set of COVID-19-related excess mortality estimates for a uniform time period for a total of 187 countries included in their analysis regardless of vital registration data availability.

In addition to deaths from SARS-CoV-2 infection, social distancing mandates and other pandemic restrictions might have decreased deaths from some diseases and injuries, such as road accidents,^11^, ^12^, ^13^ and increased others, such as deaths from chronic and acute conditions affected by deferred care-seeking in overstretched health-care systems,^14^, ^15^ relative to expected or baseline conditions. Such changes to baseline patterns of disease and injury death affect the excess mortality from the pandemic; differentiating how much excess mortality is due to SARS-CoV-2 infection and how much is due to other societal, economic, or behavioural changes associated with the pandemic is challenging, especially without detailed data on specific causes of death in many countries. Although separating out the contributors to excess mortality will be extremely important, understanding the total mortality impact of the pandemic is a crucial first step.

Our objective in this study was to estimate, globally and for each of the 191 countries and territories (and 252 subnational units for a subset of nations) included in our analysis, excess deaths due to the pandemic from Jan 1, 2020, to Dec 31, 2021. We leveraged data from locations where all-cause mortality data were available from the periods before and during the pandemic to estimate excess mortality for the study period; we then explored the statistical relationship between excess mortality rate and key covariates including COVID-19 seroprevalence, infection–detection ratio (IDR), and other population health-related metrics such as the Healthcare Access and Quality (HAQ) Index.^16^ We used this model to generate location-specific and global estimates of excess deaths due to the COVID-19 pandemic over the study period. Our excess mortality estimates reflect the full impact of the pandemic on mortality around the world, and not only deaths directly attributable to SARS-CoV-2 infection.

## Methods

### Overview

Our analysis of excess mortality due to the COVID-19 pandemic from Jan 1, 2020, to Dec 31, 2021, followed four key steps. First, we developed a database of all-cause mortality by week and month after accounting for reporting lags, anomalies such as heat waves, and under-registration of death. Second, we developed an ensemble model to predict expected deaths in the absence of the COVID-19 pandemic for years 2020 and 2021. In location and time combinations with data meeting our inclusion criteria, excess mortality was estimated as observed mortality minus expected mortality. Third, to estimate excess mortality due to COVID-19 over the entire study period for all countries and territories in the analysis, and especially for locations without weekly or monthly all-cause mortality data, we developed a statistical model to directly predict the excess mortality due to COVID-19, using covariates that pertained to both the COVID-19 pandemic and background population health-related metrics at the population level before SARS-CoV-2 emerged. Fourth, we estimated excess mortality due to COVID-19, propagating uncertainty from each step. Further details on each of the analytical steps required to estimate COVID-19-related excess deaths are presented herein.

This study complies with the Guidelines for Accurate and Transparent Health Estimates Reporting^17^ statement (appendix pp 3–4). Analyses were completed with Stata (version 17), Python version (3.7.7), and R (version 4.0.3). Statistical code used for these analyses can be found online. Results specific to the model run for this publication are accessible for each location online. The estimates viewable in our online tool will be iteratively updated as new data are incorporated and will ultimately supersede the results in this paper. The data used as inputs in these analyses are available for download on the Global Health Data Exchange website. These data include those available in public online repositories and those made available on request from the data provider. Data sources for reported COVID-19 mortality are also listed by location in the appendix (pp 8–37).

All maps presented in this study were generated by the authors using RStudio (R version 4.0.3) and ArcGIS Desktop (version 10.6.1), and no permissions were required to publish them.

### Database of all-cause mortality

We searched government websites, compendia developed by the World Mortality Database,^18^ the Human Mortality Database, and the European Statistical Office, and identified 74 countries and territories and an additional 266 subnational locations where either weekly or monthly all-cause mortality data were reported for periods in 2020 and 2021, and for up to 11 years previously (appendix pp 4–8, 47). These locations included 31 locations in low-income and middle-income countries. In addition to the time-detailed all-cause mortality data that are publicly available for the estimation of excess mortality, we also included empirical assessments of excess mortality for the nine provinces of South Africa (in addition to all-cause mortality data at the national level) and 12 states of India (in lieu of state-level all-cause mortality data). The Medical Research Council of South Africa used the data from both the civil registration system and the Rapid Mortality Surveillance Report to produce timely excess mortality estimates at the national and provincial levels (all nine provinces). For India, empirical assessment of excess mortality for 12 states used data from the civil registration system. For different months during the first and second waves of the COVID-19 epidemic in the 12 states in India, total numbers of deaths for those states during the corresponding months were made available (appendix pp 7–8). Using the mean reported deaths during the same periods in years 2018 and 2019, we were able to generate excess mortality rates for those Indian states after accounting for under-registration of mortality by the civil registration system at the state level.

**Figure 1:**
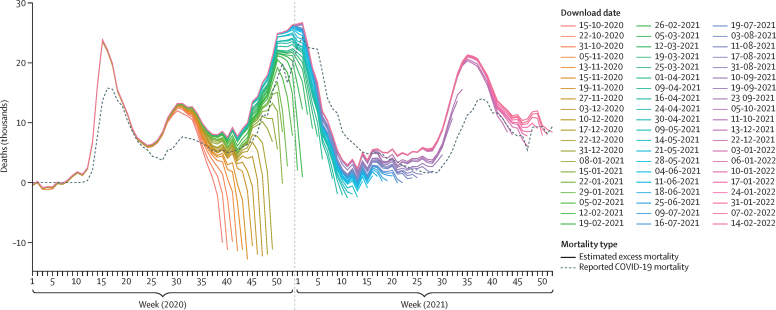
Late registration in all-cause mortality and its effect on computed excess mortality over time, USA Reported COVID-19 deaths (coloured lines) and estimated number of excess deaths due to the COVID-19 pandemic (dashed line) by week from series of weekly mortality data reported by calendar week from October, 2020, to February, 2022. The gap between the first calendar week during which mortality for a past week was reported and the subsequent reported levels indicates the gradual process by which completeness of reported deaths increases over time. Lines below 0 indicate reported deaths below the expected deaths value.

We identified three types of data issues. First, all-cause mortality from weekly or monthly reporting often has considerable lags in reporting (appendix pp 51–84). Figure 1 shows the effect of lags in reporting of all-cause mortality on excess mortality for the vital registration system in the USA; it takes approximately 20 weeks for data in the USA to be nearly complete. We systematically reviewed input vital registration data and trimmed periods that were likely to be subject to late registration for all locations in the analysis. As these data have been made public only recently, we had limited information by which to identify the precise lag time in late registration; this was particularly true for locations where data are made available more sporadically and by month. We therefore took a conservative approach and excluded data from more recent weeks and months in 2021 in our calculation of excess mortality.

Second, in many European countries, registered deaths spiked in late July and early August (calendar weeks 31–33) of 2020, which was a period when reported COVID-19 deaths were extremely low as reported in our online tool. This period coincided with a heat wave; such spikes in all-cause mortality have been observed in Europe during similar timeframes in previous years.^19^, ^20^, ^21^ Because our model cannot separate excess mortality due to COVID-19 from excess deaths occurring during a heat wave, we excluded these weeks of data for all countries in western Europe (classified according to the Global Burden of Diseases, Injuries, and Risk Factors Study [GBD] location groupings) from subsequent analyses to avoid potentially exaggerating the impact of COVID-19 on all-cause mortality during 2020.

Third, as under-reporting of deaths is common in most vital registration systems, especially those from outside the high-income group of countries, we corrected all-cause mortality data for under-registration of death as estimated in GBD 2019;^22^ six countries were corrected because GBD estimates suggested registration was less than 95% for the calendar year 2019. This under-reporting correction was in addition to, and distinct from, the late registration issue.

### Estimating expected mortality with an ensemble model

To estimate expected mortality, we developed six models, each fit separately by location. The first four models were based on first estimating the weekly (or monthly) seasonal pattern of mortality and then estimating the time trend in weekly or monthly mortality not explained by seasonality. We used a Bayesian spline to estimate the weekly seasonal pattern for each location using data from 2010, or the earliest year after 2010 when such data first became available, until around February, 2020, when the COVID-19 pandemic started for each location (appendix p 48). Second, using the same Bayesian spline, we estimated the time trend in the residuals (additional details provided in the appendix, pp 38–40). By combining the seasonal and secular trends, we generated predictions of the expected level of mortality in 2020 and 2021.

The specification of the spline can have a sizeable impact on the estimated expected mortality for a particular location. To make the results more robust to model specification, we included in our ensemble four variants according to where the second to last knot in the spline was placed: 6 months, 12 months, 18 months, and 24 months before the end of the period for the input data before the COVID-19 pandemic started for each location. We also included in the ensemble a Poisson model with fixed effects on week and year, and a model that assumed that expected mortality for 2020 and 2021 was the same as the corresponding weekly mortality observed in 2019. To derive weights for the different models in the ensemble, we assessed how each model performed in an out-of-sample predictive validity test. We fit the model to all data prior to March 1, 2019 and then evaluated how each model performed in predicting mortality between March, 2019, and February, 2020, compared with observed mortality in the same time period. We then weighted component models in the ensemble using 1 over the root mean squared error (RMSE) of the predictions for each component to down-weight component models with larger RMSE (and thus less accurate predictions) in the ensemble. A global weighting scheme was used for all locations. The distribution of RMSE by location for each of the six models included in the model ensemble and examples of the estimated excess mortality for each component model are provided in the appendix (p 49). Expected mortality from the ensemble model was subtracted from observed mortality in 2020 and 2021 to estimate excess mortality due to the COVID-19 pandemic.

### Predicting excess mortality due to the COVID-19 pandemic

**Figure 2:**
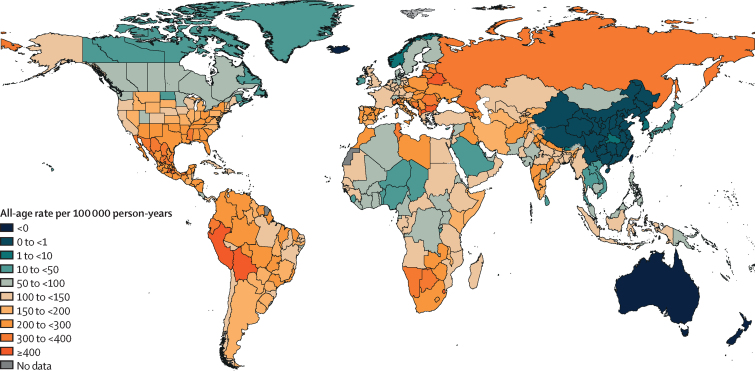
Global distribution of estimated excess mortality rate due to the COVID-19 pandemic, for the cumulative period 2020–21

Based on our estimation of expected mortality, and after applying the exclusions that account for late registration and other anomalies as previously described, we generated excess mortality estimates for each location where we had input data (figure 2). In addition, we added excess mortality estimates from two countries where the ensemble model could not be applied due to data constraints: South Africa (only the provinces) and India. We obtained national and province-level excess mortality rate estimates for South Africa which are regularly updated by the Medical Research Council of South Africa, and excess mortality estimates for select periods during the first and second waves of the COVID-19 epidemic for 12 states in India. Using these empirical excess mortality estimates, we developed a statistical model to predict excess mortality for all 191 national and 252 subnational locations in our analysis for the uniform period of Jan 1, 2020, to Dec 31, 2021 (appendix pp 40–43). This model was crucial for directly estimating excess mortality in countries where reported all-cause mortality data have not been available during the pandemic.

Various studies have examined the associations between particular underlying medical conditions and increased risk of severe COVID-19. We examined all available and relevant covariates on the basis of a meta-analysis conducted by the US Centers for Disease Control and Prevention,^23^ as well as covariates directly related to the COVID-19 pandemic, including seroprevalence (lagged by 25 days), mobility (lagged by 19 days), IDR (lagged by 19 days), and reported crude death rate due to COVID-19. For the model to be parsimonious, we used least absolute shrinkage and selection operator (LASSO) regression to help identify covariates that have sensible direction of effect on excess mortality rate (appendix pp 41–43). With the variable selection done via LASSO with input of mean level excess mortality and covariates, the following 15 covariates were included in our final log-linear model, where the dependent variable was excess mortality in logarithmic scale: lagged cumulative infection rate (seroprevalence) in log space, COVID-19 mortality rate in log space, crude death rate in log space, lagged IDR, annual inpatient admissions per capita, diabetes mortality rate in log space, HIV mortality rate in log space, lagged mobility, binned quality of vital registration data, average absolute latitude, cardiovascular disease mortality rate in log space, smoking prevalence, HAQ Index, proportion of the population aged 75 years or older, and universal health care coverage. These covariates explained 69·1% of the variation in the input data to this regression. We also calculated in-sample residuals for the locations that had directly observed excess mortality rates used in the regression (ie, India and South Africa). Regional and super-regional residuals, per the GBD regional classification system, were calculated as the mean residuals from locations included in each regional aggregate. We also calculated a country-level residual for India using the residual from the 12 states to recognise the dispersed periods and geographical regions covered by the civil registration data. Further details on the methodology are provided in the appendix (p 38–43). To validate our modelling process, we conducted out-of-sample predictive validity testing. Given the sparsity of input data on empirical excess mortality, we validated our model by repeatedly leaving one location out of the input data, then re-estimated the model and made predictions for the left-out location. Our analysis showed that the mean relative error of predicted excess mortality rate is 0·85% and the root mean squared error of predicted excess mortality rate is 0·00072, indicating a precise prediction model that has low bias.

In predicting excess mortality for all locations for the uniform period of Jan 1, 2020, to Dec 31, 2021, we used estimated coefficients and residuals from different aggregate levels together with all selected COVID-related covariates for the same period and other population health-related covariates for the year 2019 (appendix p 43). Ratios of estimated excess mortality to reported COVID-19 deaths were computed using the modelled excess mortality and the reported COVID-19 counts for each location.

As noted previously, policy makers and researchers have proposed many other causes of death that might have been affected by lockdown restrictions, unemployment, and increased poverty. To date, insufficient data are available to widely test for increases in deaths due to pandemic-related elements, such as deferred care, or for reductions in deaths, such as decreases in injury-related deaths due to reduced mobility.

### COVID-19 excess deaths with uncertainty

To fully account for uncertainties in both the input empirical excess mortality and the selected covariates, 100 draws of excess mortality for each location were produced by use of 100 draws of coefficients of covariates and residuals and 100 draws of all selected covariates. Draw-level coefficients and residuals were obtained by repeating the excess mortality model 100 times with use of draw-level input excess mortality and covariates. Mean excess mortality with 95% uncertainty intervals [UIs] were generated on the basis of the draw-level estimates (appendix p 43).

Predicted excess mortality rates were used to compute the number of COVID-19-related excess deaths by multiplying the predicted excess mortality rate by person-years of exposure for the period. We used estimates of population size by location from the GBD, which by definition are the total person-years of exposure for a calendar year.

### Role of the funding source

The funders of the study had no role in study design, data collection, data analysis, data interpretation, or the writing of the report.

## Results

By Dec 31, 2021, global reported deaths due to COVID-19 reached 5·94 million, but the estimated number of excess deaths was nearly 3·07-times (95% UI 2·88–3·30) greater, reaching 18·2 million (17·1–19·6). The global all-age rate of excess mortality due to the COVID-19 pandemic was 120·3 deaths (113·1–129·3) per 100 000 of the population.

The table provides summary statistics for each country and territory, including reported deaths due to COVID-19, estimated excess deaths due to the COVID-19 pandemic, the ratio of the two, and reported and excess all-age COVID-19 mortality rates. The magnitude of the excess mortality burden has varied substantially between countries. The highest estimated excess mortality rate due to COVID-19 was 734·9 deaths (95% UI 594·1–879·2) per 100 000 of the population in Bolivia (compared with the global rate of 120·3 [113·1–129·3]), whereas negative excess mortality rates were estimated in Iceland, Australia, Singapore, New Zealand, and Taiwan (province of China), representing a large range. Excess mortality rates exceeded 300 deaths per 100 000 in 21 countries. At the regional level, the highest estimated excess mortality rates were in Andean Latin America, eastern Europe, central Europe, southern sub-Saharan Africa, and central Latin America, with several locations outside these regions having similarly high rates, particularly Lebanon, Armenia, Tunisia, Libya, several regions in Italy, and several states in southern USA (figure 2, table). Rates varied substantially in western Europe; some countries had rates nearly as high as the locations listed previously, while others, such as Iceland, Norway, Ireland, and Cyprus, had some of the lowest rates in the world, at less than 50 deaths per 100 000. Mortality rates varied across north Africa and the Middle East, with high rates in Lebanon (416·2 deaths [347·4–515·5] per 100 000), Tunisia (324·2 [265·4–376·4] per 100 000), Libya (292·1 [232·2–358·8] per 100 000), and Iraq (280·1 [216·7–362·5] per 100 000). Low excess mortality rates were estimated across sub-Saharan Africa, with the notable exception of four nations in southern sub-Saharan Africa: Eswatini (634·9 [491·8–775·4] per 100 000), Lesotho (562·9 [460·8–691·4] per 100 000), Botswana (399·5 [329·1–482·1] per 100 000), and Namibia (395·6 [340·2–448·8] per 100 000). In south Asia, some states in India had excess mortality rates due to COVID-19 similar to those of some high-income countries in the northern hemisphere.

**Table:** Number of reported COVID-19 deaths and excess deaths due to the pandemic, reported and excess mortality rates, and the ratio of excess to reported mortality, 2020–21, globally and for GBD super-regions, regions, countries and territories, and select subnational locations

			**Reported COVID-19 deaths**	**Reported COVID-19 mortality rate (per 100 000)**	**Estimated excess deaths**	**Estimated excess mortality rate (per 100 000)**	**Ratio between excess mortality rate and reported COVID-19 mortality rate**
**Global**			**5 940 000**	**39·2**	**18 200 000 (17 100 000 to 19 600 000)**	**120·3 (113·1 to 129·3)**	**3·07 (2·88 to 3·30)**
**Central Europe, eastern Europe, and central Asia**			**1 170 000**	**148·2**	**2 340 000 (2 310 000 to 2 380 000)**	**296·5 (291·9 to 300·5)**	**2·00 (1·97 to 2·03)**
	**Central Asia**		**52 800**	**30·2**	**281 000 (271 000 to 292 000)**	**160·6 (155·0 to 166·8)**	**5·32 (5·14 to 5·53)**
	Armenia		7970	140·9	20 600 (19 600 to 21 900)	364·4 (347·0 to 387·6)	2·59 (2·46 to 2·75)
	Azerbaijan		8360	42·4	53 500 (51 400 to 55 600)	271·5 (260·8 to 282·2)	6·40 (6·15 to 6·66)
	Georgia		13 800	203·0	15 800 (14 100 to 18 000)	233·1 (207·6 to 264·4)	1·15 (1·02 to 1·30)
	Kazakhstan		12 900	37·1	49 000 (46 800 to 50 900)	141·0 (134·8 to 146·7)	3·80 (3·64 to 3·96)
	Kyrgyzstan		2800	22·4	22 600 (21 200 to 23 900)	180·2 (169·4 to 190·4)	8·06 (7·58 to 8·52)
	Mongolia		2060	33·4	5600 (4540 to 6430)	90·9 (73·7 to 104·4)	2·72 (2·21 to 3·13)
	Tajikistan		254	1·4	29 400 (28 300 to 30 500)	160·6 (155·0 to 166·8)	115·80 (111·78 to 120·29)
	Turkmenistan		3180	33·5	15 200 (14 700 to 15 800)	160·6 (155·0 to 166·8)	4·79 (4·62 to 4·97)
	Uzbekistan		1480	2·4	69 100 (66 000 to 73 400)	112·3 (107·4 to 119·3)	46·51 (44·47 to 49·41)
	**Central Europe**		**337 000**	**157·8**	**674 000 (657 000 to 687 000)**	**315·7 (308·0 to 322·0)**	**2·00 (1·95 to 2·04)**
	Albania		3220	64·4	17 300 (16 500 to 18 100)	346·5 (331·1 to 361·3)	5·38 (5·14 to 5·61)
	Bosnia and Herzegovina		13 400	214·8	20 900 (19 600 to 22 000)	333·9 (313·8 to 351·7)	1·55 (1·46 to 1·64)
	Bulgaria		31 000	243·0	82 500 (81 200 to 83 800)	647·3 (637·6 to 658·2)	2·66 (2·62 to 2·71)
	Croatia		12 500	156·7	22 900 (22 100 to 23 800)	285·6 (275·7 to 297·6)	1·82 (1·76 to 1·90)
	Czechia		36 100	180·1	49 100 (47 000 to 51 000)	244·8 (234·5 to 254·5)	1·36 (1·30 to 1·41)
	Hungary		39 200	217·0	53 800 (52 400 to 55 200)	297·8 (290·2 to 305·5)	1·37 (1·34 to 1·41)
	Montenegro		2410	210·9	4080 (3670 to 4470)	357·0 (321·2 to 390·9)	1·69 (1·52 to 1·85)
	North Macedonia		7960	229·9	20 200 (19 100 to 21 200)	583·6 (552·9 to 613·8)	2·54 (2·40 to 2·67)
	Poland		97 100	135·0	214 000 (210 000 to 218 000)	297·2 (291·7 to 302·7)	2·20 (2·16 to 2·24)
	Romania		58 800	161·9	119 000 (115 000 to 125 000)	328·7 (318·1 to 345·2)	2·03 (1·97 to 2·13)
	Serbia		12 700	77·7	37 400 (35 200 to 40 700)	228·9 (215·4 to 248·7)	2·95 (2·77 to 3·20)
	Slovakia		16 600	163·7	25 400 (23 800 to 26 600)	250·4 (234·6 to 261·5)	1·53 (1·43 to 1·60)
	Slovenia		5590	144·0	6980 (6070 to 7830)	179·9 (156·5 to 201·8)	1·25 (1·09 to 1·40)
	**Eastern Europe**		**783 000**	**194·4**	**1 390 000 (1 370 000 to 1 410 000)**	**345·2 (340·6 to 349·5)**	**1·78 (1·75 to 1·80)**
	Belarus		5580	31·5	85 600 (81 600 to 93 400)	483·1 (460·7 to 527·0)	15·34 (14·63 to 16·74)
	Estonia		1930	77·8	5630 (5180 to 6180)	226·7 (208·3 to 248·9)	2·92 (2·68 to 3·20)
	Latvia		4570	129·3	12 400 (11 700 to 13 100)	352·0 (332·1 to 371·5)	2·72 (2·57 to 2·87)
	Lithuania		7390	142·5	20 000 (19 200 to 20 800)	385·0 (370·3 to 400·4)	2·70 (2·60 to 2·81)
	Moldova		10 300	152·8	16 500 (15 100 to 18 100)	245·2 (223·9 to 268·8)	1·60 (1·47 to 1·76)
	Russia		651 000	228·1	1 070 000 (1 060 000 to 1 080 000)	374·6 (369·7 to 378·4)	1·64 (1·62 to 1·66)
	Ukraine		102 000	125·0	181 000 (177 000 to 186 000)	221·7 (217·0 to 228·2)	1·77 (1·74 to 1·83)
**High income**			**1 840 000**	**87·9**	**2 640 000 (2 520 000 to 2 750 000)**	**125·8 (120·2 to 131·1)**	**1·43 (1·37 to 1·49)**
	**Australasia**		**2300**	**4·0**	**−19 000 (−22 200 to −15 200)**	**−32·9 (−38·6 to −26·4)**	**−8·23 (−9·65 to −6·60)**
	Australia		2250	4·7	−18 100 (−21 400 to −14 400)	−37·6 (−44·5 to −29·9)	−8·03 (−9·52 to −6·39)
	New Zealand		51	0·5	−872 (−1330 to −451)	−9·3 (−14·1 to −4·8)	−17·10 (−26·06 to −8·84)
	**High-income Asia Pacific**		**24 900**	**6·8**	**114 000 (103 000 to 124 000)**	**30·9 (28·1 to 33·8)**	**4·56 (4·15 to 4·99)**
	Brunei		98	11·7	132 (44 to 217)	15·8 (5·2 to 25·9)	1·35 (0·44 to 2·22)
	Japan		18 400	7·3	111 000 (103 000 to 116 000)	44·1 (41·0 to 46·4)	6·02 (5·58 to 6·33)
	Singapore		828	7·4	−1770 (−2360 to −1090)	−15·8 (−21·0 to −9·8)	−2·14 (−2·85 to −1·32)
	South Korea		5620	5·4	4630 (−658 to 9580)	4·4 (−0·6 to 9·1)	0·82 (−0·12 to 1·70)
	**High-income North America**		**855 000**	**121·5**	**1 180 000 (1 120 000 to 1 230 000)**	**167·1 (158·9 to 174·9)**	**1·38 (1·31 to 1·44)**
	Canada		30 300	41·9	43 700 (39 900 to 47 300)	60·5 (55·2 to 65·4)	1·44 (1·32 to 1·56)
		Alberta	3310	40·3	5560 (4660 to 6470)	67·7 (56·7 to 78·8)	1·68 (1·41 to 1·96)
		British Columbia	2420	24·2	5260 (4350 to 6250)	52·5 (43·4 to 62·3)	2·17 (1·80 to 2·58)
		Manitoba	1390	54·5	2450 (1840 to 3110)	95·8 (72·1 to 122·0)	1·76 (1·32 to 2·24)
		New Brunswick	160	11·1	391 (259 to 494)	27·0 (17·9 to 34·2)	2·44 (1·62 to 3·09)
		Newfoundland and Labrador	19	2·0	106 (62 to 145)	11·0 (6·4 to 15·0)	5·59 (3·26 to 7·62)
		Northwest Territories	12	14·4	32 (26 to 37)	38·1 (31·6 to 44·8)	2·64 (2·19 to 3·10)
		Nova Scotia	111	6·1	327 (208 to 431)	18·0 (11·5 to 23·8)	2·95 (1·87 to 3·88)
		Nunavut	4	8·3	13 (10 to 16)	27·7 (21·4 to 32·4)	3·35 (2·59 to 3·92)
		Ontario	10 200	35·3	15 600 (13 000 to 17 500)	53·9 (44·9 to 60·8)	1·53 (1·27 to 1·72)
		Quebec	11 700	73·0	12 400 (10 600 to 14 100)	77·0 (66·0 to 87·9)	1·05 (0·90 to 1·20)
		Saskatchewan	955	43·6	1660 (1440 to 1910)	75·8 (66·0 to 87·2)	1·74 (1·51 to 2·00)
		Yukon	15	19·7	32 (26 to 37)	42·4 (34·5 to 49·1)	2·15 (1·75 to 2·49)
	Greenland		1	1·0	22 (14 to 29)	21·5 (13·5 to 27·8)	22·25 (13·99 to 28·78)
	USA		824 000	130·6	1 130 000 (1 080 000 to 1 180 000)	179·3 (170·7 to 187·5)	1·37 (1·31 to 1·44)
		Alabama	16 500	176·6	27 400 (26 000 to 28 600)	293·5 (278·5 to 307·2)	1·66 (1·58 to 1·74)
		Alaska	975	71·5	1640 (1380 to 1950)	120·4 (101·5 to 143·1)	1·68 (1·42 to 2·00)
		Arizona	24 200	171·9	31 900 (30 700 to 33 300)	226·2 (218·1 to 235·9)	1·32 (1·27 to 1·37)
		Arkansas	9150	162·3	14 400 (13 200 to 15 400)	255·5 (234·7 to 272·6)	1·57 (1·45 to 1·68)
		California	76 500	98·4	112 000 (108 000 to 116 000)	144·3 (138·6 to 148·7)	1·47 (1·41 to 1·51)
		Colorado	10 300	94·5	12 500 (11 800 to 13 400)	115·2 (108·3 to 123·2)	1·22 (1·15 to 1·30)
		Connecticut	9160	136·0	10 100 (9020 to 11 100)	150·5 (134·0 to 164·5)	1·11 (0·98 to 1·21)
		Delaware	2400	131·3	3660 (3360 to 4020)	200·9 (184·2 to 220·6)	1·53 (1·40 to 1·68)
		Washington, DC	1210	93·5	−62 (−405 to 313)	−4·7 (−31·3 to 24·1)	−0·05 (−0·33 to 0·26)
		Florida	62 400	153·0	86 400 (84 000 to 88 700)	211·9 (206·1 to 217·4)	1·38 (1·35 to 1·42)
		Georgia	31 400	155·6	44 300 (43 100 to 45 700)	219·3 (213·1 to 226·1)	1·41 (1·37 to 1·45)
		Hawaii	1080	39·7	851 (442 to 1320)	31·2 (16·2 to 48·4)	0·79 (0·41 to 1·22)
		Idaho	4160	120·8	6200 (5760 to 6700)	179·8 (167·2 to 194·5)	1·49 (1·38 to 1·61)
		Illinois	30 300	120·3	43 400 (40 800 to 45 700)	172·4 (162·3 to 181·6)	1·43 (1·35 to 1·51)
		Indiana	19 000	149·7	21 500 (20 600 to 22 700)	169·4 (162·3 to 178·2)	1·13 (1·08 to 1·19)
		Iowa	7860	132·0	7940 (6870 to 8840)	133·4 (115·4 to 148·5)	1·01 (0·87 to 1·12)
		Kansas	7010	127·8	9580 (8610 to 10 400)	174·8 (157·0 to 189·9)	1·37 (1·23 to 1·49)
		Kentucky	12 100	143·6	20 400 (19 000 to 21 700)	241·5 (224·8 to 257·3)	1·68 (1·57 to 1·79)
		Louisiana	15 000	172·6	22 300 (20 800 to 23 600)	257·1 (240·0 to 271·3)	1·49 (1·39 to 1·57)
		Maine	1530	60·2	2600 (1760 to 3290)	102·4 (69·2 to 129·6)	1·70 (1·15 to 2·15)
		Maryland	11 800	101·5	19 400 (18 100 to 20 700)	167·6 (156·1 to 178·2)	1·65 (1·54 to 1·76)
		Massachusetts	20 300	146·4	15 100 (13 500 to 16 600)	109·1 (97·5 to 120·0)	0·75 (0·67 to 0·82)
		Michigan	29 000	154·7	33 600 (31 800 to 35 500)	179·4 (169·8 to 189·5)	1·16 (1·10 to 1·22)
		Minnesota	10 700	99·5	10 100 (8730 to 11 200)	94·1 (81·6 to 105·0)	0·95 (0·82 to 1·05)
		Mississippi	10 400	190·0	18 100 (16 900 to 19 100)	329·7 (308·0 to 347·0)	1·74 (1·62 to 1·83)
		Missouri	16 200	141·3	22 100 (21 200 to 23 100)	192·1 (184·4 to 201·4)	1·36 (1·30 to 1·43)
		Montana	2910	143·7	3560 (2810 to 4190)	176·1 (138·9 to 207·3)	1·23 (0·97 to 1·44)
		Nebraska	3340	88·9	4710 (3870 to 5400)	125·3 (103·1 to 143·7)	1·41 (1·16 to 1·62)
		Nevada	8420	135·0	10 300 (9550 to 11 200)	165·2 (153·1 to 179·4)	1·22 (1·13 to 1·33)
		New Hampshire	1960	75·5	3470 (3100 to 3940)	133·7 (119·6 to 151·7)	1·77 (1·58 to 2·01)
		New Jersey	29 000	166·4	34 800 (32 900 to 36 500)	199·5 (188·6 to 209·4)	1·20 (1·13 to 1·26)
		New Mexico	5860	148·7	9450 (8940 to 10 000)	240·1 (227·1 to 254·0)	1·61 (1·53 to 1·71)
		New York	59 400	155·8	78 100 (74 500 to 81 100)	204·8 (195·2 to 212·5)	1·31 (1·25 to 1·36)
		North Carolina	19 400	98·7	33 700 (32 300 to 35 500)	171·3 (163·9 to 180·4)	1·74 (1·66 to 1·83)
		North Dakota	2060	140·9	165 (−192 to 607)	11·3 (−13·1 to 41·6)	0·08 (−0·09 to 0·29)
		Ohio	31 600	142·2	47 100 (45 100 to 49 000)	211·6 (202·9 to 220·3)	1·49 (1·43 to 1·55)
		Oklahoma	12 400	167·5	18 500 (17 200 to 19 500)	249·3 (232·8 to 263·9)	1·49 (1·39 to 1·58)
		Oregon	5970	74·3	10 100 (8920 to 11 100)	125·5 (111·1 to 138·4)	1·69 (1·49 to 1·86)
		Pennsylvania	36 700	150·8	42 000 (39 900 to 44 200)	172·5 (164·1 to 181·6)	1·14 (1·09 to 1·20)
		Rhode Island	3070	148·0	2350 (1720 to 2870)	113·3 (83·1 to 138·6)	0·77 (0·56 to 0·94)
		South Carolina	14 600	152·5	23 800 (23 000 to 24 700)	248·1 (239·5 to 257·7)	1·63 (1·57 to 1·69)
		South Dakota	2490	150·3	2590 (1990 to 3110)	156·4 (120·2 to 188·2)	1·04 (0·80 to 1·25)
		Tennessee	20 800	160·5	26 800 (25 900 to 28 100)	206·7 (199·2 to 216·5)	1·29 (1·24 to 1·35)
		Texas	75 700	133·8	114 000 (111 000 to 117 000)	200·8 (195·2 to 205·9)	1·50 (1·46 to 1·54)
		Utah	3810	61·8	4810 (4260 to 5470)	78·1 (69·2 to 88·7)	1·26 (1·12 to 1·44)
		Vermont	471	39·2	2080 (1610 to 2460)	173·1 (133·8 to 204·7)	4·41 (3·41 to 5·22)
		Virginia	15 600	96·5	24 500 (23 600 to 25 600)	151·8 (146·1 to 158·7)	1·57 (1·51 to 1·64)
		Washington	9900	68·8	13 100 (10 400 to 15 900)	90·8 (72·3 to 110·3)	1·32 (1·05 to 1·60)
		West Virginia	5340	160·5	9260 (8340 to 10 000)	278·4 (250·8 to 301·7)	1·73 (1·56 to 1·88)
		Wisconsin	11 200	97·0	12 700 (11 100 to 14 100)	110·2 (96·3 to 122·3)	1·14 (0·99 to 1·26)
		Wyoming	1530	142·3	2120 (1670 to 2530)	197·9 (155·6 to 235·6)	1·39 (1·09 to 1·66)
	**Southern Latin America**		**162 000**	**129·0**	**189 000 (172 000 to 214 000)**	**150·2 (136·6 to 169·7)**	**1·16 (1·06 to 1·32)**
	Argentina		117 000	137·5	142 000 (125 000 to 165 000)	166·8 (147·1 to 194·1)	1·21 (1·07 to 1·41)
	Chile		39 100	113·7	37 200 (35 200 to 39 300)	108·2 (102·3 to 114·3)	0·95 (0·90 to 1·00)
	Uruguay		6170	97·6	9820 (8670 to 11 500)	155·4 (137·2 to 181·5)	1·59 (1·41 to 1·86)
	**Western Europe**		**796 000**	**94·7**	**1 180 000 (1 120 000 to 1 230 000)**	**140·0 (133·5 to 146·3)**	**1·48 (1·41 to 1·54)**
	Andorra		140	87·6	328 (286 to 376)	205·5 (178·9 to 235·1)	2·35 (2·04 to 2·68)
	Austria		13 700	80·7	18 300 (16 400 to 20 200)	107·5 (96·1 to 118·5)	1·33 (1·19 to 1·47)
	Belgium		28 300	126·5	32 800 (30 400 to 35 000)	146·6 (135·8 to 156·3)	1·16 (1·07 to 1·24)
	Cyprus		646	25·7	809 (509 to 1110)	32·2 (20·2 to 43·9)	1·25 (0·79 to 1·71)
	Denmark		3270	29·6	10 400 (8900 to 11 700)	94·1 (80·5 to 106·3)	3·18 (2·72 to 3·60)
	Finland		1740	16·1	8780 (7190 to 10 200)	80·8 (66·2 to 94·0)	5·03 (4·12 to 5·86)
	France		122 000	97·4	155 000 (151 000 to 160 000)	124·2 (120·5 to 127·7)	1·28 (1·24 to 1·31)
	Germany		112 000	66·4	203 000 (193 000 to 210 000)	120·5 (115·1 to 125·1)	1·82 (1·73 to 1·88)
		Baden-Württemberg	13 000	58·6	25 300 (23 400 to 26 900)	114·6 (105·9 to 121·5)	1·96 (1·81 to 2·07)
		Bavaria	19 600	72·2	34 600 (31 700 to 36 500)	127·2 (116·6 to 134·3)	1·76 (1·62 to 1·86)
		Berlin	4010	56·0	8380 (8000 to 8920)	117·2 (111·8 to 124·6)	2·09 (2·00 to 2·22)
		Brandenburg	4680	92·6	7170 (6660 to 7600)	141·9 (131·8 to 150·4)	1·53 (1·42 to 1·63)
		Bremen	599	43·9	1420 (1290 to 1530)	103·7 (94·6 to 112·4)	2·36 (2·16 to 2·56)
		Hamburg	1990	54·7	3880 (3570 to 4150)	106·9 (98·3 to 114·1)	1·95 (1·80 to 2·09)
		Hesse	8550	68·1	15 200 (14 400 to 15 900)	121·2 (114·4 to 126·6)	1·78 (1·68 to 1·86)
		Lower Saxony	6810	42·3	14 300 (12 300 to 15 600)	88·5 (76·3 to 97·0)	2·09 (1·80 to 2·29)
		Mecklenburg-Western Pomerania	1470	44·9	3410 (2920 to 3810)	104·0 (89·0 to 116·3)	2·32 (1·98 to 2·59)
		North Rhine-Westphalia	20 300	55·8	42 300 (40 200 to 44 000)	116·5 (110·7 to 121·1)	2·09 (1·98 to 2·17)
		Rhineland-Palatinate	4620	56·1	8530 (7430 to 9220)	103·5 (90·2 to 111·9)	1·85 (1·61 to 2·00)
		Saarland	1240	61·5	2290 (2090 to 2440)	113·0 (103·2 to 120·3)	1·84 (1·68 to 1·96)
		Saxony	12 800	154·4	16 800 (15 500 to 18 100)	202·0 (187·0 to 218·0)	1·31 (1·21 to 1·41)
		Saxony-Anhalt	4250	93·0	6400 (5950 to 6830)	140·3 (130·3 to 149·6)	1·51 (1·40 to 1·61)
		Schleswig-Holstein	1880	32·4	4520 (3820 to 5090)	77·7 (65·8 to 87·6)	2·40 (2·03 to 2·70)
		Thuringia	5840	132·4	8140 (7520 to 8770)	184·6 (170·4 to 198·9)	1·39 (1·29 to 1·50)
	Greece		20 800	104·1	25 400 (23 400 to 27 400)	127·1 (117·0 to 137·2)	1·22 (1·12 to 1·32)
	Iceland		37	5·6	−314 (−703 to 11)	−47·8 (−107·1 to 1·6)	−8·49 (−19·01 to 0·29)
	Ireland		5910	63·5	1170 (84 to 2330)	12·5 (0·9 to 25·0)	0·20 (0·01 to 0·39)
	Israel		8240	45·3	9280 (7860 to 10 600)	51·0 (43·2 to 58·2)	1·13 (0·95 to 1·28)
	Italy		137 000	120·6	259 000 (242 000 to 276 000)	227·4 (212·0 to 242·5)	1·89 (1·76 to 2·01)
		Abruzzo	2640	106·4	4570 (4040 to 5130)	184·2 (162·7 to 206·9)	1·73 (1·53 to 1·94)
		Basilicata	634	60·2	1700 (1360 to 2040)	161·9 (128·9 to 193·9)	2·69 (2·14 to 3·22)
		Calabria	1610	44·3	6920 (6180 to 7670)	190·0 (169·8 to 210·5)	4·29 (3·83 to 4·75)
		Campania	8470	77·8	18∼600 (17 300 to 19 900)	170·6 (159·2 to 182·6)	2·19 (2·04 to 2·35)
		Emilia-Romagna	14 200	164·8	19 900 (18 600 to 21 100)	230·2 (215·7 to 244·7)	1·40 (1·31 to 1·48)
		Friuli-Venezia Giulia	4210	184·0	5060 (4610 to 5610)	221·1 (201·3 to 245·0)	1·20 (1·09 to 1·33)
		Lazio	9270	85·5	18 400 (17 100 to 19 600)	169·4 (158·0 to 180·8)	1·98 (1·85 to 2·11)
		Liguria	4580	158·0	9840 (9410 to 10 300)	339·2 (324·4 to 356·0)	2·15 (2·05 to 2·25)
		Lombardy	35 100	185·9	59 500 (56 600 to 62 000)	315·1 (300·1 to 328·5)	1·70 (1·61 to 1·77)
		Marche	3240	109·6	7400 (6790 to 8040)	250·0 (229·5 to 271·5)	2·28 (2·09 to 2·48)
		Molise	512	90·0	1900 (1780 to 2070)	334·5 (312·1 to 363·1)	3·72 (3·47 to 4·03)
		Piedmont	12 000	146·0	27 100 (25 600 to 28 700)	328·9 (310·6 to 347·5)	2·25 (2·13 to 2·38)
		Provincia autonoma di Bolzano	1300	130·5	1670 (1430 to 2040)	167·3 (143·0 to 203·7)	1·28 (1·10 to 1·56)
		Provincia autonoma di Trento	1420	136·4	1790 (1580 to 2030)	172·2 (151·4 to 194·4)	1·26 (1·11 to 1·42)
		Puglia	6990	91·7	17 700 (16 300 to 19 000)	232·1 (214·0 to 249·6)	2·53 (2·33 to 2·72)
		Sardinia	1730	56·3	4670 (4090 to 5320)	152·3 (133·2 to 173·3)	2·70 (2·36 to 3·08)
		Sicily	7500	79·3	19 400 (18 000 to 20 600)	204·5 (189·7 to 218·0)	2·58 (2·39 to 2·75)
		Tuscany	7590	107·5	12 700 (11 700 to 13 800)	179·6 (165·4 to 195·0)	1·67 (1·54 to 1·81)
		Umbria	1500	87·2	3180 (2960 to 3440)	184·5 (171·7 to 199·7)	2·11 (1·97 to 2·29)
		Aosta Valley	488	204·2	713 (572 to 840)	298·3 (239·5 to 351·7)	1·46 (1·17 to 1·72)
		Veneto	12 400	132·5	16 600 (15 300 to 17 800)	177·5 (164·0 to 190·7)	1·34 (1·24 to 1·44)
	Luxembourg		915	76·0	1070 (685 to 1400)	89·2 (56·8 to 116·4)	1·17 (0·75 to 1·53)
	Malta		476	58·2	735 (528 to 925)	89·9 (64·6 to 113·0)	1·54 (1·11 to 1·94)
	Monaco		38	53·1	53 (40 to 62)	74·4 (55·5 to 86·3)	1·40 (1·04 to 1·63)
	Netherlands		21 400	65·8	45 500 (42 700 to 48 000)	140·0 (131·3 to 147·6)	2·13 (2·00 to 2·24)
	Norway		1300	12·8	742 (−4 to 1630)	7·2 (−0·0 to 15·9)	0·57 (−0·00 to 1·25)
	Portugal		19 000	94·8	40 400 (38 100 to 42 400)	202·2 (190·7 to 212·2)	2·13 (2·01 to 2·24)
	San Marino		100	160·3	118 (100 to 138)	189·6 (159·7 to 221·5)	1·18 (1·00 to 1·38)
	Spain		98 900	114·1	162 000 (157 000 to 166 000)	186·7 (181·3 to 191·5)	1·64 (1·59 to 1·68)
		Andalucia	11 800	75·2	24 000 (21 800 to 29 400)	153·0 (139·0 to 187·7)	2·03 (1·85 to 2·50)
		Aragon	4070	168·6	6050 (5270 to 6850)	250·3 (218·3 to 283·5)	1·48 (1·29 to 1·68)
		Asturias	2160	115·2	3430 (2810 to 3940)	182·9 (150·1 to 210·3)	1·59 (1·30 to 1·83)
		Balearic Islands	1070	50·2	2980 (2400 to 3640)	139·9 (112·8 to 170·7)	2·79 (2·25 to 3·40)
		Basque Country	4990	121·2	7410 (6370 to 8200)	179·8 (154·5 to 199·1)	1·48 (1·28 to 1·64)
		Canary Islands	1140	28·7	3700 (2960 to 4840)	93·1 (74·3 to 121·8)	3·24 (2·59 to 4·24)
		Cantabria	635	59·0	1430 (1200 to 1620)	132·4 (111·3 to 150·9)	2·24 (1·89 to 2·56)
		Castile and León	6420	147·3	9980 (9110 to 11 000)	228·8 (208·8 to 251·8)	1·55 (1·42 to 1·71)
		Castilla–La Mancha	6580	176·9	8710 (8040 to 9450)	234·0 (215·9 to 253·8)	1·32 (1·22 to 1·43)
		Catalonia	24 500	174·1	35 600 (33 200 to 40 600)	252·6 (235·9 to 288·8)	1·45 (1·35 to 1·66)
		Ceuta	131	84·4	255 (227 to 282)	164·3 (146·1 to 181·5)	1·95 (1·73 to 2·15)
		Extremadura	2010	103·4	3500 (3070 to 4490)	179·9 (157·8 to 230·5)	1·74 (1·53 to 2·23)
		Galicia	2760	56·4	5790 (5060 to 6440)	118·2 (103·3 to 131·5)	2·10 (1·83 to 2·33)
		La Rioja	844	144·0	1220 (1050 to 1460)	208·9 (178·4 to 249·7)	1·45 (1·24 to 1·73)
		Madrid	18 500	148·8	27 000 (24 500 to 29 400)	217·8 (197·0 to 236·6)	1·46 (1·32 to 1·59)
		Melilla	121	76·4	304 (248 to 386)	192·1 (156·5 to 243·6)	2·51 (2·05 to 3·19)
		Murcia	1830	66·8	3550 (3000 to 3970)	129·7 (109·5 to 145·2)	1·94 (1·64 to 2·18)
		Navarre	1290	108·0	2250 (1980 to 2440)	188·3 (165·7 to 204·0)	1·74 (1·53 to 1·89)
		Valencian Community	8120	87·9	14 800 (13 300 to 16 000)	160·2 (144·6 to 173·5)	1·82 (1·64 to 1·97)
	Sweden		15 300	77·2	18 100 (16 900 to 19 500)	91·2 (85·2 to 98·1)	1·18 (1·10 to 1·27)
	Switzerland		12 000	72·0	15 500 (14 000 to 17 000)	93·1 (83·9 to 101·8)	1·29 (1·16 to 1·42)
	UK		173 000	130·1	169 000 (163 000 to 174 000)	126·8 (122·3 to 130·9)	0·97 (0·94 to 1·01)
		England	147 000	131·1	142 000 (137 000 to 145 000)	125·8 (122·1 to 128·7)	0·96 (0·93 to 0·98)
		Northern Ireland	4010	107·3	4930 (3800 to 6170)	131·8 (101·6 to 165·0)	1·23 (0·95 to 1·54)
		Scotland	12 500	117·3	13 900 (12 300 to 15 400)	130·6 (115·7 to 145·1)	1·11 (0·99 to 1·24)
		Wales	9180	149·5	8320 (7480 to 9050)	135·5 (121·9 to 147·5)	0·91 (0·82 to 0·99)
**Latin America and the Caribbean**			**1 520 000**	**134·7**	**2 860 000 (2 680 000 to 3 070 000)**	**254·0 (237·6 to 272·2)**	**1·89 (1·76 to 2·02)**
	**Andean Latin America**		**257 000**	**211·2**	**622 000 (580 000 to 669 000)**	**511·9 (477·8 to 550·5)**	**2·42 (2·26 to 2·61)**
	Bolivia		19 700	89·8	161 000 (130 000 to 193 000)	734·9 (594·1 to 879·2)	8·18 (6·61 to 9·79)
	Ecuador		34 100	101·5	112 000 (106 000 to 117 000)	333·4 (315·1 to 348·0)	3·28 (3·10 to 3·43)
	Peru		203 000	307·2	349 000 (328 000 to 367 000)	528·6 (497·5 to 556·4)	1·72 (1·62 to 1·81)
	**Caribbean**		**26 800**	**31·5**	**103 000 (68 100 to 140 000)**	**120·7 (80·1 to 164·3)**	**3·83 (2·54 to 5·22)**
	Antigua and Barbuda		119	71·8	43 (−89 to 249)	26·1 (−53·9 to 150·2)	0·36 (−0·75 to 2·09)
	The Bahamas		717	100·3	1060 (547 to 1440)	147·6 (76·4 to 200·9)	1·47 (0·76 to 2·00)
	Barbados		260	47·3	553 (319 to 789)	100·7 (58·1 to 143·6)	2·13 (1·23 to 3·03)
	Belize		602	77·6	982 (540 to 1330)	126·5 (69·6 to 172·0)	1·63 (0·90 to 2·22)
	Bermuda		110	94·1	145 (84 to 213)	123·9 (72·1 to 182·3)	1·32 (0·77 to 1·94)
	Cuba		8320	39·6	26 600 (15 000 to 46 800)	126·8 (71·5 to 223·0)	3·20 (1·80 to 5·63)
	Dominica		47	38·2	149 (83 to 224)	120·6 (67·3 to 182·3)	3·16 (1·76 to 4·78)
	Dominican Republic		4250	20·6	20 800 (10 400 to 29 100)	100·7 (50·3 to 141·0)	4·89 (2·45 to 6·85)
	Grenada		200	106·3	255 (132 to 392)	135·7 (70·2 to 208·3)	1·28 (0·66 to 1·96)
	Guyana		1050	76·7	2690 (1390 to 3930)	196·0 (101·3 to 286·7)	2·55 (1·32 to 3·74)
	Haiti		766	3·3	27 900 (14 100 to 42 200)	118·3 (59·7 to 178·9)	36·38 (18·36 to 55·04)
	Jamaica		2470	47·5	6460 (3680 to 8720)	124·1 (70·6 to 167·5)	2·61 (1·49 to 3·53)
	Puerto Rico		3300	54·0	6000 (5040 to 6950)	98·0 (82·3 to 113·5)	1·82 (1·53 to 2·10)
	Saint Kitts and Nevis		28	26·2	30 (−50 to 74)	28·6 (−46·9 to 69·1)	1·09 (−1·79 to 2·63)
	Saint Lucia		295	89·8	501 (286 to 710)	152·3 (87·1 to 216·0)	1·70 (0·97 to 2·41)
	Saint Vincent and the Grenadines		81	38·2	234 (121 to 361)	110·6 (57·3 to 170·5)	2·89 (1·50 to 4·46)
	Suriname		1190	109·0	2590 (1370 to 3790)	237·2 (125·7 to 346·9)	2·18 (1·15 to 3·18)
	Trinidad and Tobago		2870	111·1	5130 (2640 to 7830)	198·8 (102·2 to 303·5)	1·79 (0·92 to 2·73)
	Virgin Islands		89	47·4	526 (301 to 837)	280·3 (160·1 to 445·5)	5·91 (3·38 to 9·40)
	**Central Latin America**		**599 000**	**124·1**	**1 320 000 (1 240 000 to 1 430 000)**	**274·4 (256·6 to 296·4)**	**2·21 (2·07 to 2·39)**
	Colombia		130 000	145·9	179 000 (171 000 to 187 000)	201·2 (191·5 to 209·4)	1·38 (1·31 to 1·44)
	Costa Rica		7350	82·8	6220 (5450 to 7310)	70·0 (61·4 to 82·4)	0·85 (0·74 to 0·99)
	El Salvador		3820	32·2	26 900 (21 000 to 32 800)	226·8 (177·1 to 276·4)	7·04 (5·49 to 8·57)
	Guatemala		16 400	56·4	51 100 (43 200 to 59 200)	175·7 (148·6 to 203·2)	3·11 (2·63 to 3·60)
	Honduras		10 400	56·0	55 400 (46 900 to 67 100)	297·1 (251·7 to 360·0)	5·31 (4·50 to 6·43)
	Mexico		418 000	170·2	798 000 (741 000 to 867 000)	325·1 (301·6 to 353·3)	1·91 (1·77 to 2·08)
		Aguascalientes	4470	161·5	7070 (6420 to 7650)	255·8 (232·2 to 276·6)	1·58 (1·44 to 1·71)
		Baja California	14 100	193·1	24 700 (21 600 to 27 400)	337·0 (295·4 to 374·0)	1·75 (1·53 to 1·94)
		Baja California Sur	3060	198·3	3740 (3330 to 4220)	242·2 (216·3 to 273·4)	1·22 (1·09 to 1·38)
		Campeche	3250	181·9	4940 (4430 to 5540)	276·0 (247·6 to 309·4)	1·52 (1·36 to 1·70)
		Chiapas	4730	43·1	23 100 (20 500 to 25 800)	210·9 (187·0 to 235·0)	4·90 (4·34 to 5·46)
		Chihuahua	11 800	162·8	26 600 (23 300 to 29 500)	367·7 (321·9 to 407·8)	2·26 (1·98 to 2·51)
		Coahuila	11 200	182·7	21 600 (19 400 to 23 600)	352·7 (317·2 to 386·3)	1·93 (1·74 to 2·11)
		Colima	2480	175·4	2540 (2220 to 2870)	179·3 (157·1 to 202·4)	1·02 (0·90 to 1·15)
		Durango	4960	139·6	8610 (7490 to 9630)	242·3 (210·7 to 271·1)	1·74 (1·51 to 1·94)
		Guanajuato	16 600	138·7	32 900 (30 900 to 35 200)	274·6 (258·1 to 294·1)	1·98 (1·86 to 2·12)
		Guerrero	9610	139·5	15 900 (14 500 to 17 500)	230·4 (210·7 to 254·7)	1·65 (1·51 to 1·83)
		Hidalgo	10 000	164·4	16 200 (14 900 to 17 300)	265·5 (244·9 to 284·1)	1·62 (1·49 to 1·73)
		Jalisco	23 200	142·9	34 200 (31 000 to 38 000)	210·5 (190·6 to 233·8)	1·47 (1·33 to 1·64)
		México	73 900	220·6	145 000 (134 000 to 156 000)	433·6 (398·9 to 466·3)	1·97 (1·81 to 2·11)
		Mexico City	61 300	339·9	104 000 (95 400 to 112 000)	576·9 (528·6 to 620·9)	1·70 (1·56 to 1·83)
		Michoacán de Ocampo	10 600	115·6	20 200 (18 600 to 22 000)	219·0 (202·4 to 238·4)	1·90 (1·75 to 2·06)
		Morelos	7070	184·9	11 200 (9930 to 12300)	291·6 (259·5 to 321·9)	1·58 (1·40 to 1·74)
		Nayarit	3150	131·4	4170 (3780 to 4610)	173·8 (157·4 to 192·0)	1·32 (1·20 to 1·46)
		Nuevo León	19 900	177·1	32 800 (30 700 to 35 500)	292·3 (273·6 to 315·8)	1·65 (1·55 to 1·78)
		Oaxaca	8290	102·4	17 600 (16 200 to 19 700)	217·8 (199·6 to 243·7)	2·13 (1·95 to 2·38)
		Puebla	20 600	160·9	51 100 (48 200 to 55 200)	399·7 (376·7 to 431·5)	2·48 (2·34 to 2·68)
		Querétaro	7000	151·7	10 300 (9380 to 11 300)	224·0 (203·2 to 244·0)	1·48 (1·34 to 1·61)
		Quintana Roo	5240	143·5	8840 (7720 to 10 300)	241·7 (211·3 to 281·1)	1·68 (1·47 to 1·96)
		San Luis Potosí	8190	150·0	16 900 (15 700 to 18 000)	308·9 (287·8 to 330·1)	2·06 (1·92 to 2·20)
		Sinaloa	10 200	173·9	17 900 (15 900 to 19 900)	303·9 (270·2 to 337·7)	1·75 (1·55 to 1·94)
		Sonora	11 300	198·3	19 300 (17 200 to 21 500)	340·6 (303·3 to 377·9)	1·72 (1·53 to 1·91)
		Tabasco	7330	158·0	16 000 (14 400 to 18 100)	344·7 (309·8 to 390·8)	2·18 (1·96 to 2·47)
		Tamaulipas	9280	136·3	21 800 (19 700 to 23 700)	320·1 (288·7 to 347·2)	2·35 (2·12 to 2·55)
		Tlaxcala	3640	140·1	13 400 (12 400 to 14 700)	517·2 (476·3 to 567·0)	3·69 (3·40 to 4·05)
		Veracruz de Ignacio de la Llave	20 800	133·2	44 300 (41 400 to 47 800)	284·4 (266·0 to 306·5)	2·14 (2·00 to 2·30)
		Yucatán	6070	135·1	11 300 (10 200 to 12 500)	252·1 (227·6 to 279·1)	1·87 (1·68 to 2·07)
		Zacatecas	4610	146·8	9780 (9040 to 10 400)	311·4 (288·1 to 332·8)	2·12 (1·96 to 2·27)
	Nicaragua		223	1·8	33 400 (31 300 to 36 100)	274·4 (256·6 to 296·4)	149·99 (140·28 to 162·04)
	Panama		7430	96·2	10 200 (8840 to 11 300)	131·5 (114·4 to 145·8)	1·37 (1·19 to 1·52)
	Venezuela		5430	9·1	164 000 (153 000 to 177 000)	274·4 (256·6 to 296·4)	30·17 (28·21 to 32·59)
	**Tropical Latin America**		**636 000**	**145·1**	**815 000 (751 000 to 870 000)**	**186·0 (171·5 to 198·5)**	**1·28 (1·18 to 1·37)**
	Brazil		619 000	146·0	792 000 (730 000 to 847 000)	186·9 (172·2 to 199·8)	1·28 (1·18 to 1·37)
		Acre	1850	101·3	2470 (2040 to 2860)	135·0 (111·4 to 156·3)	1·33 (1·10 to 1·54)
		Alagoas	6380	85·8	7270 (6050 to 8390)	97·8 (81·3 to 112·8)	1·14 (0·95 to 1·31)
		Amapá	2020	125·8	3370 (2860 to 3820)	209·5 (178·1 to 237·7)	1·67 (1·42 to 1·89)
		Amazonas	13 800	167·2	20 400 (18 500 to 23 000)	246·1 (223·9 to 278·1)	1·47 (1·34 to 1·66)
		Bahia	27 500	87·4	45 700 (41 900 to 48 500)	145·1 (132·9 to 154·2)	1·66 (1·52 to 1·77)
		Ceará	24 800	124·3	50 900 (45 900 to 57 500)	255·1 (230·2 to 288·0)	2·05 (1·85 to 2·32)
		Federal District	11 100	183·4	10 900 (9700 to 12 000)	180·8 (160·2 to 198·5)	0·99 (0·87 to 1·08)
		Espírito Santo	13 300	171·6	17 400 (15 100 to 20 000)	224·3 (194·1 to 258·1)	1·31 (1·13 to 1·50)
		Goiás	24 700	186·2	29 700 (27 800 to 31 300)	223·9 (209·5 to 236·4)	1·20 (1·13 to 1·27)
		Maranhão	10 400	64·3	32 900 (29 600 to 45 800)	204·2 (183·3 to 284·1)	3·17 (2·85 to 4·41)
		Mato Grosso	13 800	200·2	16 100 (14 800 to 17 100)	233·7 (214·7 to 248·0)	1·17 (1·07 to 1·24)
		Mato Grosso do Sul	9730	176·1	9800 (8720 to 10 700)	177·4 (157·9 to 194·0)	1·01 (0·90 to 1·10)
		Minas Gerais	56 700	133·7	74 200 (69 000 to 78 800)	175·1 (162·9 to 185·8)	1·31 (1·22 to 1·39)
		Pará	17 100	96·7	25 500 (23 000 to 27 400)	144·0 (130·3 to 155·0)	1·49 (1·35 to 1·60)
		Paraíba	9600	111·8	9230 (8420 to 10 200)	107·6 (98·1 to 118·9)	0·96 (0·88 to 1·06)
		Paraná	40 900	184·6	43 000 (40 200 to 45 600)	194·3 (181·3 to 205·7)	1·05 (0·98 to 1·11)
		Pernambuco	20 400	103·4	32 200 (29 600 to 34 400)	163·0 (149·7 to 174·1)	1·58 (1·45 to 1·68)
		Piauí	7280	103·3	10 100 (8910 to 11 100)	143·7 (126·6 to 158·2)	1·39 (1·23 to 1·53)
		Rio de Janeiro	69 500	197·9	80 200 (74 300 to 85 300)	228·4 (211·8 to 242·9)	1·15 (1·07 to 1·23)
		Rio Grande do Norte	7570	103·0	8920 (7870 to 9810)	121·4 (107·1 to 133·5)	1·18 (1·04 to 1·30)
		Rio Grande do Sul	36 400	170·3	36 400 (33 900 to 39 200)	170·2 (158·4 to 183·2)	1·00 (0·93 to 1·08)
		Rondônia	6730	190·6	9510 (8650 to 10 200)	269·0 (244·7 to 289·4)	1·41 (1·28 to 1·52)
		Roraima	2080	180·7	2110 (1910 to 2370)	183·9 (166·2 to 206·0)	1·02 (0·92 to 1·14)
		Santa Catarina	20 200	146·8	20 200 (18 600 to 21 600)	147·0 (135·4 to 157·3)	1·00 (0·92 to 1·07)
		São Paulo	155 000	172·8	182 000 (169 000 to 193 000)	202·9 (188·0 to 215·3)	1·17 (1·09 to 1·25)
		Sergipe	6060	126·3	5900 (5020 to 6690)	123·1 (104·7 to 139·6)	0·97 (0·83 to 1·11)
		Tocantins	3940	121·6	5480 (4880 to 6040)	169·2 (150·7 to 186·6)	1·39 (1·24 to 1·54)
	Paraguay		16 600	117·7	22 500 (21 600 to 23 300)	159·2 (153·1 to 165·3)	1·35 (1·30 to 1·40)
**North Africa and the Middle East**			**374 000**	**31·2**	**1 730 000 (1 570 000 to 1 990 000)**	**144·7 (131·0 to 165·9)**	**4·64 (4·20 to 5·32)**
	**North Africa and the Middle East**		**374 000**	**31·2**	**1 730 000 (1 570 000 to 1 990 000)**	**144·7 (131·0 to 165·9)**	**4·64 (4·20 to 5·32)**
	Afghanistan		7360	8·1	192 000 (142 000 to 242 000)	211·9 (157·4 to 267·1)	26·06 (19·36 to 32·86)
	Algeria		6280	7·5	54 400 (41 600 to 65 500)	65·1 (49·8 to 78·4)	8·67 (6·63 to 10·43)
	Bahrain		1390	48·0	3920 (3060 to 4780)	134·7 (105·4 to 164·4)	2·81 (2·20 to 3·43)
	Egypt		21 800	10·9	265 000 (248 000 to 278 000)	133·3 (124·9 to 140·0)	12·19 (11·42 to 12·80)
	Iran		132 000	77·1	274 000 (231 000 to 335 000)	160·5 (135·2 to 196·4)	2·08 (1·75 to 2·55)
	Iraq		24 200	36·9	183 000 (142 000 to 237 000)	280·1 (216·7 to 362·5)	7·59 (5·87 to 9·82)
	Jordan		12 700	53·5	27 000 (21 300 to 33 200)	114·4 (90·2 to 140·5)	2·14 (1·69 to 2·63)
	Kuwait		2470	28·5	3560 (2670 to 4300)	41·1 (30·9 to 49·6)	1·44 (1·08 to 1·74)
	Lebanon		9120	105·0	36 100 (30 200 to 44 800)	416·2 (347·4 to 515·5)	3·96 (3·31 to 4·91)
	Libya		5710	45·4	36 700 (29 200 to 45 100)	292·1 (232·2 to 358·8)	6·43 (5·11 to 7·90)
	Morocco		14 800	21·6	157 000 (126 000 to 203 000)	228·3 (182·9 to 294·9)	10·56 (8·46 to 13·64)
	Oman		4120	47·5	12 300 (11 500 to 12 900)	141·5 (133·0 to 148·8)	2·98 (2·80 to 3·13)
	Palestine		4660	48·9	12 600 (10 600 to 15 300)	132·2 (111·3 to 160·5)	2·70 (2·28 to 3·28)
	Qatar		618	10·7	1560 (1170 to 1800)	27·0 (20·3 to 31·3)	2·52 (1·89 to 2·92)
	Saudi Arabia		8880	12·5	32 900 (21 100 to 41 600)	46·4 (29·9 to 58·8)	3·70 (2·38 to 4·69)
	Sudan		3330	4·3	83 600 (59 900 to 100 000	108·5 (77·8 to 130·4)	25·12 (18·00 to 30·17)
	Syria		2900	6·6	27 100 (17 200 to 35 800)	61·7 (39·1 to 81·4)	9·36 (5·94 to 12·36)
	Tunisia		25 600	114·4	72 500 (59 300 to 84 100)	324·2 (265·4 to 376·4)	2·83 (2·32 to 3·29)
	Turkey		82 400	52·9	185 000 (160 000 to 217 000)	118·6 (103·1 to 139·2)	2·24 (1·95 to 2·63)
	United Arab Emirates		2160	21·1	9340 (6500 to 11 800)	91·3 (63·5 to 115·6)	4·32 (3·01 to 5·47)
	Yemen		1980	3·3	65 600 (37 800 to 97 600)	108·0 (62·2 to 160·9)	33·04 (19·03 to 49·21)
**South Asia**			**558 000**	**16·0**	**5 270 000 (4 710 000 to 5 700 000)**	**151·7 (135·5 to 163·9)**	**9·46 (8·45 to 10·22)**
	**South Asia**		**558 000**	**16·0**	**5 270 000 (4 710 000 to 5 700 000)**	**151·7 (135·5 to 163·9)**	**9·46 (8·45 to 10·22)**
	Bangladesh		28 100	9·2	413 000 (347 000 to 504 000)	134·7 (113·2 to 164·3)	14·72 (12·36 to 17·94)
	Bhutan		3	0·2	108 (45 to 160)	7·4 (3·1 to 10·9)	36·06 (15·14 to 53·25)
	India		489 000	18·3	4 070 000 (3 710 000 to 4 360 000)	152·5 (138·6 to 163·3)	8·33 (7·58 to 8·92)
		Andhra Pradesh	14 500	14·1	194 000 (170 000 to 211 000)	189·2 (165·6 to 206·3)	13·38 (11·71 to 14·59)
		Arunachal Pradesh	282	8·9	1790 (968 to 2400)	56·4 (30·5 to 75·6)	6·34 (3·43 to 8·51)
		Assam	6160	9·1	119 000 (96 200 to 137 000)	176·6 (142·1 to 202·8)	19·38 (15·60 to 22·26)
		Bihar	12 100	5·2	323 000 (257 000 to 388 000)	137·7 (109·7 to 165·5)	26·68 (21·27 to 32·07)
		Chhattisgarh	13 600	22·3	150 000 (132 000 to 167 000)	246·7 (215·9 to 274·2)	11·05 (9·67 to 12·28)
		Delhi	25 100	65·6	61 300 (41 600 to 77 100)	160·2 (108·8 to 201·5)	2·44 (1·66 to 3·07)
		Goa	3520	124·6	3400 (1550 to 4960)	120·2 (54·8 to 175·6)	0·96 (0·44 to 1·41)
		Gujarat	10 100	7·7	131 000 (103 000 to 149 000)	99·2 (78·2 to 113·3)	12·91 (10·18 to 14·76)
		Haryana	10 100	18·1	132 000 (116 000 to 146 000)	236·4 (207·3 to 262·2)	13·09 (11·48 to 14·52)
		Himachal Pradesh	3860	27·1	33 600 (26 500 to 39 200)	235·9 (186·1 to 275·3)	8·71 (6·87 to 10·17)
		Jammu & Kashmir and Ladakh	4750	17·7	41 600 (29 400 to 48 300)	155·4 (109·9 to 180·6)	8·76 (6·19 to 10·18)
		Jharkhand	5140	7·1	82 700 (61 300 to 99 600)	115·0 (85·2 to 138·4)	16·08 (11·92 to 19·37)
		Karnataka	38 300	28·7	284 000 (223 000 to 327 000)	212·9 (166·9 to 245·0)	7·41 (5·81 to 8·52)
		Kerala	58 500	84·3	114 000 (90 700 to 133 000)	165·1 (130·7 to 191·4)	1·96 (1·55 to 2·27)
		Madhya Pradesh	10 500	6·2	223 000 (188 000 to 253 000)	132·2 (111·2 to 149·7)	21·20 (17·83 to 24·00)
		Maharashtra	142 000	59·6	616 000 (484 000 to 736 000)	259·5 (203·7 to 309·8)	4·36 (3·42 to 5·20)
		Manipur	2000	29·8	17 700 (13 900 to 21 300)	263·8 (207·6 to 317·1)	8·85 (6·96 to 10·64)
		Meghalaya	1480	23·1	8910 (6620 to 11 100)	138·9 (103·1 to 173·1)	6·00 (4·46 to 7·48)
		Mizoram	543	22·9	2950 (2020 to 3700)	124·3 (85·1 to 156·2)	5·43 (3·71 to 6·82)
		Nagaland	711	22·7	5370 (3430 to 7380)	171·0 (109·2 to 235·1)	7·55 (4·82 to 10·38)
		Odisha	8510	9·5	130 000 (108 000 to 148 000)	145·4 (120·5 to 165·5)	15·24 (12·63 to 17·35)
		Punjab	16 600	28·0	135 000 (98 600 to 154 000)	227·1 (166·1 to 259·1)	8·10 (5·93 to 9·25)
		Rajasthan	8960	5·7	135 000 (97 600 to 162 000)	85·9 (62·1 to 102·8)	15·07 (10·88 to 18·03)
		Sikkim	409	34·5	992 (641 to 1260)	83·6 (54·1 to 106·5)	2·42 (1·57 to 3·09)
		Tamil Nadu	36 800	24·0	260 000 (199 000 to 301 000)	169·8 (130·3 to 196·7)	7·06 (5·42 to 8·18)
		Telangana	4030	5·2	55 400 (43 300 to 65 400)	71·9 (56·2 to 84·9)	13·75 (10·74 to 16·24)
		Tripura	826	11·0	9260 (6780 to 10 500)	123·5 (90·5 to 140·5)	11·21 (8·21 to 12·75)
		Uttar Pradesh	22 900	4·8	517 000 (418 000 to 602 000)	108·2 (87·4 to 125·8)	22·58 (18·24 to 26·27)
		Uttarakhand	7420	32·5	65 000 (51 200 to 75 700)	284·6 (224·1 to 331·5)	8·77 (6·90 to 10·21)
		West Bengal	19 800	10·5	220 000 (132 000 to 254 000)	116·7 (70·2 to 134·9)	11·14 (6·70 to 12·87)
	Nepal		11 600	19·1	123 000 (107 000 to 142 000)	202·5 (175·9 to 232·7)	10·61 (9·22 to 12·20)
	Pakistan		28 900	6·6	664 000 (498 000 to 847 000)	152·6 (114·3 to 194·7)	22·99 (17·22 to 29·32)
		Azad Jammu & Kashmir	746	9·8	12 900 (10 200 to 16 600)	170·3 (134·3 to 218·2)	17·35 (13·68 to 22·22)
		Balochistan	364	1·4	18 100 (10 500 to 26 500)	67·8 (39·5 to 99·3)	49·64 (28·94 to 72·74)
		Gilgit-Baltistan	186	5·3	5120 (3470 to 6820)	146·8 (99·5 to 195·3)	27·53 (18·67 to 36·65)
		Islamabad Capital Territory	967	22·8	3790 (2960 to 4600)	89·2 (69·7 to 108·2)	3·92 (3·07 to 4·75)
		Khyber Pakhtunkhwa	5930	8·0	186 000 (118 000 to 262 000)	252·8 (159·4 to 355·4)	31·44 (19·83 to 44·21)
		Punjab	13 100	5·9	313 000 (230 000 to 403 000)	141·1 (103·5 to 181·4)	23·98 (17·59 to 30·82)
		Sindh	7640	7·8	125 000 (96 700 to 159 000)	127·7 (99·2 to 162·9)	16·30 (12·66 to 20·78)
**Southeast Asia, east Asia, and Oceania**			**329 000**	**7·5**	**1 250 000 (1 060 000 to 1 500 000)**	**28·6 (24·2 to 34·3)**	**3·79 (3·22 to 4·55)**
	**East Asia**		**5760**	**0·2**	**15 500 (4320 to 27 900)**	**0·5 (0·1 to 0·9)**	**2·69 (0·75 to 4·85)**
	China		4820	0·2	17 900 (7540 to 30 100)	0·6 (0·3 to 1·0)	3·71 (1·56 to 6·25)
		Hubei	4510	4·0	10 600 (5710 to 15 500)	9·4 (5·1 to 13·7)	2·34 (1·27 to 3·42)
		All other provinces	311	0·0	7330 (1630 to 14 100)	0·3 (0·1 to 0·5)	23·57 (5·25 to 45·30)
	North Korea		86	0·2	318 (134 to 536)	0·6 (0·3 to 1·0)	3·71 (1·56 to 6·25)
	Taiwan (province of China)		850	1·8	−2720 (−4460 to −736)	−5·9 (−9·6 to −1·6)	−3·20 (−5·25 to −0·87)
	**Oceania**		**1570**	**7·3**	**17 500 (11 900 to 24 700)**	**81·3 (55·4 to 115·1)**	**11·11 (7·58 to 15·73)**
	Fiji		698	41·1	1480 (1030 to 1930)	87·1 (61·0 to 113·6)	2·12 (1·48 to 2·76)
	Guam		270	86·3	333 (292 to 382)	106·6 (93·5 to 122·3)	1·23 (1·08 to 1·42)
	Northern Mariana Islands		13	17·2	31 (18 to 44)	41·0 (23·7 to 57·4)	2·39 (1·38 to 3·35)
	Papua New Guinea		590	3·1	15 600 (10 400 to 22 200)	82·0 (54·9 to 116·8)	26·43 (17·69 to 37·68)
	Vanuatu		1	0·3	35 (10 to 72)	9·4 (2·7 to 19·2)	34·98 (10·18 to 71·57)
	**Southeast Asia**		**322 000**	**24·0**	**1 210 000 (1 030 000 to 1 460 000)**	**90·8 (77·0 to 109·0)**	**3·78 (3·20 to 4·54)**
	Cambodia		3010	9·0	17 500 (12 700 to 23 700)	52·5 (38·2 to 71·1)	5·80 (4·22 to 7·85)
	Indonesia		144 000	27·5	736 000 (594 000 to 955 000)	140·7 (113·6 to 182·5)	5·11 (4·12 to 6·63)
	Laos		372	2·8	4640 (3180 to 6190)	34·7 (23·8 to 46·3)	12·47 (8·56 to 16·65)
	Malaysia		31 500	53·3	48 100 (40 300 to 58 200)	81·4 (68·2 to 98·5)	1·53 (1·28 to 1·85)
	Maldives		262	27·3	296 (231 to 372)	30·8 (24·0 to 38·7)	1·13 (0·88 to 1·42)
	Mauritius		762	32·5	659 (331 to 987)	28·2 (14·1 to 42·2)	0·87 (0·43 to 1·30)
	Myanmar		19 300	19·2	10 1000 (71 800 to 140 000)	100·9 (71·7 to 139·7)	5·25 (3·73 to 7·26)
	Philippines		53 000	23·8	184 000 (175 000 to 197 000)	82·9 (78·8 to 88·7)	3·48 (3·31 to 3·72)
	Seychelles		134	69·1	116 (98 to 144)	60·0 (50·7 to 74·0)	0·87 (0·73 to 1·07)
	Sri Lanka		15 000	34·3	12 900 (7670 to 18 100)	29·5 (17·6 to 41·4)	0·86 (0·51 to 1·21)
	Thailand		21 700	15·3	35 200 (26 600 to 42 200)	24·9 (18·7 to 29·8)	1·62 (1·22 to 1·94)
	Timor-Leste		122	4·8	1300 (976 to 1610)	51·1 (38·4 to 63·3)	10·65 (8·00 to 13·19)
	Vietnam		32 400	16·6	72 300 (39 600 to 111 000)	37·0 (20·3 to 57·1)	2·23 (1·22 to 3·44)
**Sub-Saharan Africa**			**150 000**	**7·2**	**2 130 000 (1 730 000 to 2 820 000)**	**101·6 (82·4 to 134·5)**	**14·20 (11·51 to 18·79)**
	**Central sub-Saharan Africa**		**3910**	**1·5**	**195 000 (142 000 to 273 000)**	**76·8 (55·7 to 107·5)**	**50·00 (36·26 to 70·00)**
	Angola		1770	3·0	64 000 (46 000 to 88 900)	107·3 (77·2 to 149·0)	36·17 (26·01 to 50·20)
	Central African Republic		101	0·9	14 100 (8980 to 21 600)	118·4 (75·5 to 181·6)	139·24 (88·86 to 213·67)
	Congo (Brazzaville)		367	3·7	11 700 (9320 to 15 000)	117·2 (93·3 to 150·4)	31·91 (25·39 to 40·95)
	DR Congo		1200	0·7	98 700 (66 400 to 151 000)	59·2 (39·8 to 90·4)	81·94 (55·08 to 125·13)
	Equatorial Guinea		175	6·3	2770 (2090 to 3620)	99·9 (75·5 to 130·8)	15·82 (11·95 to 20·71)
	Gabon		288	8·6	3980 (3240 to 4770)	119·0 (96·7 to 142·5)	13·83 (11·25 to 16·57)
	**Eastern sub-Saharan Africa**		**28 800**	**3·6**	**1 010 000 (780 000 to 1 440 000)**	**127·1 (98·5 to 181·5)**	**35·00 (27·11 to 49·97)**
	Burundi		34	0·2	4310 (2730 to 6350)	19·8 (12·5 to 29·1)	126·85 (80·33 to 186·68)
	Comoros		157	12·4	1480 (1090 to 1850)	117·2 (86·1 to 146·6)	9·42 (6·92 to 11·79)
	Djibouti		189	8·1	3850 (2940 to 4840)	164·3 (125·7 to 206·8)	20·36 (15·57 to 25·63)
	Eritrea		76	0·5	7210 (5130 to 10 300)	51·0 (36·3 to 72·9)	94·90 (67·52 to 135·68)
	Ethiopia		6940	3·5	208 000 (150 000 to 313 000)	104·6 (75·4 to 157·5)	30·00 (21·63 to 45·19)
	Kenya		5380	5·7	171 000 (127 000 to 266 000)	181·2 (135·3 to 281·8)	31·75 (23·70 to 49·38)
	Madagascar		1030	2·0	65 100 (49 000 to 94 000)	125·4 (94·3 to 180·9)	63·44 (47·67 to 91·51)
	Malawi		2360	6·8	54 900 (45 400 to 68 900)	158·9 (131·4 to 199·4)	23·24 (19·22 to 29·16)
	Mozambique		2010	3·6	78 100 (54 100 to 109 000)	139·2 (96·4 to 193·8)	38·91 (26·95 to 54·17)
	Rwanda		1350	5·5	21 900 (17 500 to 27 500)	89·8 (71·4 to 112·6)	16·26 (12·93 to 20·38)
	Somalia		1330	2·6	89 000 (45 800 to 153 000)	172·1 (88·6 to 295·8)	66·78 (34·37 to 114·77)
	South Sudan		135	0·6	14 700 (8460 to 24 000)	66·1 (37·9 to 107·6)	109·08 (62·64 to 177·57)
	Tanzania		737	0·7	132 000 (102 000 to 188 000)	127·1 (98·5 to 181·5)	178·81 (138·54 to 255·32)
	Uganda		3290	4·2	73 500 (55 900 to 95 400)	93·5 (71·1 to 121·4)	22·31 (16·97 to 28·96)
	Zambia		3730	10·5	81 300 (59 100 to 115 000)	228·2 (165·9 to 322·8)	21·77 (15·83 to 30·79)
	**Southern sub-Saharan Africa**		**104 000**	**71·6**	**449 000 (418 000 to 483 000)**	**308·6 (287·3 to 331·6)**	**4·31 (4·01 to 4·63)**
	Botswana		2440	56·8	17 200 (14 200 to 20 700)	399·5 (329·1 to 482·1)	7·03 (5·79 to 8·48)
	Eswatini		1300	60·9	13 600 (10 500 to 16 600)	634·9 (491·8 to 775·4)	10·42 (8·07 to 12·73)
	Lesotho		665	21·0	17 800 (14 600 to 21 900)	562·9 (460·8 to 691·4)	26·82 (21·96 to 32·94)
	Namibia		3630	79·8	18 000 (15 500 to 20 400)	395·6 (340·2 to 448·8)	4·95 (4·26 to 5·62)
	South Africa		91 100	88·6	302 000 (287 000 to 332 000)	293·2 (279·0 to 322·8)	3·31 (3·15 to 3·64)
	Zimbabwe		5000	17·6	80 900 (64 000 to 101 000)	283·6 (224·7 to 352·7)	16·16 (12·80 to 20·09)
	**Western sub-Saharan Africa**		**13 200**	**1·5**	**480 000 (343 000 to 650 000)**	**53·0 (37·9 to 71·8)**	**36·37 (25·98 to 49·26)**
	Benin		161	0·7	8250 (5560 to 11 500)	33·6 (22·7 to 46·7)	51·23 (34·56 to 71·17)
	Burkina Faso		318	0·8	21 400 (14 200 to 31 800)	51·0 (33·8 to 75·9)	67·18 (44·53 to 99·91)
	Cape Verde		352	34·4	659 (505 to 790)	64·5 (49·4 to 77·3)	1·87 (1·44 to 2·24)
	Cameroon		1850	3·1	41 300 (30 800 to 53 600)	70·3 (52·4 to 91·2)	22·32 (16·63 to 28·94)
	Chad		181	0·6	15 200 (8570 to 23 500)	47·0 (26·5 to 72·7)	83·87 (47·34 to 129·85)
	Côte d'Ivoire		714	1·3	33 900 (24 600 to 47 100)	63·4 (45·9 to 88·0)	47·50 (34·43 to 66·00)
	The Gambia		343	7·8	6340 (5230 to 7840)	144·9 (119·6 to 179·2)	18·48 (15·25 to 22·85)
	Ghana		1300	2·1	35 900 (28 100 to 44 700)	58·3 (45·6 to 72·5)	27·75 (21·71 to 34·51)
	Guinea		391	1·6	26 700 (18 500 to 38 900)	111·0 (77·0 to 161·8)	68·23 (47·35 to 99·49)
	Guinea-Bissau		149	4·0	4350 (3310 to 5560)	116·4 (88·8 to 148·9)	29·17 (22·25 to 37·29)
	Liberia		287	3·3	7970 (6310 to 10 700)	90·6 (71·7 to 121·5)	27·77 (21·97 to 37·24)
	Mali		660	1·5	33 500 (22 100 to 47 500)	76·1 (50·3 to 107·8)	50·75 (33·55 to 71·95)
	Mauritania		866	10·9	7990 (5680 to 10 500)	100·5 (71·5 to 131·7)	9·22 (6·56 to 12·09)
	Niger		275	0·6	18 100 (10 300 to 27 700)	39·5 (22·4 to 60·3)	65·86 (37·38 to 100·67)
	Nigeria		3030	0·7	163 000 (93 500 to 238 000)	37·8 (21·7 to 55·3)	53·66 (30·85 to 78·54)
	São Tomé and Príncipe		57	14·8	235 (157 to 303)	61·1 (40·8 to 78·6)	4·13 (2·76 to 5·32)
	Senegal		1890	6·4	38 600 (31 900 to 50 000)	130·1 (107·6 to 168·5)	20·46 (16·92 to 26·51)
	Sierra Leone		123	0·8	7570 (4980 to 10 300)	47·3 (31·2 to 64·5)	61·54 (40·52 to 83·92)
	Togo		248	1·6	9030 (6920 to 12 000)	58·0 (44·5 to 77·2)	36·42 (27·91 to 48·49)

Data in parentheses are 95% uncertainty intervals.

**Figure 3:**
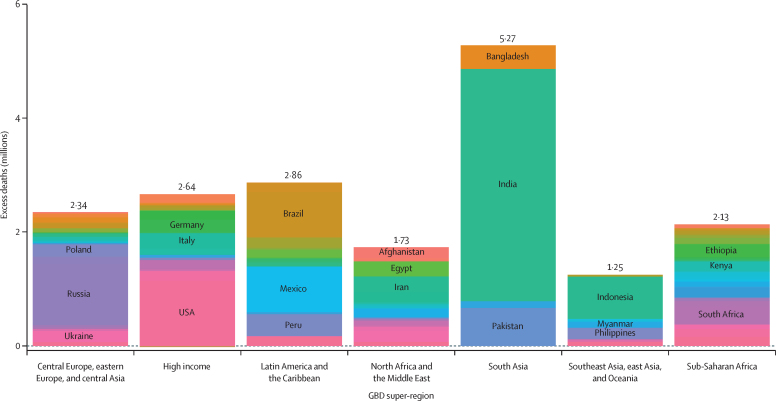
Stacked bar chart of regional distribution of excess deaths for 2020–21 The distribution of excess deaths by GBD super-region for the cumulative period. Countries labelled on each stacked bar are the three countries with the highest estimated excess deaths within the GBD super-region. GBD=Global Burden of Diseases, Injuries, and Risk Factors Study.

In terms of cumulative number of excess deaths due to the COVID-19 pandemic from Jan 1, 2020, to Dec 31, 2021, the highest numbers of deaths were estimated in India (4·07 million [95% UI 3·71–4·36]) and the USA (1·13 million [1·08–1·18]; figure 3, table). Five more countries had cumulative excess deaths exceeding 500 000 over this period: Russia (1·07 million [1·06–1·08]), Mexico (798 000 [741 000–867 000]), Brazil (792 000 [730 000–847 000]), Indonesia (736 000 [594 000–955 000]), and Pakistan (664 000 [498 000–847 000]). These seven countries accounted for more than half of the global excess deaths due to COVID-19 over the 24-month period. Among these countries, the excess mortality rate was highest in Russia (374·6 deaths [369·7–378·4] per 100 000) and Mexico (325·1 [301·6–353·3]), and was similar in Brazil (186·9 [172·2–199·8]) and the USA (179·3 [170·7–187·5]). An additional six countries individually had more than 250 000 excess deaths estimated during this period: Bangladesh (413 000 [347 000–504 000]), Peru (349 000 [328 000–367 000]), South Africa (302 000 [287 000–332 000]), Iran (274 000 [231 000–335 000]), Egypt (265 000 [248 000–278 000]), and Italy (259 000 [242 000–276 000]). These high-burden countries are distributed across all GBD regions of the world, reinforcing the evidence for the truly global nature of the pandemic. At the regional level, the number of excess deaths due to the COVID-19 pandemic was largest in the regions of south Asia, north Africa and the Middle East, and eastern Europe.

**Figure 4:**
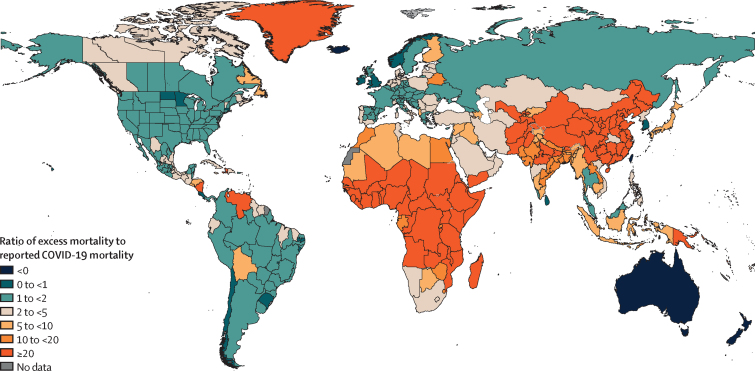
Global distribution of the ratio between estimated excess mortality rate due to the COVID-19 pandemic and reported COVID-19 mortality rate, for the cumulative period 2020–21

We also computed the ratio of excess mortality rate to reported COVID-19 mortality rate as a measurement of undercounting of the true mortality impact of the pandemic. There were wide variations in the ratio across and within regions (figure 4; table). In high-income North America, the ratios were comparatively low, ranging from −0·05 to 4·41 between states in the USA, and from 1·05 to 5·59 between provinces in Canada. The ratio was 2·21 (95% UI 2·07–2·39) for central Latin America, with higher ratios in some countries including El Salvador (7·04 [5·49–8·57]) and lower ratios in others such as Costa Rica (0·85 [0·74–0·99]). Within Mexico, ratios were higher than 2·0 in ten states and ranged from 1·02 (0·90–1·15) in Colima to 4·90 (4·34–5·46) in Chiapas. In almost all national and subnational locations in southern and tropical Latin America, the ratios were lower than 2 and for some locations lower than 1, with the only exceptions being the states of Maranhão and Ceará in Brazil, where the ratios of excess mortality rate to reported COVID-19 mortality rate were 3·17 (2·85–4·41) and 2·05 (1·85–2·32), respectively. In north Africa and the Middle East, the ratios were higher than 10 in Morocco (10·56 [8·46–13·64]), Egypt (12·19 [11·42–12·80]), Sudan (25·12 [18·00–30·17]), Afghanistan (26·06 [19·36–32·86]), and Yemen (33·04 [19·03–49·21]). In sub-Saharan Africa, ratios were generally high, with a median ratio of 29·59 (IQR 46·15). At the overall country level, the ratio ranged from −17·10 (−26·06 to −8·84) in New Zealand to 139·24 (88·86–213·67) in the Central African Republic. For South Africa, which was the only nation in sub-Saharan Africa where direct estimates of excess mortality based on vital registration data were possible, the ratio was 3·31 (3·15–3·64). In south Asia, national-level ratios ranged from 8·33 (7·58–8·92) in India to 36·06 (15·14–53·25) in Bhutan. The most extreme ratios in the region were found in the states and provinces of India and Pakistan, ranging from 0·96 (0·44–1·41) in Goa, India to 49·64 (28·94–72·74) in Balochistan, Pakistan.

Based largely on empirical excess mortality observed in 12 Indian states, including during the surge in COVID-19 cases between April and July, 2021, we found that at the national level, India had an estimated 152·5 excess deaths (95% UI 138·6–163·3) per 100 000 of the population over the study period. This was much higher than its reported COVID-19 mortality rate of 18·3 per 100 000 over the same period. Heterogeneity in excess mortality among the 30 states of India was extremely high. From Jan 1, 2020, to Dec 31, 2021, eight Indian states had excess mortality rates higher than 200 per 100 000, a level only exceeded by 50 other countries in the world. These states were Uttarakhand, Manipur, Maharashtra, Chhattisgarh, Haryana, Himachal Pradesh, Punjab, and Karnataka. Conversely, Arunachal Pradesh, Telangana, Sikkim, Rajasthan, Gujarat, Uttar Pradesh, Jharkhand, West Bengal, and Goa had excess mortality rates that were lower than the global average of 120·6 per 100 000 (although 95% UIs overlap). Similarly, subnational heterogeneity was evident in the excess death counts. Seven states in India had excess deaths higher than 200 000 as of Dec 31, 2021. These were West Bengal (220 000 [132 000–254 000]), Madhya Pradesh (223 000 [188 000–253 000]), Tamil Nadu (260 000 [199 000–301 000]), Karnataka (284 000 [223 000–327 000]), Bihar (323 000 [257 000–388 000]), Uttar Pradesh (517 000 [418 000–602 000]), and Maharashtra (616 000 [484 000–736 000]). Although the excess mortality rates due to the COVID-19 pandemic among Indian states are not the highest in the world, because of India's large population, the country accounted for 22·3% (20·3–23·9) of global excess deaths as of Dec 31, 2021 (data not shown). Bihar, Uttar Pradesh, and Maharashtra had excess deaths higher than South Africa (302 000 [287 000–332 000]), with South Africa ranking tenth among all countries.

## Discussion

Understanding the true mortality impact of the COVID-19 pandemic is crucial for public health decision making. Our analysis suggests that 18·2 million (95% UI 17·1–19·6) people died globally because of the COVID-19 pandemic (as measured by excess mortality) between the beginning of the pandemic (Jan 1, 2020) and the end of Dec 2021, which is 3·07-times (2·88–3·30) higher than the reported number of COVID-19-related deaths. Our findings indicate that the full impact of the pandemic has been much greater than what is suggested by official statistics. The highest COVID-19 excess mortality rates were observed in Andean Latin America, eastern Europe, and central Europe, with high mortality rates in many high-income northern hemisphere countries and similarly high rates in nearly all of Latin America. Comparatively low excess mortality rates were seen in east Asia, Australia, and high-income Asia Pacific. The April–August 2021 surge in south Asia brought cumulative excess mortality rates from the COVID-19 pandemic across that region up to or above the levels that were observed in some high-income countries. The cumulative global excess deaths from the pandemic makes COVID-19 potentially one of the leading global causes of death during the period of the pandemic, given the rates and trends in other causes of deaths before the pandemic.^22^

The magnitude of disease burden might have changed for many causes of death during the pandemic period due to both direct effects of lockdowns and the resulting economic turmoil. To correctly divide excess deaths into those directly due to SARS-CoV-2 infection and those associated with changes in other diseases and injuries, multiple drivers of change in mortality since the onset of the pandemic need to be considered. Unfortunately, to date only 36 countries have released 2020 cause of death data with detailed stratification by cause, to our knowledge. Although studies on specific populations in particular locations have been reported, we found insufficient statistical evidence to estimate these other changes in causes of death in a robust manner. Findings are mixed among the available evidence for alternative drivers of mortality that might have served to either increase or decrease excess mortality during the pandemic period. Some excess mortality might be attributable to reductions in health-care use across a number of causes;^14^, ^15^, ^24^, ^25^ however, the effect of reduced health-care use on health outcomes is difficult to prove. Many mechanisms have been proposed, including reduced vaccination rates^26^ and reduced births in hospitals.^27^, ^28^ However, demonstrated increases in cause-specific mortality from reduced health-care use do not have a generalised pattern in the countries with data released to date; the effect of changes in health-care use on excess mortality might also be greater in later years, rather than in 2020 or 2021. Even hypothesised decreases in injury-related deaths might be in doubt, as the US National Traffic and Safety Board has reported that road traffic deaths increased in 2020 in many US states despite decreases in transport mobility.^29^ There is convincing evidence that rates of anxiety and depression increased during the pandemic period, which might lead to increases in deaths from suicide;^30^, ^31^ however, to date, evidence of increased suicide mortality is scarce apart from in Japan, where reported suicide deaths increased during the pandemic.^32^ Conversely, opioid deaths have increased in some US states and decreased in others compared with expected levels.^33^, ^34^, ^35^, ^36^ Deaths from some chronic conditions such as ischaemic heart disease or chronic respiratory disease decreased in particular months of 2020,^25^ most notably in May and June in Europe; these decreases might have occurred because frail individuals who died from COVID-19 earlier in the year might otherwise have died from these chronic conditions. Supporting evidence for reductions in chronic conditions (particularly respiratory conditions) includes reductions in air pollution in the months of March and April in 2020,^37^ but with the possible exception of India,^38^ aggregated concentrations of air pollution during the entire pandemic period up to Dec 31, 2021 were only slightly lower than prepandemic concentrations. The most compelling evidence to date of a change in cause-specific mortality in the pandemic period is the decrease, especially in the Northern Hemisphere, in flu and respiratory syncytial virus (RSV) deaths seen in the months of January to March, 2021; decreases in cases of 80% or more have been reported to WHO. Given the scarce and inconsistent evidence of the effect the COVID-19 pandemic on cause-specific deaths, and the extremely scarce high-quality data on causes of death during the pandemic, our excess mortality estimates reflect the full impact of the pandemic on mortality around the world (after correcting for known biases), not just the deaths directly attributable to SARS-CoV-2 infection. As cause of death data for detailed causes are made available for 2020 for more countries in the future, we will be able to delineate the fraction of excess deaths due to SARS-CoV-2 infection versus indirect effects of the pandemic and responses to it. Separating the total number of deaths due to SARS-CoV-2 infection from excess mortality due to the pandemic will also be crucial for accurately estimating the infection–fatality ratio in every location, since that ratio requires the total number of deaths directly due to infection as an input.

Nonetheless, of the 12·3 million more excess deaths versus reported COVID-19 deaths, a substantial fraction of these will probably be shown to be from SARS-CoV-2 infection. This fraction, however, is likely to vary by location. For instance, in some high-income countries like Belgium and Sweden, which have used audits to investigate potential COVID-19-related deaths and have taken an inclusive approach to statistical reporting (available on the European Statistical Recovery Dashboard),^39^ the ratio between excess deaths and reported COVID-19 deaths is close to 1. This outcome suggests that in some locations, the net change in mortality rates across other diseases and injuries during the pandemic period has been minimal. Conversely, audited cause of death data from both Russia and Mexico indicate that a substantial proportion of excess deaths could not be attributed to SARS-CoV-2 infection in these locations. Although these and other findings suggest that a higher fraction of excess deaths are likely due to SARS-CoV-2 infection in high-income countries than elsewhere, sufficient evidence on this topic is too sparse to make any conclusion for all countries. As increased data on the pandemic period are eventually made available, more sophisticated analyses will be possible to dissect the effects of different drivers in different locations.

The highest ratios of excess deaths to reported COVID-19 deaths were observed in parts of central Asia and most countries in sub-Saharan Africa (excluding southern sub-Saharan Africa). These high ratios are unlikely to be fully explained by dramatic increases in other causes of death during the pandemic and must at least partly be related to a paucity in extensive testing, medical practices, or state guidance on what should count as a death from COVID-19. Behavioural data, available in our online tool, suggest that in comparison with high-income countries including Canada, many states in the USA, and some countries in western Europe, mask use across these regions was generally much lower throughout the pandemic; mobility, measured via cell phone application use, was much higher; and many fewer social distancing mandates were put in place. Furthermore, our variable selection analysis found that in addition to pandemic-related indicators such as IDR and mobility, background population health-related metrics such as HAQ Index, crude death rate, and inpatient admission rates are also predictive of excess mortality due to the COVID-19 pandemic. These factors might explain the large and under-reported COVID-19 epidemics in sub-Saharan Africa and central Asia, but more data are needed to fully understand the patterns in these regions. More attention should be given to locations like these, where dramatically low IDRs and rates of reported COVID-19 deaths have masked the true severity of the pandemic.

Weekly reporting of all-cause deaths has proven to be extremely useful in understanding the mortality impacts of the COVID-19 pandemic. Many countries have made a concerted effort to report data from vital registration in a much timelier manner than normal. Our analysis found that the lag between occurrence and registration of death varied substantially between countries, even between high-income countries with robust vital registration systems. Although such lags in reporting documented here show there are limitations to this rapid reporting in some countries such as the USA, these data have substantial value for epidemiological surveillance. Governments should try to use digital reporting of cause of death data and rapid release of the data to the public health community. If more countries invested in timely reporting, we would not only be able to track the effects of this pandemic and future pandemics, but we could more rapidly evaluate the effect of behavioural changes, such as social distancing, on mortality rates of other diseases and injuries.

At the global level, our excess mortality estimates are very similar to those reported by *The Economist*, the only other source of COVID-19 excess mortality at a global scale for a similar time period. For a majority of the countries and territories covered by both analyses (104 of 187), the uncertainty intervals from this study and those from *The Economist* overlap, meaning no statistically significant difference between them. However, there are dramatic differences in the estimated excess mortality counts between the two studies for many countries (appendix p 50). The relative difference between the estimates from each study, defined as the ratio between excess deaths from *The Economist* study over those from our study minus 1, ranges from −382·7% (Vanuatu) to 2282·3% (China). In terms of absolute relative difference, at least a 25% difference is observed in 129 of 187 countries. Some 23 countries have absolute relative differences between the two studies of higher than 100%. For the 121 countries and territories for which both studies predict more than 5000 excess deaths, the estimates from *The Economist* tend to be lower than in this study. Although the global total produced by *The Economist* was similar at 18·0 million (95% UI 12·9–21·0) excess deaths, which is about 212 000 deaths fewer than the estimate derived in this study, country contributions to the totals varied. *The Economist* estimated 192 000 fewer excess deaths for Mexico, 140 000 for the USA, and 140 000 for Peru, and 1·07 million additional excess deaths for India, 409 000 for China, and 193 000 for Sudan. For sub-Saharan Africa, the absolute relative differences range from 0·6% in Gabon to 310·7% in Burundi. The absolute relative difference is at least 50% among 21 out of 46 countries in the region.

This study has a number of limitations. First, different modelling strategies, although closely related, were used for the estimation of excess mortality due to COVID-19 depending on the availability of time series data on all-cause mortality before and during the pandemic. For countries without reporting of all-cause mortality by week or month, we used a statistical model to predict the COVID-19 excess mortality rate. Direct measurement would be preferable to modelled excess mortality estimates not based on all-cause mortality data, which are usually more robust, from the locations themselves. Second, we excluded from the assessment of excess mortality the most recent weeks and months in 2021 because of the evidence that late registration can take 20 weeks or longer to catch up. These exclusions help make our assessment closer to the true impact of COVID-19 for the period when data were kept in the analysis, improving the predictions made based on these input data for the entire modelled period. However, there is subjectivity to the exact limit on the intervals to be excluded because many countries have not provided weekly reports to formally evaluate late registration timing. Third, we estimated cumulative COVID-19 excess mortality rate, rather than estimating temporal excess mortality rate by week or month. This was intentional, as we found considerable lags in the reporting of COVID-19 death data compared with more precise data from vital statistics after allowing time for reporting of the day of death. In some cases, either all-cause mortality or COVID-19 mortality is tabulated by time of registration, rather than time of occurrence. The disconnect between timing of these two data streams leads to unrealistic values when COVID-19 mortality rate, a key covariate in predicting excess mortality rate, is used to predict time-detailed excess mortality rate. Simple simulation studies that we conducted (not shown) showed that even 1–2-week differences in reporting lags between the sources could lead to large swings in the weekly ratios between excess mortality and reported COVID-19 mortality. For this reason, we believe that analysing the cumulative excess mortality is much more robust. Fourth, as studies from a few selected countries including the Netherlands and Sweden have suggested,^40^ we suspect most of the excess mortality during the pandemic is from COVID-19. However, sufficient empirical evidence is absent in most countries. Given the high amount of heterogeneity in epidemiological profiles among countries, it is prudent not to make such strong assumptions before more research on this topic is done. Fifth, although our variable selection process produced 15 covariates that we included in our final model, inclusion of other variables might have improved our model predictions. Sixth, strict lockdown and mediation interventions can lead to negative excess mortality during the pandemic (ie, expected mortality is higher than observed all-cause mortality during the pandemic). We estimated that several countries, including Australia and New Zealand, have had negative excess mortality during the pandemic. This observation is probably due to decreases in mortality from diseases and injuries for which exposure to related risks has been reduced during the pandemic. In our prediction model for locations without time-detailed all-cause mortality before and during the pandemic, we did not attempt to predict negative excess mortality rates, as such evidence is not strong enough from a small number of locations to extrapolate to the whole world. Future studies are warranted to examine the effect of lockdown on changes in specific causes of death. Related to this decision not to predict negative excess mortality, our excess mortality estimates for the provinces in China were driven by covariates only. Although we recognise that similar, if not stricter, lockdown practices have been implemented in China compared with those we have seen in countries such as Australia and New Zealand, there is an absence of empirical information on vital registration data to make proper excess mortality estimates with the models we have developed. As more information becomes available in the near future, further improvements to our estimates are warranted. Seventh, various drivers are responsible for the changes in all-cause and cause-specific mortality in a population. Therefore, in estimating excess mortality due to COVID-19, the roles of confounders of changes in mortality during the pandemic should be resolved. We excluded mortality spikes in selected summer weeks in Europe that coincided with a heatwave, when the pandemic was at its lowest point in terms of transmission. However, scarcity of empirical evidence on other confounders and the information on cause-specific mortality that is associated with such confounders prevented us from further and more detailed data analysis. As the body of evidence grows in the future on both potential confounders and cause-specific mortality, such information should be incorporated into future iterations of the analysis presented in this study. Eighth, our excess mortality estimates are based on all-age mortality data, male and female data combined, from vital registration systems, and therefore cannot be disaggregated by age or sex. COVID-19 disproportionally affects older and fragile populations, and we should expect varying age patterns of excess mortality based on a population's demographic and epidemiological profiles. Unfortunately, weekly and monthly vital registration data by age and sex for 2020 and 2021 are currently too sparse (and tend to be in broad age groups when available) to robustly model excess mortality by age and sex, or to use to build models that predict this age pattern across countries with acceptable out-of-sample predictive validity. We intend to evaluate age-specific and sex-specific excess mortality in future iterations of this analysis when data disaggregated by age and sex become more widely available. Finally, the development and deployment of SARS-COV-2 vaccines have considerably lowered mortality rates among people who contract the virus and among the general population. As a result, we expect trends in excess mortality due to COVID-19 to change over time as the coverage of vaccination increases among populations and as new variants emerge. There will be great value in continuing to estimate excess mortality over time as these and other factors shift.

The full magnitude of the COVID-19 pandemic has been much greater in 2020 and 2021 than is indicated by reported deaths due to COVID-19, with COVID-19 potentially being a leading cause of mortality in 2020 and 2021. Further research and increased availability of cause of death data will be crucial for distinguishing the proportion of excess mortality that was directly caused by SARS-CoV-2 infection. Finding ways to strengthen death reporting systems and mitigate political barriers to accurate reporting will be important for tracking and monitoring the continuation of the existing COVID-19 pandemic and future pandemic events.

## Data sharing

The data sources used in these analyses are available to download on the Global Health Data Exchange website. Reported COVID-19 death data sources are also listed by location and institution in the appendix (pp 8–37).

**This online publication has been corrected. The corrected version first appeared at thelancet.com on April 14, 2022**

## Declaration of interests

C Adolph reports support for the present manuscript from the Benificus Foundation for collection of data on state-level social distancing policies in the USA. X Dai reports support for the present manuscript from paid salary through employment at the Institute for Health Metrics and Evaluation and the University of Washington. N Fullman reports funding support from WHO as a consultant from June to September, 2019, and Gates Ventures since June, 2020, all outside the submitted work. S Nomura reports support for the present manuscript from a Ministry of Education, Culture, Sports, Science and Technology of Japan grant. D M Pigott reports support for the present manuscript from the Bill & Melinda Gates Foundation. D M Pigott also reports grants or contracts from the Bill & Melinda Gates Foundation, outside the submitted work. All other authors declare no competing interests.
