
JOURNAL INFORMATION
==============================
NLM Title Abbreviation: Lancet
Journal ID: Lancet
NLM Journal ID: 2985213R

Lancet (London, England)

ISSN: 0140-6736
EISSN: 1474-547X

ARTICLE INFORMATION
==============================
PMCID: PMC9009836
PMID: 35430019
DOI: 10.1016/S0140-6736(22)00621-3
Article ID: S0140-6736(22)00621-3
Article version: 2
Subjects: Department of Error

Department of Error

Print publication date: 2022 Apr 16
Volume: 399
Issue: 10334
First page: 1468
Last page: 1468
Copyright: © 2022 The Author(s). Published by Elsevier Ltd.
Copyright year: 2022
Copyright holder: Elsevier Ltd
License: This is an open access article under the CC BY license (http://creativecommons.org/licenses/by/4.0/).
License URL: https://creativecommons.org/licenses/by/4.0/

Erratum for: Estimating excess mortality due to the COVID-19 pandemic: a systematic analysis of COVID-19-related mortality, 2020–21 399 10334 16 4 2022 1513 1536 Lancet (London, England) 10.1016/S0140-6736(21)02796-3 PMC8912932 35279232

==============================
COVID-19 Excess Mortality Collaborators. Estimating excess mortality due to the COVID-19 pandemic: a systematic analysis of COVID-19-related mortality, 2020–21. Lancet 2022; 399: 1513–36—In this Article, Tanzania and Uganda should have been listed under eastern sub-Saharan Africa in the table. The totals for eastern sub-Saharan Africa correctly included Tanzania and Uganda in the original version and have not been changed. The uncertainty intervals for The Economist estimate of excess deaths in the Research in Context panel and Discussion section have been updated to 18·0 million (95% uncertainty interval 12·9–21·0). These corrections have been made to the online version as of April 14, 2022, and the printed version is correct.

