# Supplementary material for: Estimating excess mortality due to the COVID-19 pandemic: a systematic analysis of COVID-19-related mortality, 2020–21
Source: Lancet. 2022 Apr 16;399(10334):1513–36. doi: 10.1016/S0140-6736(21)02796-3 (PMC8912932; doi:10.1016/S0140-6736(21)02796-3)
Supplement: Supplementary appendix [file mmc1.pdf]

THE LANCET

Supplementary appendix

This appendix formed part of the original submission and has been peer reviewed. We post it as supplied by the authors.

Supplement to: COVID-19 Excess Mortality Collaborators. Estimating excess mortality due to the COVID-19 pandemic: a systematic analysis of COVID-19-related mortality, 2020–21. *Lancet* 2022; published online March 10. https://doi.org/10.1016/S0140-6736(21)02796-3.

Appendix to "Estimating excess mortality due to SARS-CoV-2 pandemic: a systematic analysis of COVID-19 mortality, 2020–2021."

Table of contents

Section 1. Overview ... 3

Section 2. GATHER checklist ... 3

Table S1. Checklist for compliance with the Guidelines for Accurate and Transparent Health Estimates Reporting (GATHER) ... 3

Section 3. Input Data ... 4

Table S2. Locations for which input data were available, including data source, whether input is weekly or monthly, and dates covered by the data. ... 4

Table S3. Sources used for reported COVID-19 mortality ... 8

Section 4. Methods ... 38

Section 4.1 Estimating expected mortality ... 38

Section 4.1.1 REGMOD ... 38

Section 4.1.2 Poisson regression ... 39

Section 4.1.3 Last-year ... 39

Section 4.1.4 Ensemble ... 39

Table S4. Root Mean Squared Error and weights for models used in the Ensemble modelling process ... 40

Section 4.2 Predicting excess mortality for all locations ... 40

Section 4.2.1 Compiling a database on excess mortality rate and its relevant covariates for various time period by location after accounting for late registration and under registration. ... 40

Section 4.2.2 A statistical model for predicting excess mortality rate ... 41

Table S5. Covariates selected for use in the final model with direction of influence ... 41

Table S6. List of covariates assessed for use in the model ... 42

Section 4.2.3. Predict excess mortality rate for the cumulative period of January 1 2020 and December, 31 2021 ... 43

Section 5. References ... 43

Section 6. Author contributions ... 45

Managing the estimation or publication process ... 45

Writing the first draft of the manuscript ... 45

Primary responsibility for applying analytical methods to produce estimates ... 45

Primary responsibility for seeking, cataloguing, extracting, or cleaning data; designing or coding figures and tables ... 45

Providing data or critical feedback on data sources ... 45

Developing methods or computational machinery ... 45

Providing critical feedback on methods or results ... 45

Drafting the work or revising it critically for important intellectual content ... 45

Managing the overall research enterprise 46
Section 7. Supplemental Tables and Figures 47
Figure S1. Map of all cause data availability 47
Figure S2. Map of reported Covid19 mortality data availability 47
Figure S3. Estimating expected mortality based on seasonality and secular trends 48
Figure S4. (a) The distribution of RMSE by location for each of six models included in the ensemble model, and (b) the estimated excess mortality for Spain for each component model and for the ensemble. 49
Figure S5. Comparison to *The Economist* excess mortality estimates 50
Figure S6. Late registration in all-cause deaths by countries and territories 51

Section 1. Overview

This Appendix accompanies the paper "Estimating excess mortality due to SARS-CoV-2 pandemic: a systematic analysis of COVID-19 mortality, 2020–2021."

The aim of this paper is to use a novel method to estimate total excess deaths attributable to the COVID-19 pandemic from (a) weekly and monthly all-cause death counts for before and during the pandemic, and (b) official reported counts of COVID-19 deaths. We compile all-cause mortality data from 74 countries and territories and use our model to make estimates of excess mortality for a total of 191 countries and territories.

In the Appendix we describe, in detail, the input data and methods, and include supplemental results. We also include a checklist to demonstrate compliance with the Guidelines for Accurate and Transparent Health Estimates Reporting (GATHER).[1]

Section 2. GATHER checklist

Table S1. Checklist for compliance with the Guidelines for Accurate and Transparent Health Estimates Reporting (GATHER)

Item #	Checklist item	Reported on page #
**Objectives and funding**		
**1**	Define the indicator(s), populations (including age, sex, and geographic entities), and time period(s) for which estimates were made.	Main text: Methods (Overview)
**2**	List the funding sources for the work.	Main text (summary and acknowledgments)
**Data Inputs**		
For all data inputs from multiple sources that are synthesized as part of the study:		
**3**	Describe how the data were identified and how the data were accessed.	Main text (Methods) and appendix (section 3)
**4**	Specify the inclusion and exclusion criteria. Identify all ad-hoc exclusions.	Main text (Methods) and appendix (section 3)
**5**	Provide information on all included data sources and their main characteristics. For each data source used, report reference information or contact name/institution, population represented, data collection method, year(s) of data collection, sex and age range, diagnostic criteria or measurement method, and sample size, as relevant.	Methods appendix (section 3), online data citation tools: http://ghdx.healthdata.org/record/ihme-data/covid_19_excess_mortality
**6**	Identify and describe any categories of input data that have potentially important biases (e.g., based on characteristics listed in item 5).	Main text (Methods) and appendix (section 3)
For data inputs that contribute to the analysis but were not synthesized as part of the study:		
**7**	Describe and give sources for any other data inputs.	Online data citation tools: http://ghdx.healthdata.org/record/ihme-data/covid_19_excess_mortality
For all data inputs:		
**8**	Provide all data inputs in a file format from which data can be efficiently extracted (e.g., a spreadsheet rather than a PDF), including all relevant meta-data listed in item 5. For any data inputs that cannot be shared because of ethical or legal reasons, such as third-party ownership, provide a contact name or the name of the institution that retains the right to the data.	Online data citation tools: http://ghdx.healthdata.org/record/ihme-data/covid_19_excess_mortality
**Data analysis**		
**9**	Provide a conceptual overview of the data analysis method. A diagram may be helpful.	Main text (Methods) and appendix (section 4)
**10**	Provide a detailed description of all steps of the analysis, including mathematical formulae. This description should cover, as relevant, data cleaning, data pre-processing, data adjustments and weighting of data sources, and mathematical or statistical model(s).	Main text (Methods) and appendix (section 4)
**11**	Describe how candidate models were evaluated and how the final model(s) were selected.	Appendix (section 4)
**12**	Provide the results of an evaluation of model performance, if done, as well as the results of any relevant sensitivity analysis.	Appendix (section 4)
**13**	Describe methods for calculating uncertainty of the estimates. State which sources of uncertainty were, and were not, accounted for in the uncertainty analysis.	Appendix (section 4)
**14**	State how analytic or statistical source code used to generate estimates can be accessed.	Github link: https://github.com/ihmeuw-demographics/publication_covid_em
**Results and Discussion**		

**15**	Provide published estimates in a file format from which data can be efficiently extracted.	Online tools: http://ghdx.healthdata.org/record/ihme-data/covid_19_excess_mortality
**16**	Report a quantitative measure of the uncertainty of the estimates (e.g. uncertainty intervals).	95% UIs are given for all findings, including in the main text, methods appendix, and online tools (Online data citation tools: http://ghdx.healthdata.org/record/ihme-data/covid_19_excess_mortality)
**17**	Interpret results in light of existing evidence. If updating a previous set of estimates, describe the reasons for changes in estimates.	Discussion; appendix figure S5; description for updates available at: https://www.healthdata.org/covid/updates
**18**	Discuss limitations of the estimates. Include a discussion of any modelling assumptions or data limitations that affect interpretation of the estimates.	Main text (Methods and Discussion [limitation section]) and appendix

Section 3. Input Data

To estimate total COVID-19 mortality, we collated the following inputs:

1. Weekly all-cause mortality data for 2010 to present. For some locations, monthly inputs were used because weekly values were not available (Table S2). Where data back to 2010 was not available, we extracted all available death counts between 2010 and present.
2. Estimates of excess mortality from COVID-19 from publications that have access to data from locations not available to us.
3. Reported COVID-19 mortality for all locations in the analysis
4. Other covariates pertaining to both the COVID-19 pandemic and background population health metrics for all locations in the analysis.

Table S2 provides more details on the sources used for (1) and (2) above.

Table S2. Locations for which input data were available, including data source, whether input is weekly or monthly, and dates covered by the data.

Location	Source	Time unit	Time period
Macao Special Administrative Region of China	Macao Monthly Bulletin of Statistics	Month	Dec 2015 - Nov 2021
Hong Kong Special Administrative Region of China	Hong Kong Monthly Digest of Statistics	Month	Jan 2018 - Nov 2021
Taiwan (Province of China)	Taiwan Human Mortality Database Short-term Mortality Fluctuations (STMF); Taiwan Monthly Bulletin of Interior Statistics; World Mortality Database (WMD)	Month	Jan 2010 - Dec 2021
Philippines	Philippines Vital Registration	Month	Jan 2015 - Nov 2021
Philippines, subnational	Philippines Vital Registration	Month	Jan 2015 - Nov 2021
Thailand	Thailand Monthly Death Count Statistics	Month	Jan 2015 - Dec 2021
South Africa	South Africa Report on Weekly Deaths	Week	Week 1, 2014 - Week 52, 2021
Armenia	Armenia Deaths by Week, Sex and 5-year Age Group - Eurostat	Week	Week 1, 2017 - Week 38, 2021
Azerbaijan	Azerbaijan Socioeconomic Indicators January-November 2021	Month	Jan 2018 - Nov 2021
Georgia	Georgia Summary Vital Statistics, January-June 2020 (Preliminary Data)	Week	Week 14, 2015 - Week 13, 2021
Kazakhstan	Kazakhstan Monthly Number of Deaths; World Mortality Database (WMD)	Month	Jan 2018 - Nov 2021
Kyrgyzstan	Kyrgyzstan Natural Movement of the Population	Month	Jan 2018 - Nov 2021
Mongolia	Mongolia Deaths by Region and Month	Month	Jan 2016 - Dec 2021

Location	Source	Time unit	Time period
Uzbekistan	Uzbekistan Deaths by Month of Death - United Nations Statistics Division; Uzbekistan Demographic Situation January-December 2021; World Mortality Database (WMD)	Month	Jan 2013 - Dec 2021
Albania	Albania Deaths by Week, Sex and 5-year Age Group - Eurostat	Week	Week 1, 2017 - Week 37, 2021
Bosnia and Herzegovina	Bosnia And Herzegovina Natural Population Movement And Marriages	Month	Jan 2018 - Sep 2021
Bulgaria	Bulgaria Human Mortality Database Short-term Mortality Fluctuations (STMF)	Week	Week 1, 2010 - Week 52, 2021
Croatia	Croatia Human Mortality Database Short-term Mortality Fluctuations (STMF)	Week	Week 1, 2010 - Week 52, 2021
Czechia	Czech Republic Human Mortality Database Short-term Mortality Fluctuations (STMF)	Week	Week 1, 2010 - Week 51, 2021
Hungary	Hungary Human Mortality Database Short-term Mortality Fluctuations (STMF)	Week	Week 1, 2010 - Week 52, 2021
North Macedonia	North Macedonia Deaths by Month of Death - United Nations Statistics Division; North Macedonia Monthly Statistical Report; World Mortality Database (WMD)	Month	Jan 2010 - Nov 2021
Montenegro	Montenegro Monthly Statistical Review	Month	Jan 2017 - Oct 2021
Poland	Poland Human Mortality Database Short-term Mortality Fluctuations (STMF)	Week	Week 1, 2010 - Week 52, 2021
Romania	Romania Deaths by Week, Sex and 5-year Age Group - Eurostat	Week	Week 1, 2017 - Week 47, 2021
Serbia	Serbia Deaths by Week, Sex and 5-year Age Group - Eurostat	Week	Week 1, 2017 - Week 47, 2021
Slovakia	Slovakia Human Mortality Database Short-term Mortality Fluctuations (STMF)	Week	Week 1, 2010 - Week 51, 2021
Slovenia	Slovenia Human Mortality Database Short-term Mortality Fluctuations (STMF)	Week	Week 1, 2010 - Week 52, 2021
Belarus	Belarus Deaths by Month of Death - United Nations Statistics Division; World Mortality Database (WMD)	Month	Jan 2011 - Mar 2021
Estonia	Estonia Human Mortality Database Short-term Mortality Fluctuations (STMF)	Week	Week 1, 2010 - Week 52, 2021
Latvia	Latvia Human Mortality Database Short-term Mortality Fluctuations (STMF)	Week	Week 1, 2010 - Week 52, 2021
Lithuania	Lithuania Human Mortality Database Short-term Mortality Fluctuations (STMF)	Week	Week 1, 2010 - Week 52, 2021
Moldova	Moldova Deaths by Month of Death - United Nations Statistics Division; Moldova Quarterly Statistical Bulletin	Month	Jan 2014 - Sep 2021
Russia	Russia Natural Movement of the Population; World Mortality Database (WMD)	Month	Jan 2016 - Dec 2021
Russia, subnational	Russia Natural Movement of the Population	Month	Jan 2016 - Nov 2021
Ukraine	Ukraine Deaths by Month of Death - United Nations Statistics Division; Ukraine Number of Live Births, Deaths January-December 2020, by Region; Ukraine Number of Live Births, Deaths January-November 2021, by Region	Month	Jan 2010 - Nov 2021
Japan	Japan Excess and Exiguous Deaths Dashboard - Weekly Deaths	Week	Week 1, 2010 - Week 38, 2021
Japan, subnational	Japan Excess and Exiguous Deaths Dashboard - Weekly Deaths	Week	Week 1, 2010 - Week 38, 2021
South Korea	South Korea Human Mortality Database Short-term Mortality Fluctuations (STMF)	Week	Week 1, 2010 - Week 47, 2021
Singapore	Singapore Deaths By Ethnic Group And Sex, Monthly	Month	Jan 2010 - Sep 2021
Australia	World Mortality Database (WMD)	Week	Week 1, 2015 - Week 43, 2021
New Zealand	New Zealand Human Mortality Database Short-term Mortality Fluctuations (STMF)	Week	Week 1, 2011 - Week 52, 2021
Austria	Austria Human Mortality Database Short-term Mortality Fluctuations (STMF)	Week	Week 1, 2010 - Week 52, 2021

Location	Source	Time unit	Time period
Belgium	Belgium Human Mortality Database Short-term Mortality Fluctuations (STMF)	Week	Week 1, 2010 - Week 52, 2021
Cyprus	Cyprus Deaths by Week, Sex and 5-year Age Group - Eurostat	Week	Week 1, 2017 - Week 47, 2021
Denmark	Denmark Human Mortality Database Short-term Mortality Fluctuations (STMF)	Week	Week 1, 2010 - Week 52, 2021
Finland	Finland Human Mortality Database Short-term Mortality Fluctuations (STMF)	Week	Week 1, 2010 - Week 51, 2021
France	France Human Mortality Database Short-term Mortality Fluctuations (STMF)	Week	Week 1, 2010 - Week 52, 2021
Germany	Germany Human Mortality Database Short-term Mortality Fluctuations (STMF)	Week	Week 1, 2016 - Week 52, 2021
Greece	Greece Human Mortality Database Short-term Mortality Fluctuations (STMF)	Week	Week 1, 2016 - Week 52, 2021
Iceland	Iceland Human Mortality Database Short-term Mortality Fluctuations (STMF)	Week	Week 1, 2010 - Week 52, 2021
Ireland	Ireland - Measuring Mortality Using Public Data Sources; Ireland Daily Death Notices; World Mortality Database (WMD)	Month	Jan 2015 - Nov 2021
Israel	Israel Human Mortality Database Short-term Mortality Fluctuations (STMF)	Week	Week 1, 2010 - Week 52, 2021
Italy	Italy Human Mortality Database Short-term Mortality Fluctuations (STMF)	Week	Week 1, 2011 - Week 47, 2021
Italy, subnational	Italy Daily Mortality Data 2011-2021	Week	Week 1, 2011 - Week 48, 2021
Luxembourg	Luxembourg Human Mortality Database Short-term Mortality Fluctuations (STMF)	Week	Week 1, 2010 - Week 52, 2021
Malta	Malta Deaths by Week, Sex and 5-year Age Group - Eurostat	Week	Week 1, 2017 - Week 52, 2021
Netherlands	Netherlands Human Mortality Database Short-term Mortality Fluctuations (STMF)	Week	Week 1, 2010 - Week 52, 2021
Norway	Norway Human Mortality Database Short-term Mortality Fluctuations (STMF)	Week	Week 1, 2010 - Week 52, 2021
Portugal	Portugal Human Mortality Database Short-term Mortality Fluctuations (STMF)	Week	Week 1, 2010 - Week 52, 2021
San Marino	San Marino Statistical Bulletin; World Mortality Database (WMD)	Month	March, 2016 - Nov, 2021
Spain	Spain Human Mortality Database Short-term Mortality Fluctuations (STMF)	Week	Week 1, 2010 - Week 52, 2021
Sweden	Sweden Human Mortality Database Short-term Mortality Fluctuations (STMF)	Week	Week 1, 2010 - Week 52, 2021
Switzerland	Switzerland Human Mortality Database Short-term Mortality Fluctuations (STMF)	Week	Week 1, 2010 - Week 52, 2021
England	United Kingdom - England and Wales Deaths Registered Weekly 2021	Week	Week 1, 2017 - Week 52, 2021
Northern Ireland	Northern Ireland Human Mortality Database Short-term Mortality Fluctuation	Week	Week 2, 2015 - Week 52, 2021
Scotland	Scotland Human Mortality Database Short-term Mortality Fluctuations (STMF)	Week	Week 1, 2010 - Week 52, 2021
Wales	United Kingdom - England and Wales Deaths Registered Weekly 2021	Week	Week 1, 2017 - Week 52, 2021
Chile	Chile Human Mortality Database Short-term Mortality Fluctuations (STMF)	Week	Week 1, 2016 - Week 52, 2021
Canada	Canada Human Mortality Database Short-term Mortality Fluctuations (STMF)	Week	Week 1, 2010 - Week 41, 2021
Greenland	Greenland Preliminary Deaths Report by Month	Month	Jan 2018 – Sep 2021
USA	United States Human Mortality Database Short-term Mortality Fluctuations (STMF); World Mortality Database (WMD)	Week	Week 2, 2015 - Week 52, 2021
USA, subnational	United States NVSS; United States Weekly Counts of Deaths by State and Select Causes	Month	Jan 2010 - Dec 2021
Puerto Rico	United States NVSS; United States Weekly Counts of Deaths by State and Select Causes	Month	Jan 2010 - Dec 2021
Saint Kitts and Nevis	Saint Kitts and Nevis Reported Deaths	Month	Jan 2016 - Dec 2020
Ecuador	Ecuador General Deaths	Week	Week 1, 2016 - Week 52, 2021

Location	Source	Time unit	Time period
Peru	Peru Death Information System (SINADEF) - System Report of Daily Deaths	Month	Jan 2017 - Dec 2021
Antigua and Barbuda	Antigua and Barbuda Deaths by Month and Sex	Month	Jan 2010 - Dec 2020
Cuba	Cuba Demographic Yearbook	Month	Jan 2010 - Dec 2020
Colombia	Colombia Vital Statistics; Colombia Excess Mortality - Non-Fetal Deaths Per Week 2020-2021	Month	Jan 2018 - Dec 2021
Costa Rica	Costa Rica Deaths by Month of Death - United Nations Statistics Division; Costa Rica Deaths 2020: the Impact of COVID-19 (preliminary); Costa Rica Registered Deaths 2020 (Preliminary)	Month	Jan 2010 - Dec 2020
Guatemala	Guatemala Vital Statistics; World Mortality Database (WMD)	Month	Jan 2013 - Aug 2021
Mexico	Mexico Vital Registration – Deaths; Mexico Database of the Statistical Bulletin on Excess Mortality 2020-2021; World Mortality Database (WMD)	Week	Week 1, 2010 - Week 49, 2021
Mexico, subnational	Mexico Vital Registration – Deaths	Week	Jan 2010 – Dec 2020
Brazil	Brazil Mortality Information System – Deaths; Brazil Mortality Information System - Deaths (Preliminary); Brazil Analysis Panel of Excess Mortality from Natural Causes	Week	Week 1, 2010 - Week 35, 2021
Brazil, subnational	Brazil Mortality Information System – Deaths; Brazil Mortality Information System - Deaths (Preliminary); Brazil Analysis Panel of Excess Mortality from Natural Causes	Week	Week 1, 2010 - Week 35, 2021
Paraguay	Paraguay Vital Statistics Sub System (SSIEV) Multi Reporting - Deaths	Month	Jan 2017 - Dec 2021
Egypt	Egypt Deaths by Month of Death - United Nations Statistics Division	Month	Jan 2010 - Jun 2021
Oman	Oman Vital Registration – Deaths; Oman Monthly Statistical Bulletin	Month	Jul 2017 - Dec 2021
Qatar	Qatar Vital Statistics Annual Bulletin; Qatar Monthly Statistics Statistics; World Mortality Database (WMD)	Month	Jan 2018 - Dec 2021
Input excess mortality from other publications			
Iran	Excess deaths associated with the Iranian COVID-19 epidemic: A province-level analysis	Week	Week 38, 2020
Mumbai	India - Mumbai Monthly Burials	Month	July 2020
South Africa, subnational	South Africa Report on Weekly Deaths	Week	Week 18, 2020 – Week 52, 2021
Input excess mortality from other publications for India States			
Andrah Pradesh	Excess mortality in India from June 2020 to June 2021 during the COVID pandemic: death registration, health facility deaths, and survey data. Preprint; Andhra Pradesh saw 400% increase in deaths in May, Tamil Nadu saw more modest excess mortality	Month	July 2020 - Oct 2020; Jan 2021 - May 2021
Assam	Excess mortality in India from June 2020 to June 2021 during the COVID pandemic: death registration, health facility deaths, and survey data. Preprint; Assam saw 28,000 more deaths than normal in months when first wave of Covid-19 struck	Month	July 2020 - Oct 2020; Jan 2021 - May 2021
Chhattisgarh	Chhattisgarh's excess deaths at least 4.8 times COVID-19 toll	Month	April - May 2021
Gujarat	Excess mortality in India from June 2020 to June 2021 during the COVID pandemic: death registration, health facility deaths, and survey data. Preprint	Month	March 2020 - May 2021
Haryana	Excess mortality in India from June 2020 to June 2021 during the COVID pandemic: death	Month	July 2020 - Dec 2020, April 2021 - May 2021

Location	Source	Time unit	Time period
	registration, health facility deaths, and survey data. Preprint		
Karnataka	Karnataka recorded 1.02 lakh ‘excess’ deaths in 2021, 5 times the COVID-19 toll	Month	Jan 2021 - June 2021
Kerala	Excess mortality in India from June 2020 to June 2021 during the COVID pandemic: death registration, health facility deaths, and survey data. Preprint	Month	Aug 2020 - May 2021
Madhya Pradesh	Excess mortality in India from June 2020 to June 2021 during the COVID pandemic: death registration, health facility deaths, and survey data. Preprint; Madhya Pradesh saw nearly three times more deaths than normal after second wave of Covid-19 struck	Month	July 2020 - May 2021
Odisha	Excess mortality in India from June 2020 to June 2021 during the COVID pandemic: death registration, health facility deaths, and survey data. Preprint	Month	Jan 2021 - June 2021
Rajasthan	Excess deaths in Rajasthan are at least five times the official COVID-19 tally	Month	April 2020 - May 2021
Tamil Nadu	Andhra Pradesh saw 400% increase in deaths in May, Tamil Nadu saw more modest excess mortality	Month	April 2020 - May 2021
Uttar Pradesh	Uttar Pradesh - 24 districts reported 110% more deaths between July and March than same period the previous year	Month	July 2020 - March 2021

Table S3. Sources used for reported COVID-19 mortality

Location	Source
Afghanistan	Johns Hopkins University. 2019 Novel Coronavirus COVID-19 (2019-nCoV) Data Repository by Johns Hopkins CSSE. Baltimore, Maryland: Johns Hopkins University.
Albania	Johns Hopkins University. 2019 Novel Coronavirus COVID-19 (2019-nCoV) Data Repository by Johns Hopkins CSSE. Baltimore, Maryland: Johns Hopkins University.
Algeria	Johns Hopkins University. 2019 Novel Coronavirus COVID-19 (2019-nCoV) Data Repository by Johns Hopkins CSSE. Baltimore, Maryland: Johns Hopkins University.
American Samoa	Johns Hopkins University. 2019 Novel Coronavirus COVID-19 (2019-nCoV) Data Repository by Johns Hopkins CSSE. Baltimore, Maryland: Johns Hopkins University.
Andorra	Johns Hopkins University. 2019 Novel Coronavirus COVID-19 (2019-nCoV) Data Repository by Johns Hopkins CSSE. Baltimore, Maryland: Johns Hopkins University.
Angola	Johns Hopkins University. 2019 Novel Coronavirus COVID-19 (2019-nCoV) Data Repository by Johns Hopkins CSSE. Baltimore, Maryland: Johns Hopkins University.
Antigua and Barbuda	Johns Hopkins University. 2019 Novel Coronavirus COVID-19 (2019-nCoV) Data Repository by Johns Hopkins CSSE. Baltimore, Maryland: Johns Hopkins University.
Argentina	Johns Hopkins University. 2019 Novel Coronavirus COVID-19 (2019-nCoV) Data Repository by Johns Hopkins CSSE. Baltimore, Maryland: Johns Hopkins University.

Armenia	Johns Hopkins University. 2019 Novel Coronavirus COVID-19 (2019-nCoV) Data Repository by Johns Hopkins CSSE. Baltimore, Maryland: Johns Hopkins University.
Australia	Johns Hopkins University. 2019 Novel Coronavirus COVID-19 (2019-nCoV) Data Repository by Johns Hopkins CSSE. Baltimore, Maryland: Johns Hopkins University.
Austria	Johns Hopkins University. 2019 Novel Coronavirus COVID-19 (2019-nCoV) Data Repository by Johns Hopkins CSSE. Baltimore, Maryland: Johns Hopkins University.
Azerbaijan	Johns Hopkins University. 2019 Novel Coronavirus COVID-19 (2019-nCoV) Data Repository by Johns Hopkins CSSE. Baltimore, Maryland: Johns Hopkins University.
Bahrain	Johns Hopkins University. 2019 Novel Coronavirus COVID-19 (2019-nCoV) Data Repository by Johns Hopkins CSSE. Baltimore, Maryland: Johns Hopkins University.
Bangladesh	Johns Hopkins University. 2019 Novel Coronavirus COVID-19 (2019-nCoV) Data Repository by Johns Hopkins CSSE. Baltimore, Maryland: Johns Hopkins University.
Barbados	Johns Hopkins University. 2019 Novel Coronavirus COVID-19 (2019-nCoV) Data Repository by Johns Hopkins CSSE. Baltimore, Maryland: Johns Hopkins University.
Belarus	Johns Hopkins University. 2019 Novel Coronavirus COVID-19 (2019-nCoV) Data Repository by Johns Hopkins CSSE. Baltimore, Maryland: Johns Hopkins University.
Belgium	Johns Hopkins University. 2019 Novel Coronavirus COVID-19 (2019-nCoV) Data Repository by Johns Hopkins CSSE. Baltimore, Maryland: Johns Hopkins University.
Belize	Johns Hopkins University. 2019 Novel Coronavirus COVID-19 (2019-nCoV) Data Repository by Johns Hopkins CSSE. Baltimore, Maryland: Johns Hopkins University.
Benin	Johns Hopkins University. 2019 Novel Coronavirus COVID-19 (2019-nCoV) Data Repository by Johns Hopkins CSSE. Baltimore, Maryland: Johns Hopkins University.
Bermuda	Johns Hopkins University. 2019 Novel Coronavirus COVID-19 (2019-nCoV) Data Repository by Johns Hopkins CSSE. Baltimore, Maryland: Johns Hopkins University.
Bhutan	Johns Hopkins University. 2019 Novel Coronavirus COVID-19 (2019-nCoV) Data Repository by Johns Hopkins CSSE. Baltimore, Maryland: Johns Hopkins University.
Bolivia	Johns Hopkins University. 2019 Novel Coronavirus COVID-19 (2019-nCoV) Data Repository by Johns Hopkins CSSE. Baltimore, Maryland: Johns Hopkins University.
Bosnia and Herzegovina	Johns Hopkins University. 2019 Novel Coronavirus COVID-19 (2019-nCoV) Data Repository by Johns Hopkins CSSE. Baltimore, Maryland: Johns Hopkins University.
Botswana	Johns Hopkins University. 2019 Novel Coronavirus COVID-19 (2019-nCoV) Data Repository by Johns Hopkins CSSE. Baltimore, Maryland: Johns Hopkins University.
Brazil, Acre	Ministry of Health (Brazil). Brazil Ministry of Health COVID-19 Coronavirus Panel. Rio de Janeiro, Brazil: Ministry of Health (Brazil).

Brazil, Alagoas	Ministry of Health (Brazil). Brazil Ministry of Health COVID-19 Coronavirus Panel. Rio de Janeiro, Brazil: Ministry of Health (Brazil).
Brazil, Amapá	Ministry of Health (Brazil). Brazil Ministry of Health COVID-19 Coronavirus Panel. Rio de Janeiro, Brazil: Ministry of Health (Brazil).
Brazil, Amazonas	Ministry of Health (Brazil). Brazil Ministry of Health COVID-19 Coronavirus Panel. Rio de Janeiro, Brazil: Ministry of Health (Brazil).
Brazil, Bahia	Ministry of Health (Brazil). Brazil Ministry of Health COVID-19 Coronavirus Panel. Rio de Janeiro, Brazil: Ministry of Health (Brazil).
Brazil, Ceará	Ministry of Health (Brazil). Brazil Ministry of Health COVID-19 Coronavirus Panel. Rio de Janeiro, Brazil: Ministry of Health (Brazil).
Brazil, Distrito Federal	Ministry of Health (Brazil). Brazil Ministry of Health COVID-19 Coronavirus Panel. Rio de Janeiro, Brazil: Ministry of Health (Brazil).
Brazil, Espírito Santo	Ministry of Health (Brazil). Brazil Ministry of Health COVID-19 Coronavirus Panel. Rio de Janeiro, Brazil: Ministry of Health (Brazil).
Brazil, Goiás	Ministry of Health (Brazil). Brazil Ministry of Health COVID-19 Coronavirus Panel. Rio de Janeiro, Brazil: Ministry of Health (Brazil).
Brazil, Maranhão	Ministry of Health (Brazil). Brazil Ministry of Health COVID-19 Coronavirus Panel. Rio de Janeiro, Brazil: Ministry of Health (Brazil).
Brazil, Mato Grosso	Ministry of Health (Brazil). Brazil Ministry of Health COVID-19 Coronavirus Panel. Rio de Janeiro, Brazil: Ministry of Health (Brazil).
Brazil, Mato Grosso do Sul	Ministry of Health (Brazil). Brazil Ministry of Health COVID-19 Coronavirus Panel. Rio de Janeiro, Brazil: Ministry of Health (Brazil).
Brazil, Minas Gerais	Ministry of Health (Brazil). Brazil Ministry of Health COVID-19 Coronavirus Panel. Rio de Janeiro, Brazil: Ministry of Health (Brazil).
Brazil, Pará	Ministry of Health (Brazil). Brazil Ministry of Health COVID-19 Coronavirus Panel. Rio de Janeiro, Brazil: Ministry of Health (Brazil).
Brazil, Paraíba	Ministry of Health (Brazil). Brazil Ministry of Health COVID-19 Coronavirus Panel. Rio de Janeiro, Brazil: Ministry of Health (Brazil).
Brazil, Paraná	Ministry of Health (Brazil). Brazil Ministry of Health COVID-19 Coronavirus Panel. Rio de Janeiro, Brazil: Ministry of Health (Brazil).
Brazil, Pernambuco	Ministry of Health (Brazil). Brazil Ministry of Health COVID-19 Coronavirus Panel. Rio de Janeiro, Brazil: Ministry of Health (Brazil).
Brazil, Piauí	Ministry of Health (Brazil). Brazil Ministry of Health COVID-19 Coronavirus Panel. Rio de Janeiro, Brazil: Ministry of Health (Brazil).

Brazil, Rio de Janeiro	Ministry of Health (Brazil). Brazil Ministry of Health COVID-19 Coronavirus Panel. Rio de Janeiro, Brazil: Ministry of Health (Brazil).
Brazil, Rio Grande do Norte	Ministry of Health (Brazil). Brazil Ministry of Health COVID-19 Coronavirus Panel. Rio de Janeiro, Brazil: Ministry of Health (Brazil).
Brazil, Rio Grande do Sul	Ministry of Health (Brazil). Brazil Ministry of Health COVID-19 Coronavirus Panel. Rio de Janeiro, Brazil: Ministry of Health (Brazil).
Brazil, Rondônia	Ministry of Health (Brazil). Brazil Ministry of Health COVID-19 Coronavirus Panel. Rio de Janeiro, Brazil: Ministry of Health (Brazil).
Brazil, Roraima	Ministry of Health (Brazil). Brazil Ministry of Health COVID-19 Coronavirus Panel. Rio de Janeiro, Brazil: Ministry of Health (Brazil).
Brazil, Santa Catarina	Ministry of Health (Brazil). Brazil Ministry of Health COVID-19 Coronavirus Panel. Rio de Janeiro, Brazil: Ministry of Health (Brazil).
Brazil, São Paulo	Ministry of Health (Brazil). Brazil Ministry of Health COVID-19 Coronavirus Panel. Rio de Janeiro, Brazil: Ministry of Health (Brazil).
Brazil, Sergipe	Ministry of Health (Brazil). Brazil Ministry of Health COVID-19 Coronavirus Panel. Rio de Janeiro, Brazil: Ministry of Health (Brazil).
Brazil, Tocantins	Ministry of Health (Brazil). Brazil Ministry of Health COVID-19 Coronavirus Panel. Rio de Janeiro, Brazil: Ministry of Health (Brazil).
Brunei	Johns Hopkins University. 2019 Novel Coronavirus COVID-19 (2019-nCoV) Data Repository by Johns Hopkins CSSE. Baltimore, Maryland: Johns Hopkins University.
Bulgaria	Johns Hopkins University. 2019 Novel Coronavirus COVID-19 (2019-nCoV) Data Repository by Johns Hopkins CSSE. Baltimore, Maryland: Johns Hopkins University.
Burkina Faso	Johns Hopkins University. 2019 Novel Coronavirus COVID-19 (2019-nCoV) Data Repository by Johns Hopkins CSSE. Baltimore, Maryland: Johns Hopkins University.
Burundi	Johns Hopkins University. 2019 Novel Coronavirus COVID-19 (2019-nCoV) Data Repository by Johns Hopkins CSSE. Baltimore, Maryland: Johns Hopkins University.
Cambodia	Johns Hopkins University. 2019 Novel Coronavirus COVID-19 (2019-nCoV) Data Repository by Johns Hopkins CSSE. Baltimore, Maryland: Johns Hopkins University.
Cameroon	Johns Hopkins University. 2019 Novel Coronavirus COVID-19 (2019-nCoV) Data Repository by Johns Hopkins CSSE. Baltimore, Maryland: Johns Hopkins University.
Canada, Alberta	Government of Canada. Canada Public Health Infobase Number of Total Cases of COVID-19. Ottawa, Canada: Government of Canada.
Canada, Alberta	Government of Canada. Canada Coronavirus Disease 2019 (COVID-19) Daily Epidemiology Update. Ottawa, Canada: Government of Canada.

Canada, British Columbia	Government of Canada. Canada Public Health Infobase Number of Total Cases of COVID-19. Ottawa, Canada: Government of Canada.
Canada, British Columbia	Government of Canada. Canada Coronavirus Disease 2019 (COVID-19) Daily Epidemiology Update. Ottawa, Canada: Government of Canada.
Canada, Manitoba	Government of Canada. Canada Public Health Infobase Number of Total Cases of COVID-19. Ottawa, Canada: Government of Canada.
Canada, Manitoba	Government of Canada. Canada Coronavirus Disease 2019 (COVID-19) Daily Epidemiology Update. Ottawa, Canada: Government of Canada.
Canada, New Brunswick	Government of Canada. Canada Public Health Infobase Number of Total Cases of COVID-19. Ottawa, Canada: Government of Canada.
Canada, New Brunswick	Government of Canada. Canada Coronavirus Disease 2019 (COVID-19) Daily Epidemiology Update. Ottawa, Canada: Government of Canada.
Canada, Newfoundland and Labrador	Government of Canada. Canada Public Health Infobase Number of Total Cases of COVID-19. Ottawa, Canada: Government of Canada.
Canada, Newfoundland and Labrador	Government of Canada. Canada Coronavirus Disease 2019 (COVID-19) Daily Epidemiology Update. Ottawa, Canada: Government of Canada.
Canada, Northwest Territories	Government of Canada. Canada Public Health Infobase Number of Total Cases of COVID-19. Ottawa, Canada: Government of Canada.
Canada, Northwest Territories	Government of Canada. Canada Coronavirus Disease 2019 (COVID-19) Daily Epidemiology Update. Ottawa, Canada: Government of Canada.
Canada, Nova Scotia	Government of Canada. Canada Public Health Infobase Number of Total Cases of COVID-19. Ottawa, Canada: Government of Canada.
Canada, Nova Scotia	Government of Canada. Canada Coronavirus Disease 2019 (COVID-19) Daily Epidemiology Update. Ottawa, Canada: Government of Canada.
Canada, Nunavut	Government of Canada. Canada Public Health Infobase Number of Total Cases of COVID-19. Ottawa, Canada: Government of Canada.
Canada, Nunavut	Government of Canada. Canada Coronavirus Disease 2019 (COVID-19) Daily Epidemiology Update. Ottawa, Canada: Government of Canada.
Canada, Ontario	Government of Canada. Canada Public Health Infobase Number of Total Cases of COVID-19. Ottawa, Canada: Government of Canada.
Canada, Ontario	Government of Canada. Canada Coronavirus Disease 2019 (COVID-19) Daily Epidemiology Update. Ottawa, Canada: Government of Canada.
Canada, Prince Edward Island	Government of Canada. Canada Public Health Infobase Number of Total Cases of COVID-19. Ottawa, Canada: Government of Canada.

Canada, Prince Edward Island	Government of Canada. Canada Coronavirus Disease 2019 (COVID-19) Daily Epidemiology Update. Ottawa, Canada: Government of Canada.
Canada, Quebec	Government of Canada. Canada Public Health Infobase Number of Total Cases of COVID-19. Ottawa, Canada: Government of Canada.
Canada, Quebec	Government of Canada. Canada Coronavirus Disease 2019 (COVID-19) Daily Epidemiology Update. Ottawa, Canada: Government of Canada.
Canada, Saskatchewan	Government of Canada. Canada Public Health Infobase Number of Total Cases of COVID-19. Ottawa, Canada: Government of Canada.
Canada, Saskatchewan	Government of Canada. Canada Coronavirus Disease 2019 (COVID-19) Daily Epidemiology Update. Ottawa, Canada: Government of Canada.
Canada, Yukon	Government of Canada. Canada Public Health Infobase Number of Total Cases of COVID-19. Ottawa, Canada: Government of Canada.
Canada, Yukon	Government of Canada. Canada Coronavirus Disease 2019 (COVID-19) Daily Epidemiology Update. Ottawa, Canada: Government of Canada.
Cape Verde	Johns Hopkins University. 2019 Novel Coronavirus COVID-19 (2019-nCoV) Data Repository by Johns Hopkins CSSE. Baltimore, Maryland: Johns Hopkins University.
Central African Republic	Johns Hopkins University. 2019 Novel Coronavirus COVID-19 (2019-nCoV) Data Repository by Johns Hopkins CSSE. Baltimore, Maryland: Johns Hopkins University.
Chad	Johns Hopkins University. 2019 Novel Coronavirus COVID-19 (2019-nCoV) Data Repository by Johns Hopkins CSSE. Baltimore, Maryland: Johns Hopkins University.
Chile	Johns Hopkins University. 2019 Novel Coronavirus COVID-19 (2019-nCoV) Data Repository by Johns Hopkins CSSE. Baltimore, Maryland: Johns Hopkins University.
China, Anhui	Johns Hopkins University. 2019 Novel Coronavirus COVID-19 (2019-nCoV) Data Repository by Johns Hopkins CSSE. Baltimore, Maryland: Johns Hopkins University.
China, Beijing	Johns Hopkins University. 2019 Novel Coronavirus COVID-19 (2019-nCoV) Data Repository by Johns Hopkins CSSE. Baltimore, Maryland: Johns Hopkins University.
China, Chongqing	Johns Hopkins University. 2019 Novel Coronavirus COVID-19 (2019-nCoV) Data Repository by Johns Hopkins CSSE. Baltimore, Maryland: Johns Hopkins University.
China, Fujian	Johns Hopkins University. 2019 Novel Coronavirus COVID-19 (2019-nCoV) Data Repository by Johns Hopkins CSSE. Baltimore, Maryland: Johns Hopkins University.
China, Gansu	Johns Hopkins University. 2019 Novel Coronavirus COVID-19 (2019-nCoV) Data Repository by Johns Hopkins CSSE. Baltimore, Maryland: Johns Hopkins University.
China, Guangdong	Johns Hopkins University. 2019 Novel Coronavirus COVID-19 (2019-nCoV) Data Repository by Johns Hopkins CSSE. Baltimore, Maryland: Johns Hopkins University.

China, Guangxi	Johns Hopkins University. 2019 Novel Coronavirus COVID-19 (2019-nCoV) Data Repository by Johns Hopkins CSSE. Baltimore, Maryland: Johns Hopkins University.
China, Guizhou	Johns Hopkins University. 2019 Novel Coronavirus COVID-19 (2019-nCoV) Data Repository by Johns Hopkins CSSE. Baltimore, Maryland: Johns Hopkins University.
China, Hainan	Johns Hopkins University. 2019 Novel Coronavirus COVID-19 (2019-nCoV) Data Repository by Johns Hopkins CSSE. Baltimore, Maryland: Johns Hopkins University.
China, Hebei	Johns Hopkins University. 2019 Novel Coronavirus COVID-19 (2019-nCoV) Data Repository by Johns Hopkins CSSE. Baltimore, Maryland: Johns Hopkins University.
China, Heilongjiang	Johns Hopkins University. 2019 Novel Coronavirus COVID-19 (2019-nCoV) Data Repository by Johns Hopkins CSSE. Baltimore, Maryland: Johns Hopkins University.
China, Henan	Johns Hopkins University. 2019 Novel Coronavirus COVID-19 (2019-nCoV) Data Repository by Johns Hopkins CSSE. Baltimore, Maryland: Johns Hopkins University.
China, Hong Kong Special Administrative Region of China	Johns Hopkins University. 2019 Novel Coronavirus COVID-19 (2019-nCoV) Data Repository by Johns Hopkins CSSE. Baltimore, Maryland: Johns Hopkins University.
China, Hubei	Johns Hopkins University. 2019 Novel Coronavirus COVID-19 (2019-nCoV) Data Repository by Johns Hopkins CSSE. Baltimore, Maryland: Johns Hopkins University.
China, Hunan	Johns Hopkins University. 2019 Novel Coronavirus COVID-19 (2019-nCoV) Data Repository by Johns Hopkins CSSE. Baltimore, Maryland: Johns Hopkins University.
China, Inner Mongolia	Johns Hopkins University. 2019 Novel Coronavirus COVID-19 (2019-nCoV) Data Repository by Johns Hopkins CSSE. Baltimore, Maryland: Johns Hopkins University.
China, Jiangsu	Johns Hopkins University. 2019 Novel Coronavirus COVID-19 (2019-nCoV) Data Repository by Johns Hopkins CSSE. Baltimore, Maryland: Johns Hopkins University.
China, Jiangxi	Johns Hopkins University. 2019 Novel Coronavirus COVID-19 (2019-nCoV) Data Repository by Johns Hopkins CSSE. Baltimore, Maryland: Johns Hopkins University.
China, Jilin	Johns Hopkins University. 2019 Novel Coronavirus COVID-19 (2019-nCoV) Data Repository by Johns Hopkins CSSE. Baltimore, Maryland: Johns Hopkins University.
China, Liaoning	Johns Hopkins University. 2019 Novel Coronavirus COVID-19 (2019-nCoV) Data Repository by Johns Hopkins CSSE. Baltimore, Maryland: Johns Hopkins University.
China, Macao Special Administrative Region of China	Johns Hopkins University. 2019 Novel Coronavirus COVID-19 (2019-nCoV) Data Repository by Johns Hopkins CSSE. Baltimore, Maryland: Johns Hopkins University.
China, Ningxia	Johns Hopkins University. 2019 Novel Coronavirus COVID-19 (2019-nCoV) Data Repository by Johns Hopkins CSSE. Baltimore, Maryland: Johns Hopkins University.
China, Qinghai	Johns Hopkins University. 2019 Novel Coronavirus COVID-19 (2019-nCoV) Data Repository by Johns Hopkins CSSE. Baltimore, Maryland: Johns Hopkins University.

China, Shaanxi	Johns Hopkins University. 2019 Novel Coronavirus COVID-19 (2019-nCoV) Data Repository by Johns Hopkins CSSE. Baltimore, Maryland: Johns Hopkins University.
China, Shandong	Johns Hopkins University. 2019 Novel Coronavirus COVID-19 (2019-nCoV) Data Repository by Johns Hopkins CSSE. Baltimore, Maryland: Johns Hopkins University.
China, Shanghai	Johns Hopkins University. 2019 Novel Coronavirus COVID-19 (2019-nCoV) Data Repository by Johns Hopkins CSSE. Baltimore, Maryland: Johns Hopkins University.
China, Shanxi	Johns Hopkins University. 2019 Novel Coronavirus COVID-19 (2019-nCoV) Data Repository by Johns Hopkins CSSE. Baltimore, Maryland: Johns Hopkins University.
China, Sichuan	Johns Hopkins University. 2019 Novel Coronavirus COVID-19 (2019-nCoV) Data Repository by Johns Hopkins CSSE. Baltimore, Maryland: Johns Hopkins University.
China, Tianjin	Johns Hopkins University. 2019 Novel Coronavirus COVID-19 (2019-nCoV) Data Repository by Johns Hopkins CSSE. Baltimore, Maryland: Johns Hopkins University.
China, Tibet	Johns Hopkins University. 2019 Novel Coronavirus COVID-19 (2019-nCoV) Data Repository by Johns Hopkins CSSE. Baltimore, Maryland: Johns Hopkins University.
China, Xinjiang	Johns Hopkins University. 2019 Novel Coronavirus COVID-19 (2019-nCoV) Data Repository by Johns Hopkins CSSE. Baltimore, Maryland: Johns Hopkins University.
China, Yunnan	Johns Hopkins University. 2019 Novel Coronavirus COVID-19 (2019-nCoV) Data Repository by Johns Hopkins CSSE. Baltimore, Maryland: Johns Hopkins University.
China, Zhejiang	Johns Hopkins University. 2019 Novel Coronavirus COVID-19 (2019-nCoV) Data Repository by Johns Hopkins CSSE. Baltimore, Maryland: Johns Hopkins University.
Colombia	Johns Hopkins University. 2019 Novel Coronavirus COVID-19 (2019-nCoV) Data Repository by Johns Hopkins CSSE. Baltimore, Maryland: Johns Hopkins University.
Comoros	Johns Hopkins University. 2019 Novel Coronavirus COVID-19 (2019-nCoV) Data Repository by Johns Hopkins CSSE. Baltimore, Maryland: Johns Hopkins University.
Congo (Brazzaville)	Johns Hopkins University. 2019 Novel Coronavirus COVID-19 (2019-nCoV) Data Repository by Johns Hopkins CSSE. Baltimore, Maryland: Johns Hopkins University.
Costa Rica	Johns Hopkins University. 2019 Novel Coronavirus COVID-19 (2019-nCoV) Data Repository by Johns Hopkins CSSE. Baltimore, Maryland: Johns Hopkins University.
Côte d'Ivoire	Johns Hopkins University. 2019 Novel Coronavirus COVID-19 (2019-nCoV) Data Repository by Johns Hopkins CSSE. Baltimore, Maryland: Johns Hopkins University.
Croatia	Johns Hopkins University. 2019 Novel Coronavirus COVID-19 (2019-nCoV) Data Repository by Johns Hopkins CSSE. Baltimore, Maryland: Johns Hopkins University.
Cuba	Johns Hopkins University. 2019 Novel Coronavirus COVID-19 (2019-nCoV) Data Repository by Johns Hopkins CSSE. Baltimore, Maryland: Johns Hopkins University.

Cyprus	Johns Hopkins University. 2019 Novel Coronavirus COVID-19 (2019-nCoV) Data Repository by Johns Hopkins CSSE. Baltimore, Maryland: Johns Hopkins University.
Czechia	Johns Hopkins University. 2019 Novel Coronavirus COVID-19 (2019-nCoV) Data Repository by Johns Hopkins CSSE. Baltimore, Maryland: Johns Hopkins University.
Denmark	Johns Hopkins University. 2019 Novel Coronavirus COVID-19 (2019-nCoV) Data Repository by Johns Hopkins CSSE. Baltimore, Maryland: Johns Hopkins University.
Djibouti	Johns Hopkins University. 2019 Novel Coronavirus COVID-19 (2019-nCoV) Data Repository by Johns Hopkins CSSE. Baltimore, Maryland: Johns Hopkins University.
Dominica	Johns Hopkins University. 2019 Novel Coronavirus COVID-19 (2019-nCoV) Data Repository by Johns Hopkins CSSE. Baltimore, Maryland: Johns Hopkins University.
Dominican Republic	Johns Hopkins University. 2019 Novel Coronavirus COVID-19 (2019-nCoV) Data Repository by Johns Hopkins CSSE. Baltimore, Maryland: Johns Hopkins University.
DR Congo	Johns Hopkins University. 2019 Novel Coronavirus COVID-19 (2019-nCoV) Data Repository by Johns Hopkins CSSE. Baltimore, Maryland: Johns Hopkins University.
Ecuador	Johns Hopkins University. 2019 Novel Coronavirus COVID-19 (2019-nCoV) Data Repository by Johns Hopkins CSSE. Baltimore, Maryland: Johns Hopkins University.
Egypt	Johns Hopkins University. 2019 Novel Coronavirus COVID-19 (2019-nCoV) Data Repository by Johns Hopkins CSSE. Baltimore, Maryland: Johns Hopkins University.
El Salvador	Johns Hopkins University. 2019 Novel Coronavirus COVID-19 (2019-nCoV) Data Repository by Johns Hopkins CSSE. Baltimore, Maryland: Johns Hopkins University.
Equatorial Guinea	Johns Hopkins University. 2019 Novel Coronavirus COVID-19 (2019-nCoV) Data Repository by Johns Hopkins CSSE. Baltimore, Maryland: Johns Hopkins University.
Eritrea	Johns Hopkins University. 2019 Novel Coronavirus COVID-19 (2019-nCoV) Data Repository by Johns Hopkins CSSE. Baltimore, Maryland: Johns Hopkins University.
Estonia	Johns Hopkins University. 2019 Novel Coronavirus COVID-19 (2019-nCoV) Data Repository by Johns Hopkins CSSE. Baltimore, Maryland: Johns Hopkins University.
Eswatini	Johns Hopkins University. 2019 Novel Coronavirus COVID-19 (2019-nCoV) Data Repository by Johns Hopkins CSSE. Baltimore, Maryland: Johns Hopkins University.
Ethiopia	Johns Hopkins University. 2019 Novel Coronavirus COVID-19 (2019-nCoV) Data Repository by Johns Hopkins CSSE. Baltimore, Maryland: Johns Hopkins University.
Federated States of Micronesia	Johns Hopkins University. 2019 Novel Coronavirus COVID-19 (2019-nCoV) Data Repository by Johns Hopkins CSSE. Baltimore, Maryland: Johns Hopkins University.
Fiji	Johns Hopkins University. 2019 Novel Coronavirus COVID-19 (2019-nCoV) Data Repository by Johns Hopkins CSSE. Baltimore, Maryland: Johns Hopkins University.

Finland	Johns Hopkins University. 2019 Novel Coronavirus COVID-19 (2019-nCoV) Data Repository by Johns Hopkins CSSE. Baltimore, Maryland: Johns Hopkins University.
France	Etalab (France), Ministry of Health (France), Ministry of Health and Solidarity (DRESS) (France). France COVID-19 Epidemic Monitoring Dashboard.
Gabon	Johns Hopkins University. 2019 Novel Coronavirus COVID-19 (2019-nCoV) Data Repository by Johns Hopkins CSSE. Baltimore, Maryland: Johns Hopkins University.
Georgia	Johns Hopkins University. 2019 Novel Coronavirus COVID-19 (2019-nCoV) Data Repository by Johns Hopkins CSSE. Baltimore, Maryland: Johns Hopkins University.
Germany, Baden-Württemberg	Robert Koch Institute. Coronavirus Disease 2019 (COVID-19) Daily Situation Report - Robert Koch Institute. Berlin, Germany: Robert Koch Institute.
Germany, Baden-Württemberg	Wikipedia. COVID-19 pandemic in Germany. San Francisco, United States of America: Wikipedia.
Germany, Bavaria	Robert Koch Institute. Coronavirus Disease 2019 (COVID-19) Daily Situation Report - Robert Koch Institute. Berlin, Germany: Robert Koch Institute.
Germany, Bavaria	Wikipedia. COVID-19 pandemic in Germany. San Francisco, United States of America: Wikipedia.
Germany, Berlin	Robert Koch Institute. Coronavirus Disease 2019 (COVID-19) Daily Situation Report - Robert Koch Institute. Berlin, Germany: Robert Koch Institute.
Germany, Berlin	Wikipedia. COVID-19 pandemic in Germany. San Francisco, United States of America: Wikipedia.
Germany, Brandenburg	Robert Koch Institute. Coronavirus Disease 2019 (COVID-19) Daily Situation Report - Robert Koch Institute. Berlin, Germany: Robert Koch Institute.
Germany, Brandenburg	Wikipedia. COVID-19 pandemic in Germany. San Francisco, United States of America: Wikipedia.
Germany, Bremen	Robert Koch Institute. Coronavirus Disease 2019 (COVID-19) Daily Situation Report - Robert Koch Institute. Berlin, Germany: Robert Koch Institute.
Germany, Bremen	Wikipedia. COVID-19 pandemic in Germany. San Francisco, United States of America: Wikipedia.
Germany, Hamburg	Robert Koch Institute. Coronavirus Disease 2019 (COVID-19) Daily Situation Report - Robert Koch Institute. Berlin, Germany: Robert Koch Institute.
Germany, Hamburg	Wikipedia. COVID-19 pandemic in Germany. San Francisco, United States of America: Wikipedia.
Germany, Hesse	Robert Koch Institute. Coronavirus Disease 2019 (COVID-19) Daily Situation Report - Robert Koch Institute. Berlin, Germany: Robert Koch Institute.
Germany, Hesse	Wikipedia. COVID-19 pandemic in Germany. San Francisco, United States of America: Wikipedia.

Germany, Lower Saxony	Robert Koch Institute. Coronavirus Disease 2019 (COVID-19) Daily Situation Report - Robert Koch Institute. Berlin, Germany: Robert Koch Institute.
Germany, Lower Saxony	Wikipedia. COVID-19 pandemic in Germany. San Francisco, United States of America: Wikipedia.
Germany, Mecklenburg-Vorpommern	Robert Koch Institute. Coronavirus Disease 2019 (COVID-19) Daily Situation Report - Robert Koch Institute. Berlin, Germany: Robert Koch Institute.
Germany, Mecklenburg-Vorpommern	Wikipedia. COVID-19 pandemic in Germany. San Francisco, United States of America: Wikipedia.
Germany, North Rhine-Westphalia	Robert Koch Institute. Coronavirus Disease 2019 (COVID-19) Daily Situation Report - Robert Koch Institute. Berlin, Germany: Robert Koch Institute.
Germany, North Rhine-Westphalia	Wikipedia. COVID-19 pandemic in Germany. San Francisco, United States of America: Wikipedia.
Germany, Rhineland-Palatinate	Robert Koch Institute. Coronavirus Disease 2019 (COVID-19) Daily Situation Report - Robert Koch Institute. Berlin, Germany: Robert Koch Institute.
Germany, Rhineland-Palatinate	Wikipedia. COVID-19 pandemic in Germany. San Francisco, United States of America: Wikipedia.
Germany, Saarland	Robert Koch Institute. Coronavirus Disease 2019 (COVID-19) Daily Situation Report - Robert Koch Institute. Berlin, Germany: Robert Koch Institute.
Germany, Saarland	Wikipedia. COVID-19 pandemic in Germany. San Francisco, United States of America: Wikipedia.
Germany, Saxony	Robert Koch Institute. Coronavirus Disease 2019 (COVID-19) Daily Situation Report - Robert Koch Institute. Berlin, Germany: Robert Koch Institute.
Germany, Saxony	Wikipedia. COVID-19 pandemic in Germany. San Francisco, United States of America: Wikipedia.
Germany, Saxony-Anhalt	Robert Koch Institute. Coronavirus Disease 2019 (COVID-19) Daily Situation Report - Robert Koch Institute. Berlin, Germany: Robert Koch Institute.
Germany, Saxony-Anhalt	Wikipedia. COVID-19 pandemic in Germany. San Francisco, United States of America: Wikipedia.
Germany, Schleswig-Holstein	Robert Koch Institute. Coronavirus Disease 2019 (COVID-19) Daily Situation Report - Robert Koch Institute. Berlin, Germany: Robert Koch Institute.
Germany, Schleswig-Holstein	Wikipedia. COVID-19 pandemic in Germany. San Francisco, United States of America: Wikipedia.
Germany, Thuringia	Robert Koch Institute. Coronavirus Disease 2019 (COVID-19) Daily Situation Report - Robert Koch Institute. Berlin, Germany: Robert Koch Institute.
Germany, Thuringia	Wikipedia. COVID-19 pandemic in Germany. San Francisco, United States of America: Wikipedia.

Ghana	Johns Hopkins University. 2019 Novel Coronavirus COVID-19 (2019-nCoV) Data Repository by Johns Hopkins CSSE. Baltimore, Maryland: Johns Hopkins University.
Greece	Johns Hopkins University. 2019 Novel Coronavirus COVID-19 (2019-nCoV) Data Repository by Johns Hopkins CSSE. Baltimore, Maryland: Johns Hopkins University.
Greenland	Johns Hopkins University. 2019 Novel Coronavirus COVID-19 (2019-nCoV) Data Repository by Johns Hopkins CSSE. Baltimore, Maryland: Johns Hopkins University.
Grenada	Johns Hopkins University. 2019 Novel Coronavirus COVID-19 (2019-nCoV) Data Repository by Johns Hopkins CSSE. Baltimore, Maryland: Johns Hopkins University.
Guam	Johns Hopkins University. 2019 Novel Coronavirus COVID-19 (2019-nCoV) Data Repository by Johns Hopkins CSSE. Baltimore, Maryland: Johns Hopkins University.
Guatemala	Ministerio de Salud Publica y Asistencia Social. Situacion de COVID-19 en Guatemala. Gobierno de Guatemala
Guinea	Johns Hopkins University. 2019 Novel Coronavirus COVID-19 (2019-nCoV) Data Repository by Johns Hopkins CSSE. Baltimore, Maryland: Johns Hopkins University.
Guinea-Bissau	Johns Hopkins University. 2019 Novel Coronavirus COVID-19 (2019-nCoV) Data Repository by Johns Hopkins CSSE. Baltimore, Maryland: Johns Hopkins University.
Guyana	Johns Hopkins University. 2019 Novel Coronavirus COVID-19 (2019-nCoV) Data Repository by Johns Hopkins CSSE. Baltimore, Maryland: Johns Hopkins University.
Haiti	Johns Hopkins University. 2019 Novel Coronavirus COVID-19 (2019-nCoV) Data Repository by Johns Hopkins CSSE. Baltimore, Maryland: Johns Hopkins University.
Honduras	Johns Hopkins University. 2019 Novel Coronavirus COVID-19 (2019-nCoV) Data Repository by Johns Hopkins CSSE. Baltimore, Maryland: Johns Hopkins University.
Hungary	Johns Hopkins University. 2019 Novel Coronavirus COVID-19 (2019-nCoV) Data Repository by Johns Hopkins CSSE. Baltimore, Maryland: Johns Hopkins University.
Iceland	Johns Hopkins University. 2019 Novel Coronavirus COVID-19 (2019-nCoV) Data Repository by Johns Hopkins CSSE. Baltimore, Maryland: Johns Hopkins University.
India, Andhra Pradesh	COVID-19 India. India COVID-19 Crowdsourced Patient Database: National Level Time Series, State-Wise Stats and Test Counts. India: COVID-19 India.
India, Andhra Pradesh	COVID-19 India. India COVID-19 Crowdsourced Patient Database: State Level Daily Changes. India: COVID-19 India.
India, Arunachal Pradesh	COVID-19 India. India COVID-19 Crowdsourced Patient Database: National Level Time Series, State-Wise Stats and Test Counts. India: COVID-19 India.
India, Arunachal Pradesh	COVID-19 India. India COVID-19 Crowdsourced Patient Database: State Level Daily Changes. India: COVID-19 India.

India, Assam	COVID-19 India. India COVID-19 Crowdsourced Patient Database: National Level Time Series, State-Wise Stats and Test Counts. India: COVID-19 India.
India, Assam	COVID-19 India. India COVID-19 Crowdsourced Patient Database: State Level Daily Changes. India: COVID-19 India.
India, Bihar	COVID-19 India. India COVID-19 Crowdsourced Patient Database: National Level Time Series, State-Wise Stats and Test Counts. India: COVID-19 India.
India, Bihar	COVID-19 India. India COVID-19 Crowdsourced Patient Database: State Level Daily Changes. India: COVID-19 India.
India, Chhattisgarh	COVID-19 India. India COVID-19 Crowdsourced Patient Database: National Level Time Series, State-Wise Stats and Test Counts. India: COVID-19 India.
India, Chhattisgarh	COVID-19 India. India COVID-19 Crowdsourced Patient Database: State Level Daily Changes. India: COVID-19 India.
India, Dadra and Nagar Haveli and Daman and Diu	COVID-19 India. India COVID-19 Crowdsourced Patient Database: National Level Time Series, State-Wise Stats and Test Counts. India: COVID-19 India.
India, Dadra and Nagar Haveli and Daman and Diu	COVID-19 India. India COVID-19 Crowdsourced Patient Database: State Level Daily Changes. India: COVID-19 India.
India, Delhi	COVID-19 India. India COVID-19 Crowdsourced Patient Database: National Level Time Series, State-Wise Stats and Test Counts. India: COVID-19 India.
India, Delhi	COVID-19 India. India COVID-19 Crowdsourced Patient Database: State Level Daily Changes. India: COVID-19 India.
India, Goa	COVID-19 India. India COVID-19 Crowdsourced Patient Database: National Level Time Series, State-Wise Stats and Test Counts. India: COVID-19 India.
India, Goa	COVID-19 India. India COVID-19 Crowdsourced Patient Database: State Level Daily Changes. India: COVID-19 India.
India, Gujarat	COVID-19 India. India COVID-19 Crowdsourced Patient Database: National Level Time Series, State-Wise Stats and Test Counts. India: COVID-19 India.
India, Gujarat	COVID-19 India. India COVID-19 Crowdsourced Patient Database: State Level Daily Changes. India: COVID-19 India.
India, Haryana	COVID-19 India. India COVID-19 Crowdsourced Patient Database: National Level Time Series, State-Wise Stats and Test Counts. India: COVID-19 India.
India, Haryana	COVID-19 India. India COVID-19 Crowdsourced Patient Database: State Level Daily Changes. India: COVID-19 India.
India, Himachal Pradesh	COVID-19 India. India COVID-19 Crowdsourced Patient Database: National Level Time Series, State-Wise Stats and Test Counts. India: COVID-19 India.

India, Himachal Pradesh	COVID-19 India. India COVID-19 Crowdsourced Patient Database: State Level Daily Changes. India: COVID-19 India.
India, Jammu & Kashmir and Ladakh	COVID-19 India. India COVID-19 Crowdsourced Patient Database: National Level Time Series, State-Wise Stats and Test Counts. India: COVID-19 India.
India, Jammu & Kashmir and Ladakh	COVID-19 India. India COVID-19 Crowdsourced Patient Database: State Level Daily Changes. India: COVID-19 India.
India, Jharkhand	COVID-19 India. India COVID-19 Crowdsourced Patient Database: National Level Time Series, State-Wise Stats and Test Counts. India: COVID-19 India.
India, Jharkhand	COVID-19 India. India COVID-19 Crowdsourced Patient Database: State Level Daily Changes. India: COVID-19 India.
India, Karnataka	COVID-19 India. India COVID-19 Crowdsourced Patient Database: National Level Time Series, State-Wise Stats and Test Counts. India: COVID-19 India.
India, Karnataka	COVID-19 India. India COVID-19 Crowdsourced Patient Database: State Level Daily Changes. India: COVID-19 India.
India, Kerala	COVID-19 India. India COVID-19 Crowdsourced Patient Database: National Level Time Series, State-Wise Stats and Test Counts. India: COVID-19 India.
India, Kerala	COVID-19 India. India COVID-19 Crowdsourced Patient Database: State Level Daily Changes. India: COVID-19 India.
India, Madhya Pradesh	COVID-19 India. India COVID-19 Crowdsourced Patient Database: National Level Time Series, State-Wise Stats and Test Counts. India: COVID-19 India.
India, Madhya Pradesh	COVID-19 India. India COVID-19 Crowdsourced Patient Database: State Level Daily Changes. India: COVID-19 India.
India, Maharashtra	COVID-19 India. India COVID-19 Crowdsourced Patient Database: National Level Time Series, State-Wise Stats and Test Counts. India: COVID-19 India.
India, Maharashtra	COVID-19 India. India COVID-19 Crowdsourced Patient Database: State Level Daily Changes. India: COVID-19 India.
India, Manipur	COVID-19 India. India COVID-19 Crowdsourced Patient Database: National Level Time Series, State-Wise Stats and Test Counts. India: COVID-19 India.
India, Manipur	COVID-19 India. India COVID-19 Crowdsourced Patient Database: State Level Daily Changes. India: COVID-19 India.
India, Meghalaya	COVID-19 India. India COVID-19 Crowdsourced Patient Database: National Level Time Series, State-Wise Stats and Test Counts. India: COVID-19 India.
India, Meghalaya	COVID-19 India. India COVID-19 Crowdsourced Patient Database: State Level Daily Changes. India: COVID-19 India.

India, Mizoram	COVID-19 India. India COVID-19 Crowdsourced Patient Database: National Level Time Series, State-Wise Stats and Test Counts. India: COVID-19 India.
India, Mizoram	COVID-19 India. India COVID-19 Crowdsourced Patient Database: State Level Daily Changes. India: COVID-19 India.
India, Nagaland	COVID-19 India. India COVID-19 Crowdsourced Patient Database: National Level Time Series, State-Wise Stats and Test Counts. India: COVID-19 India.
India, Nagaland	COVID-19 India. India COVID-19 Crowdsourced Patient Database: State Level Daily Changes. India: COVID-19 India.
India, Odisha	COVID-19 India. India COVID-19 Crowdsourced Patient Database: National Level Time Series, State-Wise Stats and Test Counts. India: COVID-19 India.
India, Odisha	COVID-19 India. India COVID-19 Crowdsourced Patient Database: State Level Daily Changes. India: COVID-19 India.
India, Punjab	COVID-19 India. India COVID-19 Crowdsourced Patient Database: National Level Time Series, State-Wise Stats and Test Counts. India: COVID-19 India.
India, Punjab	COVID-19 India. India COVID-19 Crowdsourced Patient Database: State Level Daily Changes. India: COVID-19 India.
India, Rajasthan	COVID-19 India. India COVID-19 Crowdsourced Patient Database: National Level Time Series, State-Wise Stats and Test Counts. India: COVID-19 India.
India, Rajasthan	COVID-19 India. India COVID-19 Crowdsourced Patient Database: State Level Daily Changes. India: COVID-19 India.
India, Sikkim	COVID-19 India. India COVID-19 Crowdsourced Patient Database: National Level Time Series, State-Wise Stats and Test Counts. India: COVID-19 India.
India, Sikkim	COVID-19 India. India COVID-19 Crowdsourced Patient Database: State Level Daily Changes. India: COVID-19 India.
India, Tamil Nadu	COVID-19 India. India COVID-19 Crowdsourced Patient Database: National Level Time Series, State-Wise Stats and Test Counts. India: COVID-19 India.
India, Tamil Nadu	COVID-19 India. India COVID-19 Crowdsourced Patient Database: State Level Daily Changes. India: COVID-19 India.
India, Telangana	COVID-19 India. India COVID-19 Crowdsourced Patient Database: National Level Time Series, State-Wise Stats and Test Counts. India: COVID-19 India.
India, Telangana	COVID-19 India. India COVID-19 Crowdsourced Patient Database: State Level Daily Changes. India: COVID-19 India.
India, Tripura	COVID-19 India. India COVID-19 Crowdsourced Patient Database: National Level Time Series, State-Wise Stats and Test Counts. India: COVID-19 India.

India, Tripura	COVID-19 India. India COVID-19 Crowdsourced Patient Database: State Level Daily Changes. India: COVID-19 India.
India, Uttar Pradesh	COVID-19 India. India COVID-19 Crowdsourced Patient Database: National Level Time Series, State-Wise Stats and Test Counts. India: COVID-19 India.
India, Uttar Pradesh	COVID-19 India. India COVID-19 Crowdsourced Patient Database: State Level Daily Changes. India: COVID-19 India.
India, Uttarakhand	COVID-19 India. India COVID-19 Crowdsourced Patient Database: National Level Time Series, State-Wise Stats and Test Counts. India: COVID-19 India.
India, Uttarakhand	COVID-19 India. India COVID-19 Crowdsourced Patient Database: State Level Daily Changes. India: COVID-19 India.
India, West Bengal	COVID-19 India. India COVID-19 Crowdsourced Patient Database: National Level Time Series, State-Wise Stats and Test Counts. India: COVID-19 India.
India, West Bengal	COVID-19 India. India COVID-19 Crowdsourced Patient Database: State Level Daily Changes. India: COVID-19 India.
Indonesia	Johns Hopkins University. 2019 Novel Coronavirus COVID-19 (2019-nCoV) Data Repository by Johns Hopkins CSSE. Baltimore, Maryland: Johns Hopkins University.
Iran	Johns Hopkins University. 2019 Novel Coronavirus COVID-19 (2019-nCoV) Data Repository by Johns Hopkins CSSE. Baltimore, Maryland: Johns Hopkins University.
Iraq	Johns Hopkins University. 2019 Novel Coronavirus COVID-19 (2019-nCoV) Data Repository by Johns Hopkins CSSE. Baltimore, Maryland: Johns Hopkins University.
Ireland	Johns Hopkins University. 2019 Novel Coronavirus COVID-19 (2019-nCoV) Data Repository by Johns Hopkins CSSE. Baltimore, Maryland: Johns Hopkins University.
Israel	Johns Hopkins University. 2019 Novel Coronavirus COVID-19 (2019-nCoV) Data Repository by Johns Hopkins CSSE. Baltimore, Maryland: Johns Hopkins University.
Italy, Abruzzo	Department of Civil Protection (Italy). Italy COVID-19 Situation Monitoring - Department of Civil Protection. Rome, Italy: Department of Civil Protection (Italy).
Italy, Basilicata	Department of Civil Protection (Italy). Italy COVID-19 Situation Monitoring - Department of Civil Protection. Rome, Italy: Department of Civil Protection (Italy).
Italy, Calabria	Department of Civil Protection (Italy). Italy COVID-19 Situation Monitoring - Department of Civil Protection. Rome, Italy: Department of Civil Protection (Italy).
Italy, Campania	Department of Civil Protection (Italy). Italy COVID-19 Situation Monitoring - Department of Civil Protection. Rome, Italy: Department of Civil Protection (Italy).
Italy, Emilia-Romagna	Department of Civil Protection (Italy). Italy COVID-19 Situation Monitoring - Department of Civil Protection. Rome, Italy: Department of Civil Protection (Italy).

Italy, Friuli-Venezia Giulia	Department of Civil Protection (Italy). Italy COVID-19 Situation Monitoring - Department of Civil Protection. Rome, Italy: Department of Civil Protection (Italy).
Italy, Lazio	Department of Civil Protection (Italy). Italy COVID-19 Situation Monitoring - Department of Civil Protection. Rome, Italy: Department of Civil Protection (Italy).
Italy, Liguria	Department of Civil Protection (Italy). Italy COVID-19 Situation Monitoring - Department of Civil Protection. Rome, Italy: Department of Civil Protection (Italy).
Italy, Lombardia	Department of Civil Protection (Italy). Italy COVID-19 Situation Monitoring - Department of Civil Protection. Rome, Italy: Department of Civil Protection (Italy).
Italy, Marche	Department of Civil Protection (Italy). Italy COVID-19 Situation Monitoring - Department of Civil Protection. Rome, Italy: Department of Civil Protection (Italy).
Italy, Molise	Department of Civil Protection (Italy). Italy COVID-19 Situation Monitoring - Department of Civil Protection. Rome, Italy: Department of Civil Protection (Italy).
Italy, Piemonte	Department of Civil Protection (Italy). Italy COVID-19 Situation Monitoring - Department of Civil Protection. Rome, Italy: Department of Civil Protection (Italy).
Italy, Prov. autonoma di Bolzano	Department of Civil Protection (Italy). Italy COVID-19 Situation Monitoring - Department of Civil Protection. Rome, Italy: Department of Civil Protection (Italy).
Italy, Prov. autonoma di Trento	Department of Civil Protection (Italy). Italy COVID-19 Situation Monitoring - Department of Civil Protection. Rome, Italy: Department of Civil Protection (Italy).
Italy, Puglia	Department of Civil Protection (Italy). Italy COVID-19 Situation Monitoring - Department of Civil Protection. Rome, Italy: Department of Civil Protection (Italy).
Italy, Sardegna	Department of Civil Protection (Italy). Italy COVID-19 Situation Monitoring - Department of Civil Protection. Rome, Italy: Department of Civil Protection (Italy).
Italy, Sicilia	Department of Civil Protection (Italy). Italy COVID-19 Situation Monitoring - Department of Civil Protection. Rome, Italy: Department of Civil Protection (Italy).
Italy, Toscana	Department of Civil Protection (Italy). Italy COVID-19 Situation Monitoring - Department of Civil Protection. Rome, Italy: Department of Civil Protection (Italy).
Italy, Umbria	Department of Civil Protection (Italy). Italy COVID-19 Situation Monitoring - Department of Civil Protection. Rome, Italy: Department of Civil Protection (Italy).
Italy, Valle d'Aosta	Department of Civil Protection (Italy). Italy COVID-19 Situation Monitoring - Department of Civil Protection. Rome, Italy: Department of Civil Protection (Italy).
Italy, Veneto	Department of Civil Protection (Italy). Italy COVID-19 Situation Monitoring - Department of Civil Protection. Rome, Italy: Department of Civil Protection (Italy).
Jamaica	Johns Hopkins University. 2019 Novel Coronavirus COVID-19 (2019-nCoV) Data Repository by Johns Hopkins CSSE. Baltimore, Maryland: Johns Hopkins University.

Japan	Ministry of Health, Labour and Welfare (Japan). Japan Coronavirus Disease (COVID-19) Situation Report.
Jordan	Johns Hopkins University. 2019 Novel Coronavirus COVID-19 (2019-nCoV) Data Repository by Johns Hopkins CSSE. Baltimore, Maryland: Johns Hopkins University.
Kazakhstan	*
Kenya	Johns Hopkins University. 2019 Novel Coronavirus COVID-19 (2019-nCoV) Data Repository by Johns Hopkins CSSE. Baltimore, Maryland: Johns Hopkins University.
Kiribati	Johns Hopkins University. 2019 Novel Coronavirus COVID-19 (2019-nCoV) Data Repository by Johns Hopkins CSSE. Baltimore, Maryland: Johns Hopkins University.
Kuwait	Johns Hopkins University. 2019 Novel Coronavirus COVID-19 (2019-nCoV) Data Repository by Johns Hopkins CSSE. Baltimore, Maryland: Johns Hopkins University.
Kyrgyzstan	Johns Hopkins University. 2019 Novel Coronavirus COVID-19 (2019-nCoV) Data Repository by Johns Hopkins CSSE. Baltimore, Maryland: Johns Hopkins University.
Laos	Johns Hopkins University. 2019 Novel Coronavirus COVID-19 (2019-nCoV) Data Repository by Johns Hopkins CSSE. Baltimore, Maryland: Johns Hopkins University.
Latvia	Johns Hopkins University. 2019 Novel Coronavirus COVID-19 (2019-nCoV) Data Repository by Johns Hopkins CSSE. Baltimore, Maryland: Johns Hopkins University.
Lebanon	Johns Hopkins University. 2019 Novel Coronavirus COVID-19 (2019-nCoV) Data Repository by Johns Hopkins CSSE. Baltimore, Maryland: Johns Hopkins University.
Lesotho	Johns Hopkins University. 2019 Novel Coronavirus COVID-19 (2019-nCoV) Data Repository by Johns Hopkins CSSE. Baltimore, Maryland: Johns Hopkins University.
Liberia	Johns Hopkins University. 2019 Novel Coronavirus COVID-19 (2019-nCoV) Data Repository by Johns Hopkins CSSE. Baltimore, Maryland: Johns Hopkins University.
Libya	Johns Hopkins University. 2019 Novel Coronavirus COVID-19 (2019-nCoV) Data Repository by Johns Hopkins CSSE. Baltimore, Maryland: Johns Hopkins University.
Lithuania	Johns Hopkins University. 2019 Novel Coronavirus COVID-19 (2019-nCoV) Data Repository by Johns Hopkins CSSE. Baltimore, Maryland: Johns Hopkins University.
Luxembourg	Johns Hopkins University. 2019 Novel Coronavirus COVID-19 (2019-nCoV) Data Repository by Johns Hopkins CSSE. Baltimore, Maryland: Johns Hopkins University.
Madagascar	Johns Hopkins University. 2019 Novel Coronavirus COVID-19 (2019-nCoV) Data Repository by Johns Hopkins CSSE. Baltimore, Maryland: Johns Hopkins University.
Malawi	Johns Hopkins University. 2019 Novel Coronavirus COVID-19 (2019-nCoV) Data Repository by Johns Hopkins CSSE. Baltimore, Maryland: Johns Hopkins University.
Malaysia	Johns Hopkins University. 2019 Novel Coronavirus COVID-19 (2019-nCoV) Data Repository by Johns Hopkins CSSE. Baltimore, Maryland: Johns Hopkins University.

Maldives	Johns Hopkins University. 2019 Novel Coronavirus COVID-19 (2019-nCoV) Data Repository by Johns Hopkins CSSE. Baltimore, Maryland: Johns Hopkins University.
Mali	Johns Hopkins University. 2019 Novel Coronavirus COVID-19 (2019-nCoV) Data Repository by Johns Hopkins CSSE. Baltimore, Maryland: Johns Hopkins University.
Malta	Johns Hopkins University. 2019 Novel Coronavirus COVID-19 (2019-nCoV) Data Repository by Johns Hopkins CSSE. Baltimore, Maryland: Johns Hopkins University.
Marshall Islands	Johns Hopkins University. 2019 Novel Coronavirus COVID-19 (2019-nCoV) Data Repository by Johns Hopkins CSSE. Baltimore, Maryland: Johns Hopkins University.
Mauritania	Johns Hopkins University. 2019 Novel Coronavirus COVID-19 (2019-nCoV) Data Repository by Johns Hopkins CSSE. Baltimore, Maryland: Johns Hopkins University.
Mauritius	Johns Hopkins University. 2019 Novel Coronavirus COVID-19 (2019-nCoV) Data Repository by Johns Hopkins CSSE. Baltimore, Maryland: Johns Hopkins University.
Mexico, Aguascalientes	Directorate General of Epidemiology, Secretariat of Health (Mexico), National Institute of Statistics and Geography (INEGI) (Mexico). Mexico COVID-19 Deaths 2020.
Mexico, Baja California	Directorate General of Epidemiology, Secretariat of Health (Mexico), National Institute of Statistics and Geography (INEGI) (Mexico). Mexico COVID-19 Deaths 2020.
Mexico, Baja California Sur	Directorate General of Epidemiology, Secretariat of Health (Mexico), National Institute of Statistics and Geography (INEGI) (Mexico). Mexico COVID-19 Deaths 2020.
Mexico, Campeche	Directorate General of Epidemiology, Secretariat of Health (Mexico), National Institute of Statistics and Geography (INEGI) (Mexico). Mexico COVID-19 Deaths 2020.
Mexico, Chiapas	Directorate General of Epidemiology, Secretariat of Health (Mexico), National Institute of Statistics and Geography (INEGI) (Mexico). Mexico COVID-19 Deaths 2020.
Mexico, Chihuahua	Directorate General of Epidemiology, Secretariat of Health (Mexico), National Institute of Statistics and Geography (INEGI) (Mexico). Mexico COVID-19 Deaths 2020.
Mexico, Coahuila	Directorate General of Epidemiology, Secretariat of Health (Mexico), National Institute of Statistics and Geography (INEGI) (Mexico). Mexico COVID-19 Deaths 2020.
Mexico, Colima	Directorate General of Epidemiology, Secretariat of Health (Mexico), National Institute of Statistics and Geography (INEGI) (Mexico). Mexico COVID-19 Deaths 2020.
Mexico, Durango	Directorate General of Epidemiology, Secretariat of Health (Mexico), National Institute of Statistics and Geography (INEGI) (Mexico). Mexico COVID-19 Deaths 2020.
Mexico, Guanajuato	Directorate General of Epidemiology, Secretariat of Health (Mexico), National Institute of Statistics and Geography (INEGI) (Mexico). Mexico COVID-19 Deaths 2020.
Mexico, Guerrero	Directorate General of Epidemiology, Secretariat of Health (Mexico), National Institute of Statistics and Geography (INEGI) (Mexico). Mexico COVID-19 Deaths 2020.
Mexico, Hidalgo	Directorate General of Epidemiology, Secretariat of Health (Mexico), National Institute of Statistics and Geography (INEGI) (Mexico). Mexico COVID-19 Deaths 2020.
Mexico, Jalisco	Directorate General of Epidemiology, Secretariat of Health (Mexico), National Institute of Statistics and Geography (INEGI) (Mexico). Mexico COVID-19 Deaths 2020.
Mexico, México	Directorate General of Epidemiology, Secretariat of Health (Mexico), National Institute of Statistics and Geography (INEGI) (Mexico). Mexico COVID-19 Deaths 2020.
Mexico, Mexico City	Directorate General of Epidemiology, Secretariat of Health (Mexico), National Institute of Statistics and Geography (INEGI) (Mexico). Mexico COVID-19 Deaths 2020.
Mexico, Michoacán de Ocampo	Directorate General of Epidemiology, Secretariat of Health (Mexico), National Institute of Statistics and Geography (INEGI) (Mexico). Mexico COVID-19 Deaths 2020.
Mexico, Morelos	Directorate General of Epidemiology, Secretariat of Health (Mexico), National Institute of Statistics and Geography (INEGI) (Mexico). Mexico COVID-19 Deaths 2020.
Mexico, Nayarit	Directorate General of Epidemiology, Secretariat of Health (Mexico), National Institute of Statistics and Geography (INEGI) (Mexico). Mexico COVID-19 Deaths 2020.
Mexico, Nuevo León	Directorate General of Epidemiology, Secretariat of Health (Mexico), National Institute of Statistics and Geography (INEGI) (Mexico). Mexico COVID-19 Deaths 2020.
Mexico, Oaxaca	Directorate General of Epidemiology, Secretariat of Health (Mexico), National Institute of Statistics and Geography (INEGI) (Mexico). Mexico COVID-19 Deaths 2020.
Mexico, Puebla	Directorate General of Epidemiology, Secretariat of Health (Mexico), National Institute of Statistics and Geography (INEGI) (Mexico). Mexico COVID-19 Deaths 2020.
Mexico, Querétaro	Directorate General of Epidemiology, Secretariat of Health (Mexico), National Institute of Statistics and Geography (INEGI) (Mexico). Mexico COVID-19 Deaths 2020.

Mexico, Quintana Roo	Directorate General of Epidemiology, Secretariat of Health (Mexico), National Institute of Statistics and Geography (INEGI) (Mexico). Mexico COVID-19 Deaths 2020.
Mexico, San Luis Potosí	Directorate General of Epidemiology, Secretariat of Health (Mexico), National Institute of Statistics and Geography (INEGI) (Mexico). Mexico COVID-19 Deaths 2020.
Mexico, Sinaloa	Directorate General of Epidemiology, Secretariat of Health (Mexico), National Institute of Statistics and Geography (INEGI) (Mexico). Mexico COVID-19 Deaths 2020.
Mexico, Sonora	Directorate General of Epidemiology, Secretariat of Health (Mexico), National Institute of Statistics and Geography (INEGI) (Mexico). Mexico COVID-19 Deaths 2020.
Mexico, Tabasco	Directorate General of Epidemiology, Secretariat of Health (Mexico), National Institute of Statistics and Geography (INEGI) (Mexico). Mexico COVID-19 Deaths 2020.
Mexico, Tamaulipas	Directorate General of Epidemiology, Secretariat of Health (Mexico), National Institute of Statistics and Geography (INEGI) (Mexico). Mexico COVID-19 Deaths 2020.
Mexico, Tlaxcala	Directorate General of Epidemiology, Secretariat of Health (Mexico), National Institute of Statistics and Geography (INEGI) (Mexico). Mexico COVID-19 Deaths 2020.
Mexico, Veracruz de Ignacio de la Llave	Directorate General of Epidemiology, Secretariat of Health (Mexico), National Institute of Statistics and Geography (INEGI) (Mexico). Mexico COVID-19 Deaths 2020.
Mexico, Yucatán	Directorate General of Epidemiology, Secretariat of Health (Mexico), National Institute of Statistics and Geography (INEGI) (Mexico). Mexico COVID-19 Deaths 2020.
Mexico, Zacatecas	Directorate General of Epidemiology, Secretariat of Health (Mexico), National Institute of Statistics and Geography (INEGI) (Mexico). Mexico COVID-19 Deaths 2020.
Moldova	Johns Hopkins University. 2019 Novel Coronavirus COVID-19 (2019-nCoV) Data Repository by Johns Hopkins CSSE. Baltimore, Maryland: Johns Hopkins University.
Monaco	Johns Hopkins University. 2019 Novel Coronavirus COVID-19 (2019-nCoV) Data Repository by Johns Hopkins CSSE. Baltimore, Maryland: Johns Hopkins University.
Mongolia	Johns Hopkins University. 2019 Novel Coronavirus COVID-19 (2019-nCoV) Data Repository by Johns Hopkins CSSE. Baltimore, Maryland: Johns Hopkins University.
Montenegro	Johns Hopkins University. 2019 Novel Coronavirus COVID-19 (2019-nCoV) Data Repository by Johns Hopkins CSSE. Baltimore, Maryland: Johns Hopkins University.
Morocco	Johns Hopkins University. 2019 Novel Coronavirus COVID-19 (2019-nCoV) Data Repository by Johns Hopkins CSSE. Baltimore, Maryland: Johns Hopkins University.
Mozambique	Johns Hopkins University. 2019 Novel Coronavirus COVID-19 (2019-nCoV) Data Repository by Johns Hopkins CSSE. Baltimore, Maryland: Johns Hopkins University.
Myanmar	Johns Hopkins University. 2019 Novel Coronavirus COVID-19 (2019-nCoV) Data Repository by Johns Hopkins CSSE. Baltimore, Maryland: Johns Hopkins University.
Namibia	Johns Hopkins University. 2019 Novel Coronavirus COVID-19 (2019-nCoV) Data Repository by Johns Hopkins CSSE. Baltimore, Maryland: Johns Hopkins University.
Nepal	Johns Hopkins University. 2019 Novel Coronavirus COVID-19 (2019-nCoV) Data Repository by Johns Hopkins CSSE. Baltimore, Maryland: Johns Hopkins University.
Netherlands	Johns Hopkins University. 2019 Novel Coronavirus COVID-19 (2019-nCoV) Data Repository by Johns Hopkins CSSE. Baltimore, Maryland: Johns Hopkins University.
New Zealand	Johns Hopkins University. 2019 Novel Coronavirus COVID-19 (2019-nCoV) Data Repository by Johns Hopkins CSSE. Baltimore, Maryland: Johns Hopkins University.
Nicaragua	Johns Hopkins University. 2019 Novel Coronavirus COVID-19 (2019-nCoV) Data Repository by Johns Hopkins CSSE. Baltimore, Maryland: Johns Hopkins University.

Niger	Johns Hopkins University. 2019 Novel Coronavirus COVID-19 (2019-nCoV) Data Repository by Johns Hopkins CSSE. Baltimore, Maryland: Johns Hopkins University.
Nigeria	Johns Hopkins University. 2019 Novel Coronavirus COVID-19 (2019-nCoV) Data Repository by Johns Hopkins CSSE. Baltimore, Maryland: Johns Hopkins University.
North Macedonia	Johns Hopkins University. 2019 Novel Coronavirus COVID-19 (2019-nCoV) Data Repository by Johns Hopkins CSSE. Baltimore, Maryland: Johns Hopkins University.
Northern Mariana Islands	Johns Hopkins University. 2019 Novel Coronavirus COVID-19 (2019-nCoV) Data Repository by Johns Hopkins CSSE. Baltimore, Maryland: Johns Hopkins University.
Norway	Johns Hopkins University. 2019 Novel Coronavirus COVID-19 (2019-nCoV) Data Repository by Johns Hopkins CSSE. Baltimore, Maryland: Johns Hopkins University.
Oman	Johns Hopkins University. 2019 Novel Coronavirus COVID-19 (2019-nCoV) Data Repository by Johns Hopkins CSSE. Baltimore, Maryland: Johns Hopkins University.
Pakistan, Azad Jammu & Kashmir	Ministry of National Health Services, Regulations & Coordination (Pakistan). Pakistan - Azad Jammu and Kashmir COVID-19 Statistics. Islamabad, Pakistan: Government of Pakistan.
Pakistan, Azad Jammu & Kashmir	Ministry of National Health Services, Regulations & Coordination (Pakistan). Pakistan COVID-19 Dashboard.
Pakistan, Balochistan	Ministry of National Health Services, Regulations & Coordination (Pakistan). Pakistan - Balochistan COVID-19 Statistics. Islamabad, Pakistan: Government of Pakistan.
Pakistan, Balochistan	Ministry of National Health Services, Regulations & Coordination (Pakistan). Pakistan COVID-19 Dashboard.
Pakistan, Gilgit-Baltistan	Ministry of National Health Services, Regulations & Coordination (Pakistan). Pakistan - Gilgit-Baltistan COVID-19 Statistics. Islamabad, Pakistan: Government of Pakistan.
Pakistan, Gilgit-Baltistan	Ministry of National Health Services, Regulations & Coordination (Pakistan). Pakistan COVID-19 Dashboard.
Pakistan, Islamabad Capital Territory	Ministry of National Health Services, Regulations & Coordination (Pakistan). Pakistan - Islāmābād COVID-19 Statistics. Islāmābād, Pakistan: Government of Pakistan.
Pakistan, Islamabad Capital Territory	Ministry of National Health Services, Regulations & Coordination (Pakistan). Pakistan COVID-19 Dashboard.
Pakistan, Khyber Pakhtunkhwa	Ministry of National Health Services, Regulations & Coordination (Pakistan). Pakistan - Khyber Pakhtunkhwa COVID-19 Statistics 2020.
Pakistan, Khyber Pakhtunkhwa	Ministry of National Health Services, Regulations & Coordination (Pakistan). Pakistan COVID-19 Dashboard.
Pakistan, Punjab	Ministry of National Health Services, Regulations & Coordination (Pakistan). Pakistan - Punjab COVID-19 Statistics. Islamabad, Pakistan: Government of Pakistan.

Pakistan, Punjab	Ministry of National Health Services, Regulations & Coordination (Pakistan). Pakistan COVID-19 Dashboard.
Pakistan, Sindh	Wikipedia. COVID-19 pandemic in Pakistan. San Francisco, United States of America: Wikipedia. Retrieved on April 29, 2021 fromÂ https://en.wikipedia.org/wiki/COVID-19_pandemic_in_Pakistan
Pakistan, Sindh	Ministry of National Health Services, Regulations & Coordination (Pakistan). Pakistan - Sindh COVID-19 Statistics. Islamabad, Pakistan: Government of Pakistan.
Pakistan, Sindh	Ministry of National Health Services, Regulations & Coordination (Pakistan). Pakistan COVID-19 Dashboard.
Palau	Johns Hopkins University. 2019 Novel Coronavirus COVID-19 (2019-nCoV) Data Repository by Johns Hopkins CSSE. Baltimore, Maryland: Johns Hopkins University.
Palestine	Johns Hopkins University. 2019 Novel Coronavirus COVID-19 (2019-nCoV) Data Repository by Johns Hopkins CSSE. Baltimore, Maryland: Johns Hopkins University.
Panama	Johns Hopkins University. 2019 Novel Coronavirus COVID-19 (2019-nCoV) Data Repository by Johns Hopkins CSSE. Baltimore, Maryland: Johns Hopkins University.
Papua New Guinea	Johns Hopkins University. 2019 Novel Coronavirus COVID-19 (2019-nCoV) Data Repository by Johns Hopkins CSSE. Baltimore, Maryland: Johns Hopkins University.
Paraguay	Johns Hopkins University. 2019 Novel Coronavirus COVID-19 (2019-nCoV) Data Repository by Johns Hopkins CSSE. Baltimore, Maryland: Johns Hopkins University.
Peru	Ministry of Health (Peru), National Center for Epidemiology, Prevention and Disease Control (Peru). Peru Deaths by COVID-19.
Philippines	Department of Health (Philippines). Philippines Department of Health COVID-19 Tracker. Manila, Philippines: Department of Health (Philippines).
Poland	Johns Hopkins University. 2019 Novel Coronavirus COVID-19 (2019-nCoV) Data Repository by Johns Hopkins CSSE. Baltimore, Maryland: Johns Hopkins University.
Portugal	Johns Hopkins University. 2019 Novel Coronavirus COVID-19 (2019-nCoV) Data Repository by Johns Hopkins CSSE. Baltimore, Maryland: Johns Hopkins University.
Puerto Rico	Johns Hopkins University. 2019 Novel Coronavirus COVID-19 (2019-nCoV) Data Repository by Johns Hopkins CSSE. Baltimore, Maryland: Johns Hopkins University.
Qatar	Johns Hopkins University. 2019 Novel Coronavirus COVID-19 (2019-nCoV) Data Repository by Johns Hopkins CSSE. Baltimore, Maryland: Johns Hopkins University.
Romania	Johns Hopkins University. 2019 Novel Coronavirus COVID-19 (2019-nCoV) Data Repository by Johns Hopkins CSSE. Baltimore, Maryland: Johns Hopkins University.
Russia	Federal State Statistics Service (Russia). Russia Natural Movement of the Population 2021. Moscow, Russian Federation: Federal State Statistics Service (Russia).

Rwanda	Johns Hopkins University. 2019 Novel Coronavirus COVID-19 (2019-nCoV) Data Repository by Johns Hopkins CSSE. Baltimore, Maryland: Johns Hopkins University.
Saint Kitts and Nevis	Johns Hopkins University. 2019 Novel Coronavirus COVID-19 (2019-nCoV) Data Repository by Johns Hopkins CSSE. Baltimore, Maryland: Johns Hopkins University.
Saint Lucia	Johns Hopkins University. 2019 Novel Coronavirus COVID-19 (2019-nCoV) Data Repository by Johns Hopkins CSSE. Baltimore, Maryland: Johns Hopkins University.
Saint Vincent and the Grenadines	Johns Hopkins University. 2019 Novel Coronavirus COVID-19 (2019-nCoV) Data Repository by Johns Hopkins CSSE. Baltimore, Maryland: Johns Hopkins University.
Samoa	Johns Hopkins University. 2019 Novel Coronavirus COVID-19 (2019-nCoV) Data Repository by Johns Hopkins CSSE. Baltimore, Maryland: Johns Hopkins University.
San Marino	Johns Hopkins University. 2019 Novel Coronavirus COVID-19 (2019-nCoV) Data Repository by Johns Hopkins CSSE. Baltimore, Maryland: Johns Hopkins University.
São Tomé and Príncipe	Johns Hopkins University. 2019 Novel Coronavirus COVID-19 (2019-nCoV) Data Repository by Johns Hopkins CSSE. Baltimore, Maryland: Johns Hopkins University.
Saudi Arabia	Johns Hopkins University. 2019 Novel Coronavirus COVID-19 (2019-nCoV) Data Repository by Johns Hopkins CSSE. Baltimore, Maryland: Johns Hopkins University.
Senegal	Johns Hopkins University. 2019 Novel Coronavirus COVID-19 (2019-nCoV) Data Repository by Johns Hopkins CSSE. Baltimore, Maryland: Johns Hopkins University.
Serbia	Johns Hopkins University. 2019 Novel Coronavirus COVID-19 (2019-nCoV) Data Repository by Johns Hopkins CSSE. Baltimore, Maryland: Johns Hopkins University.
Seychelles	Johns Hopkins University. 2019 Novel Coronavirus COVID-19 (2019-nCoV) Data Repository by Johns Hopkins CSSE. Baltimore, Maryland: Johns Hopkins University.
Sierra Leone	Johns Hopkins University. 2019 Novel Coronavirus COVID-19 (2019-nCoV) Data Repository by Johns Hopkins CSSE. Baltimore, Maryland: Johns Hopkins University.
Singapore	Johns Hopkins University. 2019 Novel Coronavirus COVID-19 (2019-nCoV) Data Repository by Johns Hopkins CSSE. Baltimore, Maryland: Johns Hopkins University.
Slovakia	Johns Hopkins University. 2019 Novel Coronavirus COVID-19 (2019-nCoV) Data Repository by Johns Hopkins CSSE. Baltimore, Maryland: Johns Hopkins University.
Slovenia	Johns Hopkins University. 2019 Novel Coronavirus COVID-19 (2019-nCoV) Data Repository by Johns Hopkins CSSE. Baltimore, Maryland: Johns Hopkins University.
Solomon Islands	Johns Hopkins University. 2019 Novel Coronavirus COVID-19 (2019-nCoV) Data Repository by Johns Hopkins CSSE. Baltimore, Maryland: Johns Hopkins University.
Somalia	Johns Hopkins University. 2019 Novel Coronavirus COVID-19 (2019-nCoV) Data Repository by Johns Hopkins CSSE. Baltimore, Maryland: Johns Hopkins University.

South Africa	Johns Hopkins University. 2019 Novel Coronavirus COVID-19 (2019-nCoV) Data Repository by Johns Hopkins CSSE. Baltimore, Maryland: Johns Hopkins University.
South Korea	Johns Hopkins University. 2019 Novel Coronavirus COVID-19 (2019-nCoV) Data Repository by Johns Hopkins CSSE. Baltimore, Maryland: Johns Hopkins University.
South Sudan	Johns Hopkins University. 2019 Novel Coronavirus COVID-19 (2019-nCoV) Data Repository by Johns Hopkins CSSE. Baltimore, Maryland: Johns Hopkins University.
Spain, Andalusia	Andalusia Ministry of Health and Families (Spain). Spain - Andalusia Ministry of Health and Families Coronavirus Report.
Spain, Aragon	Ministry of Health, Consumption and Social Welfare (Spain). Spain Ministry of Health, Consumption, and Social Welfare COVID-19 Situation Update. Spain: Ministry of Health, Consumption and Social Welfare (Spain).
Spain, Aragon	Institute of Health Carlos III (Spain), Ministry of Health, Consumption and Social Welfare (Spain). Spain Carlos III Health Institute Situation of COVID-19. Madrid, Spain: Institute of Health Carlos III (Spain).
Spain, Asturias	Ministry of Health, Consumption and Social Welfare (Spain). Spain Ministry of Health, Consumption, and Social Welfare COVID-19 Situation Update. Spain: Ministry of Health, Consumption and Social Welfare (Spain).
Spain, Asturias	Institute of Health Carlos III (Spain), Ministry of Health, Consumption and Social Welfare (Spain). Spain Carlos III Health Institute Situation of COVID-19. Madrid, Spain: Institute of Health Carlos III (Spain).
Spain, Balearic Islands	Government of the Balearic Islands. Spain - Balearic Islands Ministry of Health and Consumption News About the Coronavirus COVID-19.
Spain, Basque Country	Basque Government Department of Health. Spain - Basque Country Information on the Evolution of the Coronavirus Epidemiological Bulletin.
Spain, Canary Islands	Government of the Canary Islands (Spain). Spain - Canary Islands Government COVID-19 Dashboard.
Spain, Cantabria	Cantabrian Health Service (Spain), Government of Cantabria (Spain). Spain - Cantabria Epidemiological Situation of COVID-19. Spain: Cantabrian Health Service (Spain).
Spain, Castile and León	Castile and León Board, Health Commission (Spain). Spain - Castile and León Open Data: Coronavirus (COVID-19) Epidemiological Situation.
Spain, Castile and León	Castile and León Board, Health Commission (Spain). Spain - Castile and León Open Data: Situation of Hospitalized by Coronavirus.
Spain, Castilla–La Mancha	Ministry of Health, Consumption and Social Welfare (Spain). Spain Ministry of Health, Consumption, and Social Welfare COVID-19 Situation Update. Spain: Ministry of Health, Consumption and Social Welfare (Spain).
Spain, Castilla–La Mancha	Institute of Health Carlos III (Spain), Ministry of Health, Consumption and Social Welfare (Spain). Spain Carlos III Health Institute Situation of COVID-19. Madrid, Spain: Institute of Health Carlos III (Spain).

Spain, Catalonia	Statistical Institute of Catalonia (IDESCAT) (Spain). Spain - Catalonia COVID-19 Weekly Confirmed Cases and Deaths. Barcelona, Spain: Statistical Institute of Catalonia (IDESCAT) (Spain).
Spain, Catalonia	Statistical Institute of Catalonia (IDESCAT) (Spain). Spain - Catalonia COVID-19 Daily Confirmed Cases and Deaths 2020. Barcelona, Spain: Statistical Institute of Catalonia (IDESCAT) (Spain), 2020.
Spain, Ceuta	Ministry of Health, Consumption and Social Welfare (Spain). Spain Ministry of Health, Consumption, and Social Welfare COVID-19 Situation Update. Spain: Ministry of Health, Consumption and Social Welfare (Spain).
Spain, Ceuta	Institute of Health Carlos III (Spain), Ministry of Health, Consumption and Social Welfare (Spain). Spain Carlos III Health Institute Situation of COVID-19. Madrid, Spain: Institute of Health Carlos III (Spain).
Spain, Community of Madrid	Ministry of Health, Consumption and Social Welfare (Spain). Spain Ministry of Health, Consumption, and Social Welfare COVID-19 Situation Update. Spain: Ministry of Health, Consumption and Social Welfare (Spain).
Spain, Community of Madrid	City of Madrid (Spain), Madrid Health Service (Spain). Spain - Madrid Health Service COVID-19 Current Situation Daily Status Report. Madrid, Spain: City of Madrid (Spain).
Spain, Extremadura	Ministry of Health, Consumption and Social Welfare (Spain). Spain Ministry of Health, Consumption, and Social Welfare COVID-19 Situation Update. Spain: Ministry of Health, Consumption and Social Welfare (Spain).
Spain, Galicia	Galician Healthcare Service (Spain), Regional Government of Galicia (Spain). Spain - Galicia Regional Government COVID-19 Press Releases 2020. Spain: Regional Government of Galicia (Spain).
Spain, La Rioja	Government of La Rioja (Spain). Spain - La Rioja Covid-19 Tests Performed per Days. Brazil: Government of La Rioja (Spain).
Spain, Melilla	Ministry of Health, Consumption and Social Welfare (Spain). Spain Ministry of Health, Consumption, and Social Welfare COVID-19 Situation Update. Spain: Ministry of Health, Consumption and Social Welfare (Spain).
Spain, Melilla	Institute of Health Carlos III (Spain), Ministry of Health, Consumption and Social Welfare (Spain). Spain Carlos III Health Institute Situation of COVID-19. Madrid, Spain: Institute of Health Carlos III (Spain).
Spain, Murcia	Institute of Health Carlos III (Spain), Ministry of Health, Consumption and Social Welfare (Spain). Spain Carlos III Health Institute Situation of COVID-19. Madrid, Spain: Institute of Health Carlos III (Spain).
Spain, Murcia	Ministry of Health of the Region of Murcia (Spain). COVID-19 Region of Murcia - Spain. Spain: Ministry of Health of the Region of Murcia (Spain).
Spain, Navarre	Government of Navarra (Spain). Spain - Navarra COVID-19 Testing, Deaths, Hospitalization Data May-June 2020. Spain: Government of Navarra (Spain), 2020.
Spain, Valencian Community	Valencia Ministry of Universal Health and Public Health (Spain). Spain - Valencia COVID-19: Monitoring of the Situation. Spain: Valencia Ministry of Universal Health and Public Health (Spain).
Sri Lanka	Johns Hopkins University. 2019 Novel Coronavirus COVID-19 (2019-nCoV) Data Repository by Johns Hopkins CSSE. Baltimore, Maryland: Johns Hopkins University.

Sudan	Johns Hopkins University. 2019 Novel Coronavirus COVID-19 (2019-nCoV) Data Repository by Johns Hopkins CSSE. Baltimore, Maryland: Johns Hopkins University.
Suriname	Johns Hopkins University. 2019 Novel Coronavirus COVID-19 (2019-nCoV) Data Repository by Johns Hopkins CSSE. Baltimore, Maryland: Johns Hopkins University.
Sweden	Public Health Agency of Sweden. Sweden Public Health Agency COVID-19 Confirmed Cases Daily Update. Östersund, Sweden: Public Health Agency of Sweden.
Switzerland	Federal Office of Public Health (Switzerland). Switzerland Federal Office of Public Health New Coronavirus Current Situation. Berne, Switzerland: Federal Office of Public Health (Switzerland).
Syria	Johns Hopkins University. 2019 Novel Coronavirus COVID-19 (2019-nCoV) Data Repository by Johns Hopkins CSSE. Baltimore, Maryland: Johns Hopkins University.
Taiwan (Province of China)	Johns Hopkins University. 2019 Novel Coronavirus COVID-19 (2019-nCoV) Data Repository by Johns Hopkins CSSE. Baltimore, Maryland: Johns Hopkins University.
Tajikistan	Johns Hopkins University. 2019 Novel Coronavirus COVID-19 (2019-nCoV) Data Repository by Johns Hopkins CSSE. Baltimore, Maryland: Johns Hopkins University.
Tanzania	Johns Hopkins University. 2019 Novel Coronavirus COVID-19 (2019-nCoV) Data Repository by Johns Hopkins CSSE. Baltimore, Maryland: Johns Hopkins University.
Thailand	Johns Hopkins University. 2019 Novel Coronavirus COVID-19 (2019-nCoV) Data Repository by Johns Hopkins CSSE. Baltimore, Maryland: Johns Hopkins University.
The Bahamas	Johns Hopkins University. 2019 Novel Coronavirus COVID-19 (2019-nCoV) Data Repository by Johns Hopkins CSSE. Baltimore, Maryland: Johns Hopkins University.
The Gambia	Johns Hopkins University. 2019 Novel Coronavirus COVID-19 (2019-nCoV) Data Repository by Johns Hopkins CSSE. Baltimore, Maryland: Johns Hopkins University.
Timor-Leste	Johns Hopkins University. 2019 Novel Coronavirus COVID-19 (2019-nCoV) Data Repository by Johns Hopkins CSSE. Baltimore, Maryland: Johns Hopkins University.
Togo	Johns Hopkins University. 2019 Novel Coronavirus COVID-19 (2019-nCoV) Data Repository by Johns Hopkins CSSE. Baltimore, Maryland: Johns Hopkins University.
Trinidad and Tobago	Johns Hopkins University. 2019 Novel Coronavirus COVID-19 (2019-nCoV) Data Repository by Johns Hopkins CSSE. Baltimore, Maryland: Johns Hopkins University.
Tunisia	Johns Hopkins University. 2019 Novel Coronavirus COVID-19 (2019-nCoV) Data Repository by Johns Hopkins CSSE. Baltimore, Maryland: Johns Hopkins University.
Turkey	Johns Hopkins University. 2019 Novel Coronavirus COVID-19 (2019-nCoV) Data Repository by Johns Hopkins CSSE. Baltimore, Maryland: Johns Hopkins University.
Uganda	Johns Hopkins University. 2019 Novel Coronavirus COVID-19 (2019-nCoV) Data Repository by Johns Hopkins CSSE. Baltimore, Maryland: Johns Hopkins University.

UK, England	Office for National Statistics (ONS) (United Kingdom). United Kingdom - England and Wales Deaths Registered Weekly, Provisional.
UK, Northern Ireland	Office for National Statistics (ONS) (United Kingdom). United Kingdom - England and Wales Deaths Registered Weekly, Provisional.
UK, Scotland	Office for National Statistics (ONS) (United Kingdom). United Kingdom - England and Wales Deaths Registered Weekly, Provisional.
UK, Wales	Office for National Statistics (ONS) (United Kingdom). United Kingdom - England and Wales Deaths Registered Weekly, Provisional.
Ukraine	Johns Hopkins University. 2019 Novel Coronavirus COVID-19 (2019-nCoV) Data Repository by Johns Hopkins CSSE. Baltimore, Maryland: Johns Hopkins University.
United Arab Emirates	Johns Hopkins University. 2019 Novel Coronavirus COVID-19 (2019-nCoV) Data Repository by Johns Hopkins CSSE. Baltimore, Maryland: Johns Hopkins University.
Uruguay	Johns Hopkins University. 2019 Novel Coronavirus COVID-19 (2019-nCoV) Data Repository by Johns Hopkins CSSE. Baltimore, Maryland: Johns Hopkins University.
USA, Alabama	Johns Hopkins University. 2019 Novel Coronavirus COVID-19 (2019-nCoV) Data Repository by Johns Hopkins CSSE. Baltimore, Maryland: Johns Hopkins University.
USA, Alaska	Johns Hopkins University. 2019 Novel Coronavirus COVID-19 (2019-nCoV) Data Repository by Johns Hopkins CSSE. Baltimore, Maryland: Johns Hopkins University.
USA, Arizona	Johns Hopkins University. 2019 Novel Coronavirus COVID-19 (2019-nCoV) Data Repository by Johns Hopkins CSSE. Baltimore, Maryland: Johns Hopkins University.
USA, Arkansas	Johns Hopkins University. 2019 Novel Coronavirus COVID-19 (2019-nCoV) Data Repository by Johns Hopkins CSSE. Baltimore, Maryland: Johns Hopkins University.
USA, California	Johns Hopkins University. 2019 Novel Coronavirus COVID-19 (2019-nCoV) Data Repository by Johns Hopkins CSSE. Baltimore, Maryland: Johns Hopkins University.
USA, Colorado	Johns Hopkins University. 2019 Novel Coronavirus COVID-19 (2019-nCoV) Data Repository by Johns Hopkins CSSE. Baltimore, Maryland: Johns Hopkins University.
USA, Connecticut	Johns Hopkins University. 2019 Novel Coronavirus COVID-19 (2019-nCoV) Data Repository by Johns Hopkins CSSE. Baltimore, Maryland: Johns Hopkins University.
USA, Delaware	Delaware Division of Public Health (United States). United States - Delaware Division of Public Health Coronavirus Disease (COVID-19) Data Dashboard. United States of America: Delaware Division of Public Health (United States).
USA, Florida	Johns Hopkins University. 2019 Novel Coronavirus COVID-19 (2019-nCoV) Data Repository by Johns Hopkins CSSE. Baltimore, Maryland: Johns Hopkins University.
USA, Georgia	Johns Hopkins University. 2019 Novel Coronavirus COVID-19 (2019-nCoV) Data Repository by Johns Hopkins CSSE. Baltimore, Maryland: Johns Hopkins University.

USA, Hawaii	Hawaii State Department of Health. United States - Hawaii Department of Health COVID-19 Current Situation. Honolulu, HI, United States of America: Hawaii State Department of Health.
USA, Idaho	Johns Hopkins University. 2019 Novel Coronavirus COVID-19 (2019-nCoV) Data Repository by Johns Hopkins CSSE. Baltimore, Maryland: Johns Hopkins University.
USA, Illinois	Johns Hopkins University. 2019 Novel Coronavirus COVID-19 (2019-nCoV) Data Repository by Johns Hopkins CSSE. Baltimore, Maryland: Johns Hopkins University.
USA, Indiana	Johns Hopkins University. 2019 Novel Coronavirus COVID-19 (2019-nCoV) Data Repository by Johns Hopkins CSSE. Baltimore, Maryland: Johns Hopkins University.
USA, Iowa	Johns Hopkins University. 2019 Novel Coronavirus COVID-19 (2019-nCoV) Data Repository by Johns Hopkins CSSE. Baltimore, Maryland: Johns Hopkins University.
USA, Kansas	Johns Hopkins University. 2019 Novel Coronavirus COVID-19 (2019-nCoV) Data Repository by Johns Hopkins CSSE. Baltimore, Maryland: Johns Hopkins University.
USA, Kentucky	Johns Hopkins University. 2019 Novel Coronavirus COVID-19 (2019-nCoV) Data Repository by Johns Hopkins CSSE. Baltimore, Maryland: Johns Hopkins University.
USA, Louisiana	Johns Hopkins University. 2019 Novel Coronavirus COVID-19 (2019-nCoV) Data Repository by Johns Hopkins CSSE. Baltimore, Maryland: Johns Hopkins University.
USA, Maine	Johns Hopkins University. 2019 Novel Coronavirus COVID-19 (2019-nCoV) Data Repository by Johns Hopkins CSSE. Baltimore, Maryland: Johns Hopkins University.
USA, Maryland	Johns Hopkins University. 2019 Novel Coronavirus COVID-19 (2019-nCoV) Data Repository by Johns Hopkins CSSE. Baltimore, Maryland: Johns Hopkins University.
USA, Massachusetts	Johns Hopkins University. 2019 Novel Coronavirus COVID-19 (2019-nCoV) Data Repository by Johns Hopkins CSSE. Baltimore, Maryland: Johns Hopkins University.
USA, Michigan	Johns Hopkins University. 2019 Novel Coronavirus COVID-19 (2019-nCoV) Data Repository by Johns Hopkins CSSE. Baltimore, Maryland: Johns Hopkins University.
USA, Minnesota	Johns Hopkins University. 2019 Novel Coronavirus COVID-19 (2019-nCoV) Data Repository by Johns Hopkins CSSE. Baltimore, Maryland: Johns Hopkins University.
USA, Mississippi	Johns Hopkins University. 2019 Novel Coronavirus COVID-19 (2019-nCoV) Data Repository by Johns Hopkins CSSE. Baltimore, Maryland: Johns Hopkins University.
USA, Missouri	Johns Hopkins University. 2019 Novel Coronavirus COVID-19 (2019-nCoV) Data Repository by Johns Hopkins CSSE. Baltimore, Maryland: Johns Hopkins University.
USA, Montana	Johns Hopkins University. 2019 Novel Coronavirus COVID-19 (2019-nCoV) Data Repository by Johns Hopkins CSSE. Baltimore, Maryland: Johns Hopkins University.
USA, Nebraska	Johns Hopkins University. 2019 Novel Coronavirus COVID-19 (2019-nCoV) Data Repository by Johns Hopkins CSSE. Baltimore, Maryland: Johns Hopkins University.

USA, Nevada	Johns Hopkins University. 2019 Novel Coronavirus COVID-19 (2019-nCoV) Data Repository by Johns Hopkins CSSE. Baltimore, Maryland: Johns Hopkins University.
USA, New Hampshire	Johns Hopkins University. 2019 Novel Coronavirus COVID-19 (2019-nCoV) Data Repository by Johns Hopkins CSSE. Baltimore, Maryland: Johns Hopkins University.
USA, New Jersey	Johns Hopkins University. 2019 Novel Coronavirus COVID-19 (2019-nCoV) Data Repository by Johns Hopkins CSSE. Baltimore, Maryland: Johns Hopkins University.
USA, New Mexico	Johns Hopkins University. 2019 Novel Coronavirus COVID-19 (2019-nCoV) Data Repository by Johns Hopkins CSSE. Baltimore, Maryland: Johns Hopkins University.
USA, New York	New York Times. COVID-19 Cumulative Deaths and Cases in the United States by County - New York Times. New York, United States of America: New York Times.
USA, New York	New York City Department of Health and Mental Hygiene. United States - New York City Department of Health and Mental Hygiene COVID-19 Data. New York, NY, United States of America: New York City Department of Health and Mental Hygiene.
USA, New York	Johns Hopkins University. 2019 Novel Coronavirus COVID-19 (2019-nCoV) Data Repository by Johns Hopkins CSSE. Baltimore, Maryland: Johns Hopkins University.
USA, North Carolina	Johns Hopkins University. 2019 Novel Coronavirus COVID-19 (2019-nCoV) Data Repository by Johns Hopkins CSSE. Baltimore, Maryland: Johns Hopkins University.
USA, North Dakota	Johns Hopkins University. 2019 Novel Coronavirus COVID-19 (2019-nCoV) Data Repository by Johns Hopkins CSSE. Baltimore, Maryland: Johns Hopkins University.
USA, Ohio	Ohio Department of Health. United States - Ohio Department of Health Coronavirus (COVID-19) Updates. Columbus, OH, United States of America: Ohio Department of Health.
USA, Oklahoma	Johns Hopkins University. 2019 Novel Coronavirus COVID-19 (2019-nCoV) Data Repository by Johns Hopkins CSSE. Baltimore, Maryland: Johns Hopkins University.
USA, Oregon	Oregon Health Authority (United States). United States - Oregon Health Authority COVID-19 Updates. Salem, United States of America: Oregon Health Authority (United States).
USA, Pennsylvania	Johns Hopkins University. 2019 Novel Coronavirus COVID-19 (2019-nCoV) Data Repository by Johns Hopkins CSSE. Baltimore, Maryland: Johns Hopkins University.
USA, Rhode Island	Johns Hopkins University. 2019 Novel Coronavirus COVID-19 (2019-nCoV) Data Repository by Johns Hopkins CSSE. Baltimore, Maryland: Johns Hopkins University.
USA, South Carolina	Johns Hopkins University. 2019 Novel Coronavirus COVID-19 (2019-nCoV) Data Repository by Johns Hopkins CSSE. Baltimore, Maryland: Johns Hopkins University.
USA, South Dakota	Johns Hopkins University. 2019 Novel Coronavirus COVID-19 (2019-nCoV) Data Repository by Johns Hopkins CSSE. Baltimore, Maryland: Johns Hopkins University.
USA, Tennessee	Johns Hopkins University. 2019 Novel Coronavirus COVID-19 (2019-nCoV) Data Repository by Johns Hopkins CSSE. Baltimore, Maryland: Johns Hopkins University.

USA, Texas	Johns Hopkins University. 2019 Novel Coronavirus COVID-19 (2019-nCoV) Data Repository by Johns Hopkins CSSE. Baltimore, Maryland: Johns Hopkins University.
USA, Utah	Johns Hopkins University. 2019 Novel Coronavirus COVID-19 (2019-nCoV) Data Repository by Johns Hopkins CSSE. Baltimore, Maryland: Johns Hopkins University.
USA, Vermont	Johns Hopkins University. 2019 Novel Coronavirus COVID-19 (2019-nCoV) Data Repository by Johns Hopkins CSSE. Baltimore, Maryland: Johns Hopkins University.
USA, Virginia	Johns Hopkins University. 2019 Novel Coronavirus COVID-19 (2019-nCoV) Data Repository by Johns Hopkins CSSE. Baltimore, Maryland: Johns Hopkins University.
USA, Washington	New York Times. COVID-19 Cumulative Deaths and Cases in the United States by County - New York Times. New York, United States of America: New York Times.
USA, Washington, DC	Johns Hopkins University. 2019 Novel Coronavirus COVID-19 (2019-nCoV) Data Repository by Johns Hopkins CSSE. Baltimore, Maryland: Johns Hopkins University.
USA, West Virginia	Johns Hopkins University. 2019 Novel Coronavirus COVID-19 (2019-nCoV) Data Repository by Johns Hopkins CSSE. Baltimore, Maryland: Johns Hopkins University.
USA, Wisconsin	Johns Hopkins University. 2019 Novel Coronavirus COVID-19 (2019-nCoV) Data Repository by Johns Hopkins CSSE. Baltimore, Maryland: Johns Hopkins University.
USA, Wyoming	Johns Hopkins University. 2019 Novel Coronavirus COVID-19 (2019-nCoV) Data Repository by Johns Hopkins CSSE. Baltimore, Maryland: Johns Hopkins University.
Uzbekistan	Johns Hopkins University. 2019 Novel Coronavirus COVID-19 (2019-nCoV) Data Repository by Johns Hopkins CSSE. Baltimore, Maryland: Johns Hopkins University.
Vanuatu	Johns Hopkins University. 2019 Novel Coronavirus COVID-19 (2019-nCoV) Data Repository by Johns Hopkins CSSE. Baltimore, Maryland: Johns Hopkins University.
Venezuela	Johns Hopkins University. 2019 Novel Coronavirus COVID-19 (2019-nCoV) Data Repository by Johns Hopkins CSSE. Baltimore, Maryland: Johns Hopkins University.
Vietnam	Johns Hopkins University. 2019 Novel Coronavirus COVID-19 (2019-nCoV) Data Repository by Johns Hopkins CSSE. Baltimore, Maryland: Johns Hopkins University.
Virgin Islands	Johns Hopkins University. 2019 Novel Coronavirus COVID-19 (2019-nCoV) Data Repository by Johns Hopkins CSSE. Baltimore, Maryland: Johns Hopkins University.
Yemen	Johns Hopkins University. 2019 Novel Coronavirus COVID-19 (2019-nCoV) Data Repository by Johns Hopkins CSSE. Baltimore, Maryland: Johns Hopkins University.
Zambia	Johns Hopkins University. 2019 Novel Coronavirus COVID-19 (2019-nCoV) Data Repository by Johns Hopkins CSSE. Baltimore, Maryland: Johns Hopkins University.
Zimbabwe	Johns Hopkins University. 2019 Novel Coronavirus COVID-19 (2019-nCoV) Data Repository by Johns Hopkins CSSE. Baltimore, Maryland: Johns Hopkins University.

Section 4. Methods

Excess mortality due to COVID-19 is defined as the difference between reported all-cause mortality, after any necessary adjustments including under reporting, and what would have otherwise been observed without the COVID-19 pandemic. This includes both deaths directly attributed to COVID-19 – people who died from the virus – and the net effect of increases or decreases in other causes of death as a result of the pandemic and its associated behavioural and economic changes.

To estimate excess mortality, we need to begin with estimating *expected all-cause mortality* based on past levels and trends. This expected mortality becomes a counterfactual against which we can compare the observed all-cause deaths during the pandemic.

Section 4.1 Estimating expected mortality

In this section, we describe the estimation of expected mortality, only for a subset of locations where time-detailed (weekly or monthly) all-cause mortality data are available for years before and during the pandemic. Time detailed data is used instead of annual data to help capture the effect of the epidemic on all-cause mortality through the course of the pandemic at the population level.

We used an ensemble model to estimate expected mortality in 2020 and 2021, based on observed weekly or monthly all-cause mortality between the earliest year such data is available since 2010 and early 2020 before pandemic started outside of China. This ensemble had three model families: REGMOD, Poisson regression, and a simple assumption where weekly/monthly mortality rate from last-year is held constant, which we will describe in this section.

To formulate the expected mortality models detailed here, let d be death counts, y be year, t be time (week or month), and p be population, and index the data with i.

Each of the models described are fit separately by location for both sexes and all age group combined data.

Section 4.1.1 REGMOD

The first set of submodels in our ensemble belong to a family which uses the `regmod` (https://pypi.org/project/regmod/) package for general regression modelling. While `regmod` is the foundation, we also developed for this project a secondary package specifically intended for excess mortality modelling – `emmodel` (https://github.com/ihmeuw-msca/emmodel).

The `regmod` model examines levels of and trends in mortality in two parts: seasonality and secular time trend.

The seasonality model is formulated as follows:

$$d_i \sim Poisson(\mu_i)$$
$$\mu_i = \exp(\log(p_i) + \text{spline}(t_i))$$

such that death counts are the dependent variable, and they are predicted by a spline on week or month, with a population offset. Note that the year variable does not appear in this step, so the prediction is purely based on seasonality of mortality. Now let the prediction from this step by denoted $\hat{\mu}^s$.

Next, we estimate the secular time trend – or the remaining trend that is not explained by seasonality in mortality. First, create a chronological index for time: $c = ys + t$ where s is the number of detailed time units in a year (12 months; 52 or 53 weeks). Then, we fit a model for time trend:

$$d_i \sim Poisson(\mu_i)$$

$$\mu_i = \exp(\log(\hat{\mu}_i^s) + \text{spline}(c_i))$$

such that the offset is now the prediction from the seasonality model and the predictor is now our chronological index. Let the prediction from this step by denoted $\hat{\mu}^m$. This value becomes our final prediction for expected mortality – it combines both seasonality and the secular time trend.

One challenge with using splines to predict expected mortality is that extrapolation beyond the range of the input data is often highly sensitive to spline specification For the time-trend step, which is responsible for dictating the extrapolation, we chose to use splines with degree 1 (linear) rather than splines with degree 3 (cubic), because the tails on linear splines will not be as extreme, especially for small population where there tends to have more random fluctuation in mortality level in a relatively short time period. The placement of the last internal knot is the other part of spline specification which we found to be impactful. To address this, we included four different `regmod` models in our ensemble, where the four submodels varied on the basis of knot placement (last internal knot 6, 12, 18, and 24 months prior to the last included data point).

Section 4.1.2 Poisson regression

The next model family used fixed effects to model seasonality and yearly effects simultaneously:

$$d_i \sim Poisson(\lambda_i)$$
$$\log(\lambda_i) = \beta + \alpha_y + \alpha_t + \log(p_i)$$

Where β is an intercept, and α_y and α_t are random intercepts on dummy variables for year and time (week or month). We fit this model using the `glm` function in the `stats` R package. One challenge with this model is that the year effect for 2020 comes only from the first three months and is therefore sensitive to 2020 trends in those months. The year effect for 2021 is set equal to the year effect for 2020.

Section 4.1.3 Last-year

The final submodel family for our ensemble is the last-year model. This is a simple model which states that the expected weekly mortality for one year is equal to the observed mortality for the same week in the previous year. For 2021, predicted mortality is observed mortality in 2019. Formally:

$$\hat{d}_{t,y} = d_{t,y-1} \text{ for } y < 2021$$
$$\hat{d}_{t,y} = d_{t,y=2019} \text{ for } y = 2021$$

Section 4.1.4 Ensemble

The ensemble weights are formulated by first fitting all sub-models to data up to March 2019, and then comparing expected mortality for 2019 from these fits to observed mortality between March and December of 2019. This out-of-sample validation test demonstrates model performance at extrapolating levels of mortality outside of the range of the input data and is a proxy for how well the models will perform in predicting expected mortality in the absence of the COVID-19 pandemic.

After fitting our out-of-sample test models, we evaluate root mean square error across all 2019 location-weeks in our analysis (but only for all-age both-sexes combined models):

$$RMSE_m = \sqrt{\frac{\sum_i \left(d_{o,i} - d_{e,i}\right)^2}{n}}$$

Where m is the submodel, d_o is the observed death rate, d_e is the expected death rate from the model prediction, i is an index for location-week, and n is the total number of location- weeks. From here, weights are computed as $\frac{1}{RMSE^2}$ to give larger weights to submodels that have smaller root mean square error on average, effectively giving more weights to models that are more precise based on the global out of sample predictive validity testing. Then, these weights are scaled to sum to 1 across all submodels.

The resulting RMSE values and weights are given in table S4 below:

Table S4. Root Mean Squared Error and weights for models used in the Ensemble modelling process

Model Type	RMSE	Ensemble weight
Regmod, 24 month	0.000693	0.218
Regmod, 18 month	0.000707	0.209
Regmod, 12 month	0.000781	0.171
Regmod, 6 month	0.000968	0.111
Poisson fixed effects	0.000811	0.159
Previous Year	0.000889	0.132

The final expected mortality from the ensemble model is a weighted average of the sub-models, using this weighting scheme.

Section 4.2 Predicting excess mortality for all locations

In our analysis, we predict excess mortality rate for all locations for the uniform cumulative period of January 1, 2020, to December 31, 2021, using a statistical model developed for this project that examines the relationship between excess mortality rate and key covariates pertaining to both the COVID-19 pandemic and background population health metrics. This modelling process involves three steps described below.

Section 4.2.1 Compiling a database on excess mortality rate and its relevant covariates for various time period by location after accounting for late registration and under registration.

As Figure 1 in the manuscript shows, late registration issue is pervasive even for countries that have routine and complete vital registration system evaluated for data released by calendar year. For the empirical excess mortality rates we used in the regression, instead of use all weeks and months with available all-cause mortality data and estimated expected level of mortality, we restricted the input to the calculation of excess mortality to time periods that are not affected by the late registration issue by location. Detailed assessment of late registration issue by location, where routine release of data by week or month is available, can be found in appendix figure S6. In addition, we have also excluded data from weeks 31–33 in western Europe where this were sharp increases in all-cause mortality even though the transmission of COVID-19 was extremely low during the same period. This phenomenon can be demonstrated by reported all-cause mortality in Germany by week between 2016 and 2021 below.

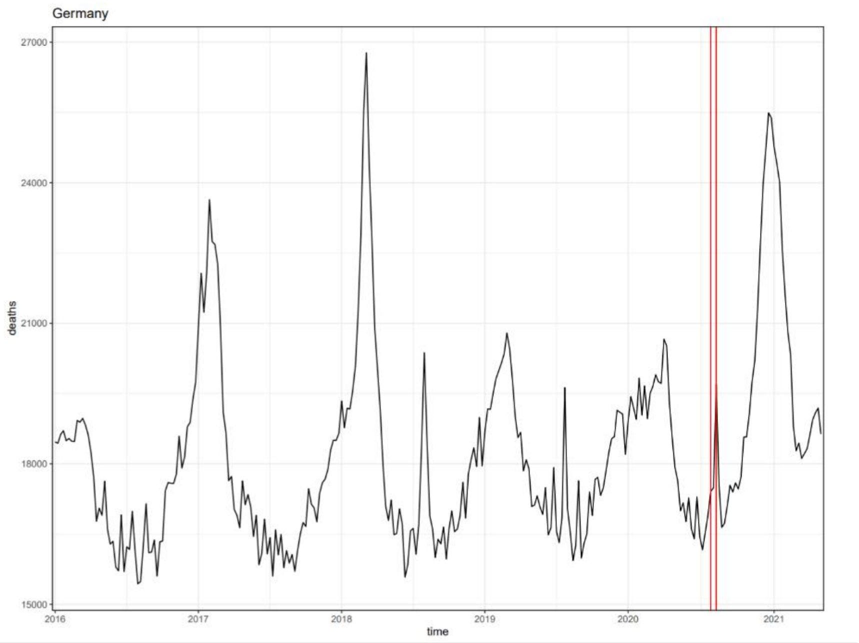

In addition to cumulative excess mortality calculated using time detailed excess mortality estimated using reported all-cause mortality as described above, we also collected excess mortality from two other countries. For South Africa, we used estimated excess mortality rate at province level provided by the Medical Research Council[2] of South Africa where all-cause mortality data for time period during the pandemic have not been made publicly available. For India, while complete and time detailed Sample Registration data and Civil Registration data are not available, report on deaths from the Civil Registration System for selected months during 2020 and 2021 were made available for 12 states in India. After accounting for under registration of the Civil Registration System by comparing reported death from it with those estimated by the Global Burden of Disease Study 2019 at the state level, we calculate excess mortality rate for those states during the months with reported deaths during the pandemic by comparing those numbers with average reported deaths for the same period in year 2018 and 2019.

Section 4.2.2 A statistical model for predicting excess mortality rate

Using data on both the COVID-19 pandemic and background population health variables from the location and time period with cumulative excess mortality as described above, we build a statistical model to predict excess mortality rate using relevant covariates. In addition to COVID-19 related covariates such as seroprevalence, we also examined a variety of population health related covariates as suggested by the meta-analysis conducted by the US Centers for Disease Control and Prevention.[3] The full list of covariates we have examined is shown in the table below:

Table S5. Covariates selected for use in the final model with direction of influence

Covariate	Scale	Direction
Average absolute latitude		Positive
Cardiovascular diseases death rate (2019)	Log	Positive
Crude death rate (2019)	Log	Positive
Diabetes death rate (2019)	Log	Positive
Healthcare access and quality Index (2019)		Negative
HIV death rate (2019)	Log	Positive
Infection detection ratio (lagged)		Negative
Inpatient admission rate (2019)		Negative
Mobility (lagged)		Positive

Proportion of population over age 75		Positive
Quality of death registration system (2019)		Negative
Reported COVID-19 death rate	Log	Positive
Seroprevalence rate (lagged)	Log	Positive
Smoking prevalence (2019)		Positive
Universal health coverage (2019)		Negative

Table S6. List of covariates assessed for use in the model

Covariate
Average absolute latitude
Cardiovascular diseases death rate (2019)
Chronic kidney disease death rate (2019)
Cirrhosis death rate (2019)
Congenital down syndrome death rate (2019)
Crude death rate (2019)
Crude death rate standard deviation 1990 (2019)
Crude death rate standard deviation 2000 (2019)
Cumulative infected
Cumulative infected (lagged)
CVD pulmonary arterial hypertension death rate (2019)
CVD stroke intracerebral haemorrhage death rate (2019)
Diabetes mellitus death rate (2019)
Diabetes prevalence (2019)
Endrocrine, metabolic, blood, and immune disorders death rate (2019)
Healthcare access and quality Index (2019)
HIV death rate (2019)
Hypertension prevelance (2019)
Infection detection ratio
Infection detection ratio (lagged)
Inpatient admission rate (2019)
Mean population age
Mobility
Mobility (lagged)
Neoplasms death rate (2019)
Neurological disorders death rate (2019)
Non-communicable diseases death rate (2019)
Obesity prevalence (2019)
Proportion of population over age 60
Proportion of population over age 70
Proportion of population over age 75
Proportion of population over age 80
Proportion of population over age 85
Quality of death registration system (2019)
Reported COVID-19 death rate
Respiratory asthma death rate (2019)
Seroprevalence rate
Seroprevalence rate (lagged)

Sickle cell disorders death rate (2019)
Smoking prevalence (2019)
Substance abuse death rate (2019)
Thalassemia death rate (2019)
Universal health coverage (2019)

To arrive at a parsimonious model, we used the Least Absolute Shrinkage and Selection Operator (LASSO) regression to help select a list of covariates that have sensible direction of effect on the excess mortality rate due to the COVID-19 pandemic. The final model can be described by the equation below:

$$\ln(y_i) = \alpha + \sum_{j=1}^{16} \beta_j \cdot x_{ij} + \epsilon_i$$

where y_i is the excess mortality rate for location i, and x is the list of covariates selected through the LASSO regression. The list of covariates included in our final model includes: lagged cumulative infections (seroprevalence) rate in log space, COVID-19 death rate in log space, crude death rate in log space, lagged IDR, annual inpatient admissions per capita, diabetes prevalence, HIV death rate in log space, lagged mobility, binned quality of vital registration data, average absolute latitude, chronic kidney disease (CKD) death rate in log space, sickle cell disorders death rate in log space, smoking prevalence, Healthcare Access and Quality Index (HAQ Index) proportion of population aged 75 or older, and substance abuse death rate in log space.

To account for the residuals not accounted for by the selected covariates, we generated regional and super regional level mean residuals for prediction of excess mortality rate for the uniform time period of January 2020 and September 2021. As the empirical data on excess mortality rate for states in India are collected for limited months during the pandemic, average of in-sample state level residuals are used for prediction of excess mortality for all states in India.

Section 4.2.3. Predict excess mortality rate for the cumulative period of January 1 2020 and December, 31 2021

To account for uncertainties in both the coefficients of the covariates and the residuals described above, and the uncertainties in the covariates for the uniform period of January 1 2020 to December 31 2021, the prediction of excess mortality for each location is done at the draw level for 100 times. For each draw level prediction, we first run the same log-linear regression using draw level input excess mortality rate for each location and draw level covariate to estimate both draw level coefficients for the covariates and the residual. Then the draw level coefficients and residuals are paired up with draw level covariates for the uniform cumulative time period of January 1, 2020, to December 31, 2021 to produce excess mortality rate for each location for this draw. The same process is repeated 100 times, from which mean and 95% uncertainty interval of excess mortality rate are generated for each location. Aggregates for region, super region, and global level are generated based these draw level predictions.

We did not set up the excess mortality prediction model to deal with situations where excess mortality rate is negative. Based on our assessment of expected mortality using ensemble model described in section 4.1.1 and the reported all-cause mortality, there are six countries and territories in the world where excess mortality rate during the pandemic was estimated to be negative, after accounting for late registration. Given the limited information available to build a sensible statistical model, we opted to use the excess mortality rate estimated using the ensemble model as the final estimates for these locations.

Section 5. References

1 Stevens GA, Alkema L, Black RE, *et al.* Guidelines for Accurate and Transparent Health Estimates Reporting: the GATHER statement. *The Lancet* 2016; **388**: e19–23.

2 South African Medical Research Council. Report on Weekly Deaths in South Africa. South African Medical Research Council. 2022; published online Feb 9. https://www.samrc.ac.za/reports/report-weekly-deaths-south-africa (accessed Feb 11, 2022).

3 Centers for Disease Control and Prevention. Science brief: evidence used to update the list of underlying medical conditions associated with higher risk for severe COVID-19. Centers for Disease Control and Prevention. 2021; published online Oct 14. https://www.cdc.gov/coronavirus/2019-ncov/science/science-briefs/underlying-evidence-table.html (accessed Oct 29, 2021).

Section 6. Author contributions

Managing the estimation or publication process

Tahiya Alam and Haidong Wang.

Writing the first draft of the manuscript

Catherine Bisignano, Katherine R Paulson, and Haidong Wang.

Primary responsibility for applying analytical methods to produce estimates

Aleksandr Y Aravkin, Ryan M Barber, Haley Comfort, Katherine R Paulson, Spencer A Pease, Stefanie Watson, and Peng Zheng.

Primary responsibility for seeking, cataloguing, extracting, or cleaning data; designing or coding figures and tables

Bree Bang-Jensen, Jhilik Chattopadhyay, Rebecca M Cogen, Haley Comfort, Samuel B Ewald, Alize J Ferrari, Meghan E Frisch, John E Fuller, Gaorui Guo, Monika Helak, Erin N Hulland, Alice Lazzar-Atwood, Kate E LeGrand, Akiaja Lindstrom, Ana M Mantilla Herrera, Erin A May, Ali H Mokdad, Mohsen Naghavi, Paulami Naik, James Kevin O'Halloran, Katherine R Paulson, Louise Penberthy, David M Pigott, Damian Francesco Santomauro, Emma Elizabeth Spurlock, Ruri Syailendrawati, Christopher E Troeger, Haidong Wang, Stefanie Watson, and Bethany Zigler.

Providing data or critical feedback on data sources

Cristiana Abbafati, Christopher Adolph, Bree Bang-Jensen, Ryan M Barber, Gregory J Bertolacci, Suman Chakrabarti, William James Dangel, Carolyn Dapper, Bruce B Duncan, Megan Erickson, Nancy Fullman, Emmanuela Gakidou, John Gallagher, Amiran Gamkrelidze, Gaorui Guo, Monika Helak, Erin N Hulland, Darwin Phan Jones, Maia Kereselidze, Kate E LeGrand, Paulo A Lotufo, Rafael Lozano, Beatrice Magistro, Deborah Carvalho Malta, Johan Månsson, Fatima Marinho, Alemnesh H Mirkuzie, Ali H Mokdad, Lorenzo Monasta, Christopher J L Murray, Mohsen Naghavi, Shuhei Nomura, Edward G O'Brien, Latera Tesfaye Olana, David M Pigott, Grace Reinke, Antonio Luiz P Ribeiro, Damian Francesco Santomauro, Maria Inês Schmidt, David H Shaw, Brittney S Sheena, Natia Skhvitaridze, Reed J D Sorensen, Awoke Temesgen Misganaw, Rebecca Walcott, Haidong Wang, Stefanie Watson, Charles Shey Wiysonge, and Nahom Alemseged Worku.

Developing methods or computational machinery

Adrien Allorant, Aleksandr Y Aravkin, Austin Carter, Emma Castro, Suman Chakrabarti, James K Collins, Haley Comfort, Kimberly Cooperrider, Xiaochen Dai, Farah Daoud, Tatiana Fedosseeva, Joseph Jon Frostad, Gaorui Guo, Jiawei He, Nathaniel J Henry, Emily Linebarger, Ali H Mokdad, Christopher J LMurray, Mohsen Naghavi, Katherine R Paulson, Spencer A Pease, Robert C Reiner Jr, David H Shaw, Brittney S Sheena, Aleksei Sholokhov, Reed J DSorensen, Emma Elizabeth Spurlock, Haidong Wang, and Peng Zheng.

Providing critical feedback on methods or results

Cristiana Abbafati, Ryan M Barber, James K Collins, Xiaochen Dai, William James Dangel, Emmanuela Gakidou, Amiran Gamkrelidze, Simon I Hay, Nathaniel J Henry, Stephen S Lim, Rafael Lozano, Deborah Carvalho Malta, Fatima Marinho, Alemnesh H Mirkuzie, Ali H Mokdad, Christopher J L Murray, Mohsen Naghavi, Latera Tesfaye Olana, Samuel M Ostroff, Katherine R Paulson, Spencer A Pease, David M Pigott, Robert C Reiner Jr, Antonio Luiz P Ribeiro, Natia Skhvitaridze, Reed J D Sorensen, Emma Elizabeth Spurlock, Roman Topor-Madry, Christopher E Troeger, Ally Walker, Haidong Wang, Stefanie Watson, Charles Shey Wiysonge, and Nahom Alemseged Worku.

Drafting the work or revising it critically for important intellectual content

Cristiana Abbafati, Catherine Bisignano, Haley Comfort, Simon I Hay, Paulo A Lotufo, Deborah Carvalho Malta, Ali H Mokdad, Lorenzo Monasta, Christopher J L Murray, Mohsen Naghavi, Samuel M Ostroff, Katherine R

Paulson, Antonio Luiz P Ribeiro, Roman Topor-Madry, Haidong Wang, Stefanie Watson, and Charles Shey Wiysonge.

Managing the overall research enterprise

Tahiya Alam, Joanne O Amlag, Sabina S Bloom, Kimberly Cooperrider, William James Dangel, Amanda Deen, Simon I Hay, Bethany Huntley, Ali H Mokdad, Christopher J L Murray, Mohsen Naghavi, Emma Elizabeth Spurlock, Roman Topor-Madry, and Haidong Wang.

Section 7. Supplemental Tables and Figures

Figure S1. Map of all cause data availability

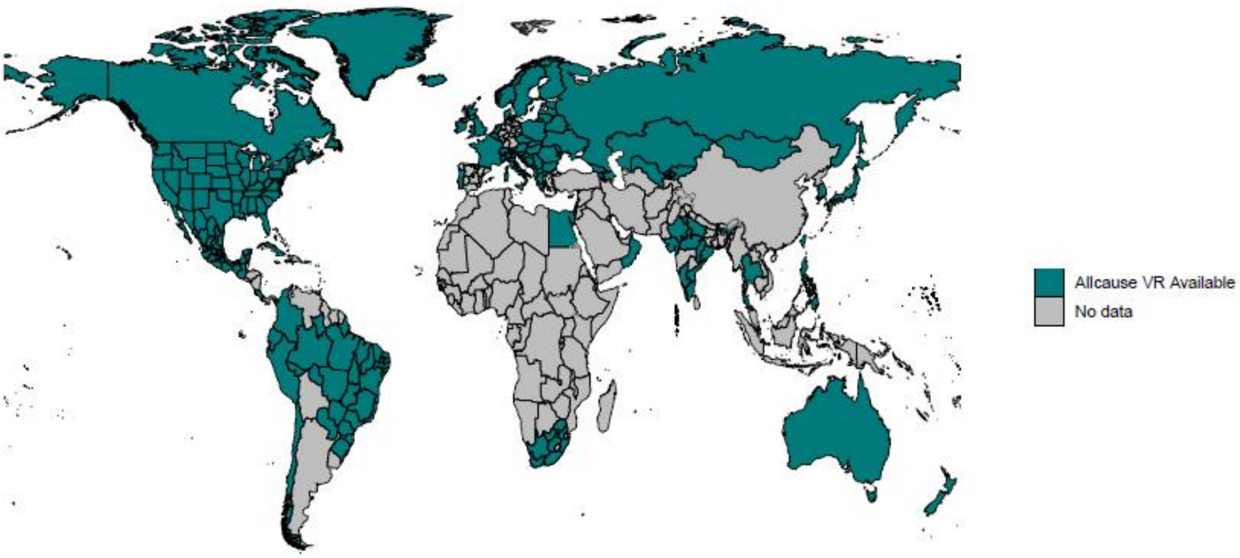

Figure S2. Map of reported Covid19 mortality data availability

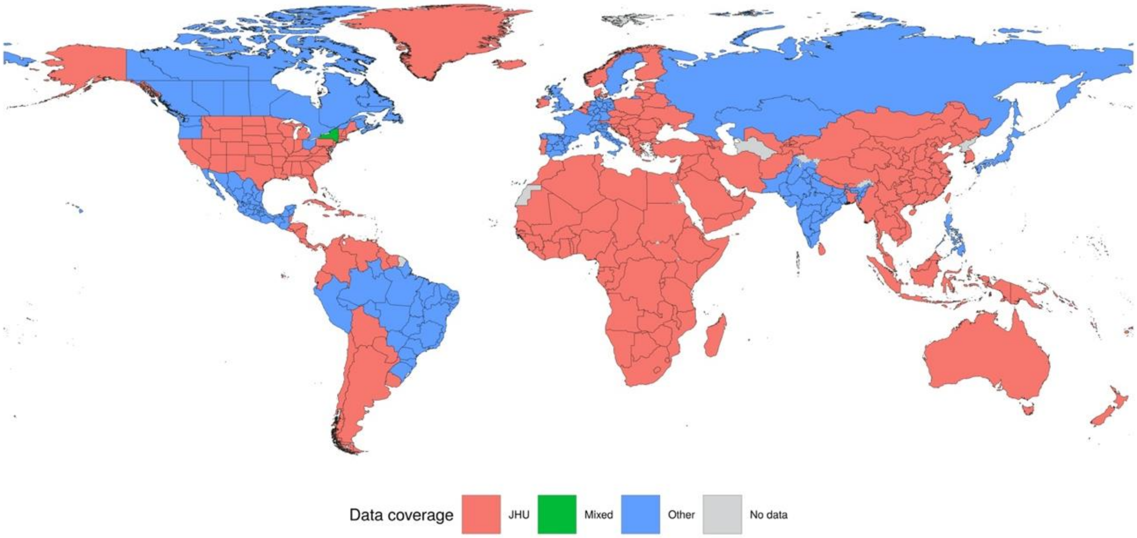

Figure S3. Estimating expected mortality based on seasonality and secular trends

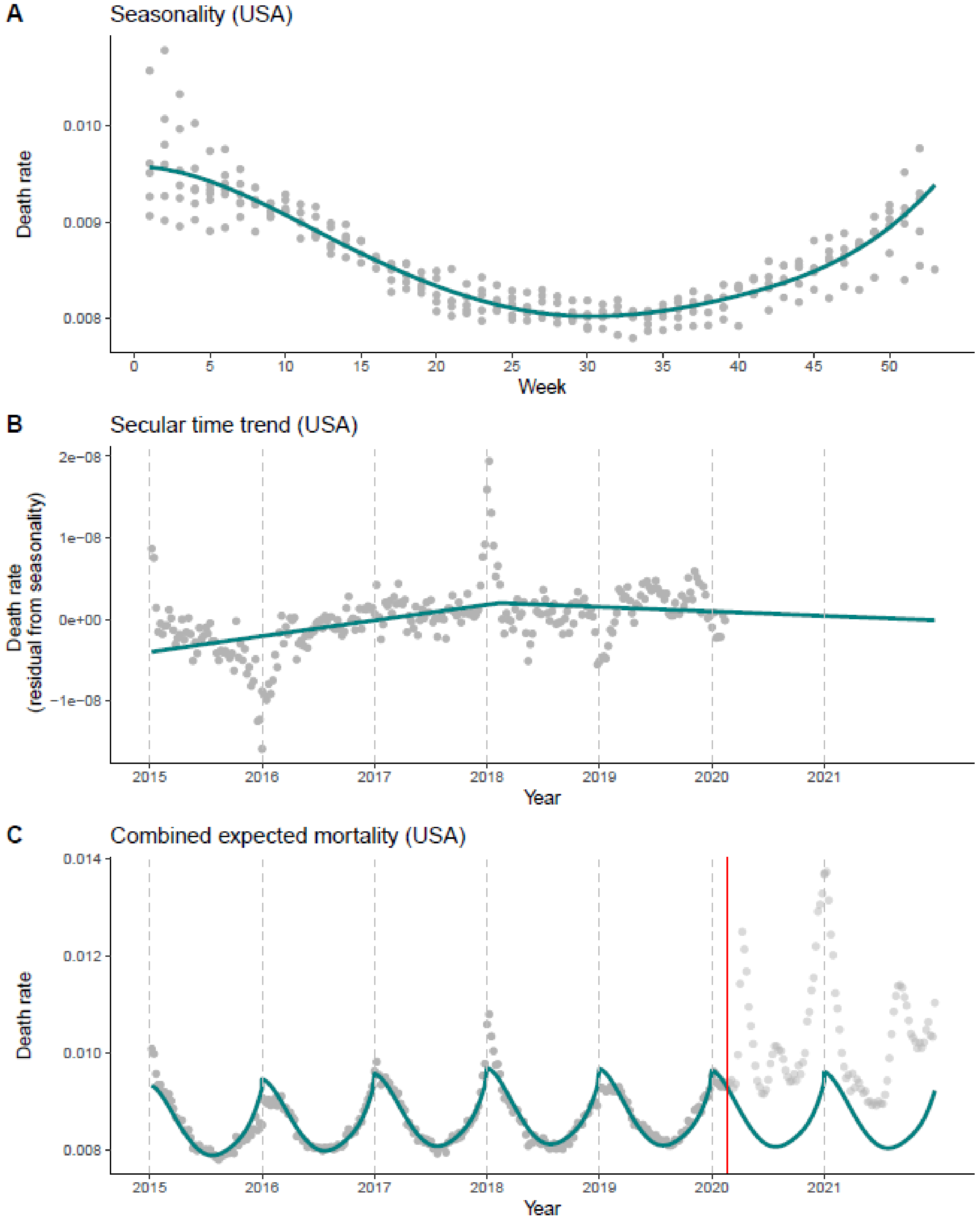

Figure S4. (a) The distribution of RMSE by location for each of six models included in the ensemble model, and (b) the estimated excess mortality for Spain for each component model and for the ensemble.

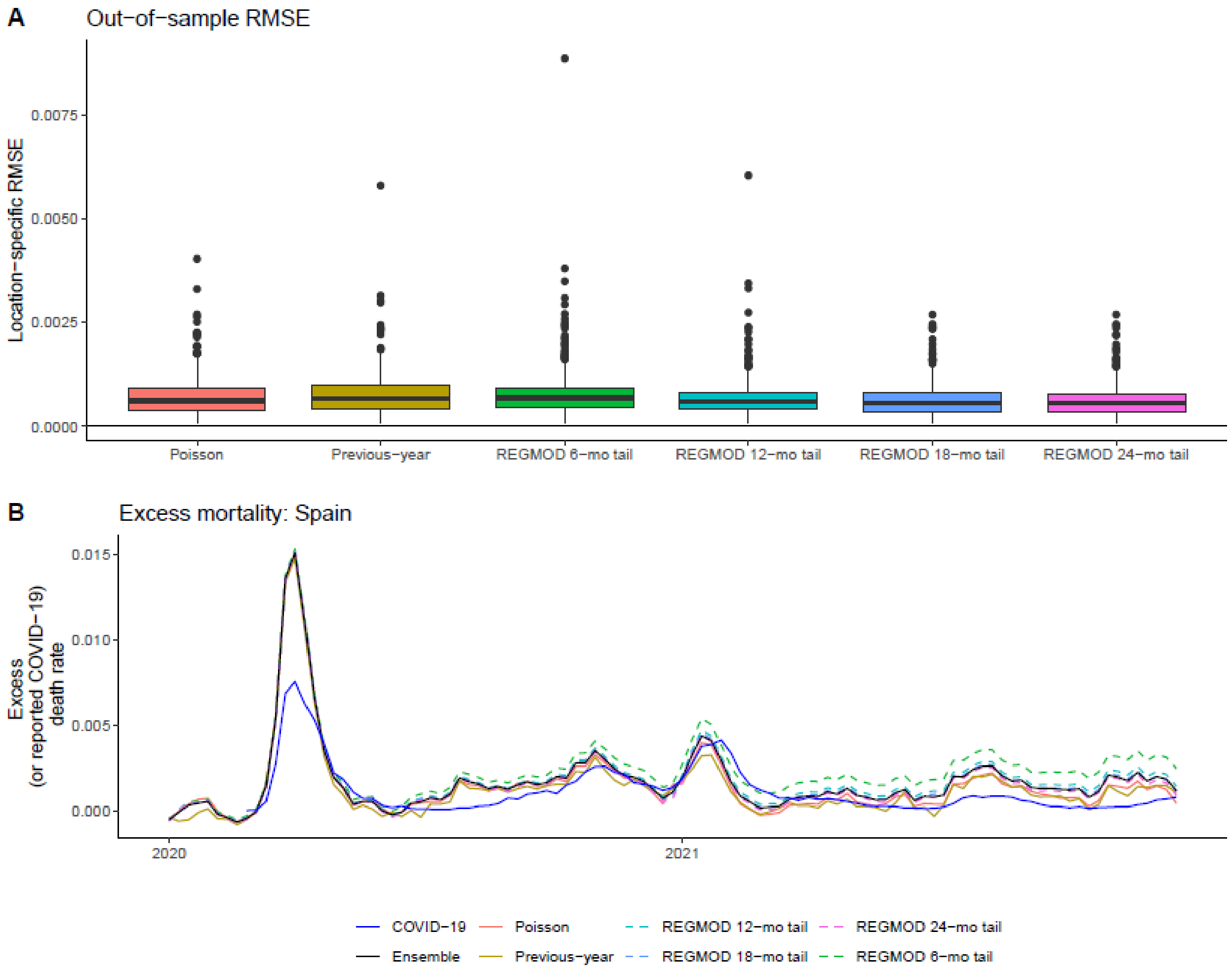

Figure S5. Comparison to *The Economist* excess mortality estimates

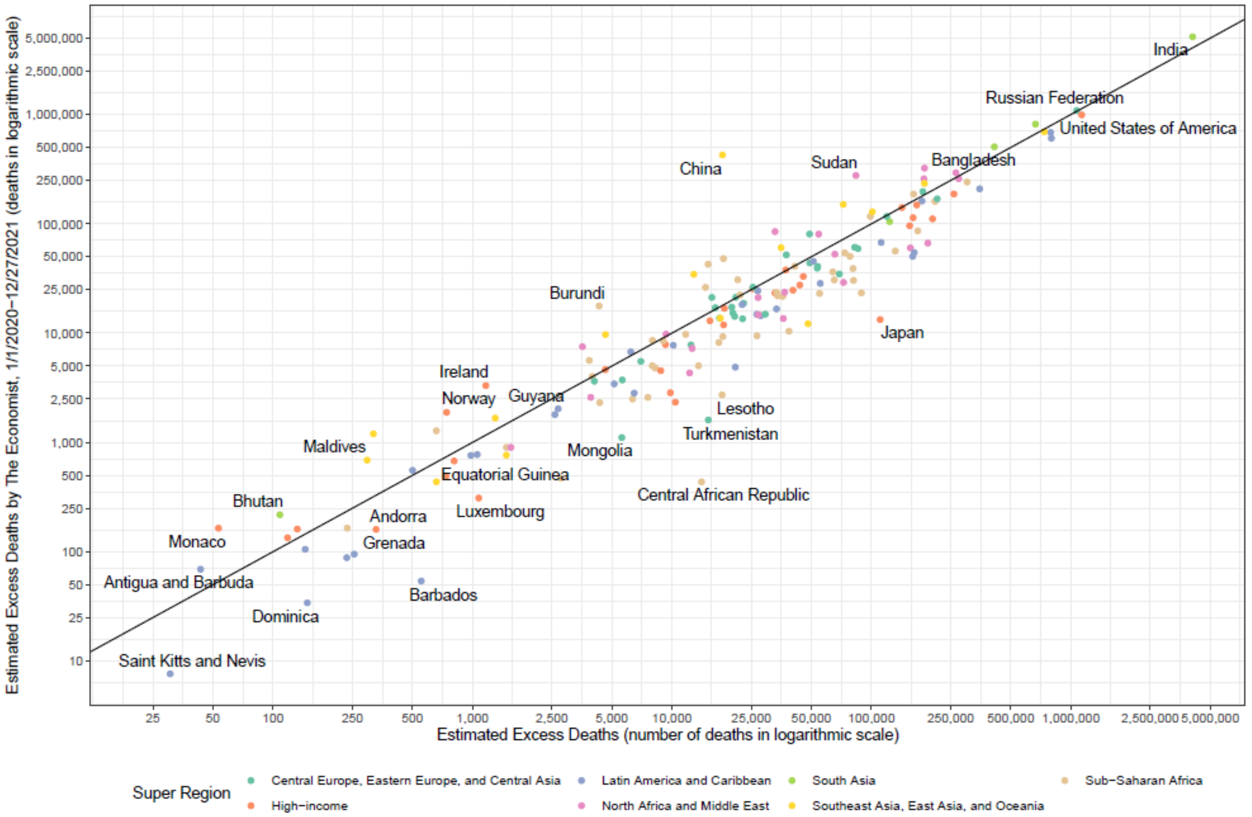

Figure S6. Late registration in all-cause deaths by countries and territories

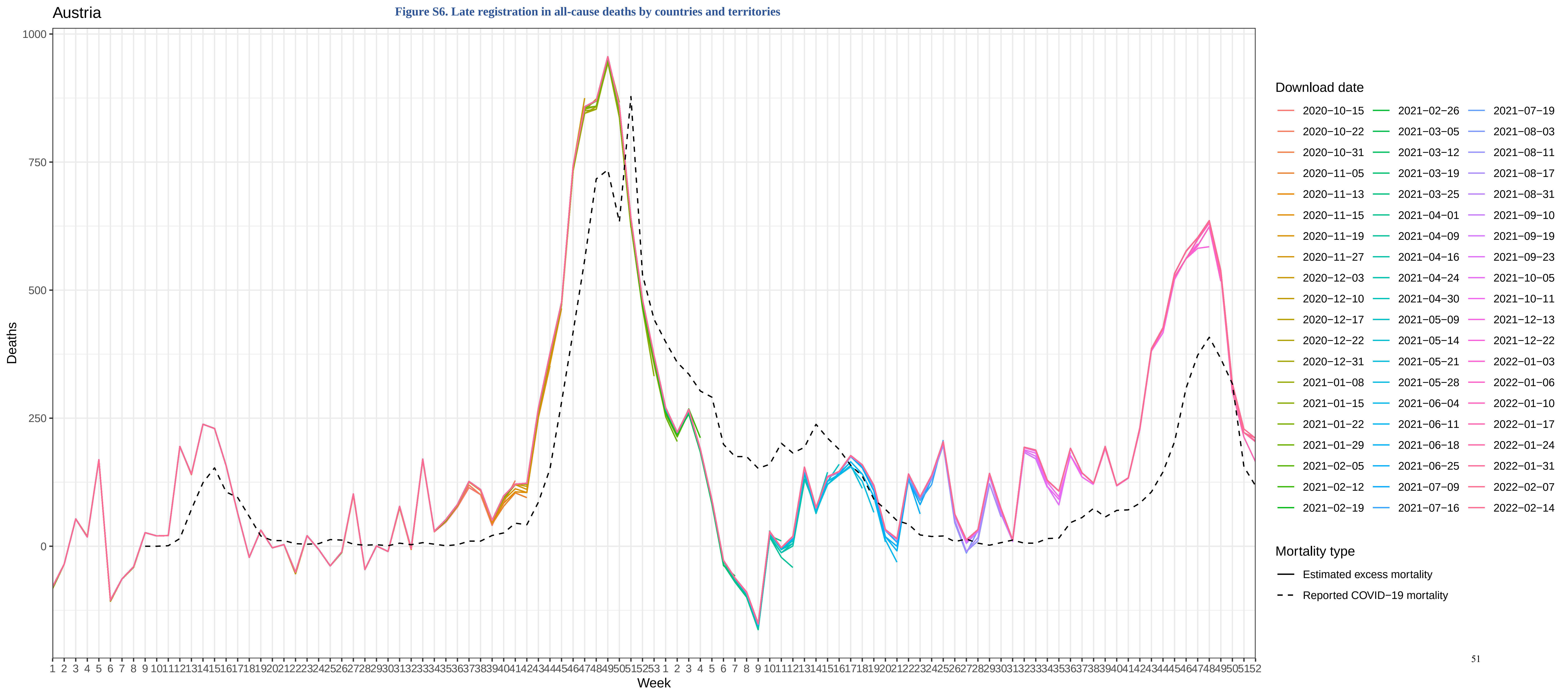

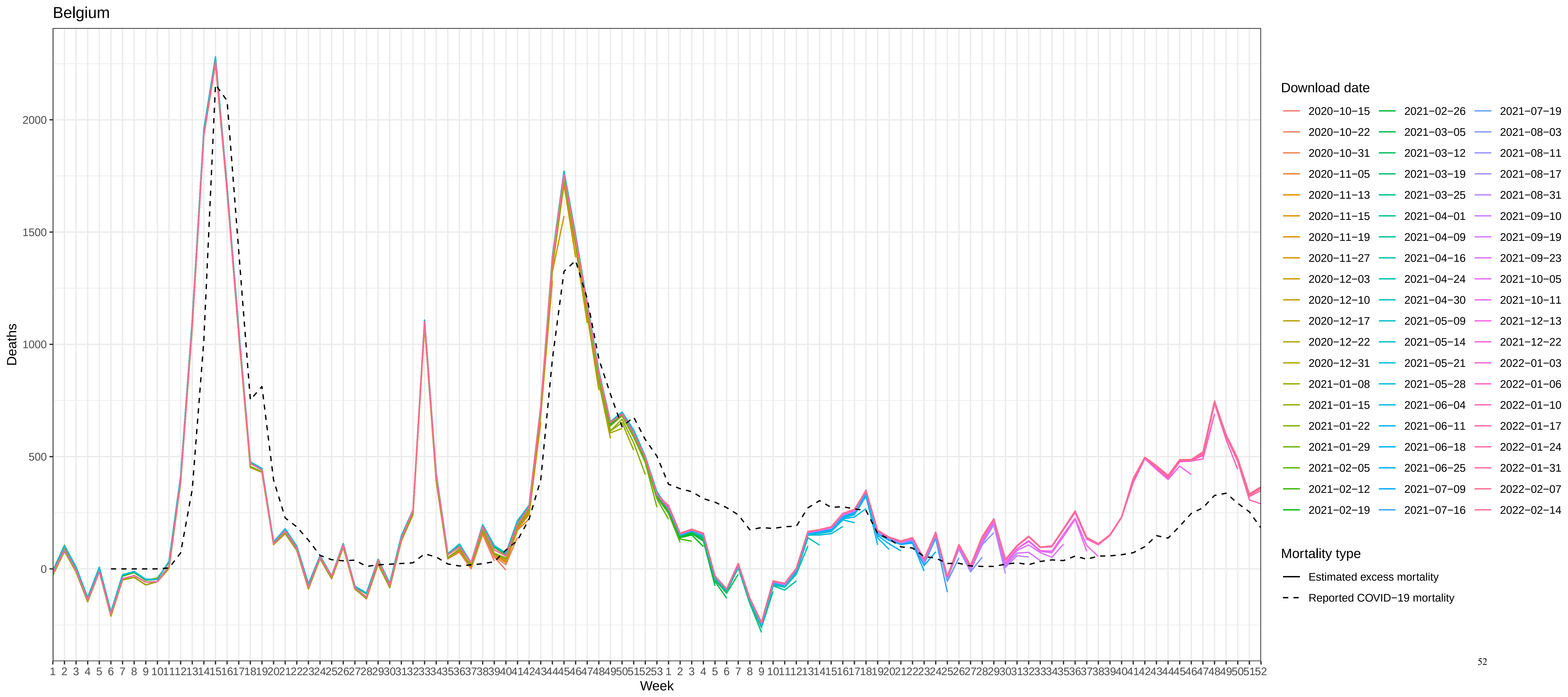

Belgium
Deaths
Week
Download date
2020−10−15
2020−10−22
2020−10−31
2020−11−05
2020−11−13
2020−11−15
2020−11−19
2020−11−27
2020−12−03
2020−12−10
2020−12−17
2020−12−22
2020−12−31
2021−01−08
2021−01−15
2021−01−22
2021−01−29
2021−02−05
2021−02−12
2021−02−19
2021−02−26
2021−03−05
2021−03−12
2021−03−19
2021−03−25
2021−04−01
2021−04−09
2021−04−16
2021−04−24
2021−04−30
2021−05−09
2021−05−14
2021−05−21
2021−05−28
2021−06−04
2021−06−11
2021−06−18
2021−06−25
2021−07−09
2021−07−16
2021−07−19
2021−08−03
2021−08−11
2021−08−17
2021−08−31
2021−09−10
2021−09−19
2021−09−23
2021−10−05
2021−10−11
2021−12−13
2021−12−22
2022−01−03
2022−01−06
2022−01−10
2022−01−17
2022−01−24
2022−01−31
2022−02−07
2022−02−14
Mortality type
Estimated excess mortality
Reported COVID−19 mortality

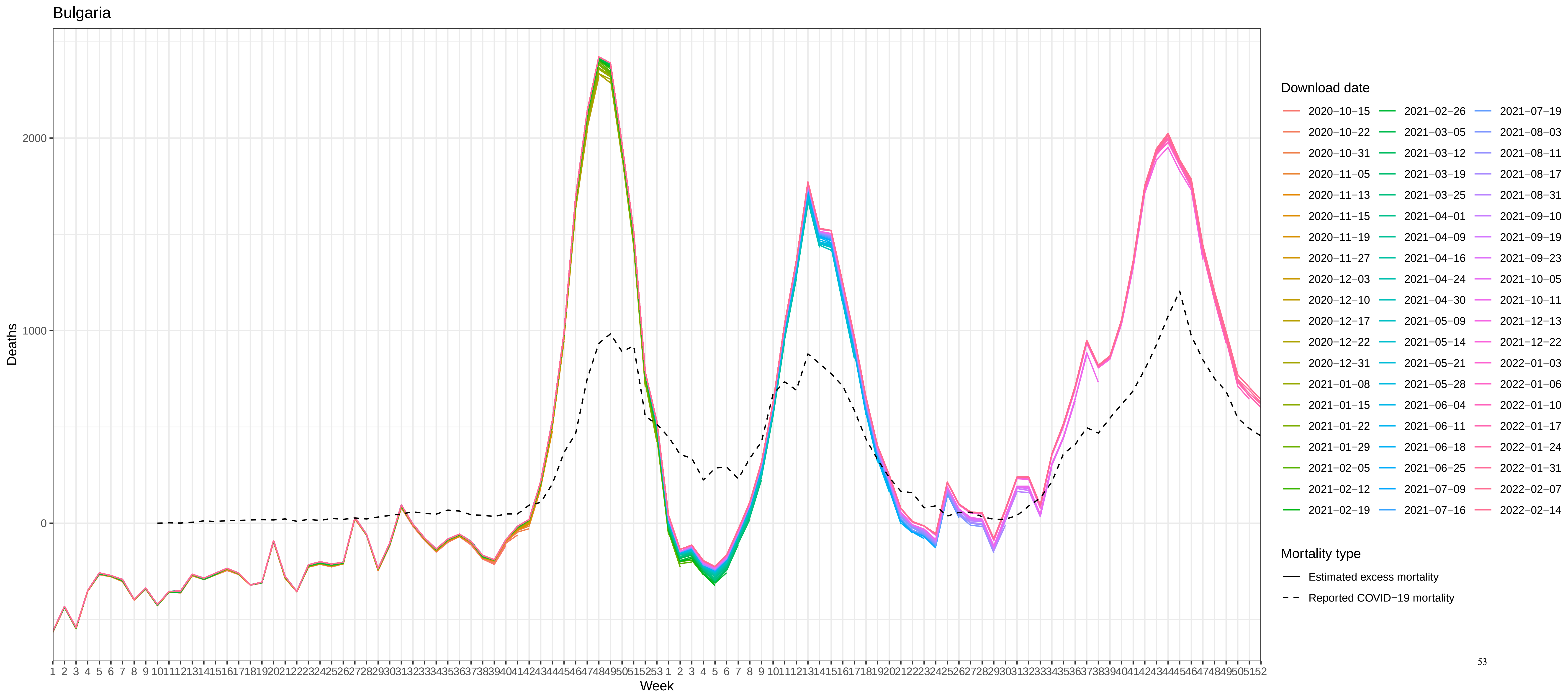
Bulgaria
Deaths
2000
1000
0
Week
1 2 3 4 5 6 7 8 9 10 11 12 13 14 15 16 17 18 19 20 21 22 23 24 25 26 27 28 29 30 31 32 33 34 35 36 37 38 39 40 41 42 43 44 45 46 47 48 49 50 51 52 53 1 2 3 4 5 6 7 8 9 10 11 12 13 14 15 16 17 18 19 20 21 22 23 24 25 26 27 28 29 30 31 32 33 34 35 36 37 38 39 40 41 42 43 44 45 46 47 48 49 50 51 52
Download date
2020−10−15
2020−10−22
2020−10−31
2020−11−05
2020−11−13
2020−11−15
2020−11−19
2020−11−27
2020−12−03
2020−12−10
2020−12−17
2020−12−22
2020−12−31
2021−01−08
2021−01−15
2021−01−22
2021−01−29
2021−02−05
2021−02−12
2021−02−19
2021−02−26
2021−03−05
2021−03−12
2021−03−19
2021−03−25
2021−04−01
2021−04−09
2021−04−16
2021−04−24
2021−04−30
2021−05−09
2021−05−14
2021−05−21
2021−05−28
2021−06−04
2021−06−11
2021−06−18
2021−06−25
2021−07−09
2021−07−16
2021−07−19
2021−08−03
2021−08−11
2021−08−17
2021−08−31
2021−09−10
2021−09−19
2021−09−23
2021−10−05
2021−10−11
2021−12−13
2021−12−22
2022−01−03
2022−01−06
2022−01−10
2022−01−17
2022−01−24
2022−01−31
2022−02−07
2022−02−14
Mortality type
Estimated excess mortality
Reported COVID−19 mortality

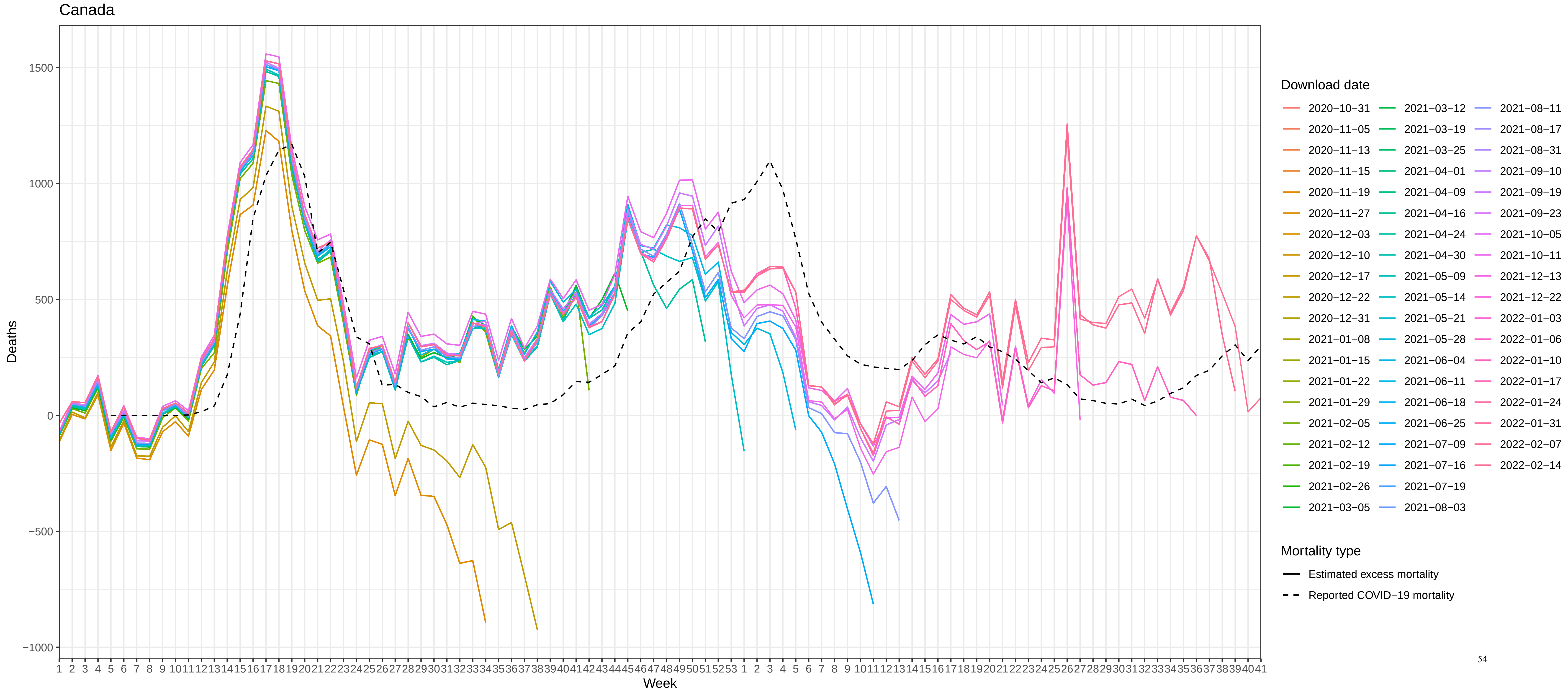

Canada
Deaths
Week
1500
1000
500
0
−500
−1000
Download date
2020−10−31
2020−11−05
2020−11−13
2020−11−15
2020−11−19
2020−11−27
2020−12−03
2020−12−10
2020−12−17
2020−12−22
2020−12−31
2021−01−08
2021−01−15
2021−01−22
2021−01−29
2021−02−05
2021−02−12
2021−02−19
2021−02−26
2021−03−05
2021−03−12
2021−03−19
2021−03−25
2021−04−01
2021−04−09
2021−04−16
2021−04−24
2021−04−30
2021−05−09
2021−05−14
2021−05−21
2021−05−28
2021−06−04
2021−06−11
2021−06−18
2021−06−25
2021−07−09
2021−07−16
2021−07−19
2021−08−03
2021−08−11
2021−08−17
2021−08−31
2021−09−10
2021−09−19
2021−09−23
2021−10−05
2021−10−11
2021−12−13
2021−12−22
2022−01−03
2022−01−06
2022−01−10
2022−01−17
2022−01−24
2022−01−31
2022−02−07
2022−02−14
Mortality type
Estimated excess mortality
Reported COVID−19 mortality

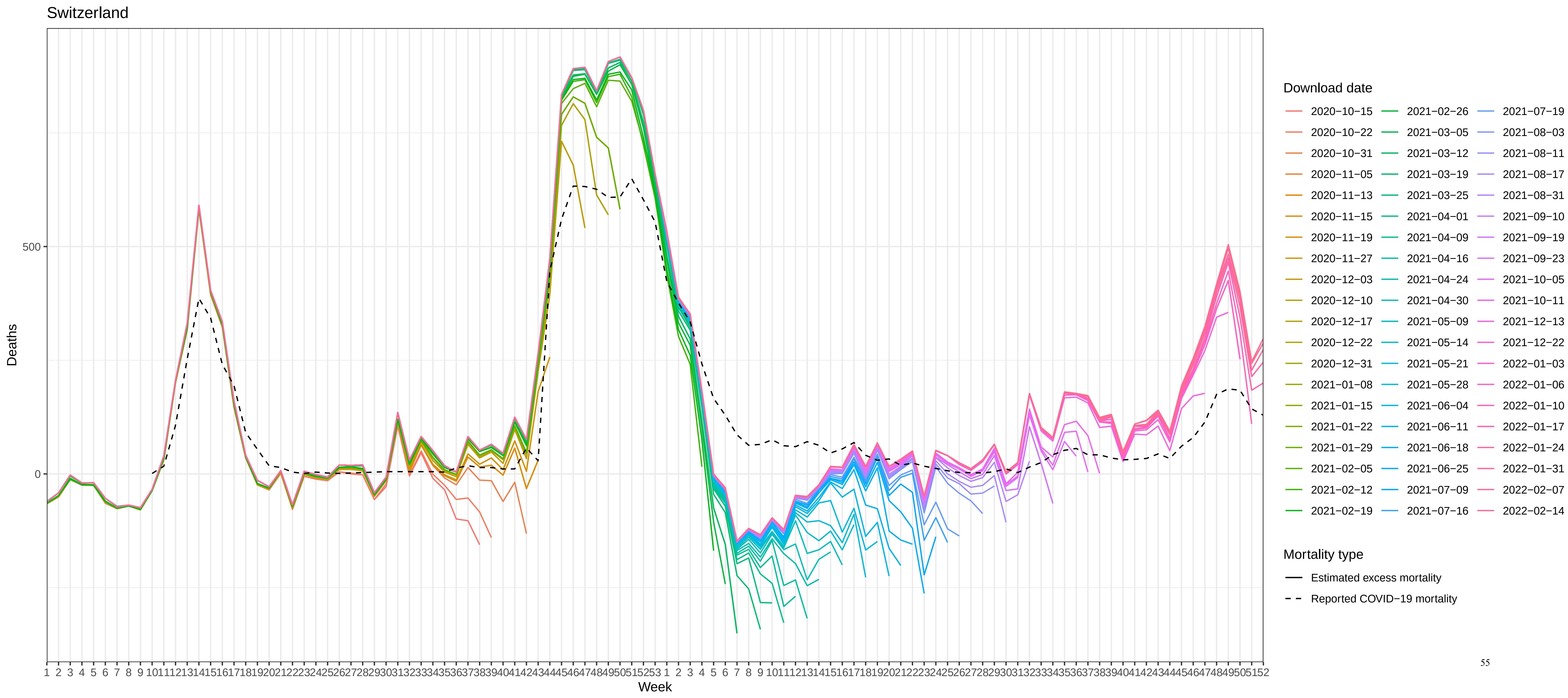
Switzerland
Deaths
500
0
Week
Download date
2020−10−15
2020−10−22
2020−10−31
2020−11−05
2020−11−13
2020−11−15
2020−11−19
2020−11−27
2020−12−03
2020−12−10
2020−12−17
2020−12−22
2020−12−31
2021−01−08
2021−01−15
2021−01−22
2021−01−29
2021−02−05
2021−02−12
2021−02−19
2021−02−26
2021−03−05
2021−03−12
2021−03−19
2021−03−25
2021−04−01
2021−04−09
2021−04−16
2021−04−24
2021−04−30
2021−05−09
2021−05−14
2021−05−21
2021−05−28
2021−06−04
2021−06−11
2021−06−18
2021−06−25
2021−07−09
2021−07−16
2021−07−19
2021−08−03
2021−08−11
2021−08−17
2021−08−31
2021−09−10
2021−09−19
2021−09−23
2021−10−05
2021−10−11
2021−12−13
2021−12−22
2022−01−03
2022−01−06
2022−01−10
2022−01−17
2022−01−24
2022−01−31
2022−02−07
2022−02−14
Mortality type
Estimated excess mortality
Reported COVID−19 mortality

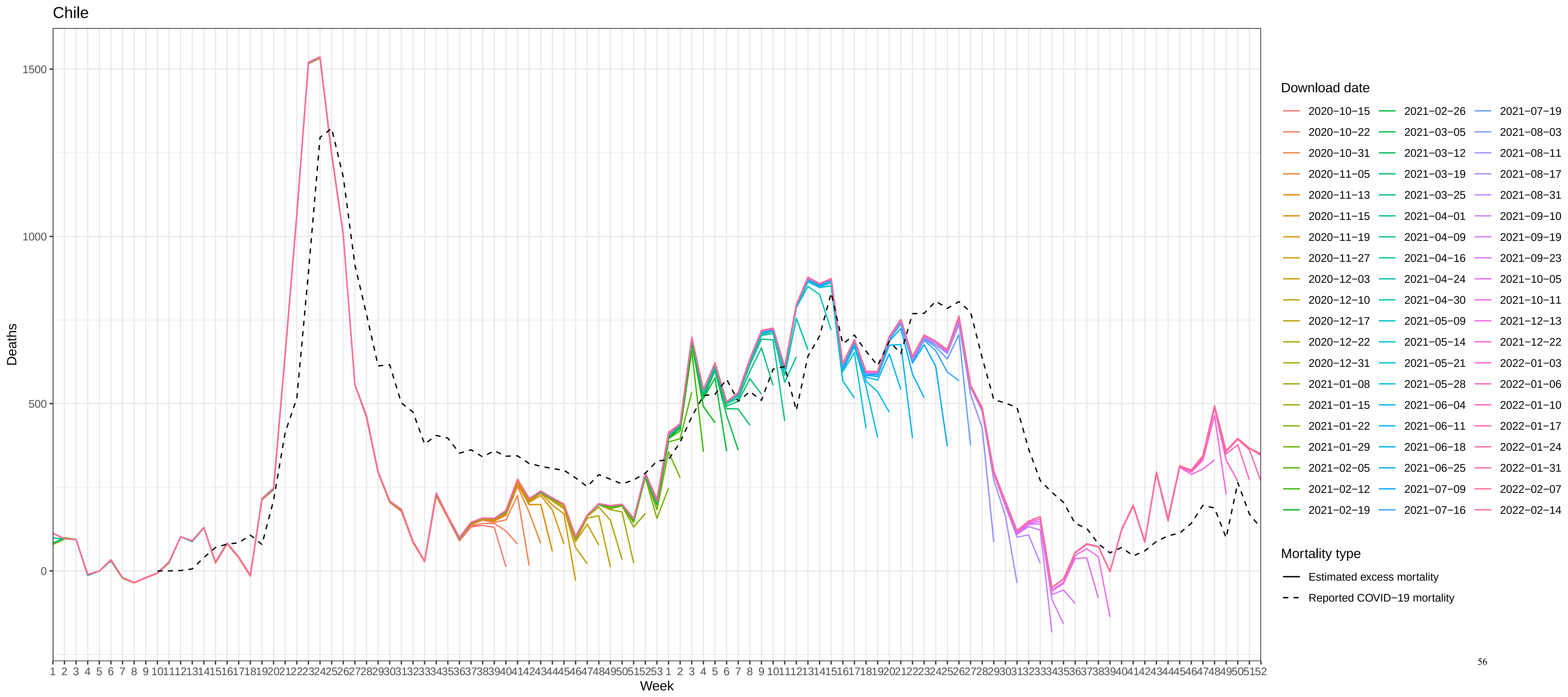
Chile
Deaths
1500
1000
500
0
Week
Download date
2020−10−15
2020−10−22
2020−10−31
2020−11−05
2020−11−13
2020−11−15
2020−11−19
2020−11−27
2020−12−03
2020−12−10
2020−12−17
2020−12−22
2020−12−31
2021−01−08
2021−01−15
2021−01−22
2021−01−29
2021−02−05
2021−02−12
2021−02−19
2021−02−26
2021−03−05
2021−03−12
2021−03−19
2021−03−25
2021−04−01
2021−04−09
2021−04−16
2021−04−24
2021−04−30
2021−05−09
2021−05−14
2021−05−21
2021−05−28
2021−06−04
2021−06−11
2021−06−18
2021−06−25
2021−07−09
2021−07−16
2021−07−19
2021−08−03
2021−08−11
2021−08−17
2021−08−31
2021−09−10
2021−09−19
2021−09−23
2021−10−05
2021−10−11
2021−12−13
2021−12−22
2022−01−03
2022−01−06
2022−01−10
2022−01−17
2022−01−24
2022−01−31
2022−02−07
2022−02−14
Mortality type
Estimated excess mortality
Reported COVID−19 mortality

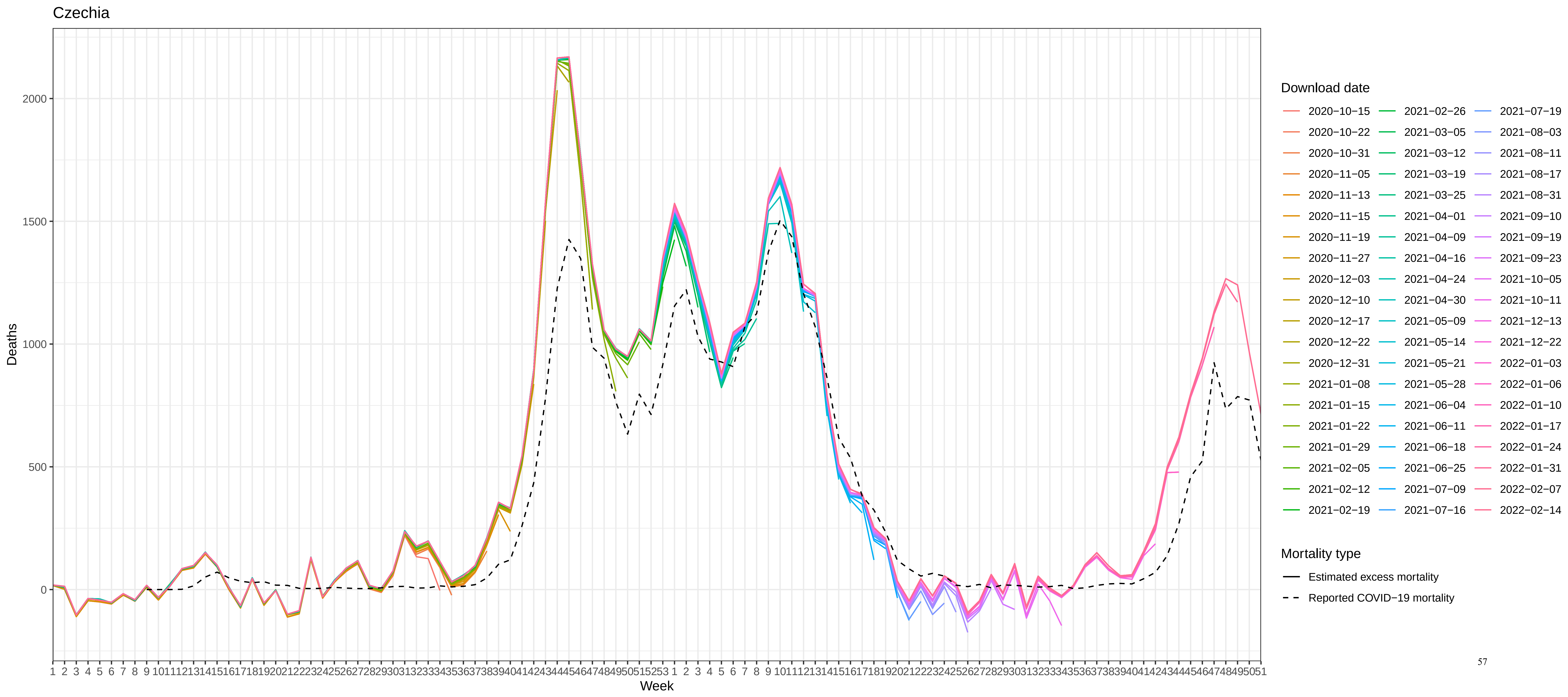
Czechia
Deaths
Week
Download date
2020−10−15
2020−10−22
2020−10−31
2020−11−05
2020−11−13
2020−11−15
2020−11−19
2020−11−27
2020−12−03
2020−12−10
2020−12−17
2020−12−22
2020−12−31
2021−01−08
2021−01−15
2021−01−22
2021−01−29
2021−02−05
2021−02−12
2021−02−19
2021−02−26
2021−03−05
2021−03−12
2021−03−19
2021−03−25
2021−04−01
2021−04−09
2021−04−16
2021−04−24
2021−04−30
2021−05−09
2021−05−14
2021−05−21
2021−05−28
2021−06−04
2021−06−11
2021−06−18
2021−06−25
2021−07−09
2021−07−16
2021−07−19
2021−08−03
2021−08−11
2021−08−17
2021−08−31
2021−09−10
2021−09−19
2021−09−23
2021−10−05
2021−10−11
2021−12−13
2021−12−22
2022−01−03
2022−01−06
2022−01−10
2022−01−17
2022−01−24
2022−01−31
2022−02−07
2022−02−14
Mortality type
Estimated excess mortality
Reported COVID−19 mortality

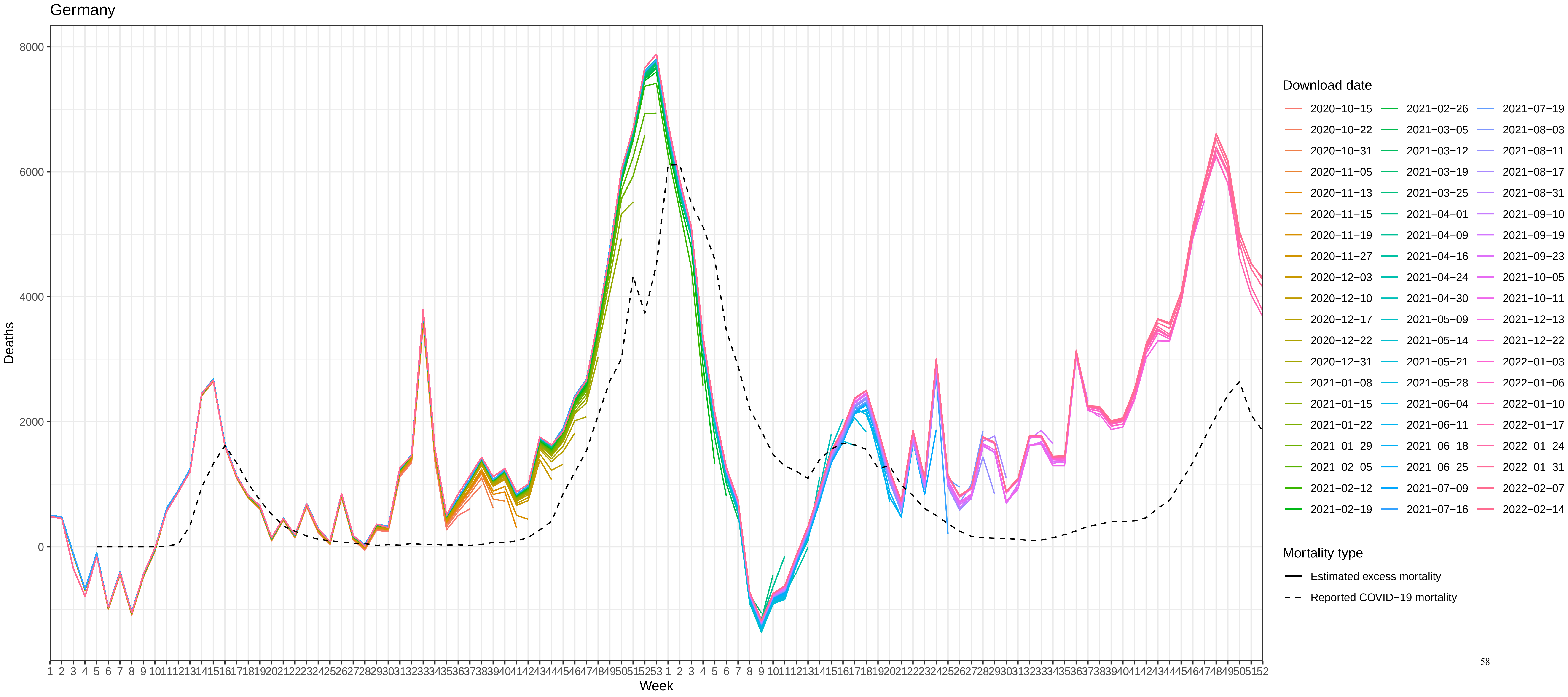
Germany
Deaths
Week
8000
6000
4000
2000
0
Download date
2020−10−15
2020−10−22
2020−10−31
2020−11−05
2020−11−13
2020−11−15
2020−11−19
2020−11−27
2020−12−03
2020−12−10
2020−12−17
2020−12−22
2020−12−31
2021−01−08
2021−01−15
2021−01−22
2021−01−29
2021−02−05
2021−02−12
2021−02−19
2021−02−26
2021−03−05
2021−03−12
2021−03−19
2021−03−25
2021−04−01
2021−04−09
2021−04−16
2021−04−24
2021−04−30
2021−05−09
2021−05−14
2021−05−21
2021−05−28
2021−06−04
2021−06−11
2021−06−18
2021−06−25
2021−07−09
2021−07−16
2021−07−19
2021−08−03
2021−08−11
2021−08−17
2021−08−31
2021−09−10
2021−09−19
2021−09−23
2021−10−05
2021−10−11
2021−12−13
2021−12−22
2022−01−03
2022−01−06
2022−01−10
2022−01−17
2022−01−24
2022−01−31
2022−02−07
2022−02−14
Mortality type
Estimated excess mortality
Reported COVID−19 mortality

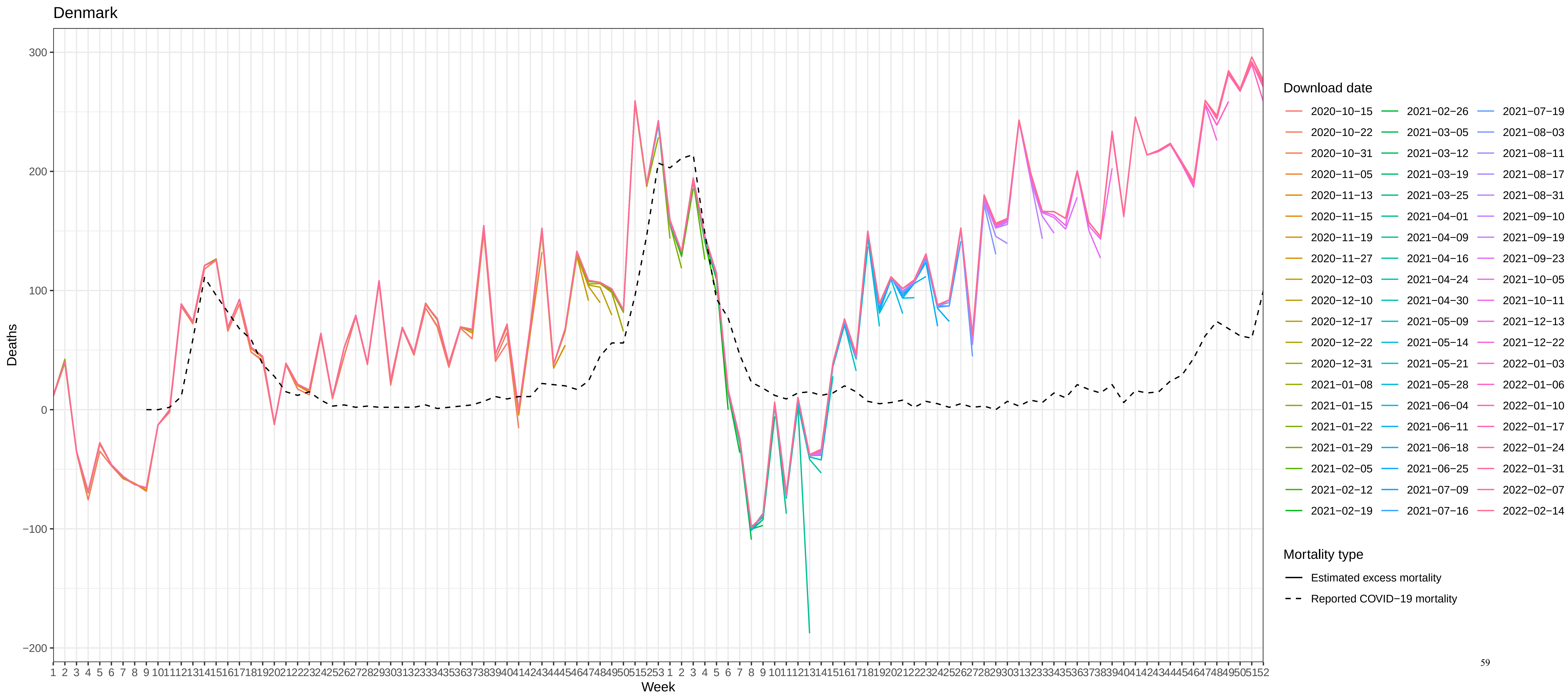

Denmark
Deaths
Week
Download date
Mortality type
Estimated excess mortality
Reported COVID−19 mortality

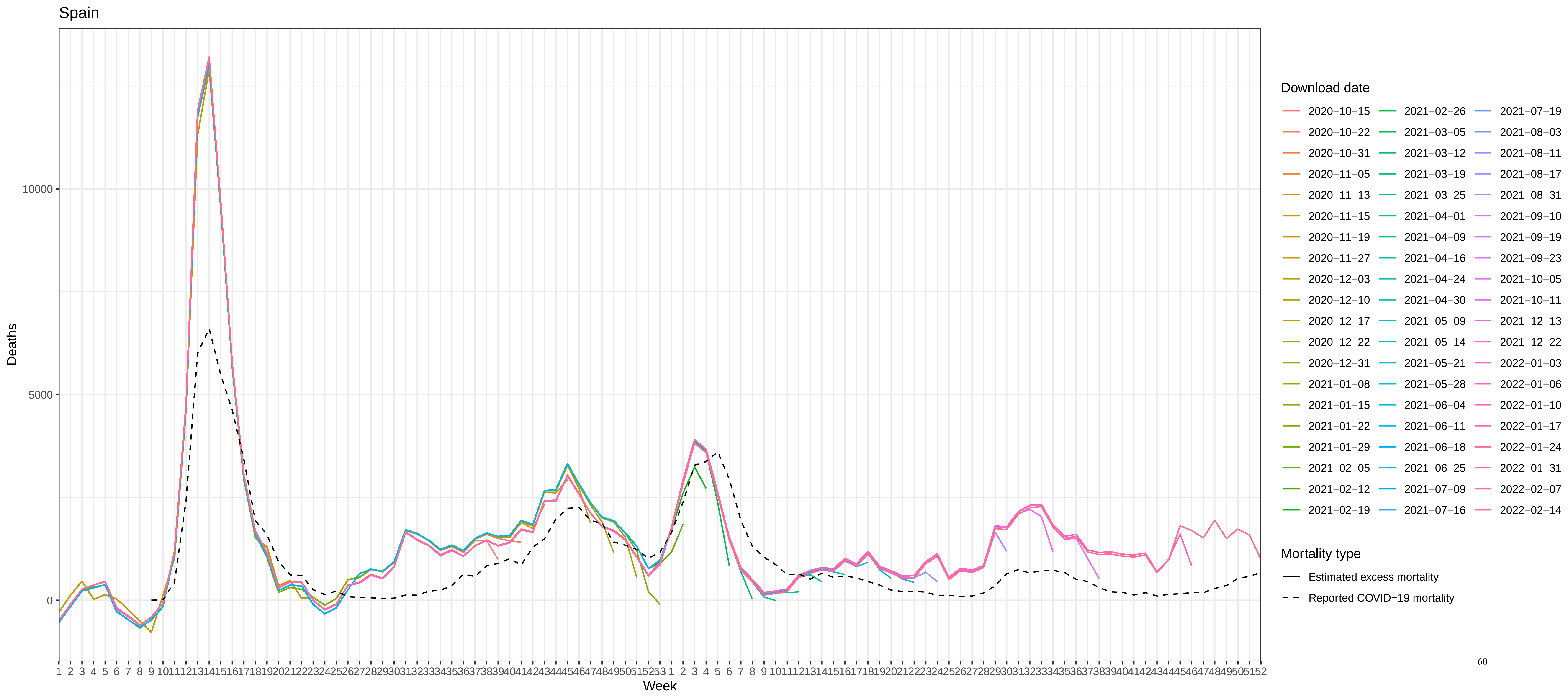
Spain
Deaths
10000
5000
0
Week
Download date
2020−10−15
2020−10−22
2020−10−31
2020−11−05
2020−11−13
2020−11−15
2020−11−19
2020−11−27
2020−12−03
2020−12−10
2020−12−17
2020−12−22
2020−12−31
2021−01−08
2021−01−15
2021−01−22
2021−01−29
2021−02−05
2021−02−12
2021−02−19
2021−02−26
2021−03−05
2021−03−12
2021−03−19
2021−03−25
2021−04−01
2021−04−09
2021−04−16
2021−04−24
2021−04−30
2021−05−09
2021−05−14
2021−05−21
2021−05−28
2021−06−04
2021−06−11
2021−06−18
2021−06−25
2021−07−09
2021−07−16
2021−07−19
2021−08−03
2021−08−11
2021−08−17
2021−08−31
2021−09−10
2021−09−19
2021−09−23
2021−10−05
2021−10−11
2021−12−13
2021−12−22
2022−01−03
2022−01−06
2022−01−10
2022−01−17
2022−01−24
2022−01−31
2022−02−07
2022−02−14
Mortality type
Estimated excess mortality
Reported COVID−19 mortality

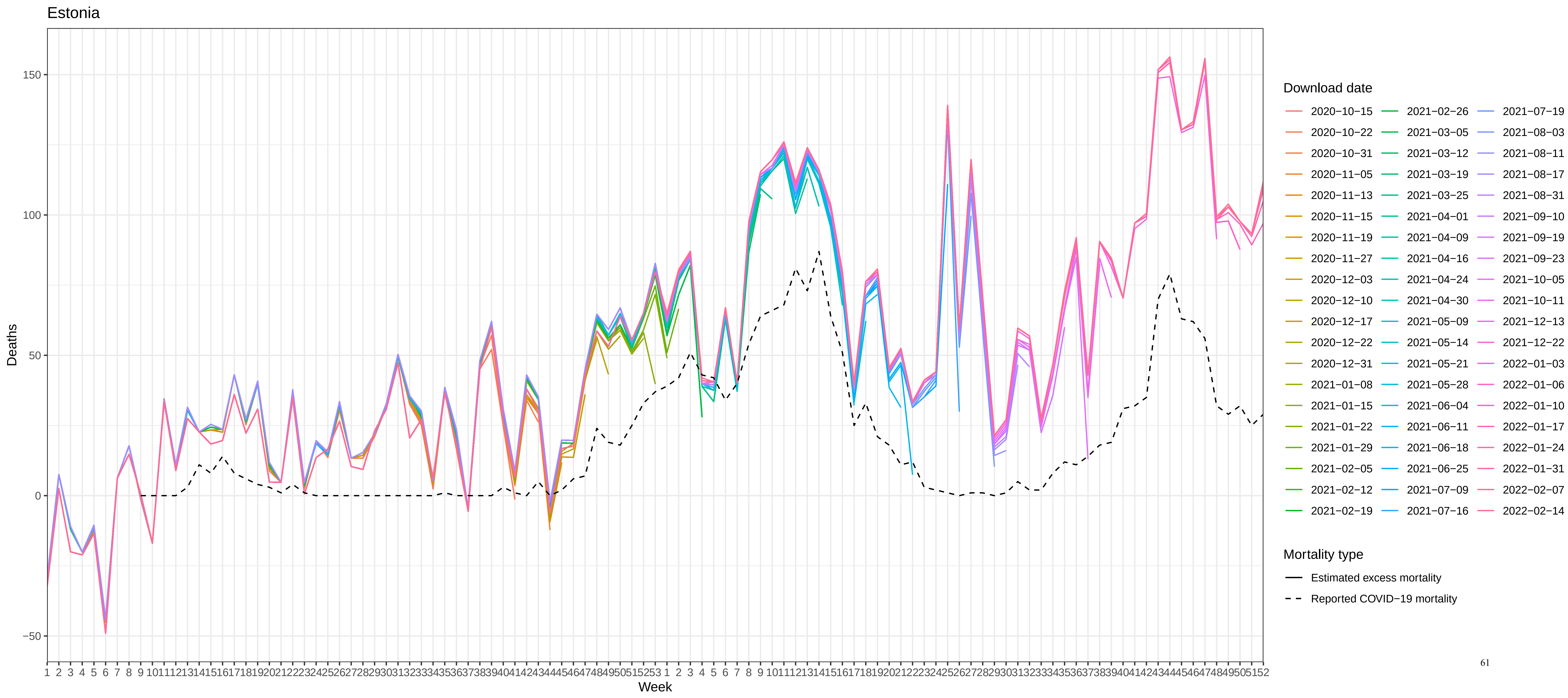
Estonia
Deaths
Week
Download date
2020−10−15
2020−10−22
2020−10−31
2020−11−05
2020−11−13
2020−11−15
2020−11−19
2020−11−27
2020−12−03
2020−12−10
2020−12−17
2020−12−22
2020−12−31
2021−01−08
2021−01−15
2021−01−22
2021−01−29
2021−02−05
2021−02−12
2021−02−19
2021−02−26
2021−03−05
2021−03−12
2021−03−19
2021−03−25
2021−04−01
2021−04−09
2021−04−16
2021−04−24
2021−04−30
2021−05−09
2021−05−14
2021−05−21
2021−05−28
2021−06−04
2021−06−11
2021−06−18
2021−06−25
2021−07−09
2021−07−16
2021−07−19
2021−08−03
2021−08−11
2021−08−17
2021−08−31
2021−09−10
2021−09−19
2021−09−23
2021−10−05
2021−10−11
2021−12−13
2021−12−22
2022−01−03
2022−01−06
2022−01−10
2022−01−17
2022−01−24
2022−01−31
2022−02−07
2022−02−14
Mortality type
Estimated excess mortality
Reported COVID−19 mortality

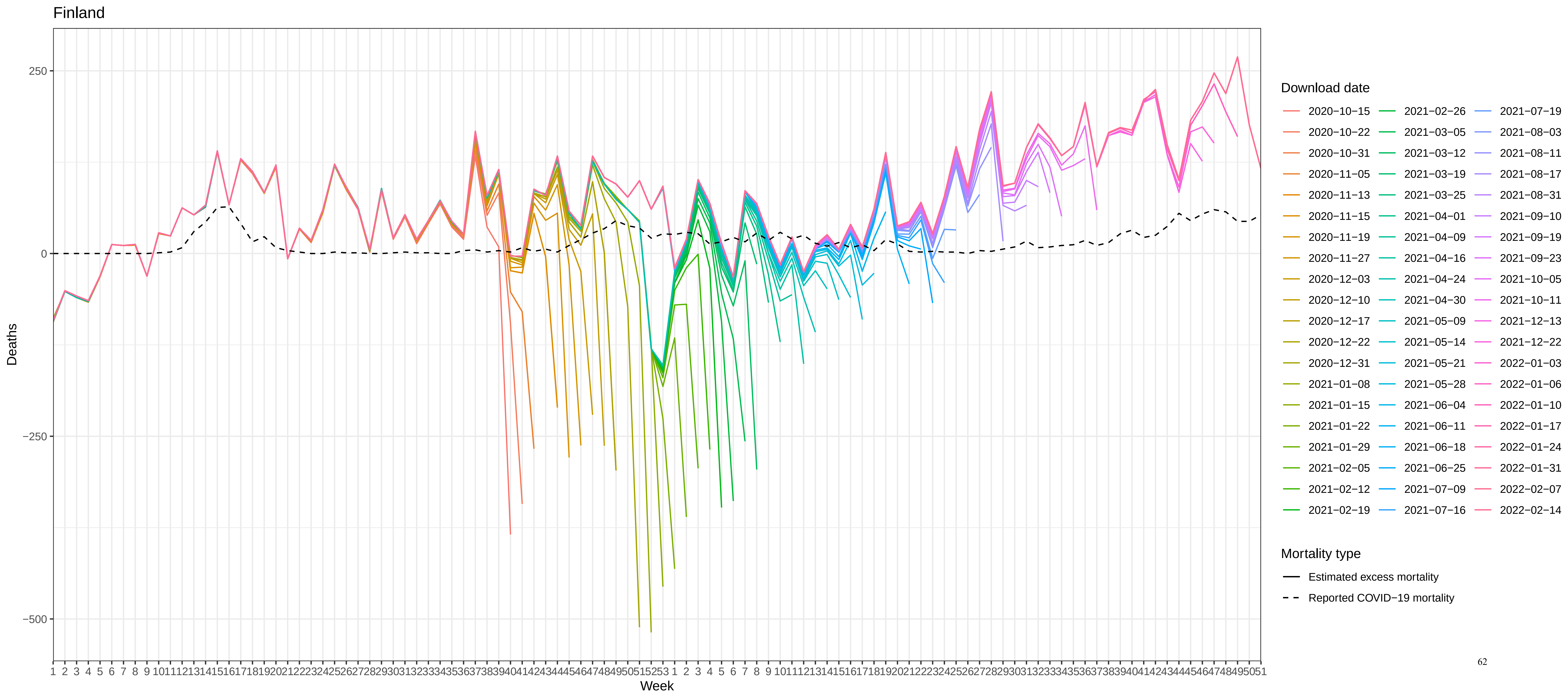
Finland
250
0
−250
−500
Deaths
Week
Download date
2020−10−15
2020−10−22
2020−10−31
2020−11−05
2020−11−13
2020−11−15
2020−11−19
2020−11−27
2020−12−03
2020−12−10
2020−12−17
2020−12−22
2020−12−31
2021−01−08
2021−01−15
2021−01−22
2021−01−29
2021−02−05
2021−02−12
2021−02−19
2021−02−26
2021−03−05
2021−03−12
2021−03−19
2021−03−25
2021−04−01
2021−04−09
2021−04−16
2021−04−24
2021−04−30
2021−05−09
2021−05−14
2021−05−21
2021−05−28
2021−06−04
2021−06−11
2021−06−18
2021−06−25
2021−07−09
2021−07−16
2021−07−19
2021−08−03
2021−08−11
2021−08−17
2021−08−31
2021−09−10
2021−09−19
2021−09−23
2021−10−05
2021−10−11
2021−12−13
2021−12−22
2022−01−03
2022−01−06
2022−01−10
2022−01−17
2022−01−24
2022−01−31
2022−02−07
2022−02−14
Mortality type
Estimated excess mortality
Reported COVID−19 mortality

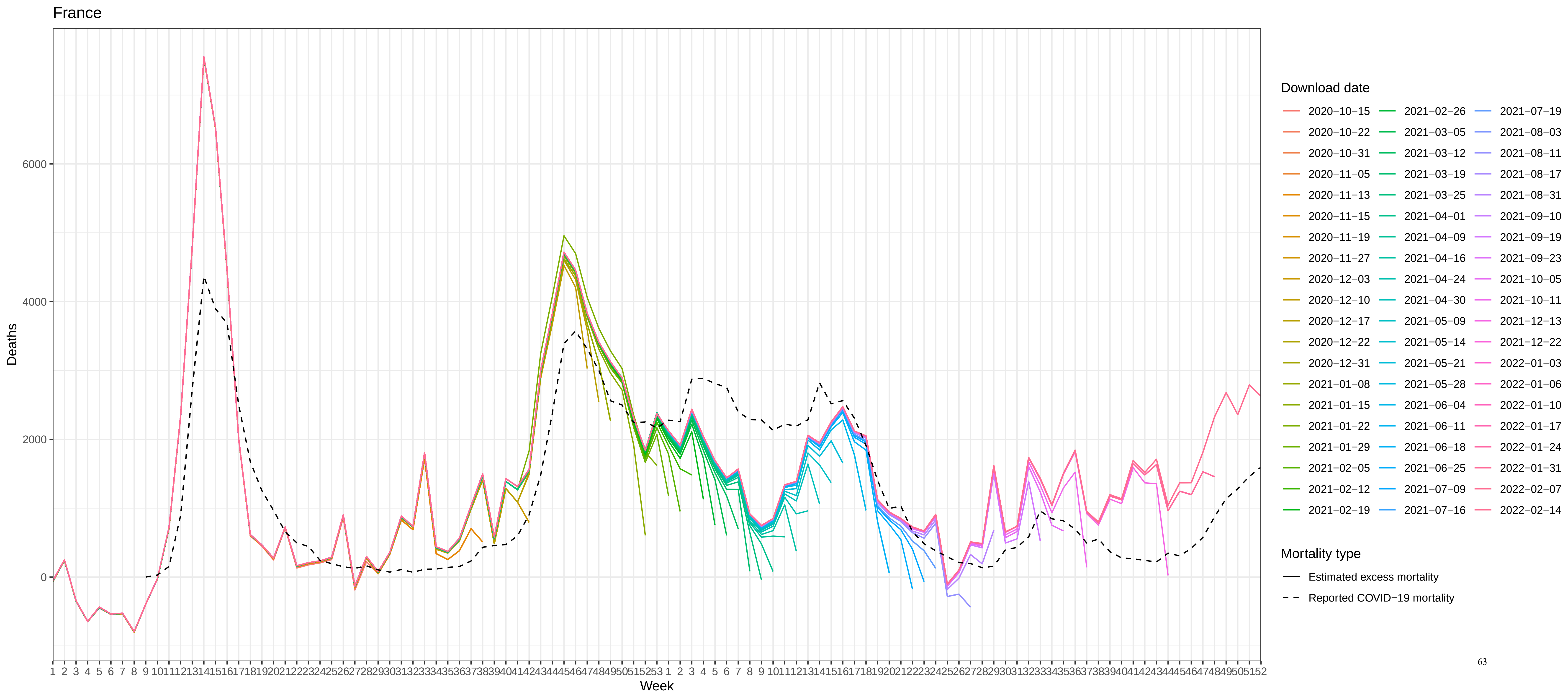
France
Deaths
Week
6000
4000
2000
0
Download date
2020−10−15
2020−10−22
2020−10−31
2020−11−05
2020−11−13
2020−11−15
2020−11−19
2020−11−27
2020−12−03
2020−12−10
2020−12−17
2020−12−22
2020−12−31
2021−01−08
2021−01−15
2021−01−22
2021−01−29
2021−02−05
2021−02−12
2021−02−19
2021−02−26
2021−03−05
2021−03−12
2021−03−19
2021−03−25
2021−04−01
2021−04−09
2021−04−16
2021−04−24
2021−04−30
2021−05−09
2021−05−14
2021−05−21
2021−05−28
2021−06−04
2021−06−11
2021−06−18
2021−06−25
2021−07−09
2021−07−16
2021−07−19
2021−08−03
2021−08−11
2021−08−17
2021−08−31
2021−09−10
2021−09−19
2021−09−23
2021−10−05
2021−10−11
2021−12−13
2021−12−22
2022−01−03
2022−01−06
2022−01−10
2022−01−17
2022−01−24
2022−01−31
2022−02−07
2022−02−14
Mortality type
Estimated excess mortality
Reported COVID−19 mortality

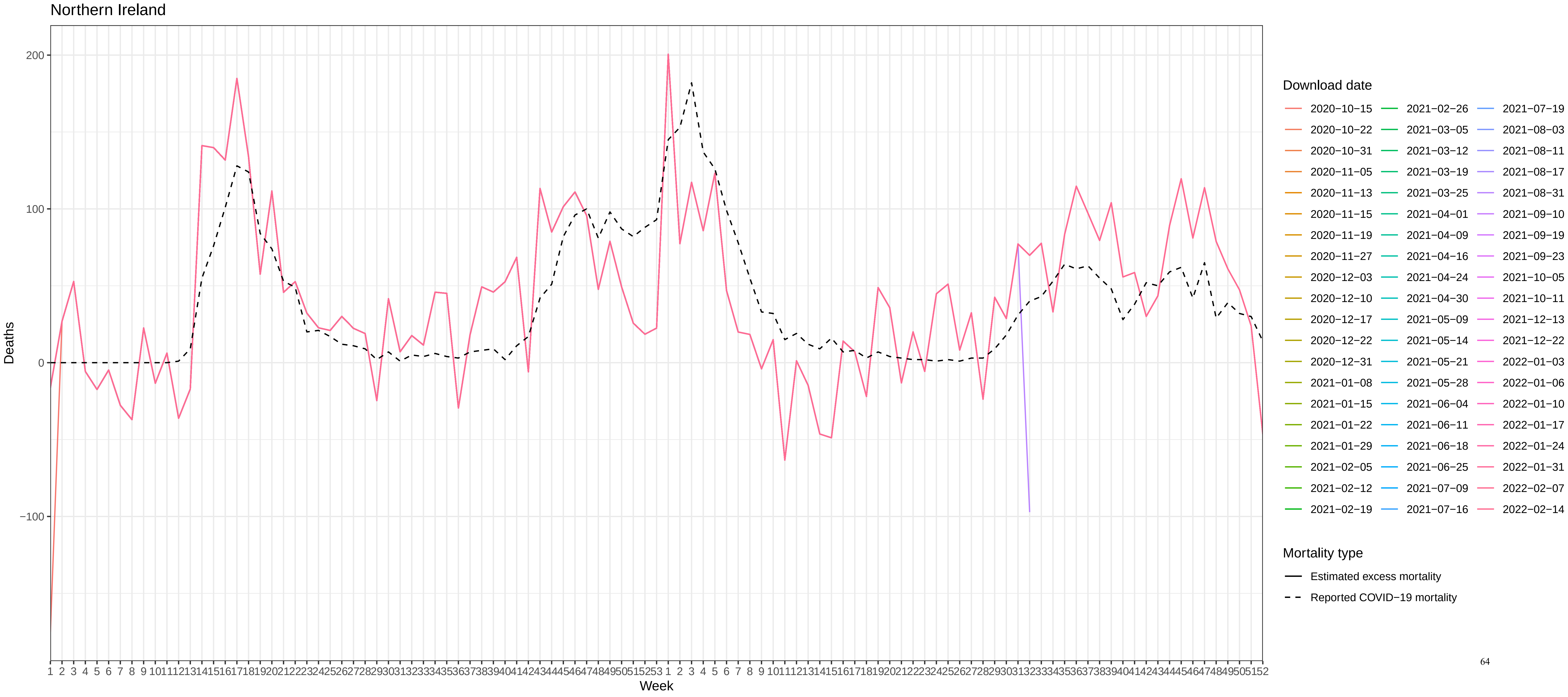
Northern Ireland
Deaths
Week
Download date
2020−10−15
2020−10−22
2020−10−31
2020−11−05
2020−11−13
2020−11−15
2020−11−19
2020−11−27
2020−12−03
2020−12−10
2020−12−17
2020−12−22
2020−12−31
2021−01−08
2021−01−15
2021−01−22
2021−01−29
2021−02−05
2021−02−12
2021−02−19
2021−02−26
2021−03−05
2021−03−12
2021−03−19
2021−03−25
2021−04−01
2021−04−09
2021−04−16
2021−04−24
2021−04−30
2021−05−09
2021−05−14
2021−05−21
2021−05−28
2021−06−04
2021−06−11
2021−06−18
2021−06−25
2021−07−09
2021−07−16
2021−07−19
2021−08−03
2021−08−11
2021−08−17
2021−08−31
2021−09−10
2021−09−19
2021−09−23
2021−10−05
2021−10−11
2021−12−13
2021−12−22
2022−01−03
2022−01−06
2022−01−10
2022−01−17
2022−01−24
2022−01−31
2022−02−07
2022−02−14
Mortality type
Estimated excess mortality
Reported COVID−19 mortality

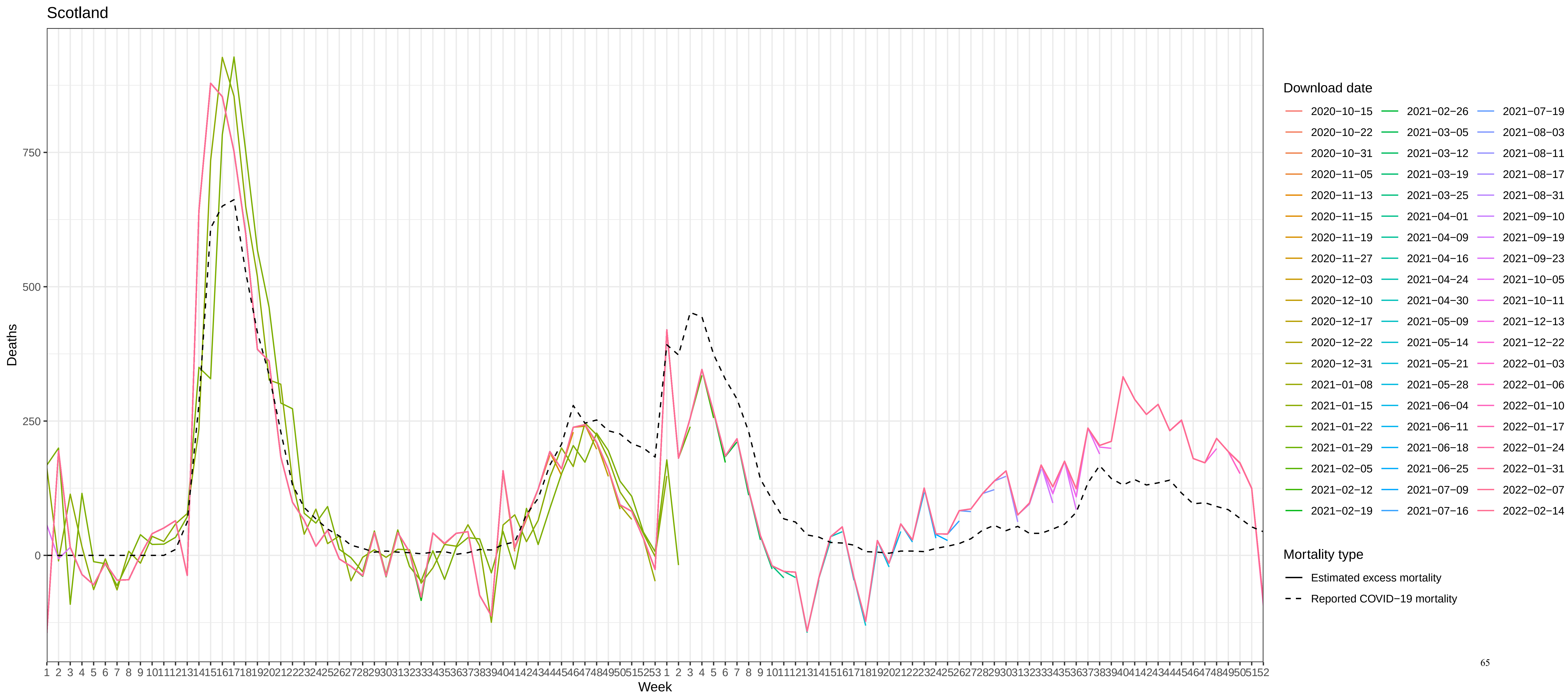

Scotland
Deaths
750
500
250
0
Week
1 2 3 4 5 6 7 8 9 10 11 12 13 14 15 16 17 18 19 20 21 22 23 24 25 26 27 28 29 30 31 32 33 34 35 36 37 38 39 40 41 42 43 44 45 46 47 48 49 50 51 52 53 1 2 3 4 5 6 7 8 9 10 11 12 13 14 15 16 17 18 19 20 21 22 23 24 25 26 27 28 29 30 31 32 33 34 35 36 37 38 39 40 41 42 43 44 45 46 47 48 49 50 51 52
Download date
2020−10−15
2020−10−22
2020−10−31
2020−11−05
2020−11−13
2020−11−15
2020−11−19
2020−11−27
2020−12−03
2020−12−10
2020−12−17
2020−12−22
2020−12−31
2021−01−08
2021−01−15
2021−01−22
2021−01−29
2021−02−05
2021−02−12
2021−02−19
2021−02−26
2021−03−05
2021−03−12
2021−03−19
2021−03−25
2021−04−01
2021−04−09
2021−04−16
2021−04−24
2021−04−30
2021−05−09
2021−05−14
2021−05−21
2021−05−28
2021−06−04
2021−06−11
2021−06−18
2021−06−25
2021−07−09
2021−07−16
2021−07−19
2021−08−03
2021−08−11
2021−08−17
2021−08−31
2021−09−10
2021−09−19
2021−09−23
2021−10−05
2021−10−11
2021−12−13
2021−12−22
2022−01−03
2022−01−06
2022−01−10
2022−01−17
2022−01−24
2022−01−31
2022−02−07
2022−02−14
Mortality type
Estimated excess mortality
Reported COVID−19 mortality

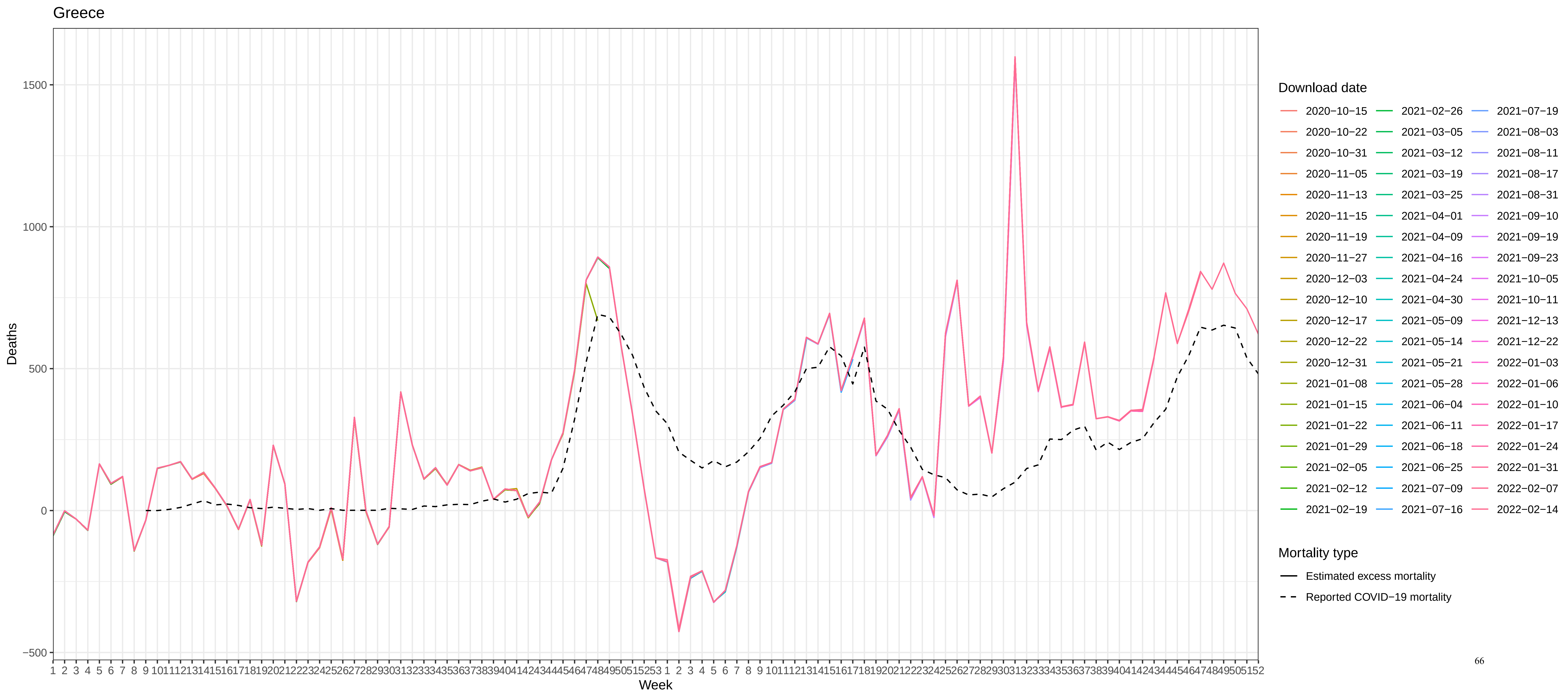

Greece
Deaths
1500
1000
500
0
−500
Week
Download date
2020−10−15
2020−10−22
2020−10−31
2020−11−05
2020−11−13
2020−11−15
2020−11−19
2020−11−27
2020−12−03
2020−12−10
2020−12−17
2020−12−22
2020−12−31
2021−01−08
2021−01−15
2021−01−22
2021−01−29
2021−02−05
2021−02−12
2021−02−19
2021−02−26
2021−03−05
2021−03−12
2021−03−19
2021−03−25
2021−04−01
2021−04−09
2021−04−16
2021−04−24
2021−04−30
2021−05−09
2021−05−14
2021−05−21
2021−05−28
2021−06−04
2021−06−11
2021−06−18
2021−06−25
2021−07−09
2021−07−16
2021−07−19
2021−08−03
2021−08−11
2021−08−17
2021−08−31
2021−09−10
2021−09−19
2021−09−23
2021−10−05
2021−10−11
2021−12−13
2021−12−22
2022−01−03
2022−01−06
2022−01−10
2022−01−17
2022−01−24
2022−01−31
2022−02−07
2022−02−14
Mortality type
Estimated excess mortality
Reported COVID−19 mortality

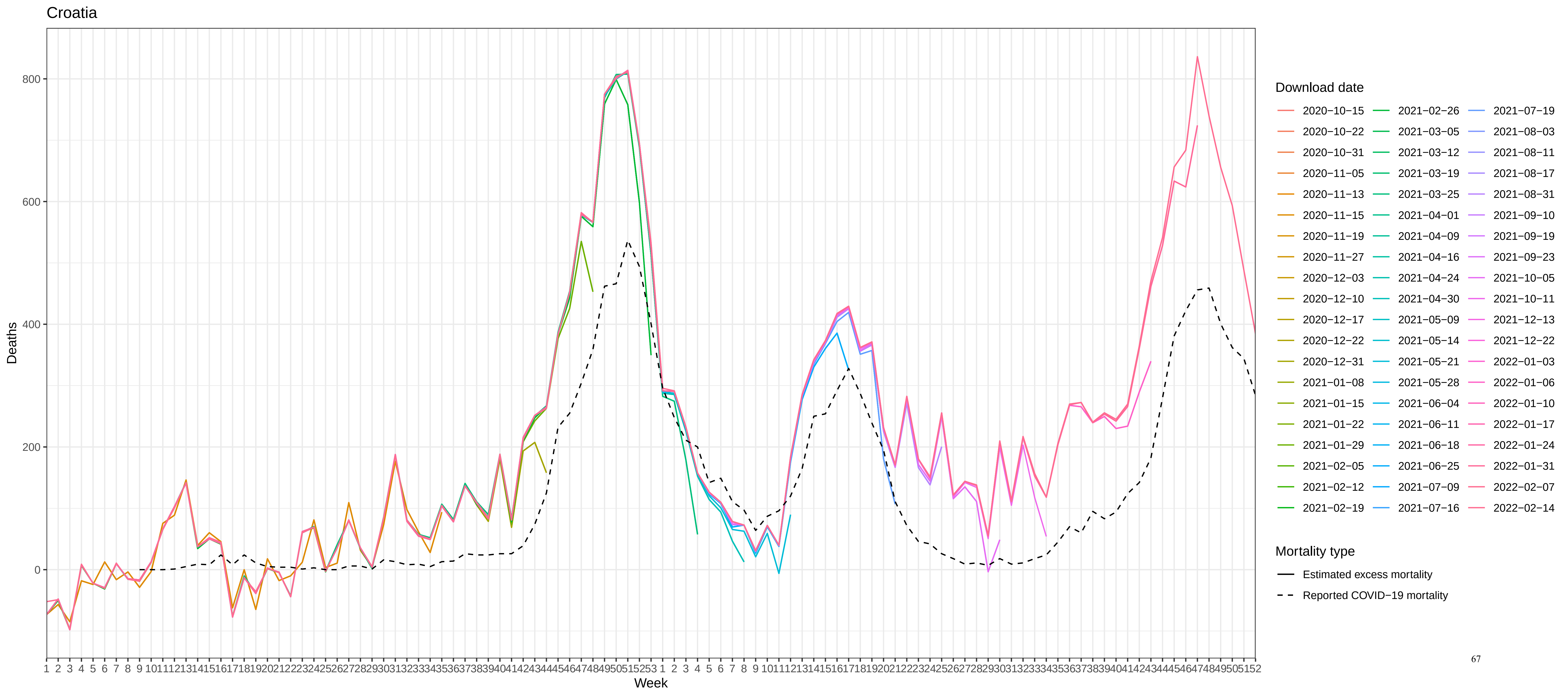

Croatia
Deaths
Week
Download date
Mortality type
Estimated excess mortality
Reported COVID−19 mortality

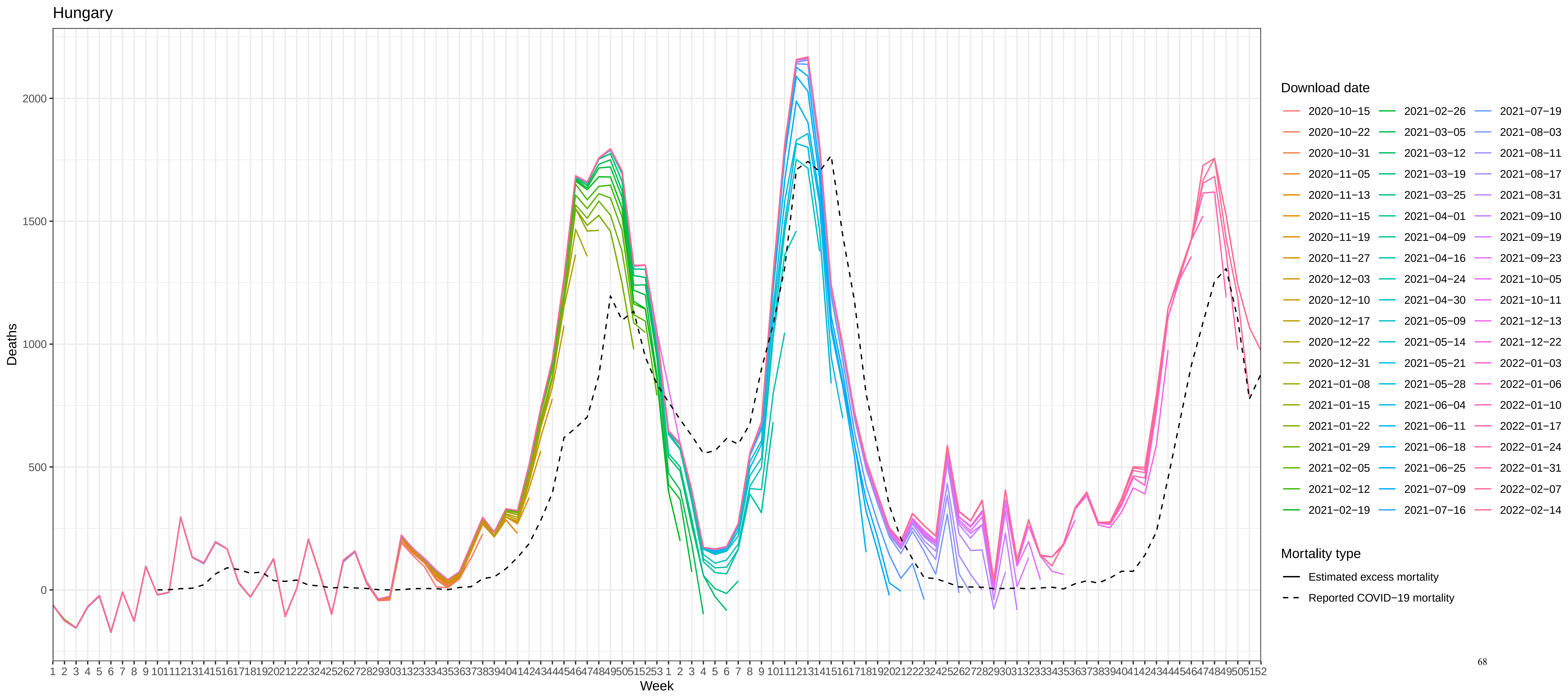
Hungary
Deaths
2000
1500
1000
500
0
Week
Download date
2020−10−15
2020−10−22
2020−10−31
2020−11−05
2020−11−13
2020−11−15
2020−11−19
2020−11−27
2020−12−03
2020−12−10
2020−12−17
2020−12−22
2020−12−31
2021−01−08
2021−01−15
2021−01−22
2021−01−29
2021−02−05
2021−02−12
2021−02−19
2021−02−26
2021−03−05
2021−03−12
2021−03−19
2021−03−25
2021−04−01
2021−04−09
2021−04−16
2021−04−24
2021−04−30
2021−05−09
2021−05−14
2021−05−21
2021−05−28
2021−06−04
2021−06−11
2021−06−18
2021−06−25
2021−07−09
2021−07−16
2021−07−19
2021−08−03
2021−08−11
2021−08−17
2021−08−31
2021−09−10
2021−09−19
2021−09−23
2021−10−05
2021−10−11
2021−12−13
2021−12−22
2022−01−03
2022−01−06
2022−01−10
2022−01−17
2022−01−24
2022−01−31
2022−02−07
2022−02−14
Mortality type
Estimated excess mortality
Reported COVID−19 mortality

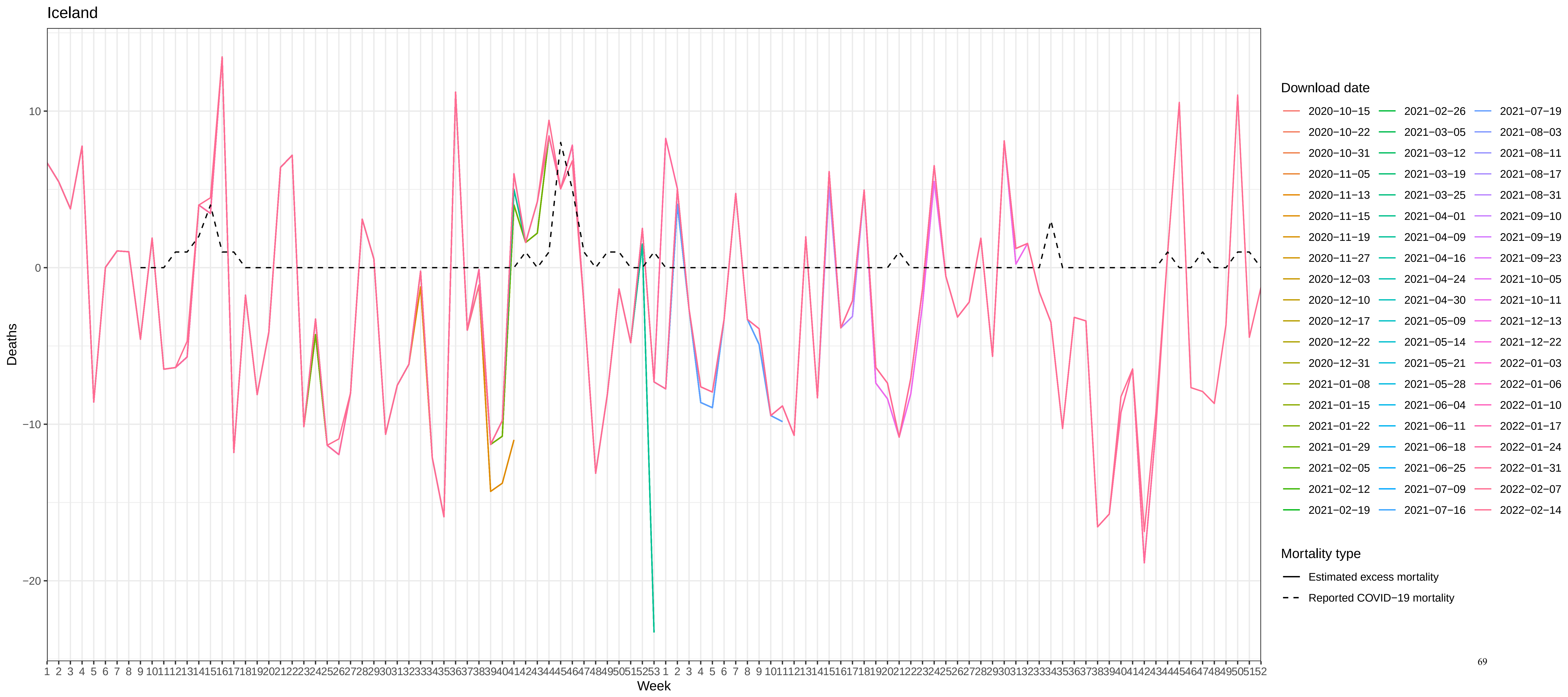
Iceland
Deaths
10
0
−10
−20
Week
Download date
2020−10−15
2020−10−22
2020−10−31
2020−11−05
2020−11−13
2020−11−15
2020−11−19
2020−11−27
2020−12−03
2020−12−10
2020−12−17
2020−12−22
2020−12−31
2021−01−08
2021−01−15
2021−01−22
2021−01−29
2021−02−05
2021−02−12
2021−02−19
2021−02−26
2021−03−05
2021−03−12
2021−03−19
2021−03−25
2021−04−01
2021−04−09
2021−04−16
2021−04−24
2021−04−30
2021−05−09
2021−05−14
2021−05−21
2021−05−28
2021−06−04
2021−06−11
2021−06−18
2021−06−25
2021−07−09
2021−07−16
2021−07−19
2021−08−03
2021−08−11
2021−08−17
2021−08−31
2021−09−10
2021−09−19
2021−09−23
2021−10−05
2021−10−11
2021−12−13
2021−12−22
2022−01−03
2022−01−06
2022−01−10
2022−01−17
2022−01−24
2022−01−31
2022−02−07
2022−02−14
Mortality type
Estimated excess mortality
Reported COVID−19 mortality

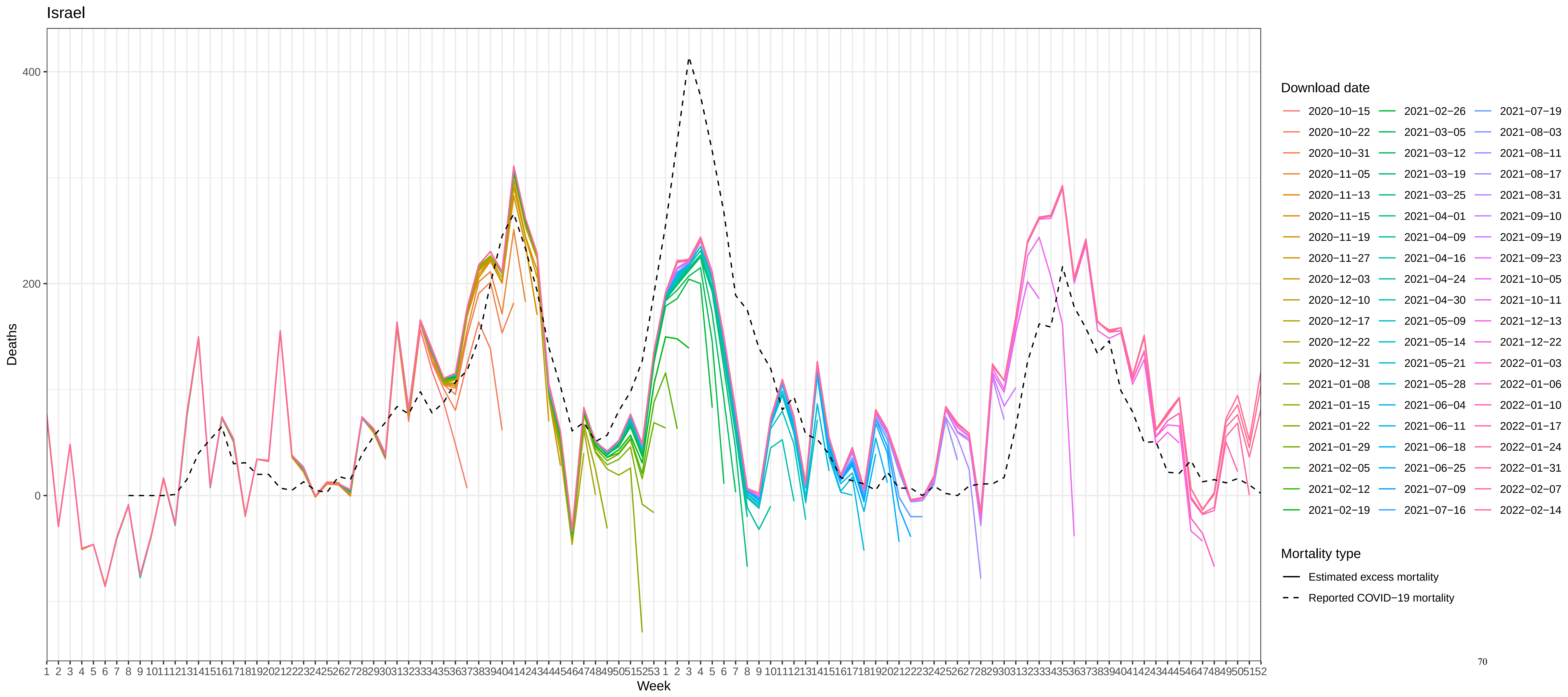
Israel
Deaths
400
200
0
Week
Download date
2020−10−15
2020−10−22
2020−10−31
2020−11−05
2020−11−13
2020−11−15
2020−11−19
2020−11−27
2020−12−03
2020−12−10
2020−12−17
2020−12−22
2020−12−31
2021−01−08
2021−01−15
2021−01−22
2021−01−29
2021−02−05
2021−02−12
2021−02−19
2021−02−26
2021−03−05
2021−03−12
2021−03−19
2021−03−25
2021−04−01
2021−04−09
2021−04−16
2021−04−24
2021−04−30
2021−05−09
2021−05−14
2021−05−21
2021−05−28
2021−06−04
2021−06−11
2021−06−18
2021−06−25
2021−07−09
2021−07−16
2021−07−19
2021−08−03
2021−08−11
2021−08−17
2021−08−31
2021−09−10
2021−09−19
2021−09−23
2021−10−05
2021−10−11
2021−12−13
2021−12−22
2022−01−03
2022−01−06
2022−01−10
2022−01−17
2022−01−24
2022−01−31
2022−02−07
2022−02−14
Mortality type
Estimated excess mortality
Reported COVID−19 mortality

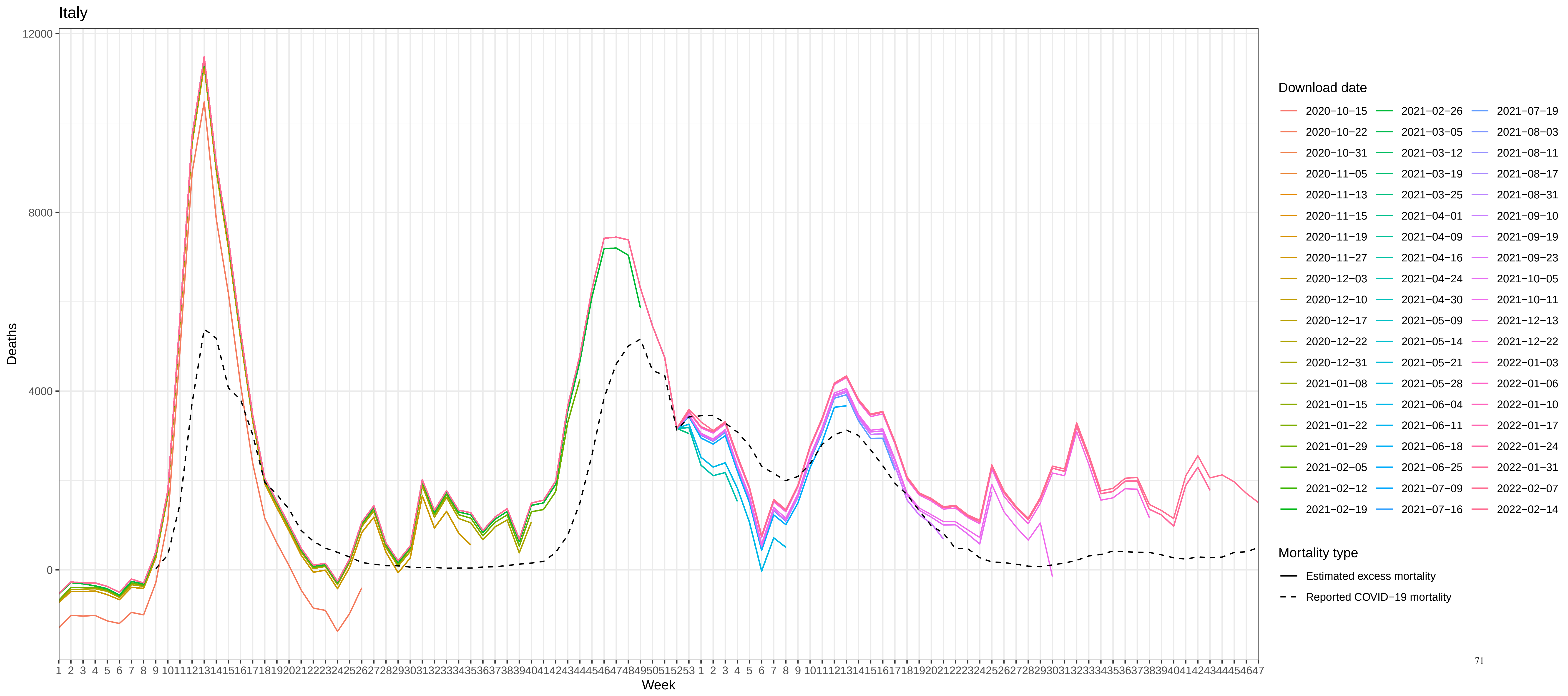
Italy
Deaths
Week
12000
8000
4000
0
Download date
2020−10−15
2020−10−22
2020−10−31
2020−11−05
2020−11−13
2020−11−15
2020−11−19
2020−11−27
2020−12−03
2020−12−10
2020−12−17
2020−12−22
2020−12−31
2021−01−08
2021−01−15
2021−01−22
2021−01−29
2021−02−05
2021−02−12
2021−02−19
2021−02−26
2021−03−05
2021−03−12
2021−03−19
2021−03−25
2021−04−01
2021−04−09
2021−04−16
2021−04−24
2021−04−30
2021−05−09
2021−05−14
2021−05−21
2021−05−28
2021−06−04
2021−06−11
2021−06−18
2021−06−25
2021−07−09
2021−07−16
2021−07−19
2021−08−03
2021−08−11
2021−08−17
2021−08−31
2021−09−10
2021−09−19
2021−09−23
2021−10−05
2021−10−11
2021−12−13
2021−12−22
2022−01−03
2022−01−06
2022−01−10
2022−01−17
2022−01−24
2022−01−31
2022−02−07
2022−02−14
Mortality type
Estimated excess mortality
Reported COVID−19 mortality

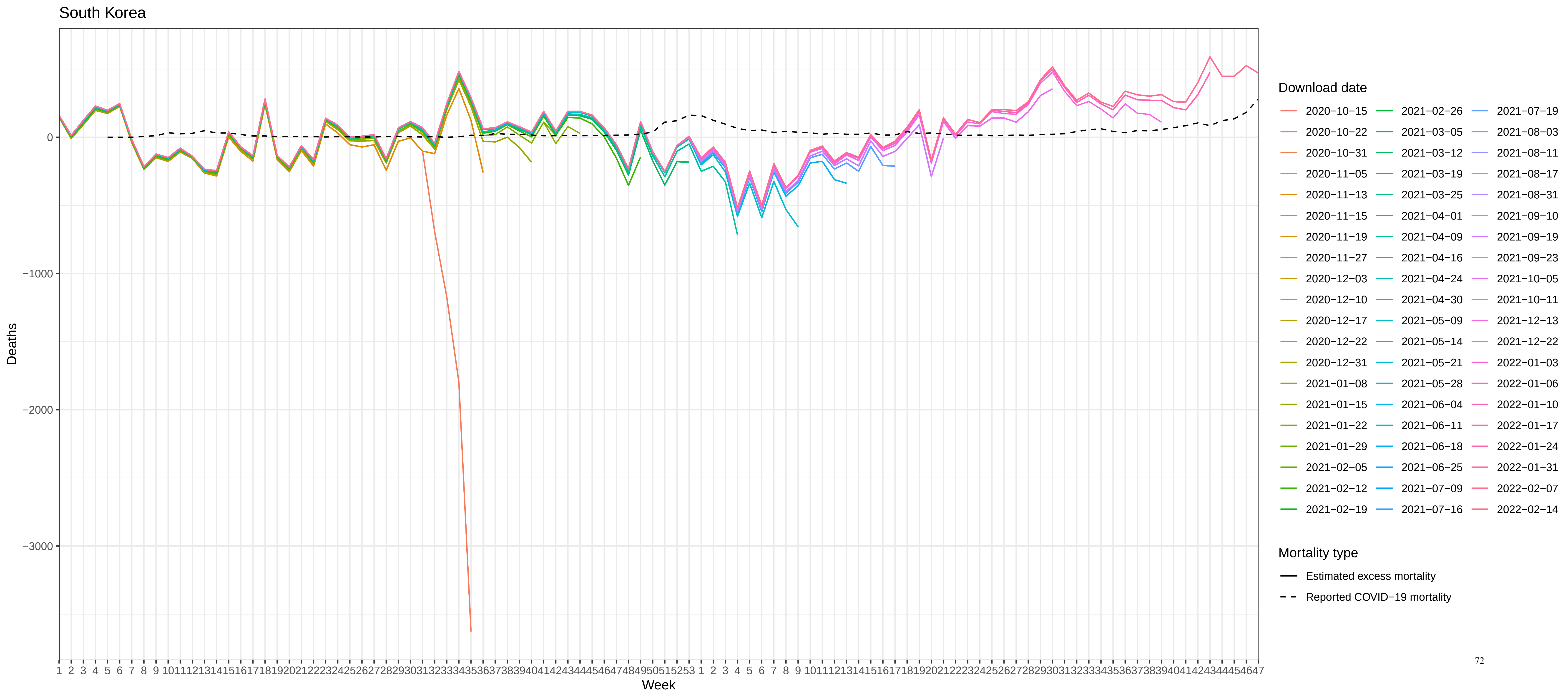
South Korea
Deaths
Week
Download date
2020−10−15
2020−10−22
2020−10−31
2020−11−05
2020−11−13
2020−11−15
2020−11−19
2020−11−27
2020−12−03
2020−12−10
2020−12−17
2020−12−22
2020−12−31
2021−01−08
2021−01−15
2021−01−22
2021−01−29
2021−02−05
2021−02−12
2021−02−19
2021−02−26
2021−03−05
2021−03−12
2021−03−19
2021−03−25
2021−04−01
2021−04−09
2021−04−16
2021−04−24
2021−04−30
2021−05−09
2021−05−14
2021−05−21
2021−05−28
2021−06−04
2021−06−11
2021−06−18
2021−06−25
2021−07−09
2021−07−16
2021−07−19
2021−08−03
2021−08−11
2021−08−17
2021−08−31
2021−09−10
2021−09−19
2021−09−23
2021−10−05
2021−10−11
2021−12−13
2021−12−22
2022−01−03
2022−01−06
2022−01−10
2022−01−17
2022−01−24
2022−01−31
2022−02−07
2022−02−14
Mortality type
Estimated excess mortality
Reported COVID−19 mortality

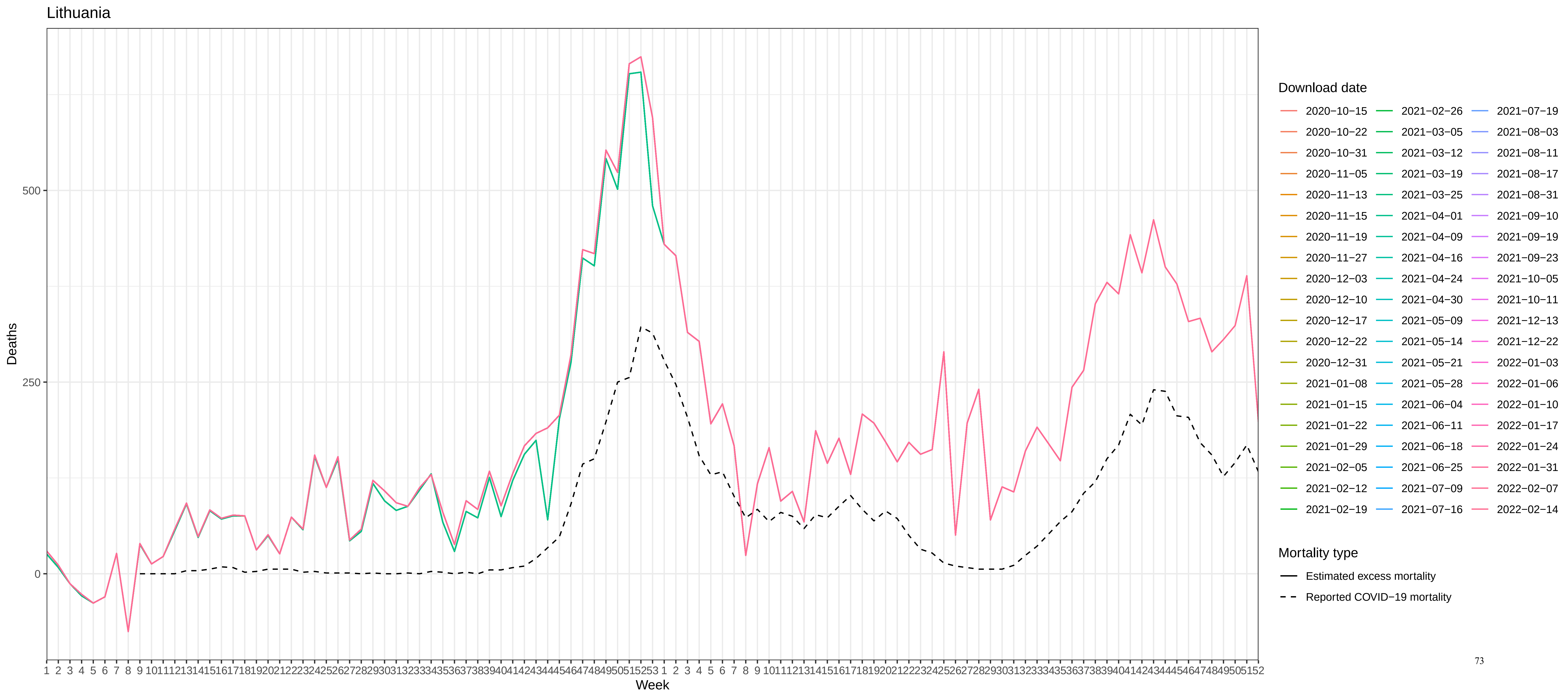

Lithuania
Deaths
500
250
0
Week
Download date
2020−10−15
2020−10−22
2020−10−31
2020−11−05
2020−11−13
2020−11−15
2020−11−19
2020−11−27
2020−12−03
2020−12−10
2020−12−17
2020−12−22
2020−12−31
2021−01−08
2021−01−15
2021−01−22
2021−01−29
2021−02−05
2021−02−12
2021−02−19
2021−02−26
2021−03−05
2021−03−12
2021−03−19
2021−03−25
2021−04−01
2021−04−09
2021−04−16
2021−04−24
2021−04−30
2021−05−09
2021−05−14
2021−05−21
2021−05−28
2021−06−04
2021−06−11
2021−06−18
2021−06−25
2021−07−09
2021−07−16
2021−07−19
2021−08−03
2021−08−11
2021−08−17
2021−08−31
2021−09−10
2021−09−19
2021−09−23
2021−10−05
2021−10−11
2021−12−13
2021−12−22
2022−01−03
2022−01−06
2022−01−10
2022−01−17
2022−01−24
2022−01−31
2022−02−07
2022−02−14
Mortality type
Estimated excess mortality
Reported COVID−19 mortality

Luxembourg

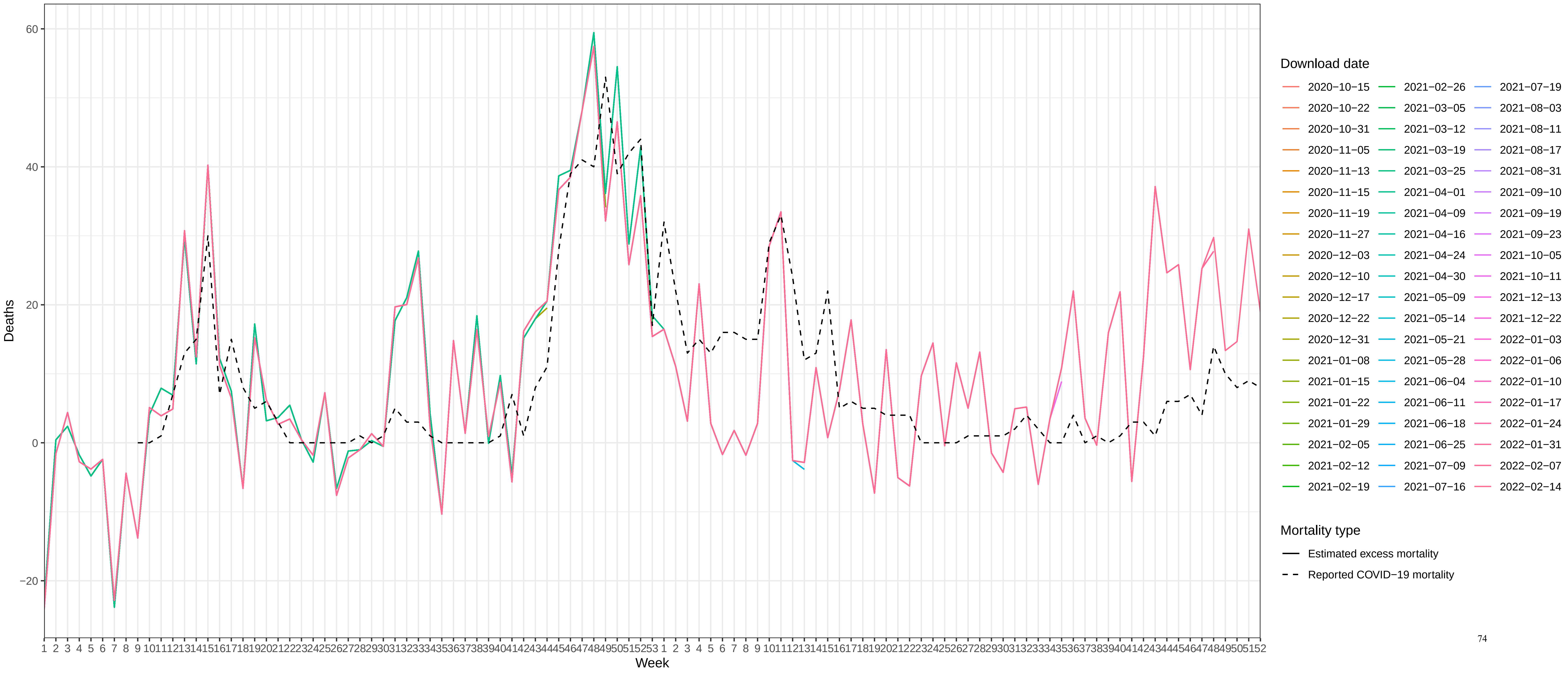
Deaths
Week
Download date
2020−10−15
2020−10−22
2020−10−31
2020−11−05
2020−11−13
2020−11−15
2020−11−19
2020−11−27
2020−12−03
2020−12−10
2020−12−17
2020−12−22
2020−12−31
2021−01−08
2021−01−15
2021−01−22
2021−01−29
2021−02−05
2021−02−12
2021−02−19
2021−02−26
2021−03−05
2021−03−12
2021−03−19
2021−03−25
2021−04−01
2021−04−09
2021−04−16
2021−04−24
2021−04−30
2021−05−09
2021−05−14
2021−05−21
2021−05−28
2021−06−04
2021−06−11
2021−06−18
2021−06−25
2021−07−09
2021−07−16
2021−07−19
2021−08−03
2021−08−11
2021−08−17
2021−08−31
2021−09−10
2021−09−19
2021−09−23
2021−10−05
2021−10−11
2021−12−13
2021−12−22
2022−01−03
2022−01−06
2022−01−10
2022−01−17
2022−01−24
2022−01−31
2022−02−07
2022−02−14
Mortality type
Estimated excess mortality
Reported COVID−19 mortality

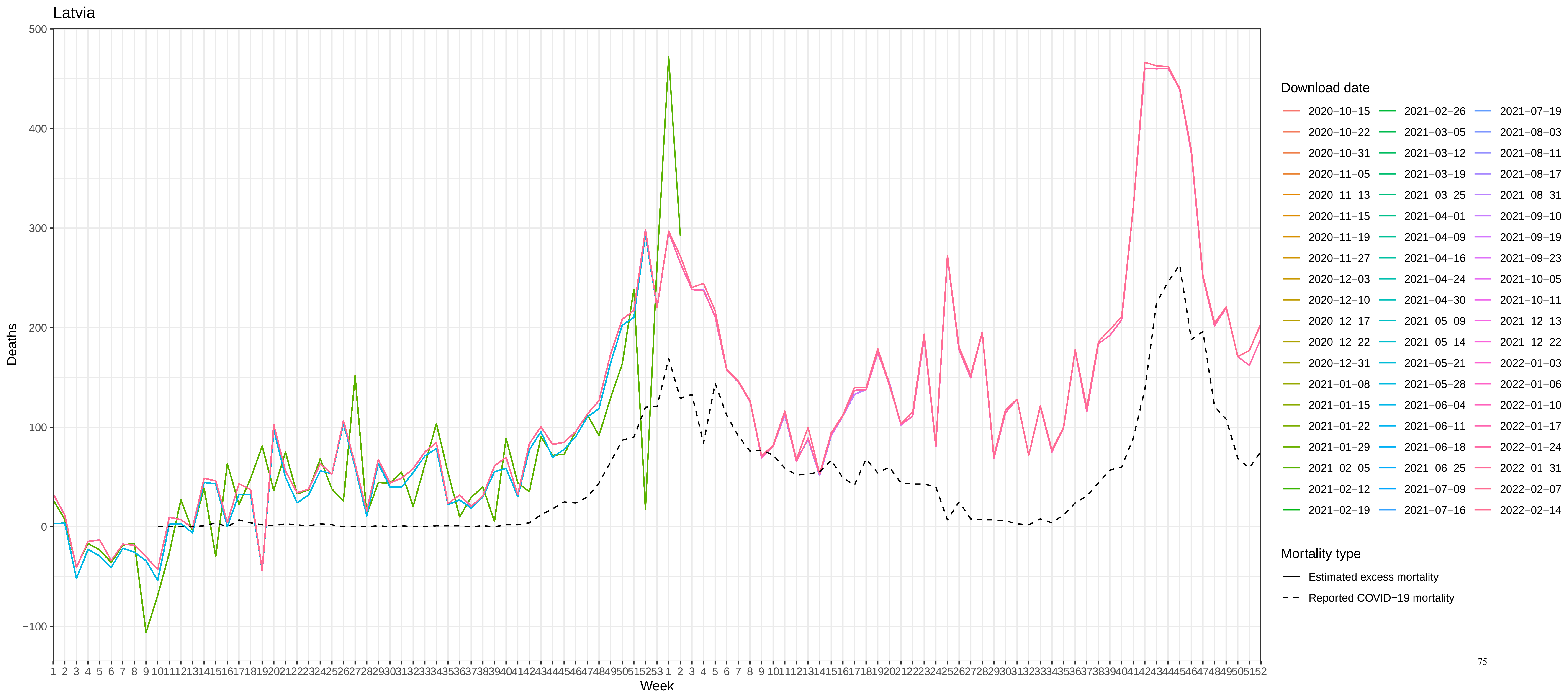
Latvia
Deaths
500
400
300
200
100
0
−100
Week
Download date
2020−10−15
2020−10−22
2020−10−31
2020−11−05
2020−11−13
2020−11−15
2020−11−19
2020−11−27
2020−12−03
2020−12−10
2020−12−17
2020−12−22
2020−12−31
2021−01−08
2021−01−15
2021−01−22
2021−01−29
2021−02−05
2021−02−12
2021−02−19
2021−02−26
2021−03−05
2021−03−12
2021−03−19
2021−03−25
2021−04−01
2021−04−09
2021−04−16
2021−04−24
2021−04−30
2021−05−09
2021−05−14
2021−05−21
2021−05−28
2021−06−04
2021−06−11
2021−06−18
2021−06−25
2021−07−09
2021−07−16
2021−07−19
2021−08−03
2021−08−11
2021−08−17
2021−08−31
2021−09−10
2021−09−19
2021−09−23
2021−10−05
2021−10−11
2021−12−13
2021−12−22
2022−01−03
2022−01−06
2022−01−10
2022−01−17
2022−01−24
2022−01−31
2022−02−07
2022−02−14
Mortality type
Estimated excess mortality
Reported COVID−19 mortality

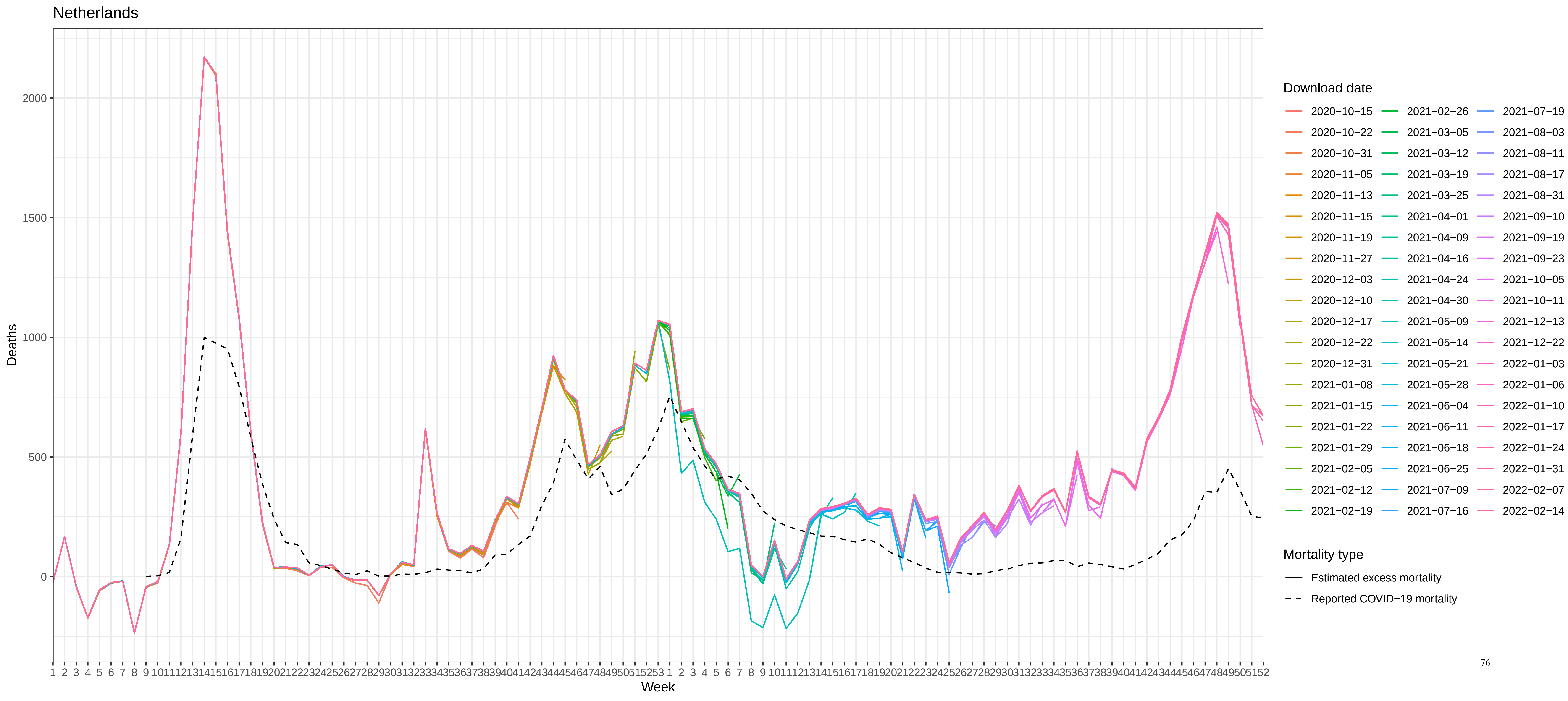
Netherlands
Deaths
Week
Download date
2020−10−15
2020−10−22
2020−10−31
2020−11−05
2020−11−13
2020−11−15
2020−11−19
2020−11−27
2020−12−03
2020−12−10
2020−12−17
2020−12−22
2020−12−31
2021−01−08
2021−01−15
2021−01−22
2021−01−29
2021−02−05
2021−02−12
2021−02−19
2021−02−26
2021−03−05
2021−03−12
2021−03−19
2021−03−25
2021−04−01
2021−04−09
2021−04−16
2021−04−24
2021−04−30
2021−05−09
2021−05−14
2021−05−21
2021−05−28
2021−06−04
2021−06−11
2021−06−18
2021−06−25
2021−07−09
2021−07−16
2021−07−19
2021−08−03
2021−08−11
2021−08−17
2021−08−31
2021−09−10
2021−09−19
2021−09−23
2021−10−05
2021−10−11
2021−12−13
2021−12−22
2022−01−03
2022−01−06
2022−01−10
2022−01−17
2022−01−24
2022−01−31
2022−02−07
2022−02−14
Mortality type
Estimated excess mortality
Reported COVID−19 mortality

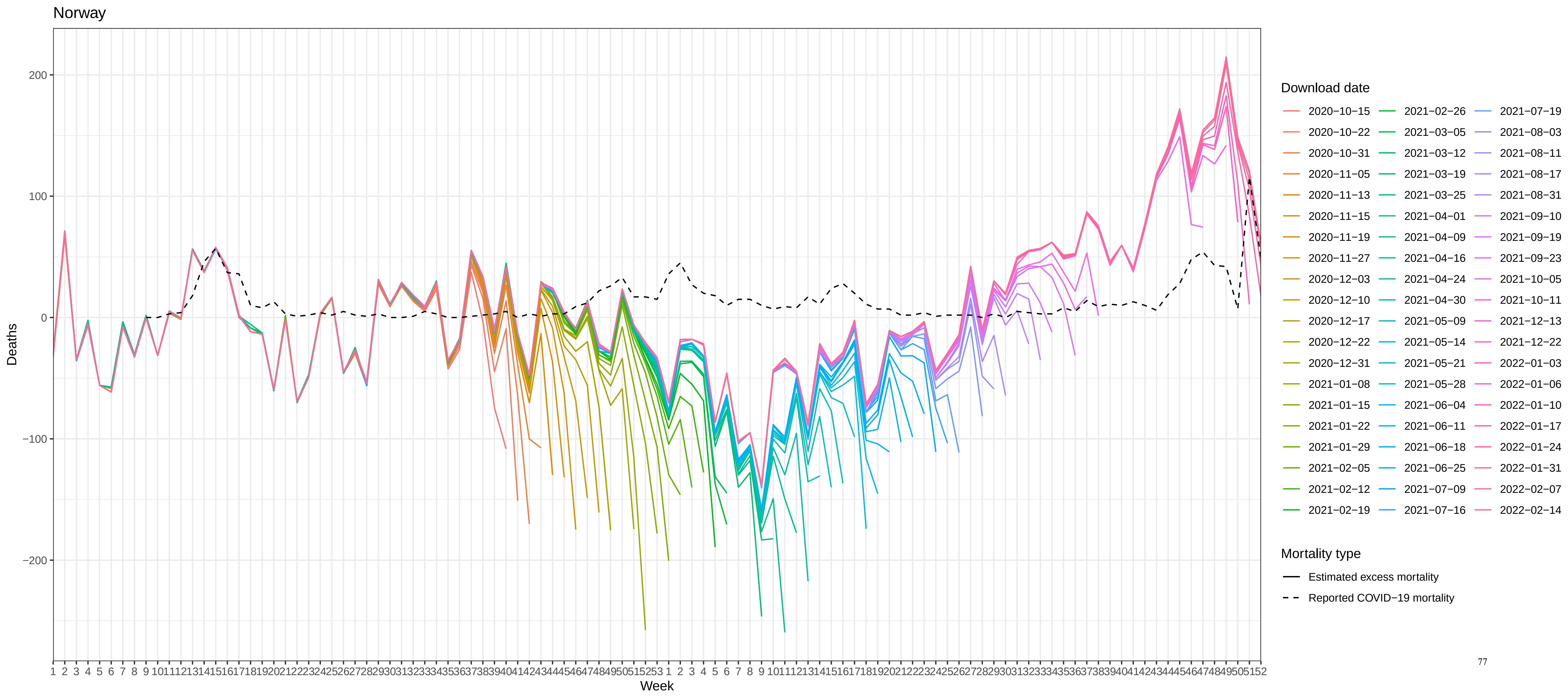
Norway
Deaths
200
100
0
−100
−200
Week
Download date
2020−10−15
2020−10−22
2020−10−31
2020−11−05
2020−11−13
2020−11−15
2020−11−19
2020−11−27
2020−12−03
2020−12−10
2020−12−17
2020−12−22
2020−12−31
2021−01−08
2021−01−15
2021−01−22
2021−01−29
2021−02−05
2021−02−12
2021−02−19
2021−02−26
2021−03−05
2021−03−12
2021−03−19
2021−03−25
2021−04−01
2021−04−09
2021−04−16
2021−04−24
2021−04−30
2021−05−09
2021−05−14
2021−05−21
2021−05−28
2021−06−04
2021−06−11
2021−06−18
2021−06−25
2021−07−09
2021−07−16
2021−07−19
2021−08−03
2021−08−11
2021−08−17
2021−08−31
2021−09−10
2021−09−19
2021−09−23
2021−10−05
2021−10−11
2021−12−13
2021−12−22
2022−01−03
2022−01−06
2022−01−10
2022−01−17
2022−01−24
2022−01−31
2022−02−07
2022−02−14
Mortality type
Estimated excess mortality
Reported COVID−19 mortality

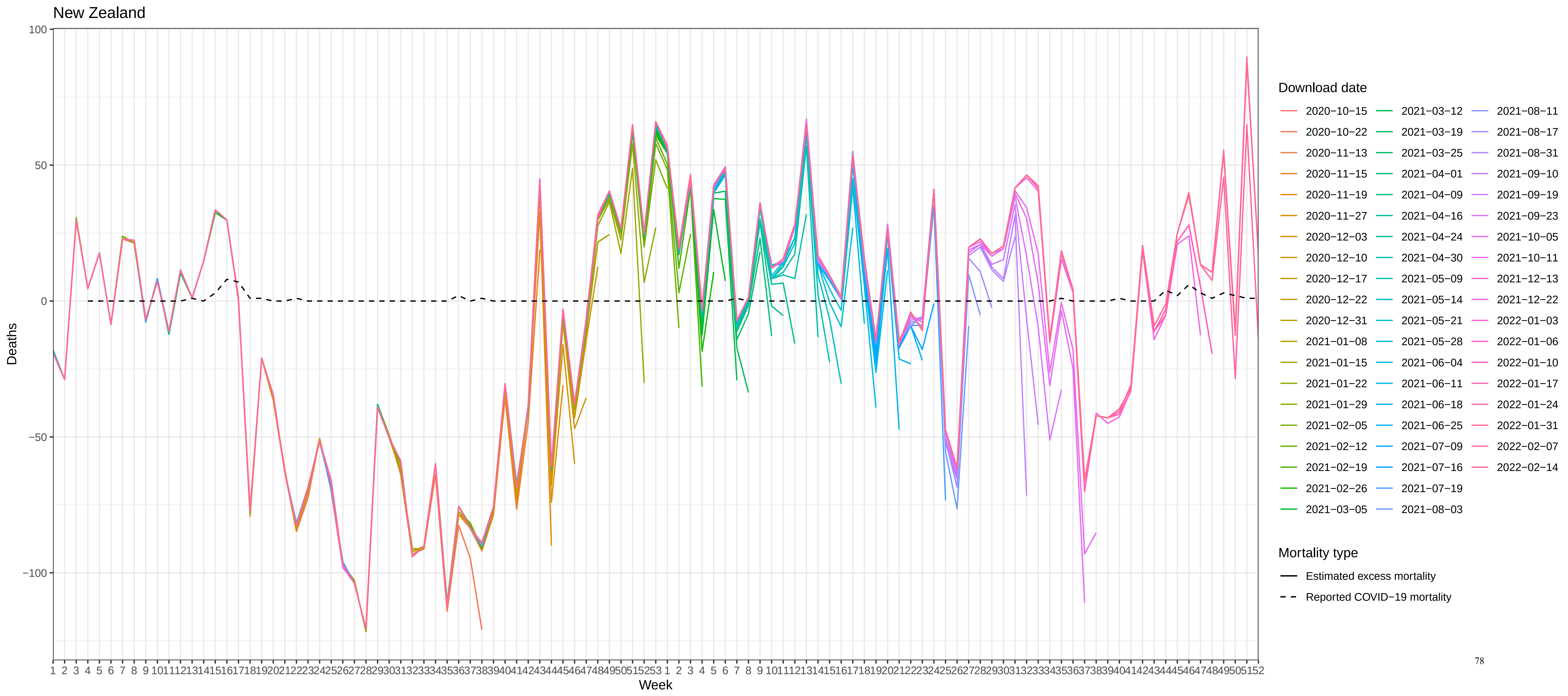
New Zealand
Deaths
Week
Download date
2020−10−15
2020−10−22
2020−11−13
2020−11−15
2020−11−19
2020−11−27
2020−12−03
2020−12−10
2020−12−17
2020−12−22
2020−12−31
2021−01−08
2021−01−15
2021−01−22
2021−01−29
2021−02−05
2021−02−12
2021−02−19
2021−02−26
2021−03−05
2021−03−12
2021−03−19
2021−03−25
2021−04−01
2021−04−09
2021−04−16
2021−04−24
2021−04−30
2021−05−09
2021−05−14
2021−05−21
2021−05−28
2021−06−04
2021−06−11
2021−06−18
2021−06−25
2021−07−09
2021−07−16
2021−07−19
2021−08−03
2021−08−11
2021−08−17
2021−08−31
2021−09−10
2021−09−19
2021−09−23
2021−10−05
2021−10−11
2021−12−13
2021−12−22
2022−01−03
2022−01−06
2022−01−10
2022−01−17
2022−01−24
2022−01−31
2022−02−07
2022−02−14
Mortality type
Estimated excess mortality
Reported COVID−19 mortality

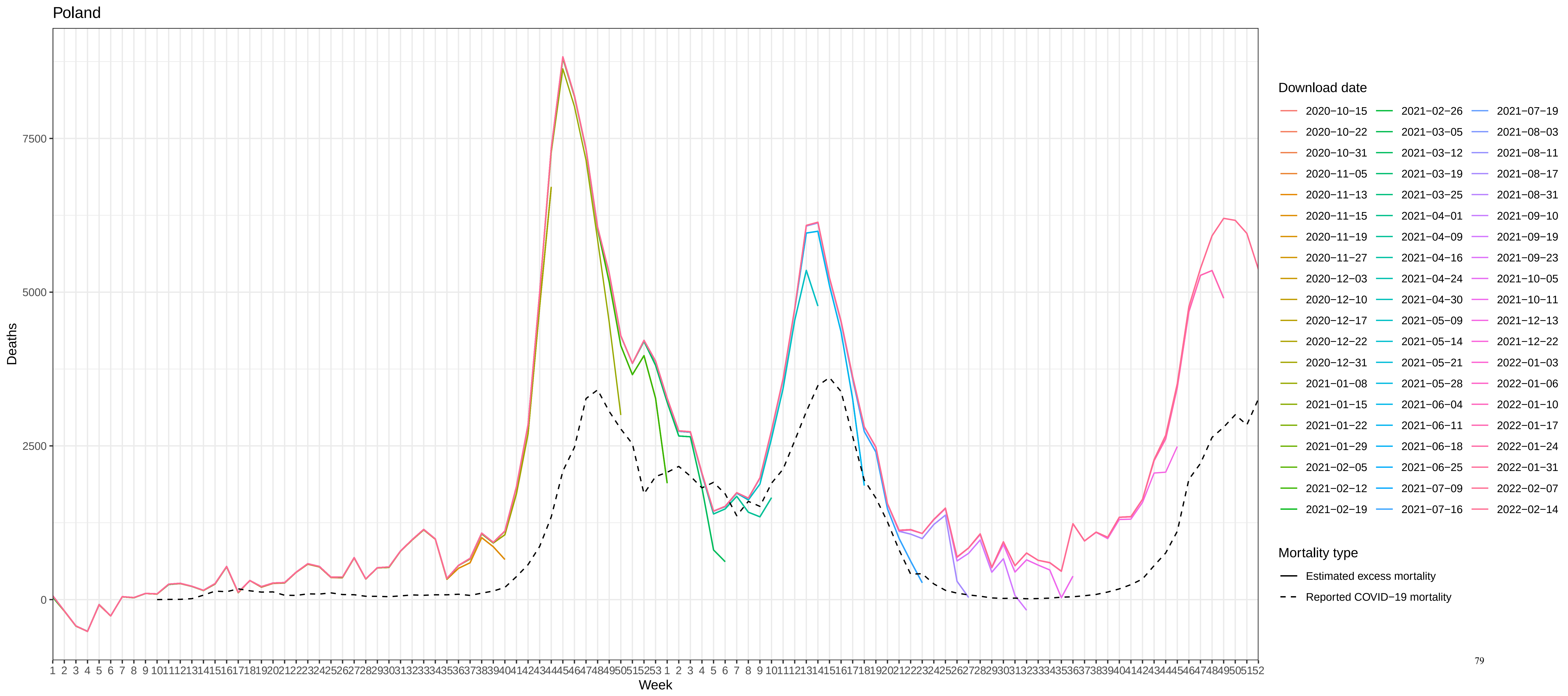
Poland
Deaths
Week
Download date
2020−10−15
2020−10−22
2020−10−31
2020−11−05
2020−11−13
2020−11−15
2020−11−19
2020−11−27
2020−12−03
2020−12−10
2020−12−17
2020−12−22
2020−12−31
2021−01−08
2021−01−15
2021−01−22
2021−01−29
2021−02−05
2021−02−12
2021−02−19
2021−02−26
2021−03−05
2021−03−12
2021−03−19
2021−03−25
2021−04−01
2021−04−09
2021−04−16
2021−04−24
2021−04−30
2021−05−09
2021−05−14
2021−05−21
2021−05−28
2021−06−04
2021−06−11
2021−06−18
2021−06−25
2021−07−09
2021−07−16
2021−07−19
2021−08−03
2021−08−11
2021−08−17
2021−08−31
2021−09−10
2021−09−19
2021−09−23
2021−10−05
2021−10−11
2021−12−13
2021−12−22
2022−01−03
2022−01−06
2022−01−10
2022−01−17
2022−01−24
2022−01−31
2022−02−07
2022−02−14
Mortality type
Estimated excess mortality
Reported COVID−19 mortality

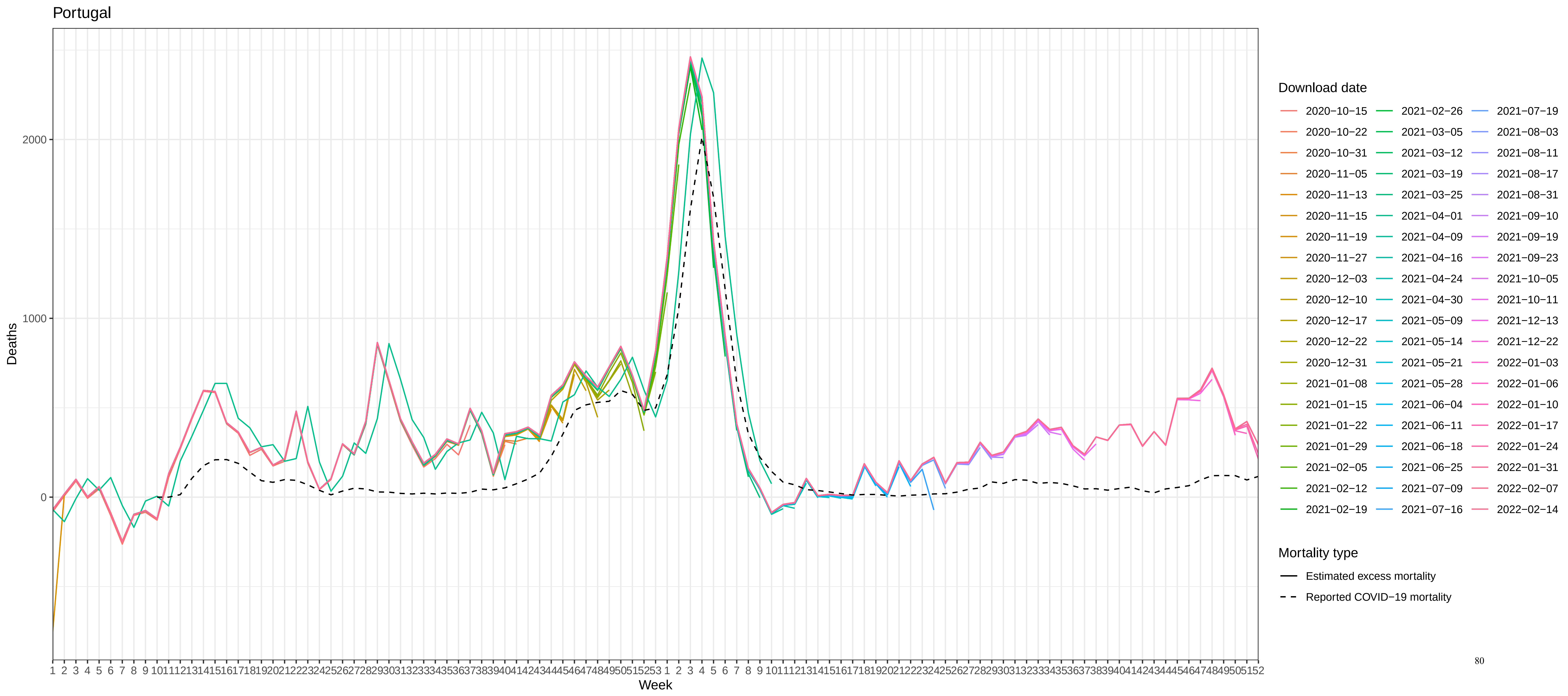
Portugal
Deaths
2000
1000
0
Week
Download date
2020−10−15
2020−10−22
2020−10−31
2020−11−05
2020−11−13
2020−11−15
2020−11−19
2020−11−27
2020−12−03
2020−12−10
2020−12−17
2020−12−22
2020−12−31
2021−01−08
2021−01−15
2021−01−22
2021−01−29
2021−02−05
2021−02−12
2021−02−19
2021−02−26
2021−03−05
2021−03−12
2021−03−19
2021−03−25
2021−04−01
2021−04−09
2021−04−16
2021−04−24
2021−04−30
2021−05−09
2021−05−14
2021−05−21
2021−05−28
2021−06−04
2021−06−11
2021−06−18
2021−06−25
2021−07−09
2021−07−16
2021−07−19
2021−08−03
2021−08−11
2021−08−17
2021−08−31
2021−09−10
2021−09−19
2021−09−23
2021−10−05
2021−10−11
2021−12−13
2021−12−22
2022−01−03
2022−01−06
2022−01−10
2022−01−17
2022−01−24
2022−01−31
2022−02−07
2022−02−14
Mortality type
Estimated excess mortality
Reported COVID−19 mortality

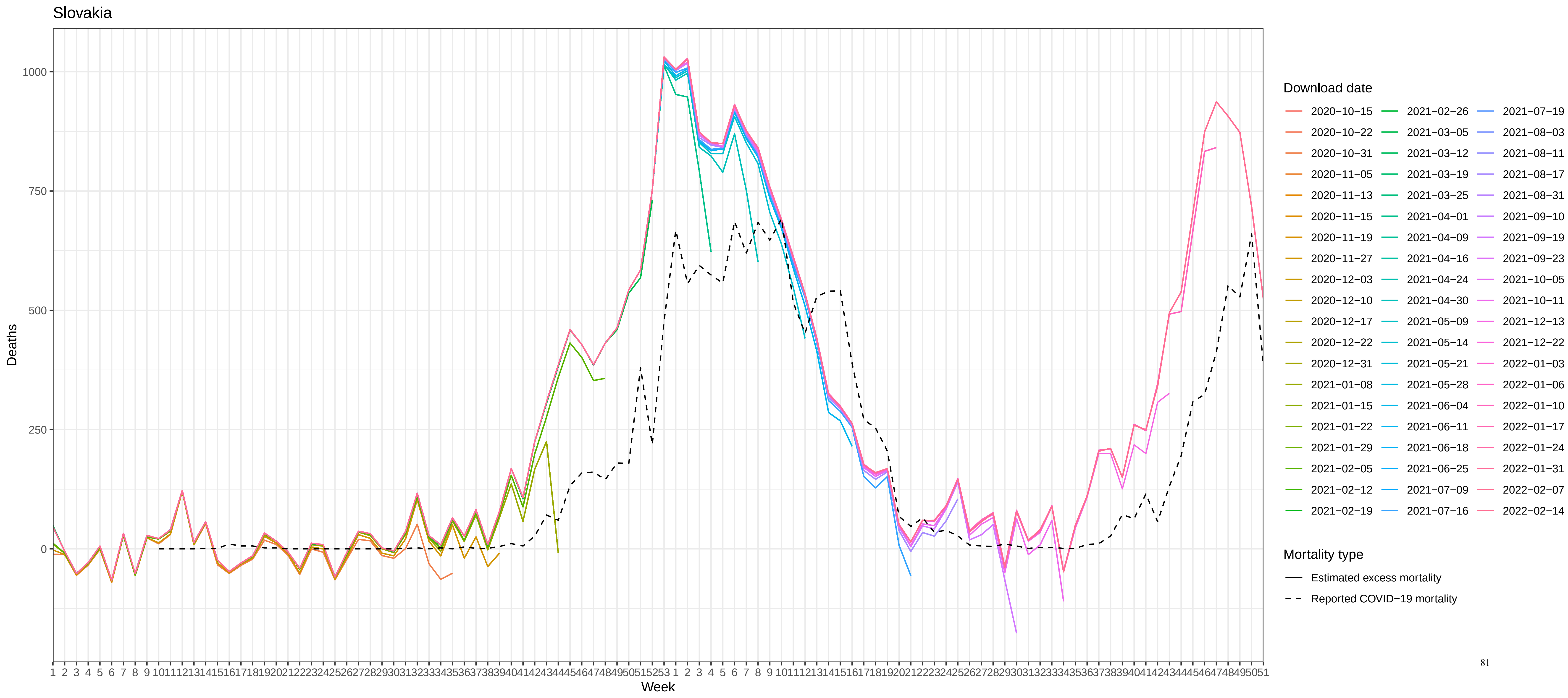
Slovakia
Deaths
Week
Download date
2020−10−15
2020−10−22
2020−10−31
2020−11−05
2020−11−13
2020−11−15
2020−11−19
2020−11−27
2020−12−03
2020−12−10
2020−12−17
2020−12−22
2020−12−31
2021−01−08
2021−01−15
2021−01−22
2021−01−29
2021−02−05
2021−02−12
2021−02−19
2021−02−26
2021−03−05
2021−03−12
2021−03−19
2021−03−25
2021−04−01
2021−04−09
2021−04−16
2021−04−24
2021−04−30
2021−05−09
2021−05−14
2021−05−21
2021−05−28
2021−06−04
2021−06−11
2021−06−18
2021−06−25
2021−07−09
2021−07−16
2021−07−19
2021−08−03
2021−08−11
2021−08−17
2021−08−31
2021−09−10
2021−09−19
2021−09−23
2021−10−05
2021−10−11
2021−12−13
2021−12−22
2022−01−03
2022−01−06
2022−01−10
2022−01−17
2022−01−24
2022−01−31
2022−02−07
2022−02−14
Mortality type
Estimated excess mortality
Reported COVID−19 mortality

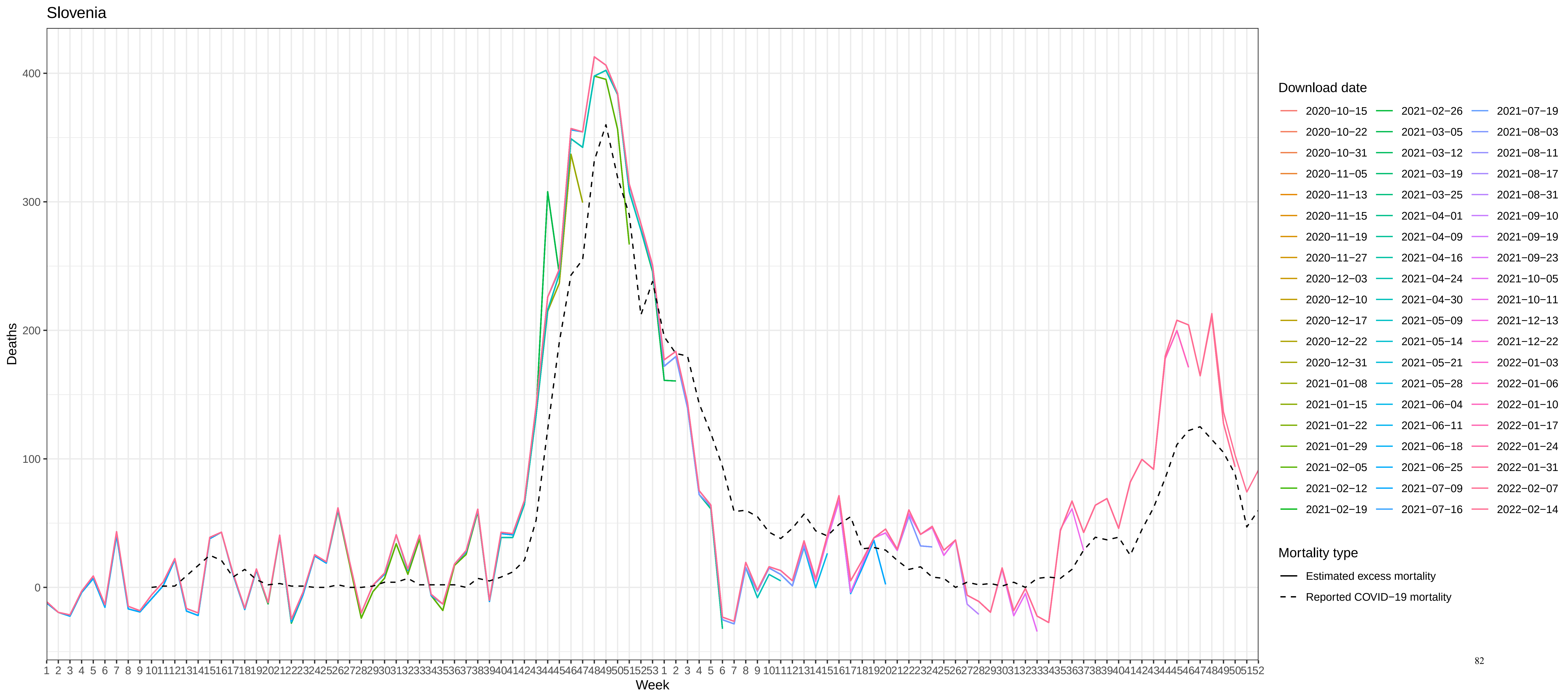
Slovenia
Deaths
Week
Download date
2020−10−15
2020−10−22
2020−10−31
2020−11−05
2020−11−13
2020−11−15
2020−11−19
2020−11−27
2020−12−03
2020−12−10
2020−12−17
2020−12−22
2020−12−31
2021−01−08
2021−01−15
2021−01−22
2021−01−29
2021−02−05
2021−02−12
2021−02−19
2021−02−26
2021−03−05
2021−03−12
2021−03−19
2021−03−25
2021−04−01
2021−04−09
2021−04−16
2021−04−24
2021−04−30
2021−05−09
2021−05−14
2021−05−21
2021−05−28
2021−06−04
2021−06−11
2021−06−18
2021−06−25
2021−07−09
2021−07−16
2021−07−19
2021−08−03
2021−08−11
2021−08−17
2021−08−31
2021−09−10
2021−09−19
2021−09−23
2021−10−05
2021−10−11
2021−12−13
2021−12−22
2022−01−03
2022−01−06
2022−01−10
2022−01−17
2022−01−24
2022−01−31
2022−02−07
2022−02−14
Mortality type
Estimated excess mortality
Reported COVID−19 mortality

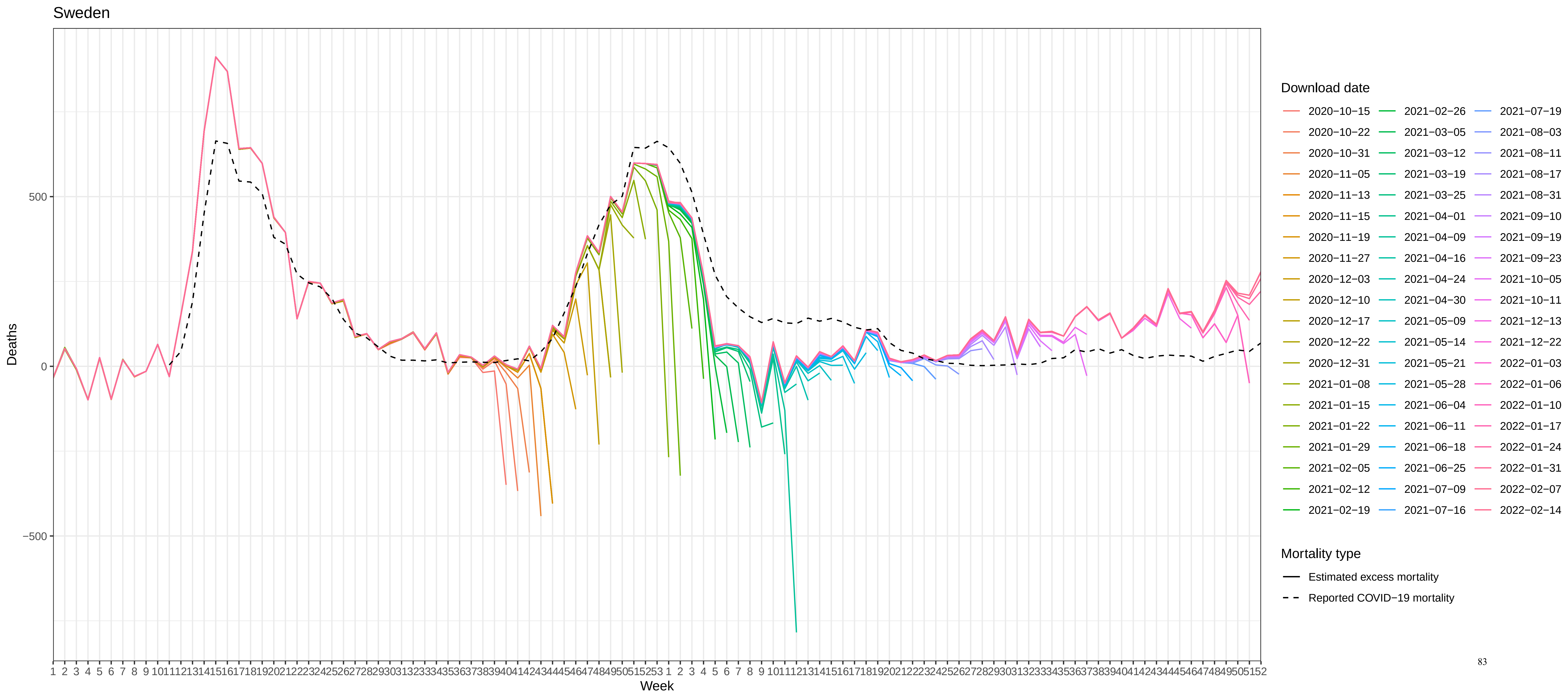
Sweden
Deaths
Week
500
0
−500
Download date
2020−10−15
2020−10−22
2020−10−31
2020−11−05
2020−11−13
2020−11−15
2020−11−19
2020−11−27
2020−12−03
2020−12−10
2020−12−17
2020−12−22
2020−12−31
2021−01−08
2021−01−15
2021−01−22
2021−01−29
2021−02−05
2021−02−12
2021−02−19
2021−02−26
2021−03−05
2021−03−12
2021−03−19
2021−03−25
2021−04−01
2021−04−09
2021−04−16
2021−04−24
2021−04−30
2021−05−09
2021−05−14
2021−05−21
2021−05−28
2021−06−04
2021−06−11
2021−06−18
2021−06−25
2021−07−09
2021−07−16
2021−07−19
2021−08−03
2021−08−11
2021−08−17
2021−08−31
2021−09−10
2021−09−19
2021−09−23
2021−10−05
2021−10−11
2021−12−13
2021−12−22
2022−01−03
2022−01−06
2022−01−10
2022−01−17
2022−01−24
2022−01−31
2022−02−07
2022−02−14
Mortality type
Estimated excess mortality
Reported COVID−19 mortality

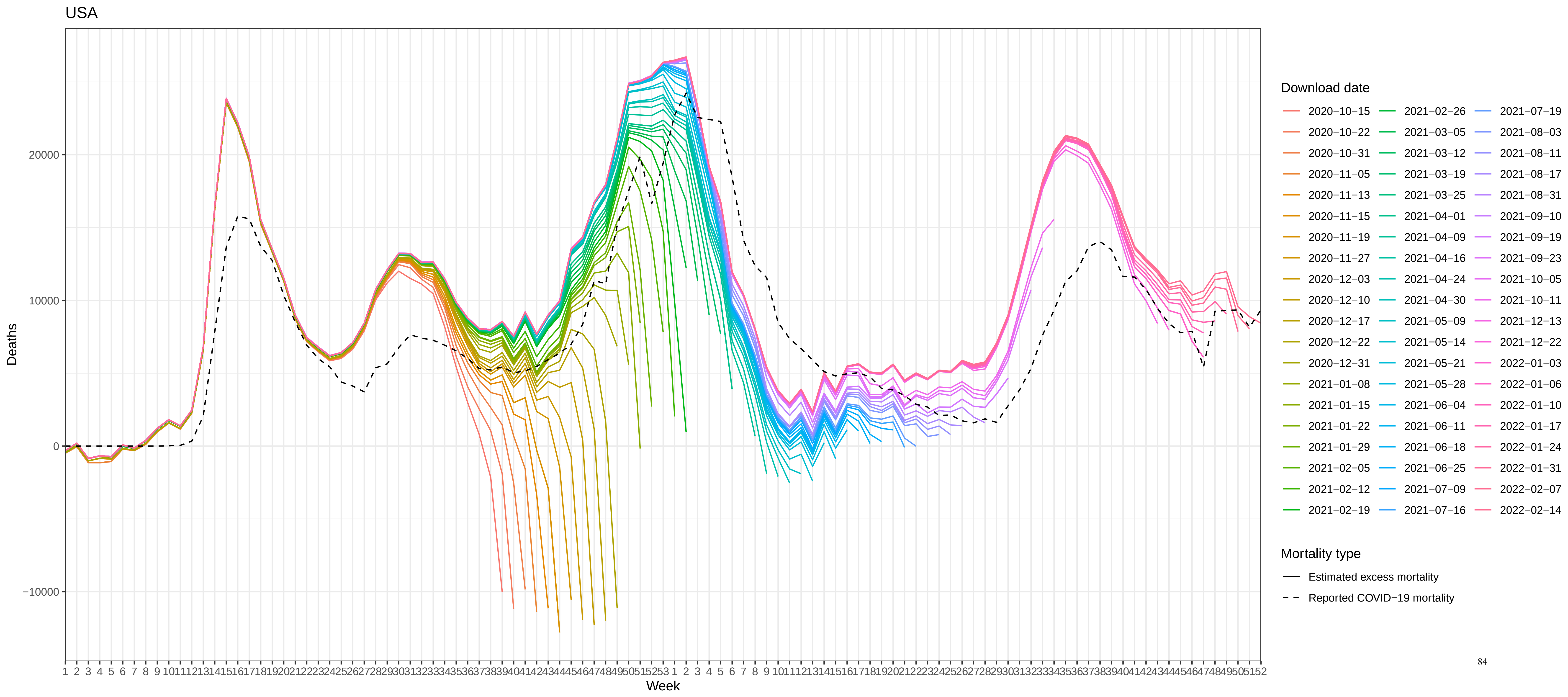
USA
Deaths
Week
Download date
2020−10−15
2020−10−22
2020−10−31
2020−11−05
2020−11−13
2020−11−15
2020−11−19
2020−11−27
2020−12−03
2020−12−10
2020−12−17
2020−12−22
2020−12−31
2021−01−08
2021−01−15
2021−01−22
2021−01−29
2021−02−05
2021−02−12
2021−02−19
2021−02−26
2021−03−05
2021−03−12
2021−03−19
2021−03−25
2021−04−01
2021−04−09
2021−04−16
2021−04−24
2021−04−30
2021−05−09
2021−05−14
2021−05−21
2021−05−28
2021−06−04
2021−06−11
2021−06−18
2021−06−25
2021−07−09
2021−07−16
2021−07−19
2021−08−03
2021−08−11
2021−08−17
2021−08−31
2021−09−10
2021−09−19
2021−09−23
2021−10−05
2021−10−11
2021−12−13
2021−12−22
2022−01−03
2022−01−06
2022−01−10
2022−01−17
2022−01−24
2022−01−31
2022−02−07
2022−02−14
Mortality type
Estimated excess mortality
Reported COVID−19 mortality
